# Supplementary material for: Modular ketal-linked prodrugs and biomaterials enabled by organocatalytic transisopropenylation of alcohols
Source: Nat Commun. 2021 Sep 20;12:5532. doi: 10.1038/s41467-021-25856-1 (PMC8452702; doi:10.1038/s41467-021-25856-1)
Supplement: Supplementary file 1 — Supplementary Information [file 41467_2021_25856_MOESM1_ESM.pdf]

Supplementary Information - Modular Ketal-Linked Prodrugs and Biomaterials Enabled by Organocatalytic Transisopropenylation of Alcohols

Na Yu,[1,2,#] Yang Xu,[1,#] Tao Liu,[1] Haiping Zhong,[1] Zunkai Xu,[1] Tianjiao Ji,[3] Hui Zou,[1] Jingqing Mu,[1] Ziqi Chen,[1] Xing-Jie Liang,[2,4] Linqi Shi,[1] Daniel S. Kohane,[3,*] Shutao Guo[1,*]

[1] Key Laboratory of Functional Polymer Materials of Ministry of Education, State Key Laboratory of Medicinal Chemical Biology and Institute of Polymer Chemistry, College of Chemistry, Nankai University, Tianjin 300071, P.R. China

[2] Translational Medicine Center, Key Laboratory of Molecular Target & Clinical Pharmacology, School of Pharmaceutical Sciences & The Second Affiliated Hospital, Guangzhou Medical University, Guangzhou 510260, P. R. China

[3] Laboratory for Biomaterials and Drug Delivery, Division of Critical Care Medicine, Children's Hospital Boston, Harvard Medical School, 300 Longwood Avenue, Boston, Massachusetts 02115, United States

[4] CAS Key Laboratory for Biological Effects of Nanomaterials and Nanosafety, National Center for Nanoscience and Technology, Beijing 100190, P.R. China

*Correspondence: stguo@nankai.edu.cn; daniel.kohane@childrens.harvard.edu

Table of Contents

1) ***Materials and Instrumentation*** ***3***
2) ***Preparation of Organocatalysts*** ***4***
3) ***Optimization of Organocatalytic Transisopropenylation Method*** ***5***
4) ***General Experimental Procedures for Organocatalytic Transisopropenylation*** ***9***
5) ***Ascertaining the Transisopropenylation Sites of Drugs*** ***11***
   Evaluation of Paclitaxel (PTX) 11
   Evaluation of Fulvestrant (FUL) 14
   Evaluation of Floxuridine (FUDR) 16
   Evaluation of Tafluprost (TAF) 18
   Evaluation of 7-Ethyl-10-hydroxycamptothecin (SN38) 19
   Evaluation of 7-Hydroxycoumarin (HDC) 21
   Evaluation of Estradiol (EST) 23
6) ***Synthesis of Drug-Derived Isopropenyl Ethers Using Other Methods*** ***24***
   Reported Methods for Synthesis of Isopropenyl Ethers 24
   Synthesis of PTX-Derived IPPE Using $[Ir(cod)Cl]_2/Na_2CO_3$ Catalytic Method 25
   Synthesis of PTX-Derived IPPE Using TMSOTf/DIPEA Method 29
   Synthesis of FUDR-Derived IPPE Using $[Ir(cod)Cl]_2/Na_2CO_3$ Catalytic Method 30
   Synthesis of FUDR-Derived IPPE Using TMSOTf/DIPEA Method 30
7) ***Experimental and Analytical Data for Isopropenyl Ethers*** ***31***
8) ***Elucidating Mechanism of Organocatalytic Transisopropenylation*** ***52***
   Monitoring the Reaction Process 52
   Organocatalytic Synthesis of 3a from 3a′ 52
   The Influence of Stoichiometric Ratios on Yield of IPPEs 53
9) ***Synthesis of Ketal-Linked Prodrugs*** ***55***
   Synthesis of HSA-K-DEX 55
   Synthesis of PEG-K-DEX 56
   Synthesis of LA-K-CAPME 57
   Synthesis of LA-K-BUF 57
   Synthesis of PTX-7-K-EG_3 58
   Synthesis of PK3F 59
   Synthesis of PK5F 60
   Synthesis of PK5E 62
10) ***In Vitro and In Vivo Characterizations of PTX Prodrugs*** ***65***
11) ***Synthesis of Ketal-Linked Biomaterials*** ***72***
12) ***NMR Spectra*** ***74***
13) ***Supplementary References*** ***153***

Supplementary Methods

1) Materials and Instrumentation

All reactions and procedures were performed using Schlenk techniques under an inert atmosphere of nitrogen. All reagents were purchased from commercial suppliers and used as received unless otherwise noted. Most of the alcohol substrates for the synthesis of isopropenyl ethers were commercially available. Alcohol substrates and 2-methoxy-1-alkenes that were not commercially available were prepared according to reported procedures. Isopropenyl acetate was distilled before use. Tetrahydrofuran (THF) and toluene were dried with sodium wire and distilled from benzophenone. Anhydrous dichloromethane (DCM) was freshly distilled from calcium hydride. Methoxy polyethylene glycol-poly(*D,L*-lactide) (mPEG-PDLLA, M_n = 5,000–3,000 g/mol) was purchased from Daigangbio Biomaterial Co., Ltd.

Reactions were monitored by thin-layer chromatography (TLC) using alkaline aluminum oxide or silica gel plates. Flash column chromatography was performed over silica gel (200-300 mesh). Reversed-phase column chromatography was performed by CombiFlash Rf Flash Chromatography Systems (Teledyne Isco, USA). ^{1}H NMR spectra were recorded at 400 MHz and ^{13}C NMR spectra were recorded at 100 MHz using Bruker AV 400 spectrometer. NMR spectra were recorded in $CDCl_3$, CD_3CN, D_2O or C_6D_6 as solvents. In some cases, a trace amount of Et_3N was added to prevent hydrolysis of enol ethers and ketals. NMR shifts were calibrated against residual $CHCl_3$ (δ 7.26, δ 77.0), ACN (δ 1.94, δ 118.0), H_2O (δ 4.79), and C_6H_6 (δ 7.16, δ 128.0), and were reported in ppm. Abbreviations for signal coupling are as follows: s, singlet; d, doublet; t, triplet; q, quartet; m, multiplet. Coupling constants were taken from the spectra directly and were uncorrected. HRMS spectra were recorded on Fourier transform ion cyclotron resonance mass spectrometer (Varian 7.0T FTMS, USA), Q-TOF LC/MS (Agilent 6520 Q-TOF LC/MS, USA) and GC-MS (Q Exactive GC Orbitrap, Thermo Scientific, USA). MALDI-TOF was measured on Bruker AutoflexIII LRF200-CID (Bruker, DEU). HPLC analyses were performed using Agilent 1260 instruments (Agilent, USA) with C18 column (Agilent, Poroshell 120 EC-C18, 4.6 mm × 100 mm, 2.7 µm). Samples were injected at a flow rate of 1.0 mL/min, column temperature at 25 ^{o}C, and UV detection wavelength at 227 nm. Diameters, polydispersity indexes and zeta potentials of micelles were measured using Zetasizer Nano ZS90 (Malvern Instruments Inc., UK). TEM samples were prepared on 300 mesh ultra-thin carbon film supported copper grids (Zhongjingkeyi Technology Co., Ltd., CHN), and were imaged using Talos F200C (ThermoFisher Scientific, Waltham, MA, USA). For CCK-8 assay, the absorbance at 450 nm was measured using SpectraMax i3x (Molecular Devices, CA, USA). Fluorescent images were captured using TCS SP8 confocal laser scanning microscope (CLSM) (Leica Microsystems, Wetzlar, GER). HCT116 human colon cancer cell line was purchased from ATCC (Manassas, VA, USA) and cultured in Dulbecco's modified Eagle's medium (DMEM) supplemented with 10% fetal bovine serum (FBS), 100 IU/mL penicillin and 100 µg/mL streptomycin in 5% CO_2 incubator at 37 ^{o}C. 3T3 murine

fibroblast cell line was purchased from ATCC (Manassas, VA, USA) and cultured in RPMI 1640 medium supplemented with 10% fetal bovine serum (FBS), 100 IU/mL penicillin and 100 μg/mL streptomycin in 5% CO_2 incubator at 37 ^{o}C. Female BALB/c nude mice (18-20 g) were purchased from Beijing Vital River Laboratory Animal Technology Co., Ltd. All experiments were carried out following the guidelines of the Beijing Laboratory Animal Center, and approved by the Ethical Commission at Nankai University.

2) Preparation of Organocatalysts

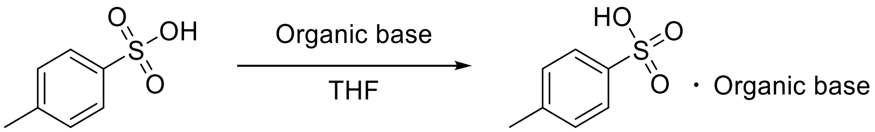

p-Toluenesulfonic acid monohydrate (1 g, 5.3 mmol) and organic base (5.3 mmol) were added into 10 mL THF in a round bottom flask. The mixture was stirred for 30 min to yield white precipitate, filtered, and washed with THF. At last, the white salt catalysts were dried by vacuum.

Pyridinium *p*-toluenesulfonate (PPTS)

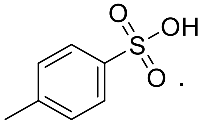

PPTS is commercially available and was ordered from commercial vendors.

2,6-Lutidinium *p*-toluenesulfonate (LPTS)

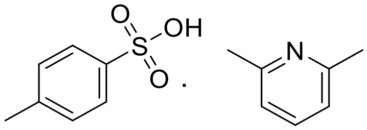

^{1}H NMR (400 MHz, D_2O) δ 8.18 (d, *J* = 7.9 Hz, 1H), 7.58 (dd, *J* = 30.6, 8.0 Hz, 4H), 7.30 (d, *J* = 7.9 Hz, 2H), 2.64 (s, 6H), 2.33 (s, 3H).

Morpholinium *p*-toluenesulfonate (MPTS)

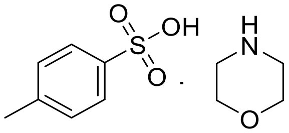

^{1}H NMR (400 MHz, D_2O) δ 7.64 (d, *J* = 8.2 Hz, 2H), 7.32 (d, *J* = 8.1 Hz, 2H), 3.95 – 3.86 (m, 4H), 3.28 – 3.20 (m, 4H), 2.35 (s, 3H).

Imidazolium *p*-toluenesulfonate (IPTS)

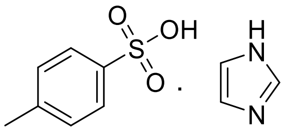

^{1}H NMR (400 MHz, D_2O) δ 8.62 (s, 1H), 7.64 (d, *J* = 8.1 Hz, 2H), 7.42 (s, 2H), 7.32 (d, *J* = 8.0 Hz, 2H), 2.34 (s, 3H).

Quinolinium *p*-toluenesulfonate (QPTS)

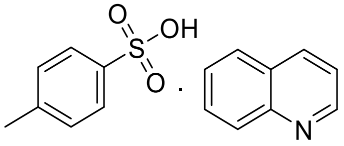

^{1}H NMR (400 MHz, D_2O) δ 9.03 (dd, *J* = 17.3, 6.8 Hz, 2H), 8.22 (d, *J* = 8.2 Hz, 1H), 8.11 (m, 2H), 7.93 (m, 2H), 7.58 (d, *J* = 8.0 Hz, 2H), 7.24 (d, *J* = 7.9 Hz, 2H), 2.29 (s, 3H).

4,4-Bipyridinium *p*-toluenesulfonate (BPPTS)

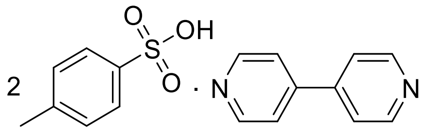

^{1}H NMR (400 MHz, D_2O) δ 8.78 (d, *J* = 5.6 Hz, 2H), 8.37 (d, *J* = 6.9 Hz, 4H), 7.84 (t, *J* = 6.0 Hz, 2H), 7.62 (d, *J* = 8.0 Hz, 4H), 7.30 (d, *J* = 7.9 Hz, 4H), 2.33 (s, 6H).

Triethylammonium *p*-toluenesulphonate (TPTS)

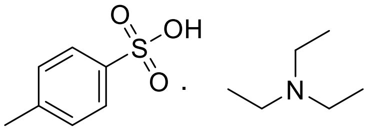

^{1}H NMR (400 MHz, D_2O) δ 7.64 (d, *J* = 8.3 Hz, 2H), 7.31 (d, *J* = 8.1 Hz, 2H), 3.11 (q, *J* = 7.3 Hz, 6H), 2.33 (s, 3H), 1.20 (t, *J* = 7.3 Hz, 9H).

3) Optimization of Organocatalytic Transisopropenylation Method

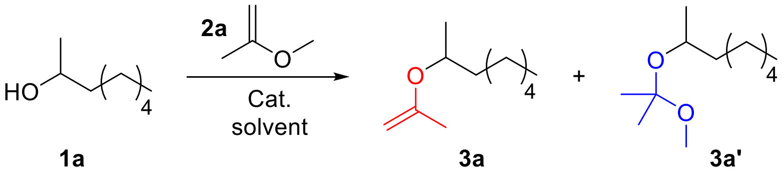

To a Schlenk flask (10 mL) was added substrate **1a** (1 mmol) and catalyst. Then solvent (2 mL) and **2a** was added to the flask, and stirred. After the reaction was completed, 0.2 mL Et_3N was

added to quench the reaction. Solvent and excess of **2a** were removed under reduced pressure. The residue was analyzed by ^{1}H NMR spectroscopy.

Supplementary Table 1. Acid evaluation

Entry	Acid	**3a/3a′**	Conversion (%)
1	Acetic acid	0/0	0
2	Chloroacetic acid	0/0	0
3	Dichloroacetic acid	0/4	4
4	Trichloroacetic acid	3/97	100
5	*p*-Toluenesulfonic acid	63/37	100
6	Triflic acid	52/48	100

Reaction condition: **1a** (0.5 M), **2a** (16 equiv.)**,** acid (0.5 mol%), 1 h, r.t., THF.

Almost no reaction occurred for weakly acidic acetic acid, chloroacetic acid, and dichloroacetic acid (entry 1–3). However, trichloroacetic acid, a strong acid, gave a high yield of MOP ketal **3a′**, and interestingly we noted the formation of IPPE **3a**, transformed from the oxocarbenium ion (entry 4). Surprisingly, *p*-toluenesulfonic acid (*p*TSA), an acid stronger than trichloroacetic acid, produced **3a** with a moderate yield (entry 5). However, for the *p*TSA-catalyzed reaction, a significant amount of side products that were dimer and oligomers of **2a** were also produced, caused by acid-initiated cationic polymerization of **2a**.[1] Nevertheless, the yield of **3a** was significantly decreased when triflic acid, stronger relative to *p*TSA, was used (entry 6).

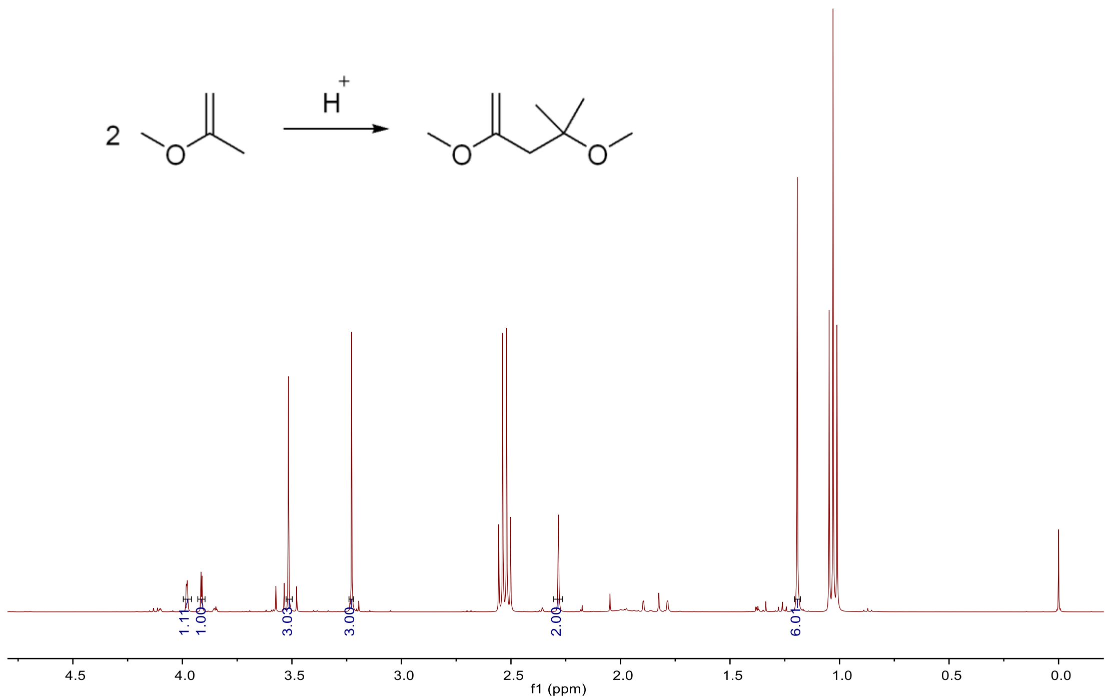

Supplementary Figure 1. ^{1}H NMR of **2a** dimer (400MHz, $CDCl_3$).

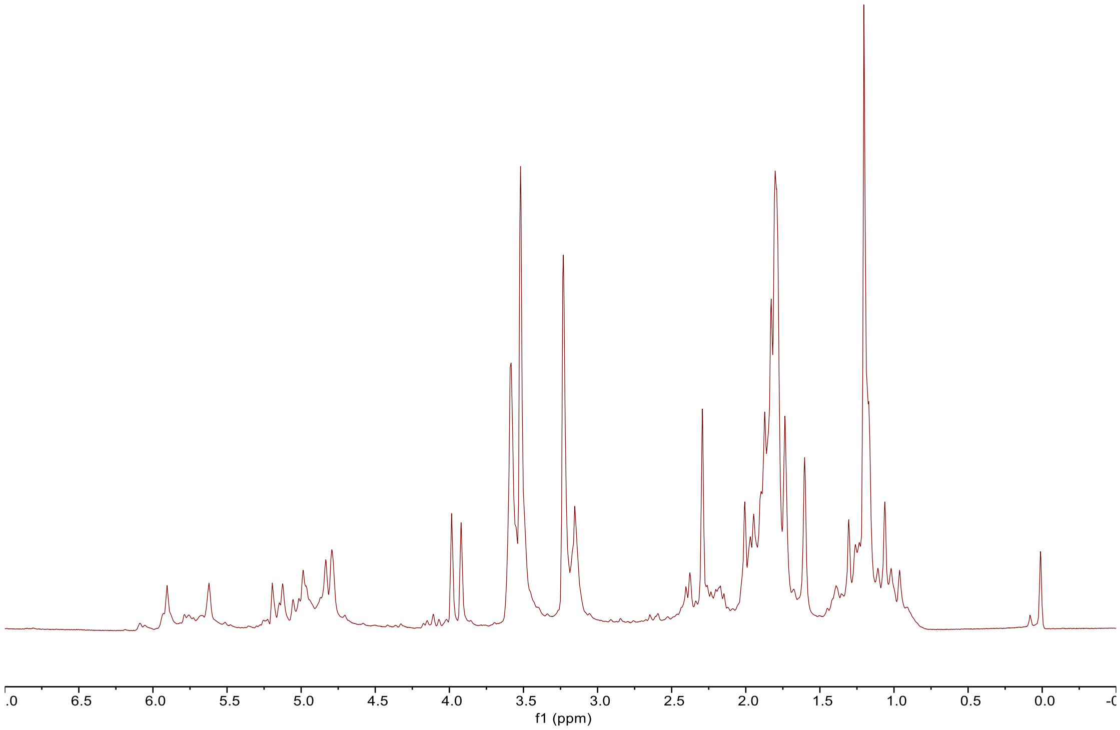

Supplementary Figure 2. ^{1}H NMR of **2a** oligomers (400MHz, $CDCl_3$).

Organocatalyst evaluation results were shown in Figure 1c in the main text. We found that side reactions nearly disappeared using *p*-toluenesulphonate LPTS as catalyst and THF as the solvent, and high yields of IPPEs were obtained.

Supplementary Table 2. Solvent evaluation

Entry	Solvent	**3a/3a′**	Conversion (%)
1	CH_2Cl_2	78/22	100
2	1,4-dioxane	77/23	100
3	THF	96/4	100
4	acetonitrile	76/24	100
5	ethyl acetate	93/7	100
6	acetone	85/15	100
7	toluene	60/40	100

Reaction condition: **1a** (0.5 M), **2a** (16 equiv.)**,** LPTS (0.5 mol%), 1 h, r.t.

Supplementary Table 3. 2-MPE equivalents evaluation

Entry	**2a** (equiv.)	**3a**/**3a′**	Conversion (%)
1	1	10/40	40
2	2	37/41	78
3	4	73/18	91
4	8	90/10	100
5	12	95/5	100
6	16	96/4	100
7	40	97/3	100

Conditions: **1a** (0.5 M), LPTS (0.5 mol%), 1 h, r.t., THF.

Supplementary Table 4. Reaction temperature evaluation

Entry	T (ºC)	**3a**/**3a′**	Conversion (%)
1	-20	0/100	100
2	0	60/40	100
3	15	94/6	100
4	25	96/4	100
5	40	96/4	100

Conditions: **1a** (0.5 M), **2a** (16 equiv.), LPTS (0.5 mol%), 1 h, THF.

Supplementary Table 5. Catalyst loading evaluation

Entry	LPTS (mol%)	**3a**/**3a′**	Conversion (%)
1	0.1	90/10	100
2	0.5	96/4	100
3	5	94/6	100
4	10	95/5	100

Conditions: **1a** (0.5 M), **2a** (16 equiv.), r.t., 1 h, THF.

Supplementary Table 6. Reaction time evaluation

Entry	t (h)	**3a**/**3a′**	Conversion (%)
1	0.017	40/60	100
2	0.5	92/8	100
3	1	96/4	100
4	4	96/4	100
5	8	94/6	100

Reaction condition: **1a** (0.5 M), **2a** (16 equiv.), LPTS (0.5 mol%), r.t., THF.

4) General Experimental Procedures for Organocatalytic Transisopropenylation

General Procedure A (Main Text Figure 2 and Supplementary Table 7)

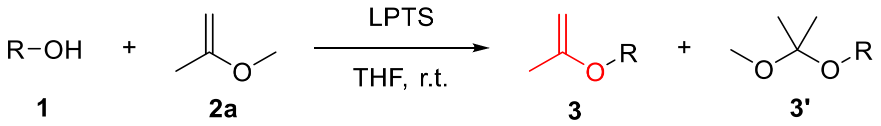

To a Schlenk flask (10 mL) was added substrate **1** (1 mmol) and LPTS. Then THF (2 mL) and **2a** (1.54 mL, 16 mmol, 16 equiv.) were added to the flask and stirred at room temperature. After the reaction was completed, 0.2 mL Et_3N was added to quench the reaction. Solvent and excess of **2a** were removed under reduced pressure. The resulting residue was purified by column chromatography or reversed-phase chromatography to give the desired products.

The substrate concentration is 0.5 M unless otherwise noted.

General Procedure B (Main Text Figure 3 and Supplementary Table 8)

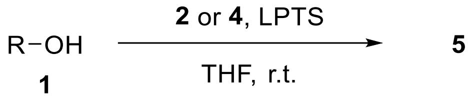

To a Schlenk flask (10 mL) was added substrate **1** (0.12 mmol) and LPTS (20 mol%). The flask was evacuated and refilled with N_2. Then THF (2 mL) and 2-methoxy-1-alkenes **2** or **4** (16 mmol, 16 equiv.) were added to the flask and stirred at room temperature for 24 h. After the reaction was complete, 0.2 mL Et_3N was added to quench the reaction. The solvent was removed under reduced pressure. The resulting residue was purified by column chromatography or reversed-phase chromatography to give the desired products.

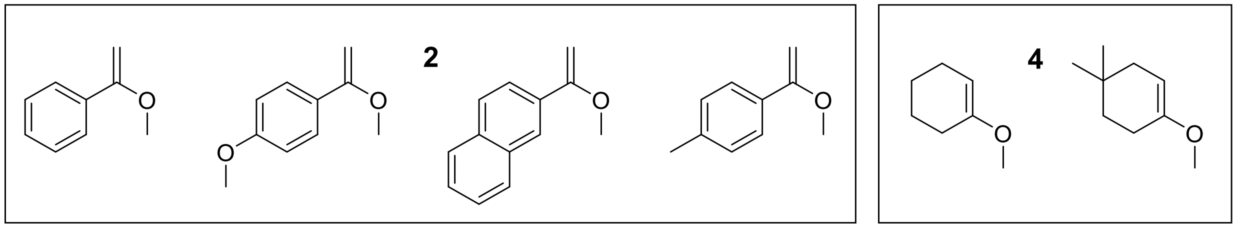

Supplementary Table 7. Synthetic scope of alcohols[a]

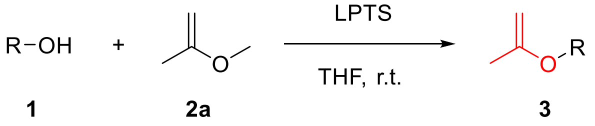

Primary alcohols

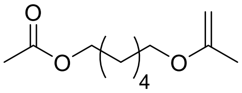

3ab: 77%[b]

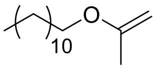

3ac: 89%

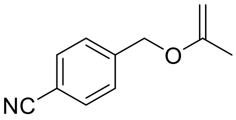

3ad: 81%[b]

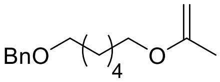

3ae: 75%[b]

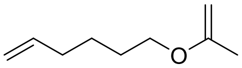

3af: 70%

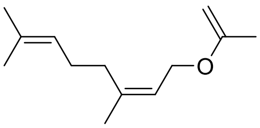

3ag: 83%

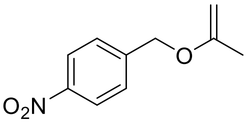

3ah: 60%[b]

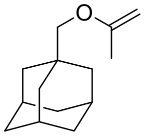

3ai: 58%[b]

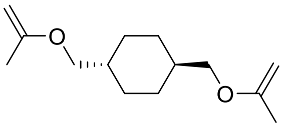

3aj: 58%

Secondary alcohols

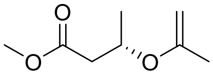

3ak: 85%[b]

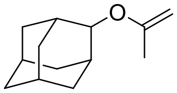

3al: 90%

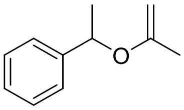

3am: 80%[b]

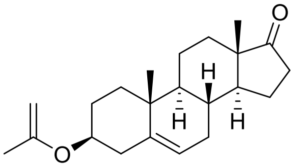

3an: 89%

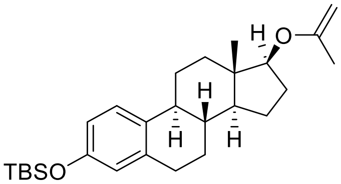

3ao: 87%

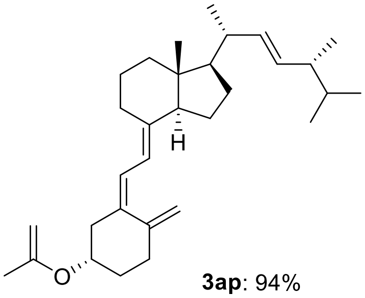

3ap: 94%

Tertiary alcohol

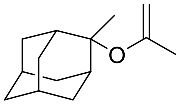

3aq: 60%[b]

[a] Reaction conditions: **1** (1.0 equiv.), **2a** (16.0 equiv.), 0.5 mol % LPTS, THF, 25 °C, 12 h. [b] 2.0 mol % LPTS, 24 h. Yields of isolated products are given.

Supplementary Table 8. Synthetic scope of enol ethers[a]

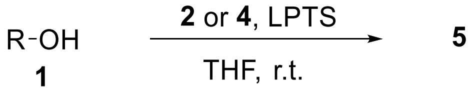

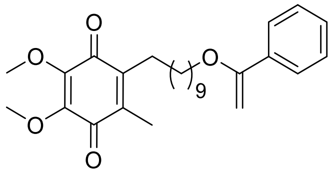

5g: 68%

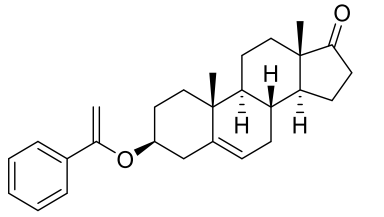

5h: 72%

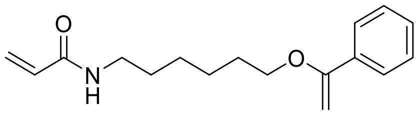

5i: 78%

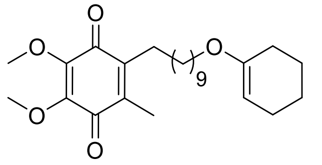

5j: 78%

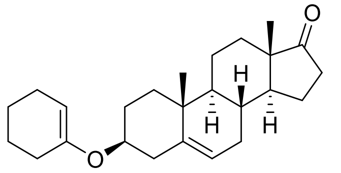

5k: 75%

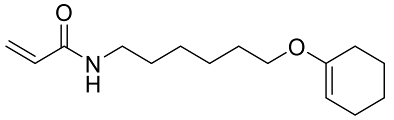

5l: 70%

[a] Reaction conditions: **1** (1.0 equiv.), **2** or **4** (16.0 equiv.), 20 mol % LPTS, THF, 25 °C, 24 h. Yields of isolated products are given.

5) Ascertaining the Transisopropenylation Sites of Drugs

For diol drugs, such as paclitaxel, fulvestrant, estradiol, floxuridine, and tafluprost, they were reacted with 2-MPE, and the products were analyzed using NMR spectroscopy to ascertain the sites of IPPE and MOP ketal formation. Some drugs with hindered tertiary alcohols and aromatic alcohols, such as 7-ethyl-10-hydroxycamptothecin and the fluorophore 7-hydroxycoumarin, were also examined, and we found phenols gave only the corresponding MOP ketals.

Evaluation of Paclitaxel (PTX)

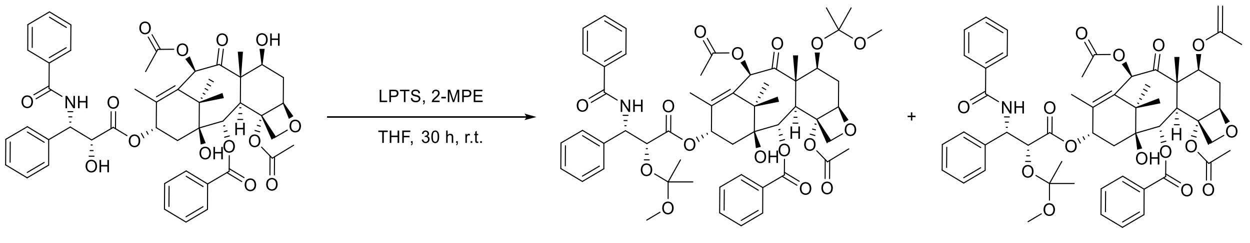

PTX | **PTX-diMOP** 48.2% conversion yield | **PTX-2'-MOP-7-IPPE** 51.8% conversion yield

To a solution of PTX (85 mg, 0.1 mmol) in anhydrous THF (1 mL) was added LPTS (5.6 mg, 0.02 mmol) and 2-MPE (154 μL) under N_2 atmosphere, and the resulting mixture was stirred for 30 h at room temperature. The reaction mixture was quenched with addition of Et_3N (1 mL) and concentrated in vacuo. The residue was chromatographed on silica gel column with EtOAc/PE (1:10 to 1:1, v/v) containing 0.5% Et_3N as an eluent to obtain a mixture of PTX-diMOP and PTX-2′-MOP-7-IPPE. The mixture was analyzed by NMR spectroscopy to contain PTX-diMOP (48.2% conversion yield) and PTX-2′-MOP-7-IPPE (51.8% conversion yield).

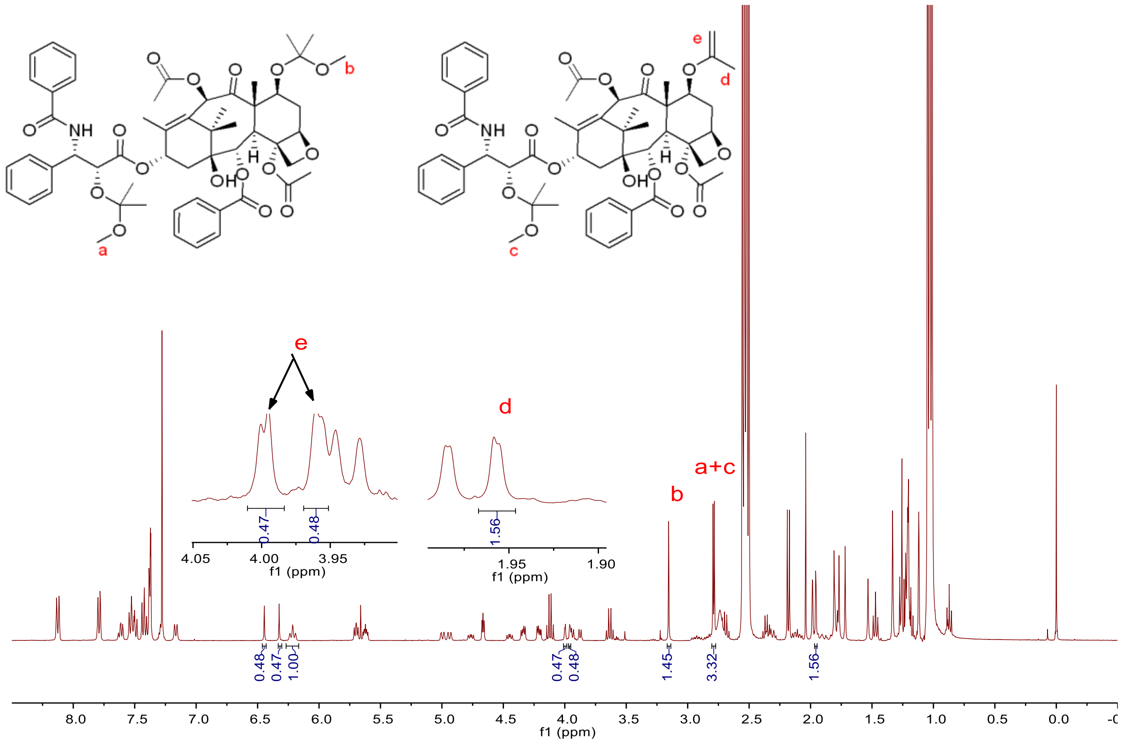

Supplementary Figure 3. ^{1}H NMR of the mixture of PTX-diMOP and PTX-2′-MOP-7-IPPE (400 MHz, $CDCl_3$).

To further ascertain the site of IPPE for PTX, a PTX-7-TES was synthesized and used as the substrate to synthesize IPPE.

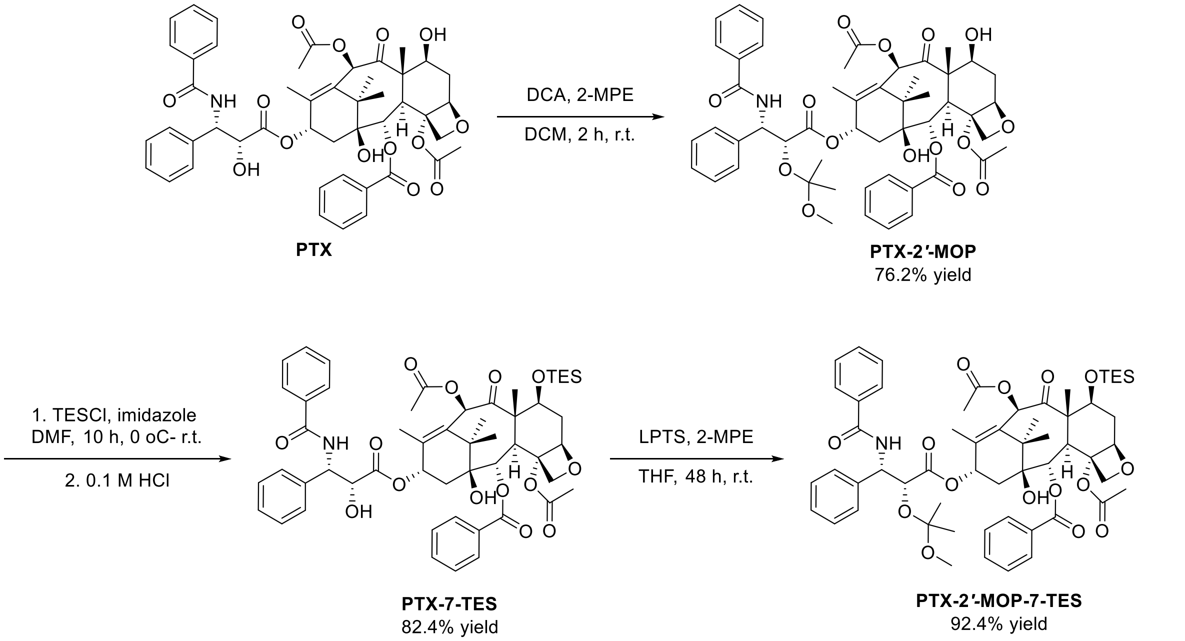

To a solution of PTX (171.0 mg, 0.2 mmol) in anhydrous DCM (2 mL) was added DCA (0.05 equiv., 100 μL of 0.1 M in DCM) and 2-MPE (360 μL) under N_2 atmosphere. The reaction was monitored by TLC at room temperature, quenched with Et_3N (200 μL) and concentrated in vacuo. The residue was chromatographed on silica gel column with EtOAc/PE (1:5 to 1:1, v/v) containing 0.5% Et_3N as an eluent to offer PTX-2′-MOP as white solid (141.0 mg, 76.2%). **^{1}H NMR** (400 MHz, $CDCl_3$) δ 8.16 – 8.06 (m, 2H), 7.83 – 7.72 (m, 2H), 7.66 – 7.58 (m, 1H), 7.57 – 7.46 (m, 3H), 7.45 – 7.34 (m, 6H), 7.28 (dt, *J* = 6.1, 2.8 Hz, 1H), 7.17 (d, *J* = 8.2 Hz, 1H), 6.29 (s, 1H), 6.22 (t, *J* = 8.9 Hz, 1H), 5.68 (d, *J* = 7.1 Hz, 1H), 5.62 (dd, *J* = 8.2, 3.3 Hz, 1H), 4.98 (dd, *J* = 9.6, 2.3 Hz, 1H), 4.66 (d, *J* = 3.3 Hz, 1H), 4.43 (dd, *J* = 11.0, 6.6 Hz, 1H), 4.26 (dd, *J* = 44.9, 8.5 Hz, 2H), 3.81 (d, *J* = 7.0 Hz, 1H), 2.78 (s, 3H), 2.52 (s, 3H), 2.34 – 2.25 (m, 1H), 2.22 (s, 3H), 2.10 (dd, *J* = 15.4, 8.9 Hz, 1H), 1.97 – 1.90 (m, 3H), 1.90 – 1.83 (m, 1H), 1.68 (s, 3H), 1.33 (s, 3H), 1.24 (s, 3H), 1.13 (d, *J* = 2.8 Hz, 6H). **^{13}C NMR** (100 MHz, $CDCl_3$) δ 203.9, 171.3, 170.0, 167.2, 167.1, 142.9, 138.5, 134.3, 133.8, 132.9, 131.8, 130.3, 129.3, 128.8, 128.7, 128.1, 127.2, 126.9, 102.4, 84.5, 81.2, 79.2, 76.5, 75.7, 75.2, 73.0, 72.2, 71.2, 58.6, 55.5, 49.7, 45.7, 43.3, 35.8, 35.7, 26.8, 24.9, 24.1, 23.0, 22.2, 20.9, 15.0, 9.7.

To a solution of PTX-2′-MOP (141.0 mg, 0.15 mmol) in anhydrous DMF (2 mL) was added imidazole (41 mg, 0.6 mmol) and the mixture was cooled to 0 °C, then was added TESCl (68 mg, 0.45 mmol) under N_2 atmosphere, and the resulting reaction mixture was stirred at room temperature. The reaction was monitored by TLC, quenched, and washed with 0.1 M HCl and extracted with EtOAc. The combined organic layer was washed with brine, dried over anhydrous $MgSO_4$ and concentrated in vacuo. The residue was chromatographed on silica gel column with EtOAc/PE (1:10 to 1:1, v/v, containing 0.5% Et_3N) as an eluent to offer PTX-7-TES as white solid (103 mg, 82.4%). **^{1}H NMR** (400 MHz, CD_3CN) δ 8.17 – 8.07 (m, 2H), 7.90 – 7.79 (m, 2H), 7.74 – 7.67 (m, 1H), 7.64 – 7.52 (m, 4H), 7.52 – 7.40 (m, 6H), 7.36 – 7.28 (m, 1H), 6.38 (s, 1H), 6.14

– 6.04 (m, 1H), 5.69 – 5.56 (m, 2H), 4.96 (dd, *J* = 9.7, 2.1 Hz, 1H), 4.74 (d, *J* = 4.5 Hz, 1H), 4.46 (dd, *J* = 10.6, 6.7 Hz, 1H), 4.19 – 4.12 (m, 2H), 3.77 (d, *J* = 7.1 Hz, 1H), 2.56 (ddd, *J* = 14.2, 9.6, 6.7 Hz, 1H), 2.39 (s, 3H), 2.33 – 2.22 (m, 2H), 2.12 (s, 3H), 1.96 (s, 3H), 1.81 (m, 1H), 1.64 (s, 3H), 1.16 (s, 3H), 1.13 (s, 3H), 0.92 (t, *J* = 7.9 Hz, 9H), 0.58 (qd, *J* = 7.9, 2.8 Hz, 6H). **^{13}C NMR** (100 MHz, CD_3CN) δ 202.4, 173.4, 171.1, 169.8, 167. 7, 166.4, 140.4, 139.5, 134.8, 134.4, 134.0, 132.2, 130.6, 130.5, 129.2, 129.1, 129.0, 128.3, 127.8, 84.2, 81.1, 78.4, 76.5, 75.5, 75.3, 74.2, 72.9, 71.7, 58.8, 56.6, 47.2, 43.7, 37.6, 36.1, 26.5, 22.8, 21.5, 20.6, 14.3, 10.3, 6.7, 5.5.

To a solution of PTX-7-TES (97.0 mg, 0.1 mmol) and LPTS (5.6 mg, 0.02 mmol) in THF (2 mL) was added 2-MPE (154 μL) under N_2 atmosphere, and the resulting reaction mixture was stirred at room temperature. The reaction was monitored by TLC, quenched with Et_3N (200 μL) and concentrated in vacuo. The residue was chromatographed on silica gel column with EtOAc/PE (1:10 to 1:3, v/v) containing 0.5% Et_3N as an eluent to offer PTX-2′-MOP-7-TES as white solid (96 mg, 92.4%). **^{1}H NMR** (400 MHz, $CDCl_3$) δ 8.01 (d, *J* = 7.6 Hz, 2H), 7.68 (d, *J* = 7.5 Hz, 2H), 7.50 (t, *J* = 7.4 Hz, 1H), 7.40 (q, *J* = 7.7 Hz, 3H), 7.31 (t, *J* = 7.6 Hz, 2H), 7.27 (s, 3H), 7.21 – 7.13 (m, 1H), 7.07 (d, *J* = 8.3 Hz, 1H), 6.34 (s, 1H), 6.10 (t, *J* = 9.2 Hz, 1H), 5.59 (d, *J* = 7.0 Hz, 1H), 5.52 (dd, *J* = 8.3, 3.1 Hz, 1H), 4.85 (d, *J* = 9.3 Hz, 1H), 4.56 (d, *J* = 3.2 Hz, 1H), 4.36 (dd, *J* = 10.6, 6.6 Hz, 1H), 4.22 (d, *J* = 8.5 Hz, 1H), 4.09 (d, *J* = 8.4 Hz, 1H), 3.72 (d, *J* = 7.0 Hz, 1H), 2.64 (s, 3H), 2.43 (s, 3H), 2.20 (dd, *J* = 15.3, 9.3 Hz, 1H), 2.06 (s, 3H), 2.03 – 1.94 (m, 2H), 1.92 (s, 3H), 1.86 – 1.74 (m, 1H), 1.59 (s, 3H), 1.22 (s, 3H), 1.11 (s, 3H), 1.07 (s, 3H), 1.02 (s, 3H), 0.82 (t, *J* = 7.9 Hz, 9H), 0.47 (q, *J* = 7.9 Hz, 6H). **^{13}C NMR** (100 MHz, $CDCl_3$) δ 201.8, 171.4, 169.9, 169.3, 167.2, 167.1, 140.6, 138.5, 134.3, 133.7, 133.6, 131.8, 130.3, 129.3, 128.8, 128.7, 128.1, 127.2, 126.9, 102.3, 84.3, 81.2, 78.8, 77.3, 76.6, 75.1, 75.0, 72.9, 72.3, 71.2, 58.5, 55.4, 49.6, 46.7, 43.4, 37.3, 35.6, 26.6, 25.0, 24.1, 23.0, 21.4, 20.9, 14.3, 10.2, 6.8, 5.3.

Thus, the 7-position but not the 2′-position of PTX was selectively transisopropenylated.

Evaluation of Fulvestrant (FUL)

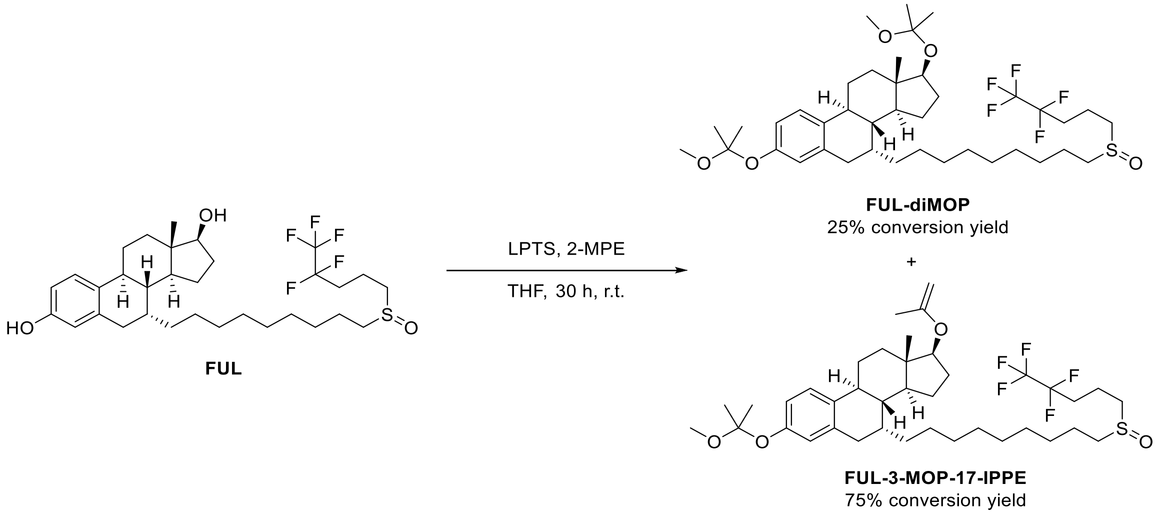

To a solution of FUL (61 mg, 0.1 mmol) and LPTS (5.6 mg, 0.02 mmol) in THF (1 mL) was added 2-MPE (154 µL) under N_2 atmosphere, and the resulting reaction mixture was stirred for 30 h at room temperature. The reaction was monitored by TLC, quenched with Et_3N (200 µL) and concentrated in vacuo. The residue was chromatographed on silica gel column with EtOAc/PE (1:10 to 1:3, v/v) containing 0.5% Et_3N as an eluent to obtain a mixture of FUL-diMOP and FUL-3-MOP-17-IPPE. The mixture was analyzed by NMR spectroscopy to contain FUL-diMOP (25% conversion yield) and FUL-3-MOP-17-IPPE (75% conversion yield).

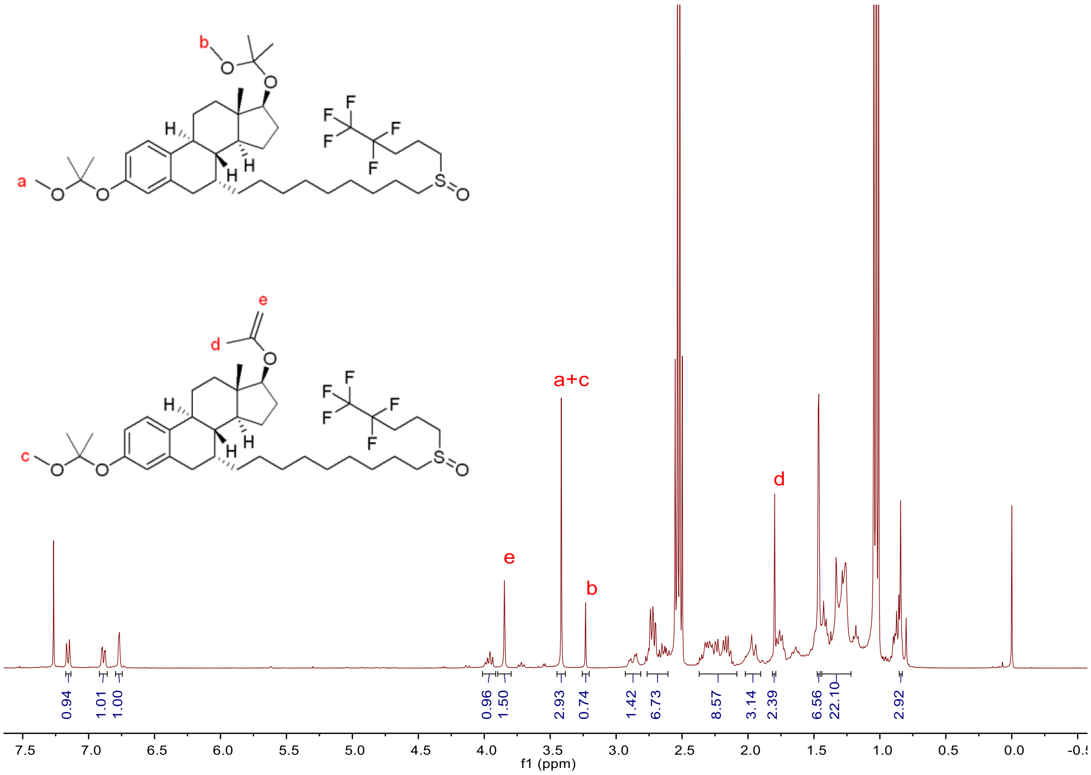

Supplementary Figure 4. ^{1}H NMR spectrum of the mixture of FUL-diMOP and FUL-3-MOP-17-IPPE (400 MHz, $CDCl_3$).

Thus, the alcohol of FUL gave the IPPE, and the phenol of FUL gave only the MOP ketal.

Evaluation of Floxuridine (FUDR)

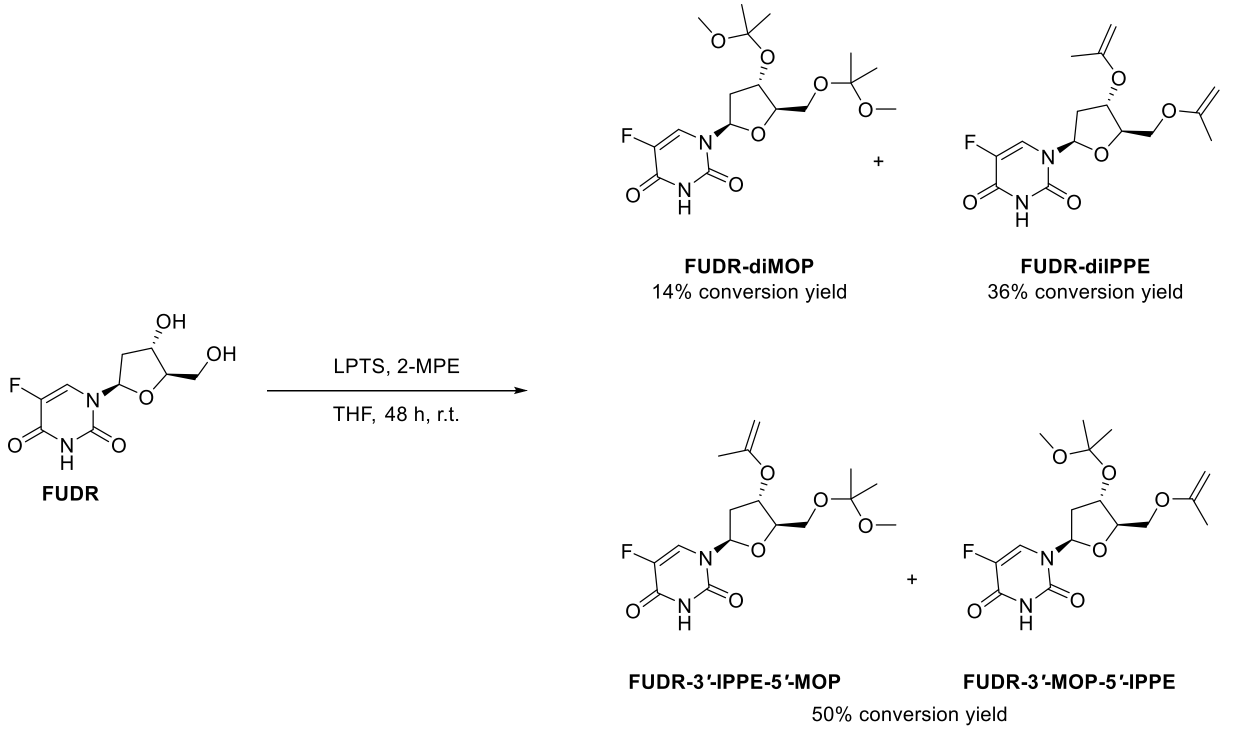

To a solution of FUDR (25 mg, 0.1 mmol) and LPTS (5.6 mg, 0.02 mmol) in THF (1 mL) was added 2-MPE (154 μL) under N_2 atmosphere, and the resulting reaction mixture was stirred for 48 h at room temperature. The reaction was monitored by TLC, quenched with Et_3N (1 mL) and concentrated in vacuo. The residue was chromatographed on silica gel column with EtOAc/PE (1:10 to 1:1, v/v) containing 0.5% Et_3N as an eluent to obtain a mixture of FUDR-diMOP, FUDR-3′-IPPE-5′-MOP, FUDR-3′-MOP-5′-IPPE and FUDR-diIPPE. The mixture was analyzed by NMR spectroscopy to contain FUDR-diMOP (14% conversion yield), FUDR-3′-IPPE-5′-MOP plus FUDR-3′-MOP-5′-IPPE (50% conversion yield) and FUDR-diIPPE (36% conversion yield).

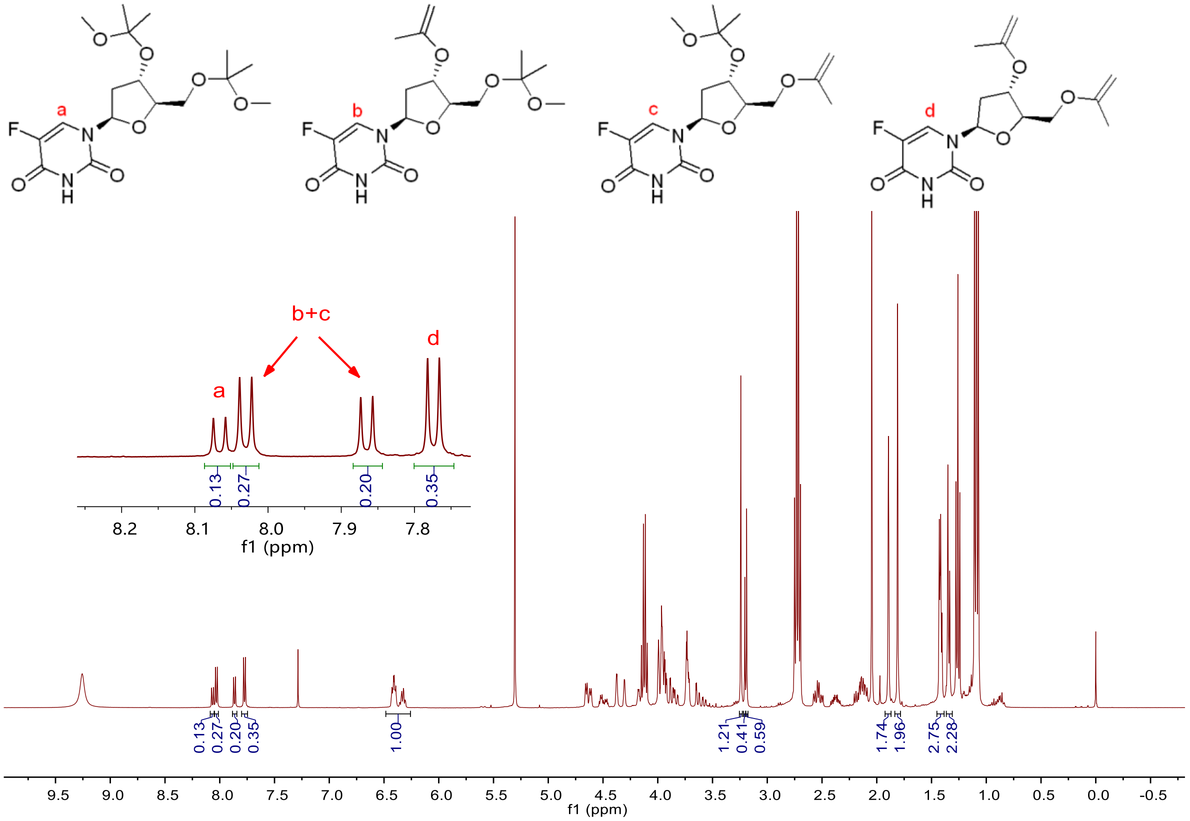

Supplementary Figure 5. ^{1}H NMR spectrum of the mixture of FUDR-diMOP, FUDR-3′-IPPE-5′-MOP, FUDR-3′-MOP-5′-IPPE and FUDR-diIPPE (400 MHz, $CDCl_3$).

Thus, two hydroxyl groups of FUDR were both converted to IPPEs using organocatalytic transisopropenylation.

Evaluation of Tafluprost (TAF)

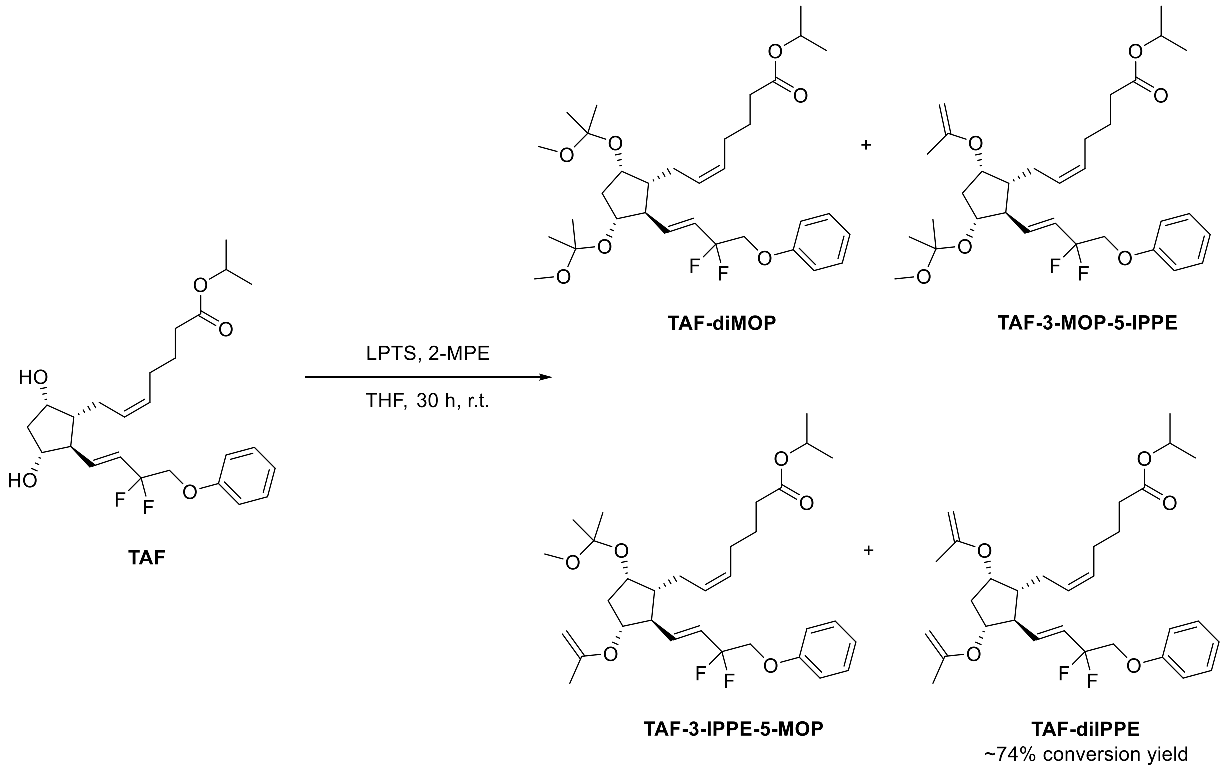

To a solution of TAF (45 mg, 0.1 mmol) and LPTS (0.56 mg, 2 μmol, 100 μL of 5.6 mg/mL in DCM) in THF (1 mL) was added 2-MPE (154 μL) under N_2 atmosphere, and the resulting reaction mixture was stirred for 30 h at room temperature. The reaction was monitored by TLC, quenched with Et_3N (200 μL) and concentrated in vacuo. The residue was chromatographed on silica gel column with EtOAc/PE (1:100 to 1:20, v/v) containing 0.5% Et_3N as an eluent to obtain a mixture of TAF-diMOP, TAF-3-MOP-5-IPPE, TAF-3-IPPE-5-MOP and TAF-diIPPE. The mixture was analyzed by NMR spectroscopy, and the majority of the mixture was TAF-diIPPE (~74% conversion yield).

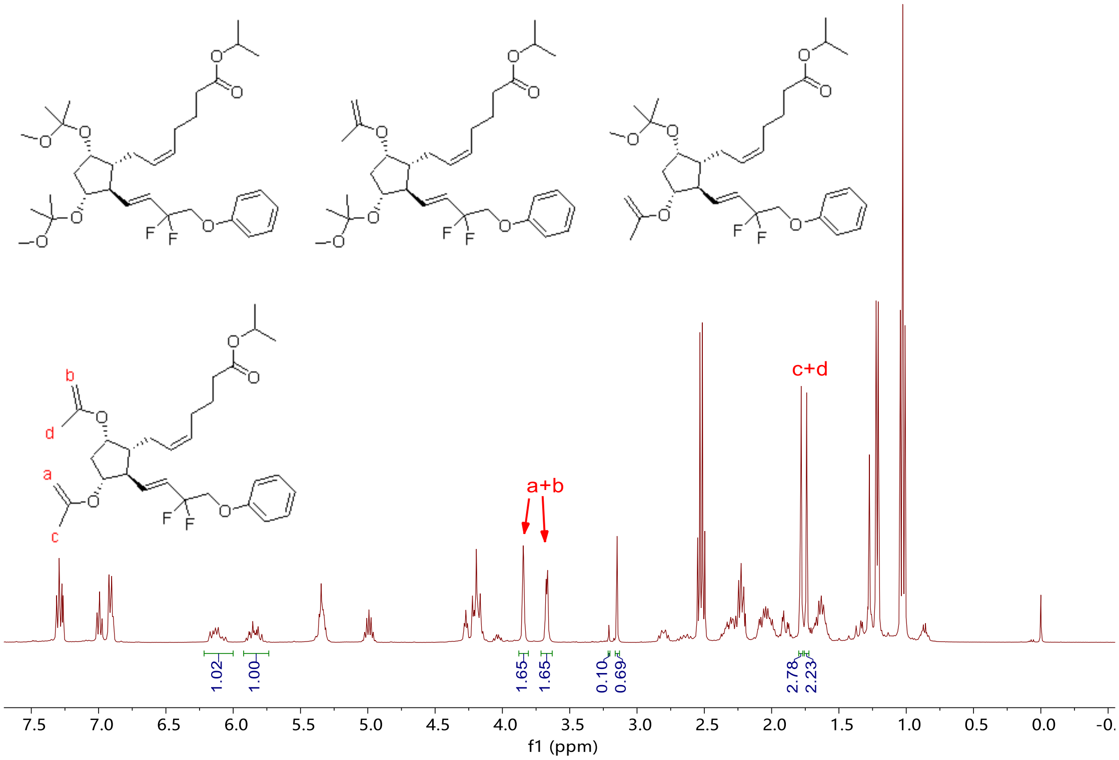

Supplementary Figure 6. ^{1}H NMR spectrum of the mixture of TAF-diMOP, TAF-3-MOP-5-IPPE, TAF-3-IPPE-5-MOP and TAF-diIPPE (400 MHz, $CDCl_3$).

Thus, two hydroxyl groups of TAF were both converted to IPPEs using organocatalytic transisopropenylation.

Evaluation of 7-Ethyl-10-hydroxycamptothecin (SN38)

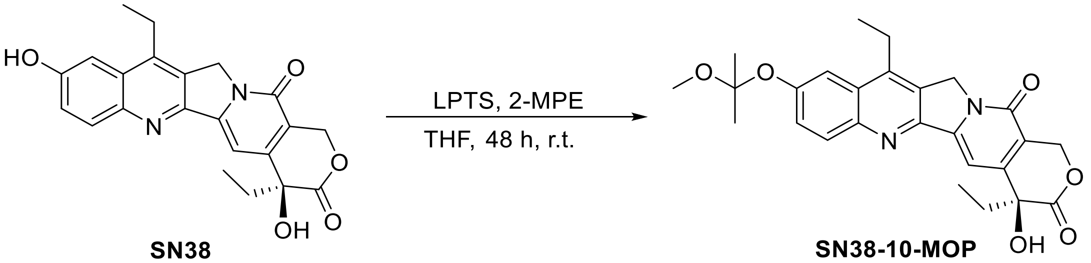

To a solution of SN38 (39 mg, 0.1 mmol) and LPTS (5.6 mg, 0.02 mmol) in THF (1 mL) was added 2-MPE (154 μL) under N_2 atmosphere, and the resulting reaction mixture was stirred for 48 h at room temperature. The reaction was monitored by TLC, quenched with Et_3N (200 μL) and concentrated in vacuo. The residue was chromatographed on silica gel column with EtOAc/PE (1:5 to 2:1, v/v) containing 0.5% Et_3N as an eluent to offer SN38-10-MOP as white solid (13 mg, 30%). **^{1}H NMR** (400MHz, $CDCl_3$) δ 8.13 (d, *J* = 9.2 Hz, 1H), 7.83 (d, *J* = 2.6 Hz, 1H), 7.67 – 7.49 (m, 2H), 5.76 (d, *J* = 16.2 Hz, 1H), 5.31 (d, *J* = 16.2 Hz, 1H), 3.49 (s, 3H), 3.14 (q, *J* = 7.7 Hz,

2H), 1.96 – 1.82 (m, 3H), 1.61 (s, 6H), 1.34 – 1.24 (m, 4H), 0.92 – 0.75 (m, 3H). **^{13}C NMR** (100MHz, $CDCl_3$) δ 157.8, 154.7, 150.3, 147.5, 146.0, 144.2, 131.6, 128.0, 126.9, 125.9, 117.9, 111.1, 104.7, 97.5, 72.8, 66.4, 49.5, 31.6, 29.7, 25.2, 23.2, 14.1, 13.9, 7.8. **HRMS** (ESI): m/z calcd for $C_{26}H_{28}N_2O_6$ $[M+Na]^+$: 487.1840; found 487.1838.

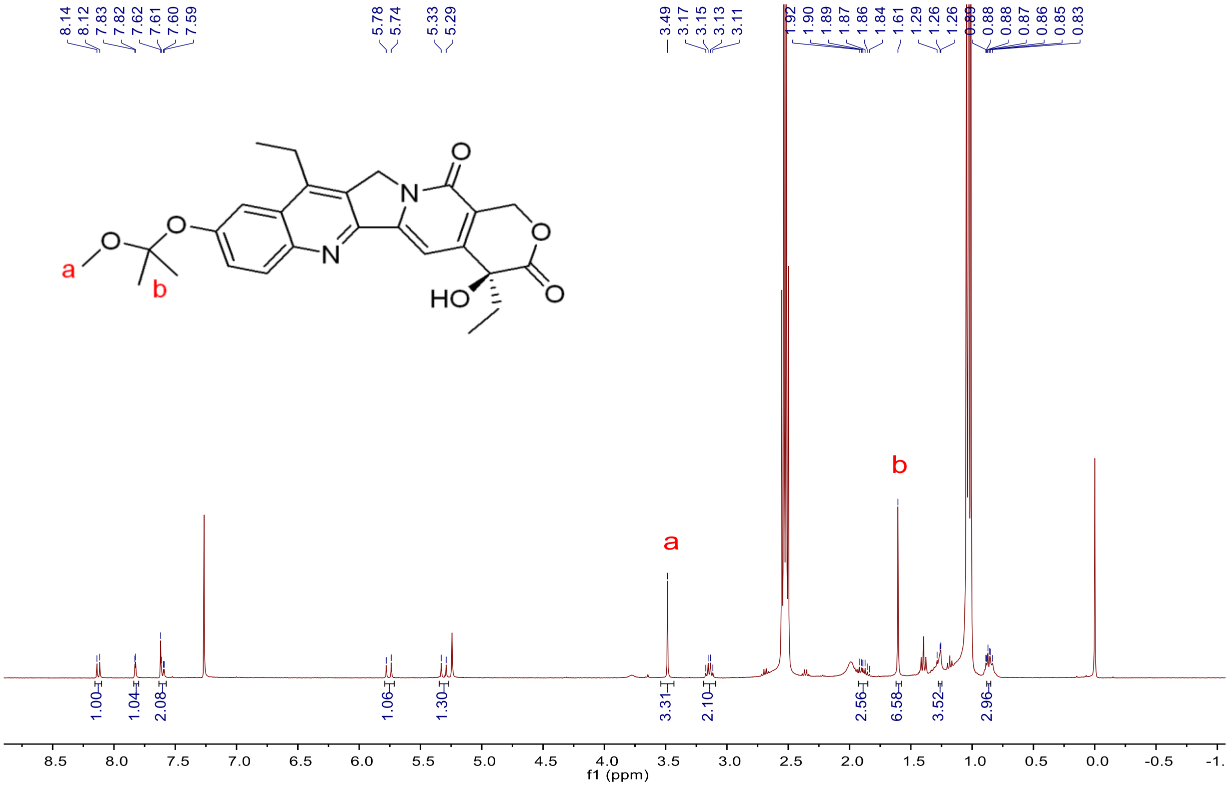

Supplementary Figure 7. ^{1}H NMR spectrum of compound SN38-10-MOP (400 MHz, $CDCl_3$).

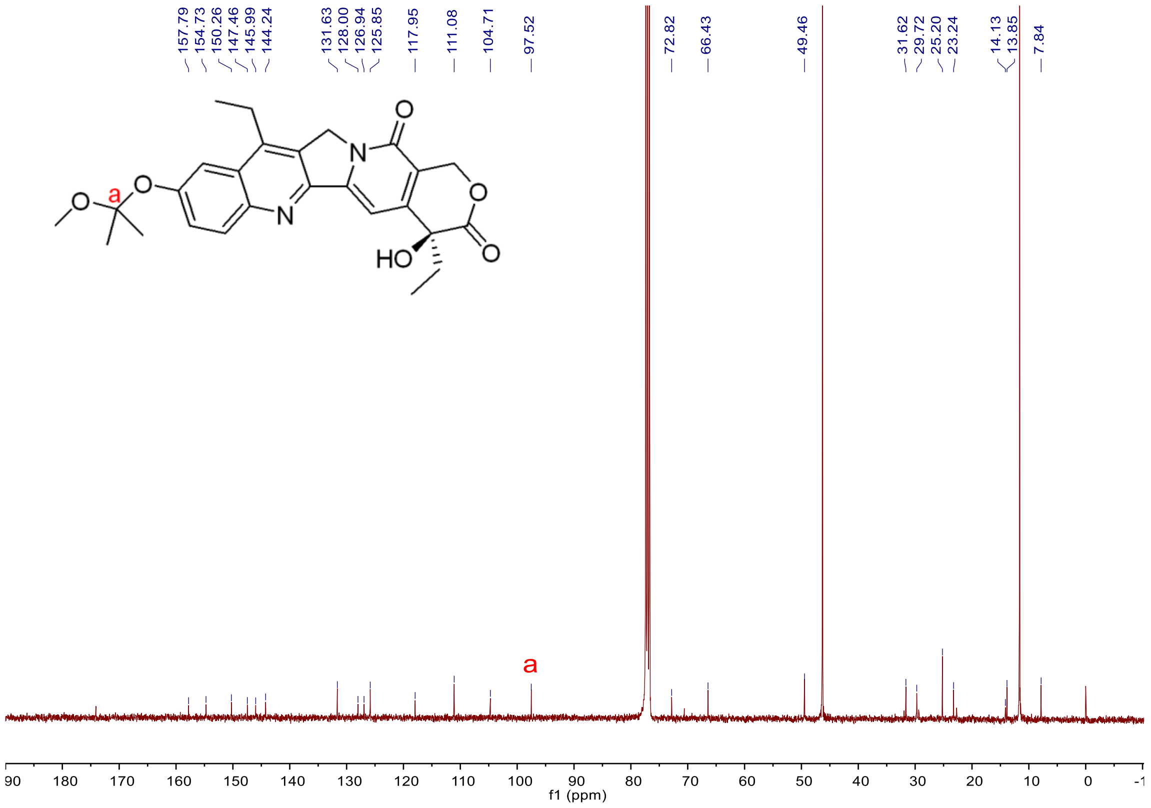

Supplementary Figure 8. ^{13}C NMR spectrum of compound SN38-10-MOP (100 MHz, $CDCl_3$).

Thus, the hindered tertiary alcohol on SN38 did not react at all due to steric hindrance, and the phenol on SN38 gave only the corresponding MOP ketal.

Evaluation of 7-Hydroxycoumarin (HDC)

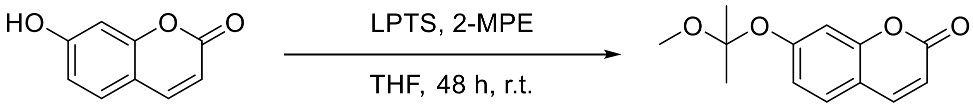

HDC **HDC-7-MOP**

28% yield

To a solution of HDC (65 mg, 0.4 mmol) and LPTS (22.3 mg, 0.08 mmol) in THF (2 mL) was added 2-MPE (615 μL) under N_2 atmosphere, and the resulting reaction mixture was stirred for 48 h at room temperature. The reaction was monitored by TLC, quenched with Et_3N (400 μL) and concentrated in vacuo. The residue was chromatographed on silica gel column with EtOAc/PE (1:20 to 2:1, v/v) containing 0.5% Et_3N as an eluent to offer HDC-7-MOP as pale-yellow solid (10.3 mg, 28%). **^{1}H NMR** (400MHz, $CDCl_3$) δ 7.64 (d, *J* = 9.5 Hz, 1H), 7.35 (d, *J* = 8.5 Hz, 1H), 7.21 (d, *J* = 2.3 Hz, 1H), 7.02 (dd, *J* = 8.5, 2.3 Hz, 1H), 6.28 (d, *J* = 9.5 Hz, 1H), 3.39 (s, 3H), 1.55 (s, 6H). **^{13}C NMR** (100MHz, $CDCl_3$) δ 154.8, 143.4, 128.1, 117.2, 113.8, 107.8, 105.0, 49.5, 31.5, 29.7, 25.0. **HRMS** (ESI): m/z calcd for $C_{13}H_{14}O_4$ $[M+Na]^+$: 257.0784; found 257.0785.

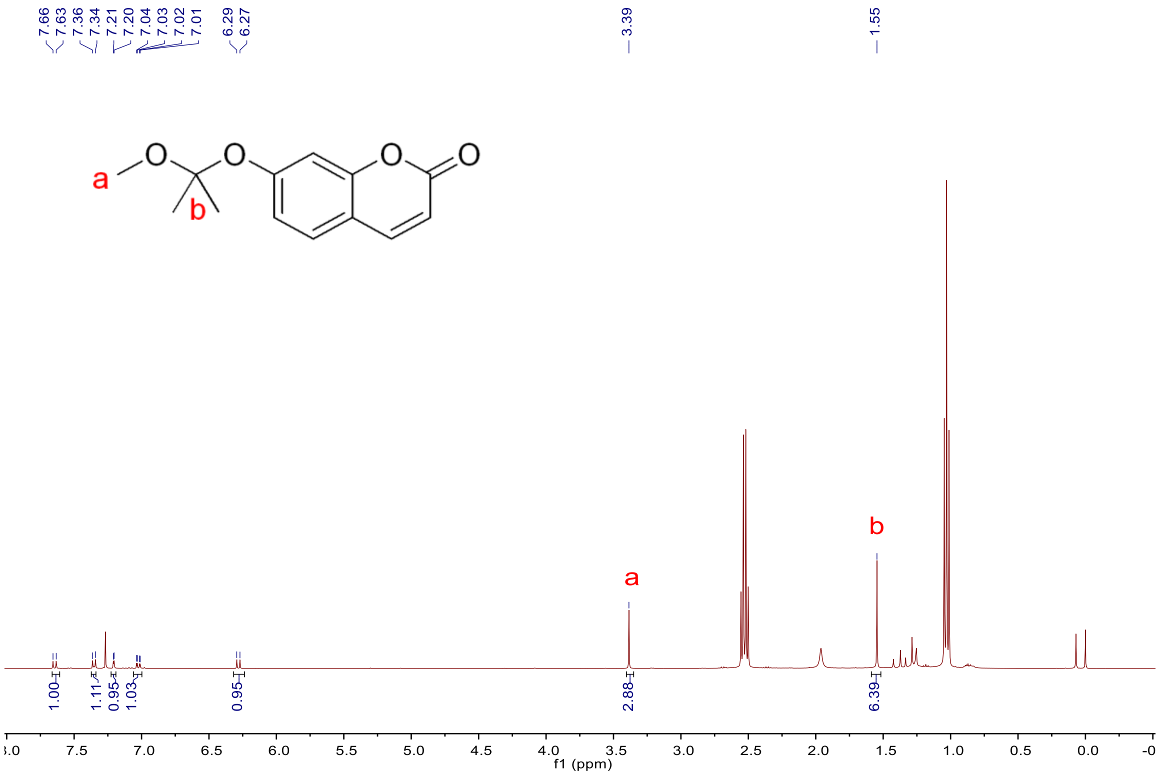

Supplementary Figure 9. ^{1}H NMR spectrum of compound HDC-7-MOP (400 MHz, $CDCl_3$).

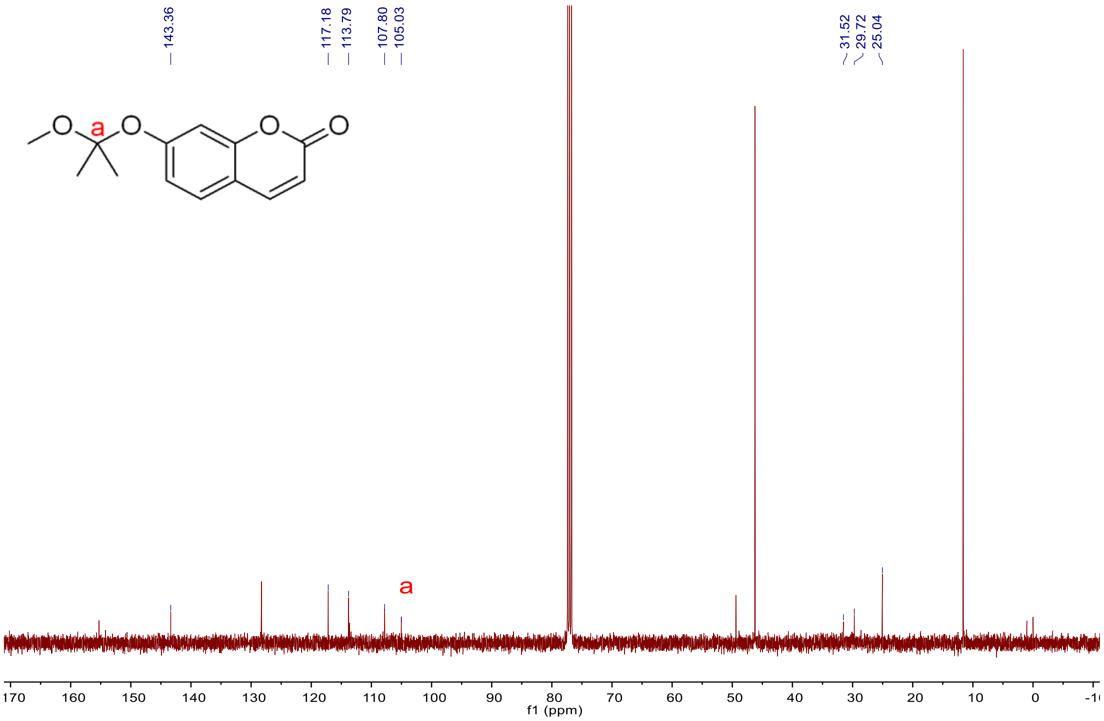

Supplementary Figure 10. ^{13}C NMR spectrum of compound HDC-7-MOP (100 MHz, $CDCl_3$).

Thus, the phenol on HDC gave only the corresponding MOP ketal.

Evaluation of Estradiol (EST)

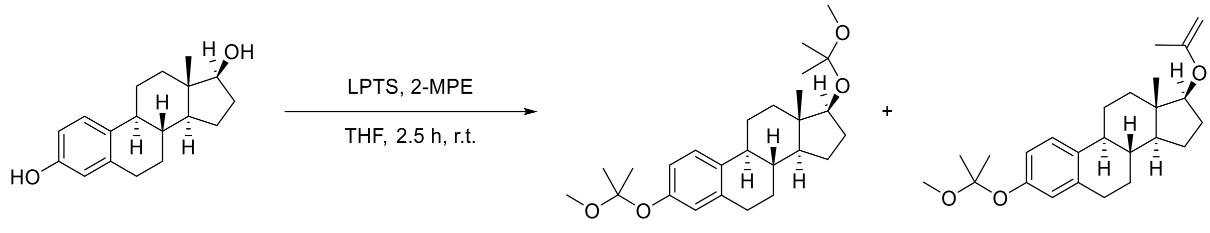

To a solution of EST (272 mg, 1 mmol) and LPTS (1.4 mg, 5 µmol) in THF (2 mL) was added 2-MPE (1.54 mL) under N_2 atmosphere, and the resulting reaction mixture was stirred for 2.5 h at room temperature. The reaction was monitored by TLC, quenched with Et_3N (200 µL) and concentrated in vacuo. The residue was chromatographed on silica gel column with EtOAc/PE (0:1 to 1:4, v/v) containing 0.5% Et_3N as an eluent to obtain a mixture of EST-diMOP and EST-3-MOP-17-IPPE. The mixture was analyzed by NMR spectroscopy to contain EST-diMOP (46% conversion yield) and EST-3-MOP-17-IPPE (54% conversion yield).

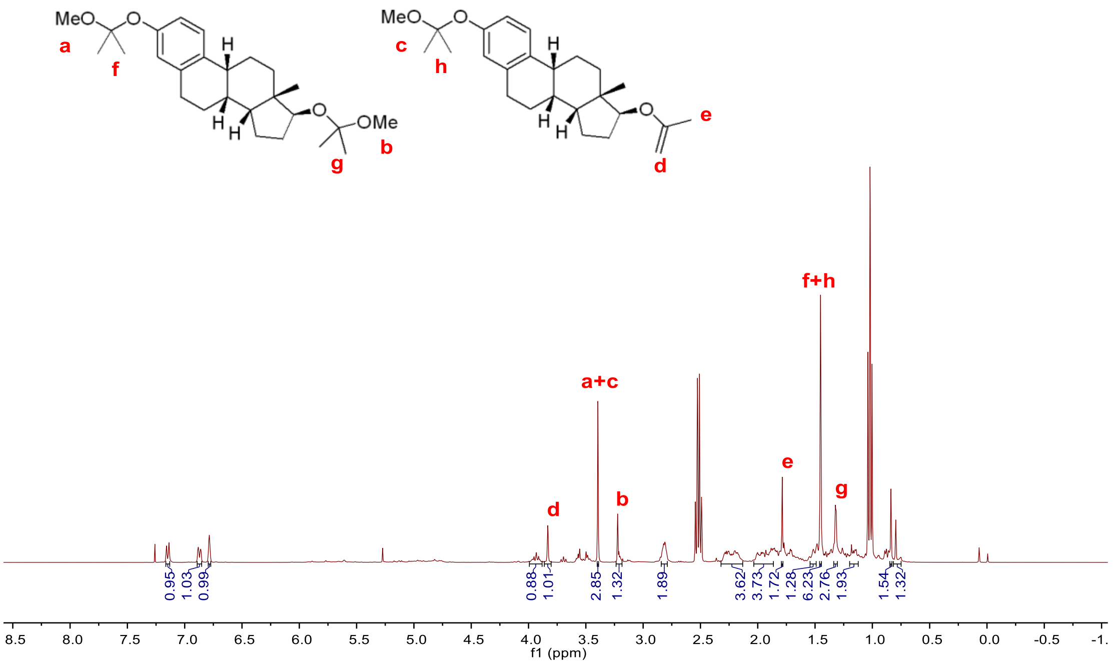

Supplementary Figure 11. ^1H NMR spectrum of the mixture of EST-diMOP and EST-3-MOP-17-IPPE (400 MHz, $CDCl_3$).

Similar as FUL, the alcohol of EST gave the IPPE, and the phenol of EST gave only the MOP ketal.

6) Synthesis of Drug-Derived Isopropenyl Ethers Using Other Methods

Reported Methods for Synthesis of Isopropenyl Ethers

Reported methods for the synthesis of IPPEs were summarized below. To our knowledge, no drug-derived IPPEs had ever been reported. Albeit that each of these methods has its pros and cons and that no attempt to synthesize IPPEs from complex alcohols has been reported, it appears none of these methods could be applied to the synthesis of IPPEs from complex drugs (e.g., taxanes). Please see our analysis below and our experiments.

Synthetic strategy	Catalyst/reagent
Alkoxylation-elimination of ketals<br>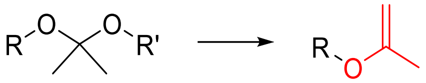	Acid-catalyzed pyrolysis[2-5]<br>Lewis acids (e.g., TMSOTf, TMSI, and TMSCl) and organic weak bases (e.g., pyridine, TEA, and DIPEA)[6-10]<br>Triisobutylaluminum[11]
Thermal decarboxylation<br>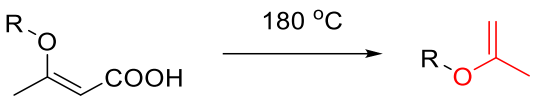	*β*-Alkoxycrotonic acids[12]
Methylenation<br>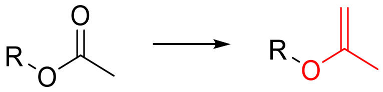	Tebbe or Petasis reagent[13, 14 15]
Wittig reaction<br>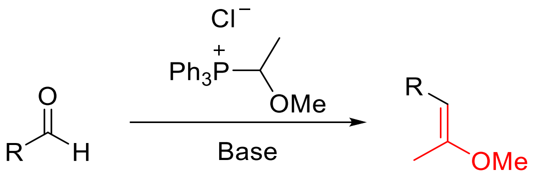	Wittig reagent[16]
Addition of alcohols to alkynes<br>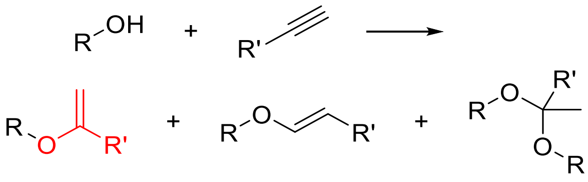	Transition metal catalysts (e.g., gold)[17-19]
Transisopropenylation<br>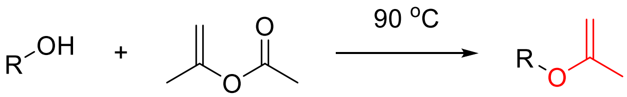	$[Ir(cod)Cl]_2/Na_2CO_3$[20-22]

Supplementary Figure 12. Methods for synthesis of IPPEs.

2,2-Dimethoxyalkanes have been used to prepare IPPEs by acid-catalyzed alkoxylation-elimination at high temperatures. Using pyrolysis for the synthesis of IPPEs, ketals that are symmetrical and derived from alcohols with low boiling points and acetone are required. Although the alkoxylation-elimination process could also be achieved at low temperatures by treating mixed ketals with strong Lewis acid and a weak organic base, this protocol is limited to sterically hindered secondary alcohols. Furthermore, these alkoxylation-elimination approaches require two-step procedures and suffer from poor functional group compatibility (high temperatures and use of strong Lewis acid), and this is also true for thermal decarboxylation of *β*-alkoxycrotonic acids.

Methylenation of acetates using Tebbe and Petasis reagents requires expensive reagents, and this strategy lacks selectivity and does not apply to substrates with multiple carbonyl groups (such as taxanes and nucleotides). For the Wittig reaction, the reaction condition is too alkaline to be compatible with base-sensitive groups (e.g., ester) and substrates, and the workup is quite tedious. Moreover, optimal substrates for Wittig reaction are less sterically hindered ketones and aldehydes.

Synthetic approaches from alcohols, such as the addition of alcohols to alkynes and transisopropenylation, are relatively more advantageous than the methods mentioned above due to the one-step procedure. However, these approaches require expensive transition metal catalysts (e.g., gold and iridium). Note that transition-metal-catalyzed addition reactions are more suitable for phenol substrates but not for alcohols. These reactions suffer from the lack of site selectivity, and therefore a mixture of isomers and byproducts is often produced. For example, most of the product is ketal, when gold catalyzes the addition of alcohols to alkynes. For the transisopropenylation approach using $[Ir(cod)Cl]_2/Na_2CO_3$ catalytic system, the substrate scope of the method appears to be also limited by harsh reaction conditions (high temperature).

Using paclitaxel and floxuridine as model hydroxy-group-containing drugs, we attempted to synthesize drug-derived IPPEs using some of the above approaches. For instance, we attempted to synthesize IPPE derivatives from drugs using transisopropenylation ($[Ir(cod)Cl]_2/Na_2CO_3$ catalytic system), and from ketalated drug derivatives using alkoxylation-elimination strategy with Lewis acid-promoted reactions. Our results indicated that these reactions could not afford paclitaxel- and floxuridine-derived IPPEs, due to harsh reaction conditions.

Synthesis of PTX-Derived IPPE Using $[Ir(cod)Cl]_2/Na_2CO_3$ Catalytic Method

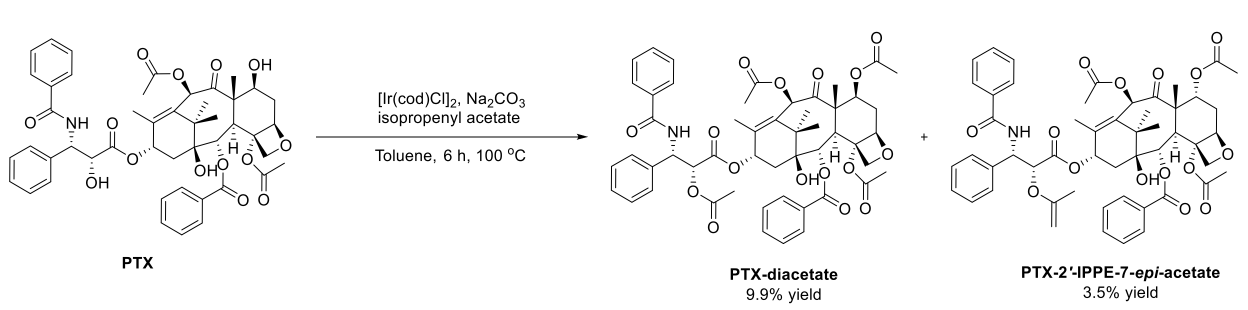

Reaction between PTX with isopropenyl acetate was catalyzed by $[Ir(cod)Cl]_2/Na_2CO_3$ and performed according to the reported procedure.[22] To a solution of PTX (341.6 mg, 0.4 mmol), anhydrous Na_2CO_3 (84.8 mg, 0.8 mmol) and $[Ir(cod)Cl]_2$ (9.7 mg, 14.4 μmol) in anhydrous toluene (5 mL) was added isopropenyl acetate (222 μL), and the mixture was heated at 100 ^{o}C. Six hours later, the mixture was cooled and diluted with hexane (0.1% Et_3N), filtered, and concentrated in vacuo. The residue was chromatographed on silica gel column with EtOAc/PE (1:5 to 2:1, v/v) as an eluent. Et_3N (0.5%) was added to prevent the hydrolysis of IPPE group. NMR analyses indicated that no desired product was obtained. By contrast, trans-acetate reaction produced PTX-diacetate (37 mg, 9.9%) as major product. A minor transisopropenylation product PTX-2′-IPPE-7-*epi*-acetate (13 mg, 3.5%) at 2′-OH of PTX was also obtained, but unfortunately, concurrent epimerization at 7-position of PTX occurred.

PTX-diacetate: **^{1}H NMR** (400 MHz, $CDCl_3$) δ 8.16 – 8.11 (m, 2H), 7.77 – 7.74 (m, 2H), 7.64 – 7.57 (m, 1H), 7.53 (d, *J* = 8.6 Hz, 4H), 7.42 – 7.37 (m, 6H), 6.95 (d, *J* = 8.2 Hz, 1H), 6.30 (s, 1H), 6.26 (d, *J* = 9.2 Hz, 1H), 5.95 (dd, *J* = 9.1, 3.3 Hz, 1H), 5.69 (d, *J* = 7.1 Hz, 1H), 5.52 (d, *J* = 3.4 Hz, 1H), 4.98 (d, *J* = 9.4 Hz, 1H), 4.45 (dd, *J* = 11.0, 6.6 Hz, 1H), 4.31 – 4.28 (m, 2H), 4.20 (d, *J* = 8.4 Hz, 1H), 3.81 (d, *J* = 7.0 Hz, 1H), 2.45 (s, 3H), 2.23 (s, 3H), 2.20 – 2.18 (m, 2H), 2.16 (s, 3H), 2.05 (s, 3H), 1.78 – 1.71 (m, 2H), 1.68 (s, 3H), 1.26 (s, 3H), 1.24 (s, 3H), 1.14 (s, 3H). **^{13}C NMR** (100 MHz, $CDCl_3$) δ 203.8, 171.2, 170.0, 169.9, 168.2, 167.7, 167.1, 167.0, 142.7, 137.0, 133.7, 132.8, 132.3, 132.0, 130.9, 130.2, 129.2, 129.1, 128.9, 128.8, 128.7, 128.5, 127.1, 126.9, 126.6, 84.5, 81.1, 79.1, 75.6, 75.1, 74.0, 72.1, 71.8, 65.6, 58.5, 52.9, 45.6, 43.2, 35.6, 30.6, 29.7, 26.8, 22.7, 20.8, 20.5, 19.2, 14.8, 9.6.

PTX-2′-IPPE-7-*epi*-acetate: **^{1}H NMR** (400 MHz, $CDCl_3$) δ 8.21 – 8.11 (m, 2H), 7.78 – 7.72 (m, 2H), 7.64 – 7.58 (m, 1H), 7.55 – 7.47 (m, 4H), 7.43 – 7.35 (m, 6H), 7.07 (d, *J* = 9.2 Hz, 1H), 6.80 (s, 1H), 6.19 (t, *J* = 8.9 Hz, 1H), 5.90 (dd, *J* = 9.2, 3.0 Hz, 1H), 5.76 (d, *J* = 7.5 Hz, 1H), 5.62 (s, 1H), 4.94 – 4.90 (m, 1H), 4.66 (d, *J* = 11.6 Hz, 1H), 4.39 – 4.29 (m, 2H), 4.09 (m, 1H), 3.96 – 3.90 (m, 2H), 3.74 – 3.66 (m, 1H), 2.45 (s, 3H), 2.18 (s, 3H), 2.14 – 2.01 (m, 2H), 1.91 (d, *J* = 4.3 Hz, 1H), 1.89 (s, 3H), 1.84 (s, 3H), 1.73 – 1.71 (m, 1H), 1.67 (s, 3H), 1.26 (s, 6H), 1.18 (s, 3H). **^{13}C NMR** (100 MHz, $CDCl_3$) δ 207.0, 171.7, 169.4, 168.8, 167.2, 166.7, 158.0, 140.0, 137.6, 133.9, 133.6, 133.2, 131.9, 130.9, 130.2, 128.9, 128.8, 128.7, 128.3, 127.2, 126.8, 84.5, 82.7, 82.2, 79.1, 78.1, 75.8, 75.3, 71.6, 65.6, 57.6, 53.3, 53.0, 42.6, 40.3, 36.1, 35.4, 30.6, 29.7, 26.0, 22.7, 20.9, 20.6, 16.2, 8.1.

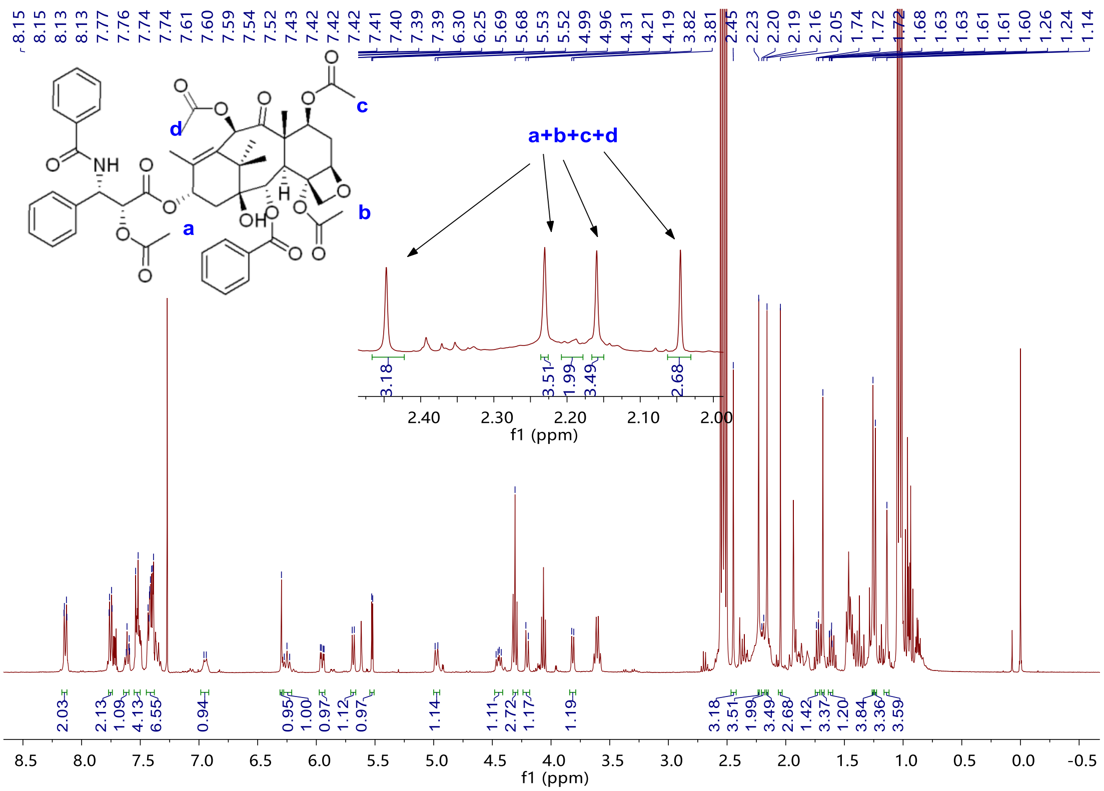

Supplementary Figure 13. ^{1}H NMR spectrum of PTX-diacetate (400 MHz, $CDCl_3$).

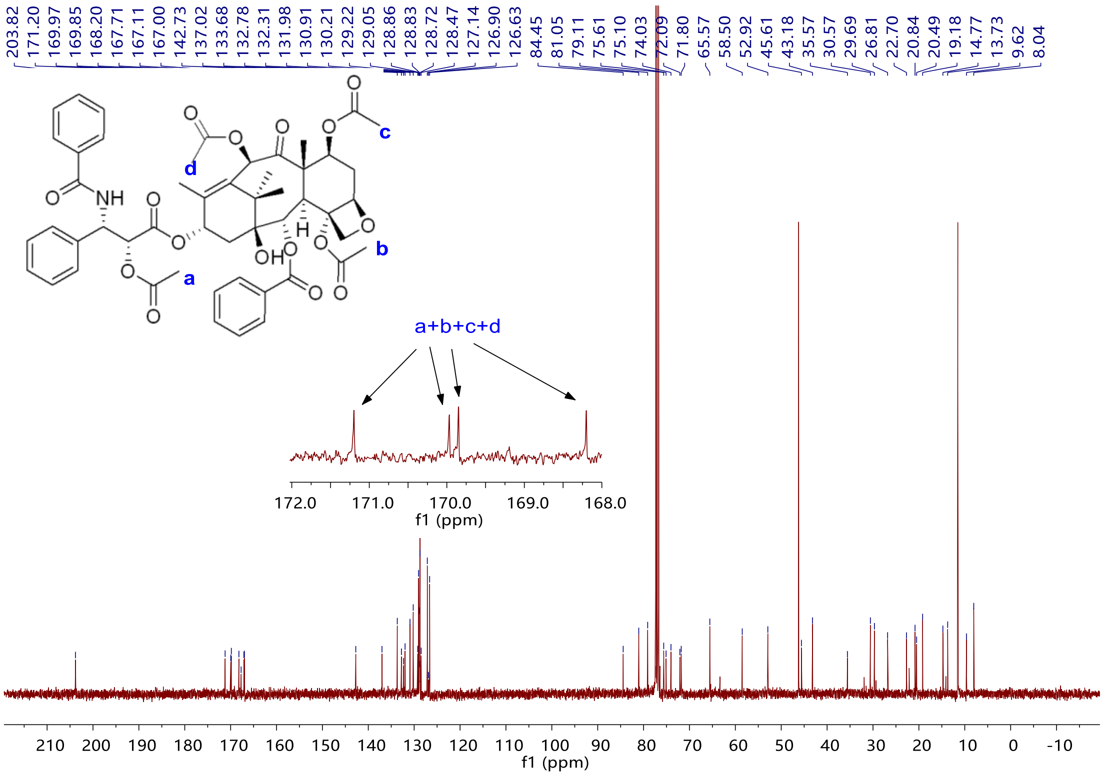

Supplementary Figure 14. ^{13}C NMR spectrum of PTX-diacetate (100 MHz, $CDCl_3$).

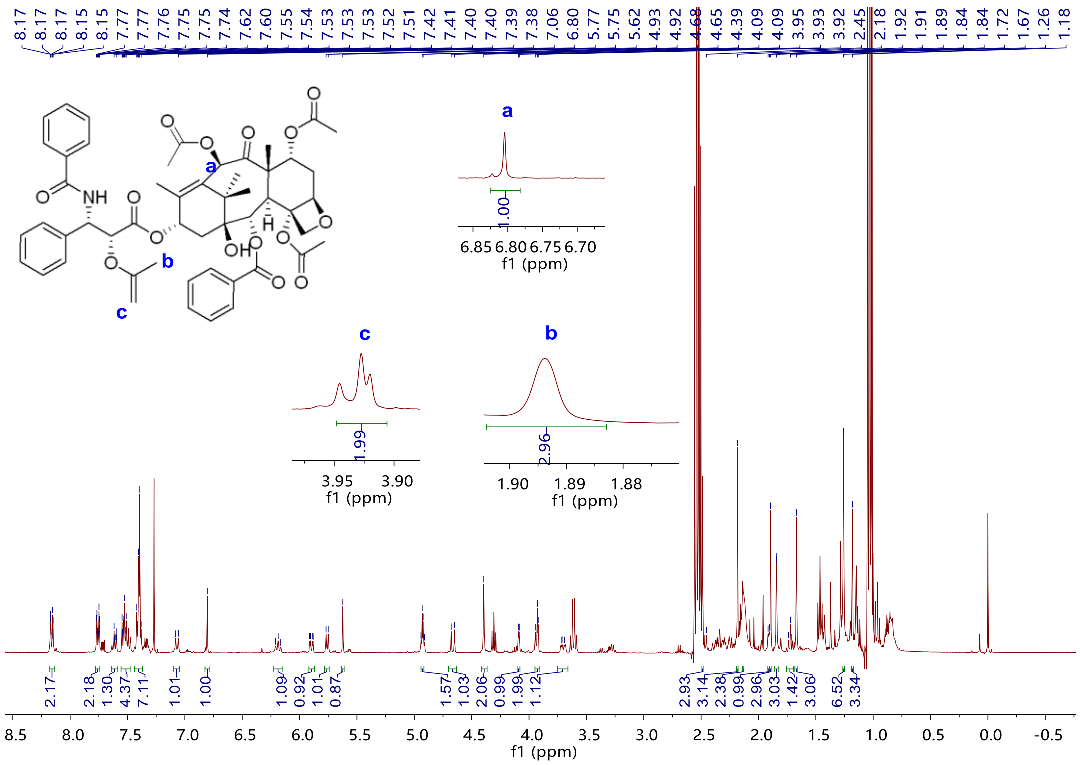

Supplementary Figure 15. ^{1}H NMR spectrum of PTX-2′-IPPE-7-*epi*-acetate (400 MHz, $CDCl_3$). Epimerization was indicated by the peak at 6.80 ppm.

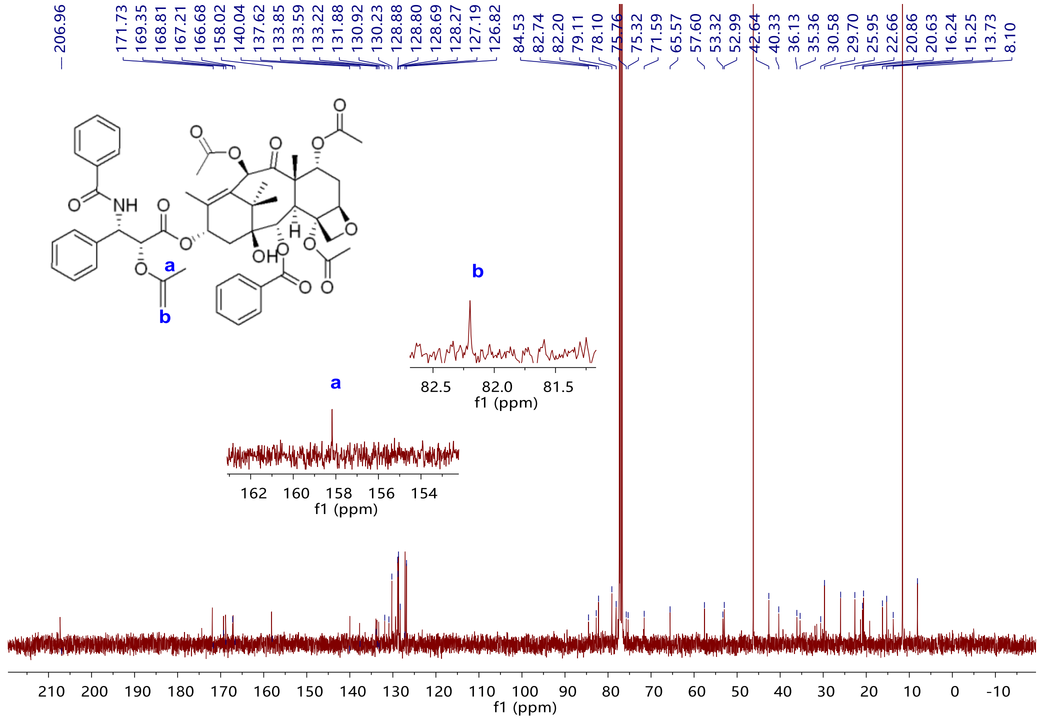

Supplementary Figure 16. ^{13}C NMR spectrum of PTX-2′-IPPE-7-*epi*-acetate (100 MHz, $CDCl_3$).

Synthesis of PTX-Derived IPPE Using TMSOTf/DIPEA Method

Elimination of methanol from PTX-diMOP using TMSOTf/DIPEA via a two-step process was attempted to synthesize PTX-diIPPE, according to reported procedures.[7]

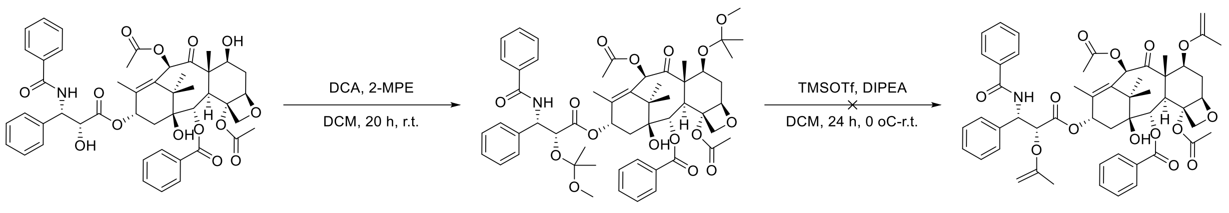

PTX PTX-diMOP 81.1% yield PTX-diPPE

To a solution of PTX (50.4 mg, 59.0 μmol) in anhydrous DCM (2 mL) was added DCA (0.01 equiv., 5.9 μL of 0.1 M in DCM) and 2-MPE (113.0 μL) under N_2 atmosphere, and the resulting reaction mixture was stirred for 20 h at room temperature. The reaction was quenched with Et_3N (1 mL) and concentrated in vacuo. The residue was chromatographed on silica gel column with EtOAc/PE (1:10 to 1:1, v/v, containing 0.5% Et_3N) as an eluent to yield PTX-diMOP as white solid (47.7 mg, 81.1%). **^{1}H NMR** (400 MHz, $CDCl_3$) δ 8.14 – 8.07 (m, 2H), 7.77 (dd, *J* = 7.1, 1.8 Hz, 2H), 7.59 (d, *J* = 7.5 Hz, 1H), 7.56 – 7.33 (m, 9H), 7.16 (d, *J* = 8.2 Hz, 1H), 6.43 (s, 1H), 6.20 (t, *J* = 9.0 Hz, 1H), 5.68 (d, *J* = 7.0 Hz, 1H), 5.63 (s, 1H), 5.62 – 5.57 (m, 1H), 4.92 (d, *J* = 9.7 Hz, 1H), 4.64 (d, *J* = 3.2 Hz, 1H), 4.43 (dd, *J* = 10.7, 6.4 Hz, 1H), 4.32 (d, *J* = 8.5 Hz, 1H), 4.19 (d, *J* = 8.5 Hz, 1H), 3.86 (d, *J* = 7.0 Hz, 1H), 3.14 (s, 3H), 2.78 (s, 3H), 2.17 (s, 3H), 1.97 (d, *J* = 1.4 Hz, 3H), 1.75 (s, 3H), 1.52 (s, 3H), 1.32 (s, 3H), 1.24 (s, 6H), 1.21 (s, 3H), 1.18 (s, 3H), 1.10 (s, 3H). **^{13}C NMR** (100 MHz, $CDCl_3$) δ 201.7, 171.3, 169.7, 169.2, 167.2, 166.9, 140.1, 138.4, 134.2, 133.6, 133.5, 131.6, 130.1, 129.3, 128.6, 128.5, 127.9, 127.0, 126.8, 102.2, 101.2, 84.4, 81.2, 78.6, 76.4, 74.9, 74.8, 72.8, 72.2, 71.2, 63.4, 57.4, 55.3, 53.0, 49.5, 48.6, 47.0, 43.3, 35.5, 34.3, 29.6, 26.5, 24.8, 24.2, 23.9, 23.5, 22.9, 22.6, 22.5, 21.3, 20.8, 14.3, 14.0, 10.9.

To a solution of PTX-diMOP (20 mg, 0.02 mmol) in anhydrous DCM (1 mL) was added anhydrous DIPEA (11.0 μL, 0.06 mmol, 3.2 equiv.) under N_2 atmosphere, and the resulting mixture was cooled to 0 ^{o}C. Then, TMSOTf (9.1 μL, 0.05 mmol, 2.6 equiv.) was added dropwise, and the reaction mixture was warmed to room temperature. After 24 h, the reaction mixture was quenched with 1M NaOH and extracted with EtOAc. The combined organic layer was washed with brine, dried over anhydrous $MgSO_4$ and concentrated in vacuo. The residue was chromatographed on silica gel column with EtOAc/PE (1:10 to 1:1, v/v, containing 0.5% Et_3N) as an eluent. TLC and NMR analyses indicated that PTX was significantly decomposed and no desired compound was yielded.

Synthesis of FUDR-Derived IPPE Using $[Ir(cod)Cl]_2/Na_2CO_3$ Catalytic Method

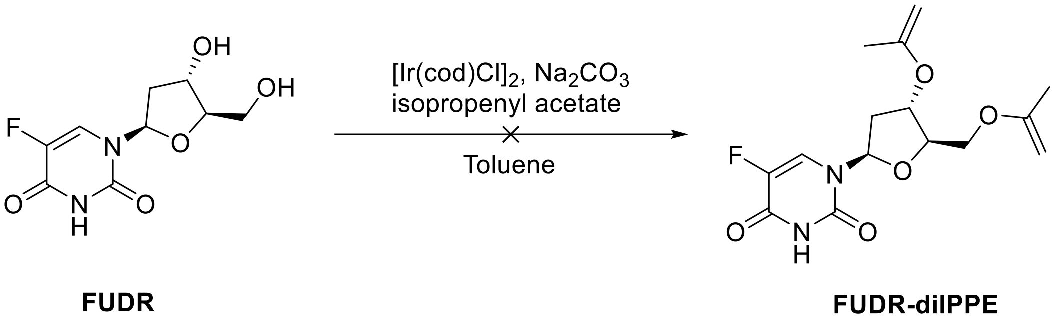

Reaction for synthesis of FUDR-diIPPE was performed under similar condition with above procedures for preparation of PTX-derived IPPE. TLC and NMR analyses indicated that no reaction occurred.

Synthesis of FUDR-Derived IPPE Using TMSOTf/DIPEA Method

Elimination of methanol from FUDR-diMOP using TMSOTf/DIPEA via a two-step process was attempted to synthesize FUDR-diIPPE, according to reported procedures.[7]

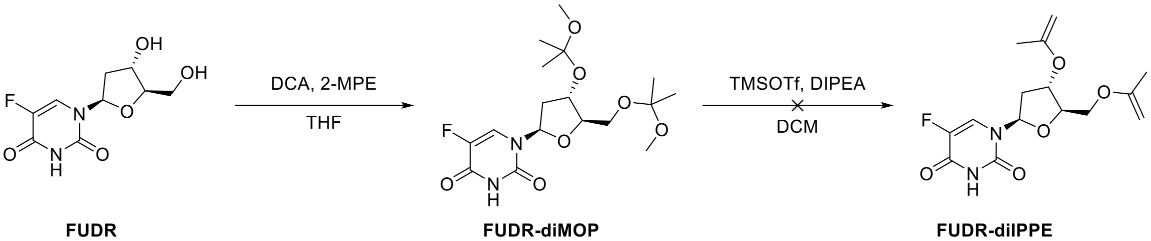

Reaction for synthesis of FUDR-diMOP was performed under similar condition with above procedures for preparation of PTX-diMOP (85.6% yield). **^{1}H NMR** (400 HMz, $CDCl_3$) δ 8.07 (d, *J* = 6.6 Hz, 1H), 6.30 (td, *J* = 6.4, 1.8 Hz, 1H), 4.45 (dt, *J* = 7.2, 3.9 Hz, 1H), 4.09 (q, *J* = 2.7 Hz, 1H), 3.71 (dd, *J* = 10.9, 2.8 Hz, 1H), 3.55 (dd, *J* = 10.9, 2.3 Hz, 1H), 3.19 (d, *J* = 13.3 Hz, 6H), 2.34 (ddd, *J* = 13.5, 6.3, 3.9 Hz, 1H), 2.10 (dt, *J* = 13.3, 6.5 Hz, 1H), 1.38 (d, *J* = 6.5 Hz, 6H), 1.32 (s, 6H).

Reaction for synthesis of FUDR-diIPPE using FUDR-diMOP was performed under similar condition with above procedures for preparation of PTX-derived IPPE. TLC and NMR analyses indicated that FUDR was decomposed and no desired compound was yielded.

7) Experimental and Analytical Data for Isopropenyl Ethers

2-(prop-1-en-2-yloxy)octane (3a)

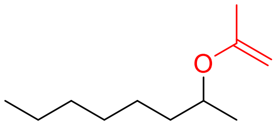

Following the general procedure A (0.5 mol% LPTS, 12 h), the titled compound was isolated by column chromatography (100:1 PE:Et_3N) as a colorless oil in 91% yield. On a large-scale preparation using 50 mmol **1a**, the titled compound was isolated by distillation (20 mbar, 80 °C) in 85% yield. **^{1}H NMR** (400 MHz, C_6D_6) δ 4.01 (m, 1H), 3.93 (d, *J* = 28.9 Hz, 2H), 1.81 (s, 3H), 1.69 – 1.60 (m, 1H), 1.36 (d, *J* = 4.5 Hz, 1H), 1.31 – 1.19 (m, 8H), 1.15 (d, *J* = 6.0 Hz, 3H), 0.88 (t, *J* = 6.9 Hz, 3H). **^{13}C NMR** (100 MHz, C_6D_6) δ 158.2, 81.2, 72.0, 36.3, 31.8, 29.3, 25.6, 22.6, 21.4, 19.0, 13.9. **HRMS** (EI): m/z calcd for $C_{11}H_{22}O$ $[M\text{-}CH_3]^+$: 155.1430; found 155.1430.

PTX-2′-TBS-7-IPPE (3b)

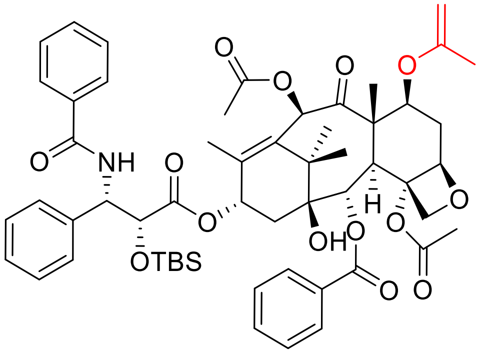

Substrate PTX-2′-TBS was synthesized as previously reported.[23] Following the general procedure A (0.05 M substrate, 20 mol% LPTS, 48 h), the titled compound was isolated by reversed-phase column chromatography (100 g HP C18 using ACN/H_2O as eluent) as white solid in 85% yield. **^{1}H NMR** (400 MHz, CD_3CN) δ 8.16 – 8.09 (m, 2H), 7.81 – 7.76 (m, 2H), 7.69 – 7.64 (m, 1H), 7.61 – 7.56 (m, 2H), 7.56 – 7.49 (m, 2H), 7.49 – 7.37 (m, 7H), 7.34 – 7.24 (m, 1H), 6.21 (s, 1H), 6.11 – 6.04 (m, 1H), 5.71 (dd, *J* = 9.4, 4.5 Hz, 1H), 5.59 (d, *J* = 7.2 Hz, 1H), 5.02 (dd, *J* = 9.8, 2.3 Hz, 1H), 4.81 – 4.71 (m, 2H), 4.21 – 4.14 (m, 2H), 3.99 – 3.92 (m, 2H), 3.86 (d, *J* = 7.2 Hz, 1H), 2.78 (ddd, *J* = 14.5, 9.8, 6.3 Hz, 1H), 2.58 (s, 3H), 2.34 (dd, *J* = 15.4, 9.6 Hz, 1H), 2.10 (s, 3H), 1.79 (d, *J* = 1.4 Hz, 3H), 1.72 (s, 3H), 1.71 – 1.66 (m, 1H), 1.64 (d, *J* = 0.7 Hz, 3H), 1.11 (d, *J* = 1.6 Hz, 6H), 0.81 (s, 9H), -0.03 (s, 3H), -0.15 (s, 3H). **^{13}C NMR** (100 MHz, CD_3CN) δ 202.6, 172.7, 171.5, 169.5, 167.6, 166.6, 159.3, 140.5, 139.2, 135.3, 134.2, 134.0, 132.2, 130.7, 129.4, 129.2, 129.1, 128.6, 128.2, 127.9, 84.3, 81.9, 81.6, 78.6, 76.6, 76.2, 75.6, 75.4, 75.1, 71.9, 57.1, 57.0, 48.4, 44.0, 36.3, 32.0, 26.5, 25.7, 23.3, 21.7, 20.6, 18.5, 14.7, 11.2, -5.2, -5.5. **HRMS** (MALDI): m/z calcd for $C_{56}H_{69}NO_{14}Si$ $[M+Na]^+$: 1030.4380; found: 1030.4382.

FUL-3-TBS-17-IPPE (3c)

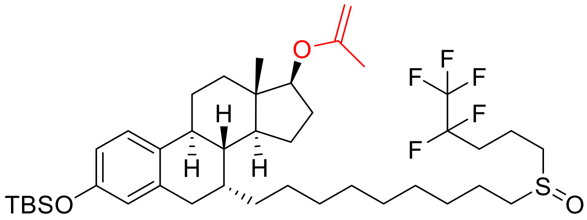

Following the general procedure A (0.05 M substrate, 20 mol% LPTS, 48 h) using FUL-3-TBS as substrate, the titled compound was isolated by reversed-phase column chromatography (100 g HP C18 using ACN/H_2O as eluent) as colorless sticky oil in 60% yield. **^{1}H NMR** (400 MHz, $CDCl_3$) δ 7.11 (d, *J* = 8.6 Hz, 1H), 6.61 (dd, *J* = 8.3, 2.7 Hz, 1H), 6.53 (d, *J* = 2.6 Hz, 1H), 3.97 (dt, *J* = 15.8, 8.3 Hz, 1H), 3.84 (s, 1H), 3.62 (d, *J* = 7.8 Hz, 1H), 2.85 (d, *J* = 16.6 Hz, 1H), 2.76 – 2.68 (m, 4H), 2.67 – 2.60 (m, 2H), 2.33 – 2.20 (m, 5H), 2.20 – 2.12 (m, 4H), 1.95 (d, *J* = 8.2 Hz, 2H), 1.79 (s, 3H), 1.75 (dd, *J* = 16.7, 9.2 Hz, 3H), 1.65 (d, *J* = 17.4 Hz, 3H), 1.51 – 1.35 (m, 8H), 1.34 – 1.27 (m, 7H), 1.18 (d, *J* = 7.5 Hz, 2H), 0.97 (s, 9H), 0.18 (s, 6H). **^{13}C NMR** (100 MHz, $CDCl_3$) δ 159.5, 153.3, 136.7, 132.4, 126.7, 120.8, 117.2, 85.3, 81.9, 52.7, 51.0, 46.4, 43.6, 41.6, 38.1, 37.8, 34.6, 33.3, 30.0, 29.7, 29.6, 29.5, 29.3, 29.2, 28.8, 28.2, 27.9, 27.2, 25.7, 23.1, 22.6, 21.3, 18.1, 14.6, 12.0, -4.4. **HRMS** (ESI): m/z calcd for $C_{41}H_{65}F_5O_3SSi$ $[M+Na]^+$: 783.4236; found 783.4239.

FUL-3-TBS was prepared as follows. To a solution of FUL (121.0 mg, 0.2 mmol) in anhydrous DMF (2 mL) was added imidazole (41 mg, 0.6 mmol) and DMAP (12 mg, 0.1 mmol), then the mixture was cooled to 0 ^oC, then was added TBSCl (39 mg, 0.26 mmol) under N_2 atmosphere, and the resulting reaction mixture was stirred at room temperature. The reaction was monitored by TLC, quenched, and washed with 0.1 M HCl and extracted with EtOAc. The combined organic layer was washed with brine, dried over anhydrous $MgSO_4$ and concentrated in vacuo. The residue was chromatographed on silica gel column with EtOAc/PE (1:10 to 1:1, v/v) as an eluent to offer FUL-3-TBS as colorless sticky oil (104 mg, 71.9%). **^{1}H NMR** (400 MHz, $CDCl_3$) δ 7.15 (d, *J* = 8.4 Hz, 1H), 6.66 (dt, *J* = 8.5, 2.7 Hz, 1H), 6.58 (d, *J* = 2.7 Hz, 1H), 3.68 (t, *J* = 8.2 Hz, 1H), 2.97 – 2.59 (m, 6H), 2.30 (m, 4H), 2.24 – 2.13 (m, 2H), 2.02 – 1.89 (m, 1H), 1.86 (dt, *J* = 12.5, 3.3 Hz, 1H), 1.82 – 1.70 (m, 3H), 1.70 – 1.60 (m, 2H), 1.56 (m, 1H), 1.45 (dd, *J* = 4.7, 2.9 Hz, 5H), 1.38 – 1.30 (m, 4H), 1.31 – 1.21 (m, 8H), 1.20 (d, *J* = 3.6 Hz, 2H), 0.91 (s, 9H), 0.77 (s, 3H), 0.05 (d, *J* = 4.6 Hz, 6H). **^{13}C NMR** (100 MHz, $CDCl_3$) δ 153.3, 136.8, 132.4, 126.7, 120.9, 117.2, 107.8, 82.0, 67.7, 52.6, 50.9, 46.5, 43.4, 41.9, 38.2, 36.9, 34.6, 33.3, 31.4, 30.5, 30.2, 30.0, 29.7, 29.6, 29.4, 29.2, 28.8, 28.2, 27.2, 25.7, 25.6, 23.9, 22.7, 22.6, 18.2, 11.1, -4.4.

FUDR-5′-TBS-3′-IPPE (3d)

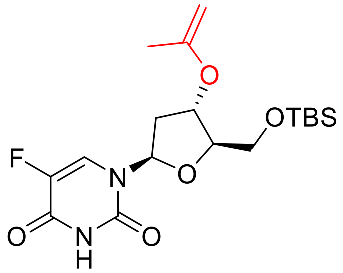

Substrate FUDR-5′-TBS was synthesized as previously reported.[24] Following the general procedure A (0.1 M substrate, 20 mol% LPTS, 48 h), the titled compound was isolated by reversed-phase column chromatography (100 g HP C18 using ACN/H_2O as eluent) as white solid in 65% yield. **^{1}H NMR** (400MHz, CD_3CN) δ 7.86 (d, *J* = 6.8 Hz, 1H), 6.16 (ddd, *J* = 7.9, 5.8, 1.9 Hz, 1H), 4.61 (dd, *J* = 5.3, 2.6 Hz, 1H), 4.13 (q, *J* = 2.7 Hz, 1H), 4.02 – 3.96 (m, 1H), 3.95 – 3.76 (m, 3H), 2.39 (ddd, *J* = 13.9, 5.9, 2.1 Hz, 1H), 2.14 (ddd, *J* = 13.9, 8.1, 5.9 Hz, 1H), 1.79 (s, 3H), 0.92 (s, 9H), 0.12 (s, 6H). **^{13}C NMR** (100MHz, CD_3CN) δ 158.8, 143.1, 140.8, 124.8, 124.4, 86.4, 85.6, 84.1, 77.4, 64.5, 38.5, 26.2, 21.2, 18.9, -5.4. **HRMS** (MALDI): m/z calcd for $C_{18}H_{29}FN_2O_5Si$ $[M+Na]^+$: 423.1722; found: 423.1720.

TAF-3,5-diIPPE (3e)

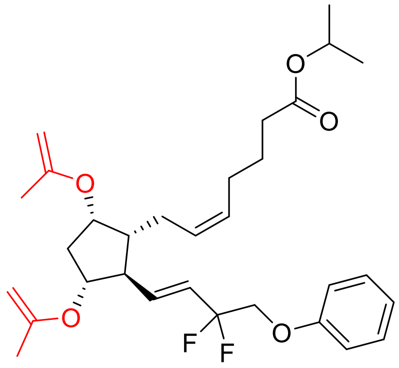

Following the general procedure A (0.1 M substrate, 2 mol% LPTS, 24 h), the titled compound was isolated by column chromatography (100:0.5 PE:Et_3N) as yellow oil in 67% yield. **^{1}H NMR** (400MHz, $CDCl_3$) δ 7.34 – 7.24 (m, 2H), 6.99 (tt, *J* = 7.4, 1.1 Hz, 1H), 6.91 (dt, *J* = 7.1, 1.1 Hz, 2H), 6.14 (ddt, *J* = 15.8, 8.8, 2.4 Hz, 1H), 5.90 – 5.73 (m, 1H), 5.37 – 5.28 (m, 2H), 5.04 – 4.92 (m, 1H), 4.27 (t, *J* = 4.8 Hz, 1H), 4.19 (t, *J* = 11.6 Hz, 3H), 3.90 – 3.78 (m, 2H), 3.67 (dd, *J* = 4.3, 1.7 Hz, 2H), 2.88 – 2.71 (m, 1H), 2.42 – 2.27 (m, 2H), 2.29 – 2.18 (m, 2H), 2.04 (ddt, *J* = 14.6, 12.7, 5.9 Hz, 3H), 1.90 (ddd, *J* = 15.3, 4.0, 1.1 Hz, 1H), 1.78 (s, 3H), 1.74 (s, 3H), 1.70 – 1.54 (m, 3H), 1.21 (d, *J* = 6.3 Hz, 6H). **^{13}C NMR** (100MHz, $CDCl_3$) δ 173.1, 158.9, 158.0, 157.6, 138.2, 138.1, 138.0, 130.1, 129.6, 128.2, 121.7, 114.8, 82.7, 82.2, 80.6, 75.4, 67.4, 51.8, 47.8, 37.0, 34.1, 26.5, 24.9, 24.8, 24.5, 21.8, 21.2, 21.1, 21.0. **HRMS** (ESI): m/z calcd for $C_{31}H_{42}F_2O_5$ $[M+Na]^+$: 555.2893; found 555.2890.

ZDV-IPPE (3f)

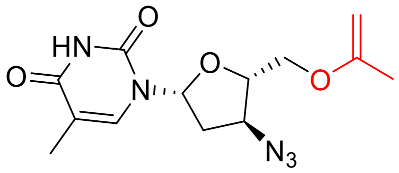

Following the general procedure A (2 mol% LPTS, 24 h), the titled compound was isolated by column chromatography (EtOAc/PE, 1:20 to 1:2, v/v, containing 0.5% Et_3N) as white solid in 52% yield. **^{1}H NMR** (400MHz, $CDCl_3$) δ 8.70 (s, 1H), 7.42 (s, 1H), 6.25 (t, *J* = 6.4 Hz, 1H), 4.37 – 4.29 (m, 1H), 4.15 (m, 1H), 4.09 – 3.83 (m, 4H), 2.48 (m, 1H), 2.34 (m, 1H), 1.93 (d, *J* = 1.2 Hz, 3H), 1.88 (d, *J* = 0.8 Hz, 3H). **^{13}C NMR** (100MHz, $CDCl_3$) δ 163.6, 158.7, 150.2, 135.2, 111.1, 85.1, 83.3, 82.6, 66.6, 60.9, 38.2, 20.9, 12.7. **HRMS** (ESI): m/z calcd for $C_{13}H_{17}N_5O_4$ $[M+Na]^+$: 330.1173; found 330.1176.

LOV-IPPE (3g)

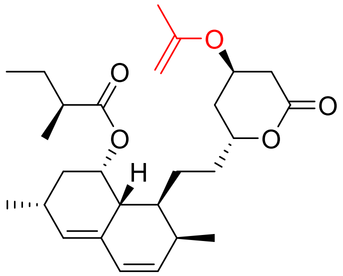

Following the general procedure A (2 mol% LPTS, 24 h), the titled compound was isolated by column chromatography (EtOAc/PE, 1:200 to 1:50, v/v, containing 0.5% Et_3N) as white solid in 63% yield. **^{1}H NMR** (400MHz, C_6D_6) δ 6.06 (d, *J* = 9.6 Hz, 1H), 5.73 (m, 1H), 5.39 (d, *J* = 33.7 Hz, 2H), 4.41 – 4.26 (m, 1H), 3.87 (d, *J* = 8.9 Hz, 2H), 3.55 (d, *J* = 2.1 Hz, 1H), 2.53 (m, 1H), 2.33 – 2.19 (m, 4H), 2.06 – 1.93 (m, 2H), 1.77 (t, *J* = 9.9 Hz, 2H), 1.73 – 1.61 (m, 3H), 1.59 (s, 3H), 1.53 – 1.31 (m, 3H), 1.21 – 1.11 (m, 2H), 1.09 (d, *J* = 7.2 Hz, 6H), 0.80 (t, *J* = 6.8 Hz, 6H). **^{13}C NMR** (100MHz, C_6D_6) δ 175.5, 167.7, 156.9, 132.7, 131.9, 129.5, 128.8, 83.0, 75.3, 67.4, 66.8, 46.5, 41.2, 37.2, 36.8, 35.0, 33.0, 32.5, 30.8, 27.6, 26.8, 23.9, 22.7, 20.7, 16.2, 13.7, 11.4. **HRMS** (ESI): m/z calcd for $C_{27}H_{40}O_5$ $[M+H]^+$: 445.2949; found 445.2950.

ABI-IPPE (3h)

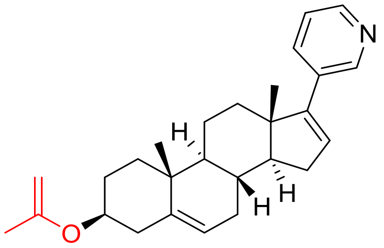

Following the general procedure A (0.2 M substrate, 2 mol% LPTS, 24 h), the titled compound was isolated by column chromatography (EtOAc/PE, 1:40 to 1:10, v/v, containing 0.5% Et_3N) as white solid in 91% yield. **^{1}H NMR** (400MHz, C_6D_6) δ 8.90 (s, 1H), 8.52 (s, 1H), 7.39 – 7.34 (m,

1H), 6.81 – 6.75 (m, 1H), 5.75 (s, 1H), 5.33 (d, J = 4.1 Hz, 1H), 4.03 (s, 2H), 3.96 (m, J = 10.9, 6.2 Hz, 1H), 2.72 – 2.64 (m, 1H), 2.43 (t, J = 12.3 Hz, 1H), 2.07 – 1.96 (m, 2H), 1.85 (d, J = 7.6 Hz, 6H), 1.58 (m, 4H), 1.36 (m, 4H), 0.94 – 0.86 (m, 8H). **^{13}C NMR** (100MHz, C_6D_6) δ 157.9, 151.9, 148.3, 140.7, 133.0, 132.7, 128.7, 122.7, 121.6, 81.7, 75.6, 57.4, 50.3, 47.1, 38.5, 37.0, 36.9, 35.1, 31.6, 30.3, 27.9, 21.5, 20.7, 19.0, 16.3. **HRMS** (ESI): m/z calcd for $C_{27}H_{35}NO$ $[M+H]^+$: 390.2791; found 390.2793.

PPT-IPPE (3i)

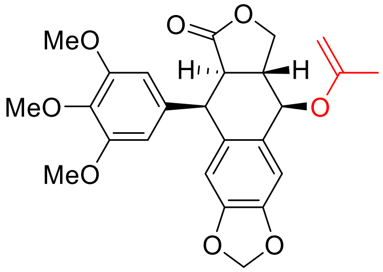

Following the general procedure A (0.2 M substrate, 5 mol% LPTS, 48 h), the titled compound was isolated by column chromatography (EtOAc/PE, 1:20 to 1:3, v/v, containing 0.5% Et_3N) as white solid in 50% yield. **^{1}H NMR** (400MHz, C_6D_6) δ 7.02 (s, 1H), 6.67 (s, 2H), 6.45 (s, 1H), 5.26 (d, J = 20.9 Hz, 2H), 4.70 (d, J = 9.3 Hz, 1H), 4.43 (d, J = 4.4 Hz, 1H), 3.89 (d, J = 7.5 Hz, 1H), 3.85 (s, 1H), 3.79 (s, 3H), 3.77 (s, 1H), 3.47 (s, 6H), 3.38 (m, 1H), 2.84 – 2.72 (m, 1H), 2.09 (m, 1H), 1.62 (s, 3H). **^{13}C NMR** (100MHz, C_6D_6) δ 172.9, 159.7, 153.4, 147.9, 147.6, 138.5, 135.2, 132.3, 130.8, 109.6, 108.8, 107.3, 101.1, 82.4, 75.7, 70.4, 60.1, 55.6, 45.1, 44.0, 39.2, 21.1. **HRMS** (ESI): m/z calcd for $C_{25}H_{26}O_8$ $[M+H]^+$: 455.1700; found 455.1703.

TEST-IPPE (3j)

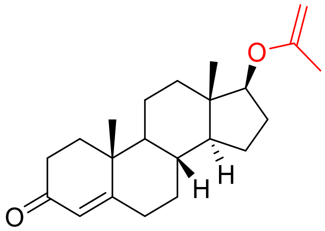

Following the general procedure A (0.5 mol% LPTS, 12 h), the titled compound was isolated by column chromatography (EtOAc/PE, 1:200 to 1:50, v/v, containing 0.5% Et_3N) as white solid in 75% yield. **^{1}H NMR** (400MHz, C_6D_6) δ 5.83 (s, 1H), 4.00 (d, J = 9.9 Hz, 2H), 3.82 (t, J = 8.1 Hz, 1H), 1.86– 2.30 (m, 6H), 1.84 (s, 3H), 1.35 – 1.57 (m, 4H), 1.04 – 1.27 (m, 5H), 0.88 – 0.97 (m, 1H), 0.85 (s, 3H), 0.70 (s, 3H), 0.49 – 0.68(m, 3H). **^{13}C NMR** (100MHz, C_6D_6) δ 196.8, 168.2, 159.1, 124.2, 85.0, 82.4, 53.5, 49.9, 42.8, 38.0, 37.2, 35.6, 35.0, 33.9, 32.2, 31.4, 27.9, 23.6, 21.1, 20.4, 17.0, 12.0. **HRMS** (ESI): m/z calcd for $C_{22}H_{32}O_2$ $[M+H]^+$: 329.2475; found 329.2478.

IDBN-IPPE (3k)

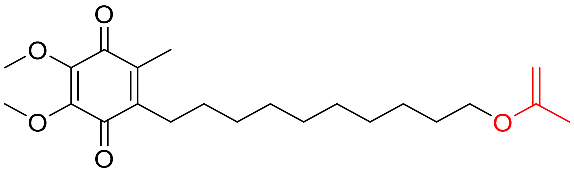

Following the general procedure A (0.5 mol% LPTS, 12 h), the titled compound was isolated by column chromatography (EtOAc/PE, 1:200 to 1:50, v/v, containing 0.5% Et_3N) as yellow sticky oil in 77% yield. **^{1}H NMR** (400MHz, C_6D_6) δ 3.93 (d, *J* = 4.0 Hz, 2H), 3.61 (s, 6H), 3.57 (t, *J* = 6.4 Hz, 2H), 2.34 – 2.28 (m, 2H), 1.83 (s, 3H), 1.77 (s, 3H), 1.64 – 1.56 (m, 2H), 1.32 (m, 4H), 1.23 (s, 10H). **^{13}C NMR** (100MHz, C_6D_6) δ 184.0, 183.6, 160.0, 144.7, 142.4, 138.1, 127.9, 81.0, 67.1, 60.3, 60.2, 29.9, 29.6, 29.5, 29.4, 29.3, 29.1, 28.7, 26.3, 26.2, 20.9, 11.4. **HRMS** (ESI): m/z calcd for $C_{22}H_{34}O_5$ $[M+H]^+$: 379.2479; found 379.2479.

Vitamin E-IPPE (3l)

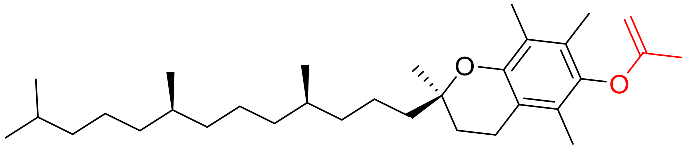

Following the general procedure A (5 mol% LPTS, 12 h), the titled compound was isolated by reversed-phase column chromatography (100 g HP C18 using ACN/H_2O as eluent) as yellow oil in 63% yield. **^{1}H NMR** (400MHz, C_6D_6) δ 3.93 (s, 1H), 3.77 (s, 1H), 2.41 (m, 2H), 2.29 (s, 3H), 2.18 (s, 3H), 2.13 (s, 3H), 1.97 (s, 3H), 1.64 (m, 2H), 1.42– 1.56 (m, 8H), 1.35 (d, *J* = 4.2 Hz, 6H), 1.20 – 1.10 (m, 10H), 0.93 (m, 12H). **^{13}C NMR** (100MHz, C_6D_6) δ 158.6, 148.7, 144.2, 134.3, 125.6, 123.0, 117.4, 84.3, 74.5, 40.1, 39.4, 37.5, 37.4, 32.9, 32.7, 31.2, 28.0, 24.9, 24.6, 23.6, 22.6, 22.5, 21.1, 20.6, 19.7, 19.6, 19.5, 19.4, 12.6, 11.9, 11.8. **HRMS** (ESI): m/z calcd for $C_{32}H_{54}O_2$ $[M+H]^+$: 471.4197; found 471.4194.

12-methyl-2,5,8,11-tetraoxatridec-12-ene (3m, n = 3)

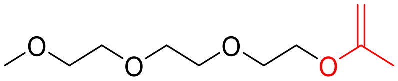

Following the general procedure A (2 mol% LPTS, 24 h), the titled compound was isolated by column chromatography (100:1:1 DCM:MeOH:Et_3N) as a colorless liquid in 75% yield. **^{1}H NMR** (400 MHz, C_6D_6) δ 3.87 (s, 1H), 3.82 (s, 1H), 3.67 – 3.62 (m, 2H), 3.52 – 3.48 (m, 2H), 3.47 – 3.44 (m, 6H), 3.36 – 3.31 (m, 2H), 3.12 (s, 3H), 1.77 (s, 3H). **^{13}C NMR** (100 MHz, C_6D_6) δ 159.7, 81.2, 72.0, 70.7, 70.6, 69.4, 66.8, 58.3, 20.7. **HRMS** (EI): m/z calcd for $C_{10}H_{20}O_4$ $[M+H]^+$: 205.1434; found 205.1437.

mPEG$_{550}$-IPPE (3m, n = 13)

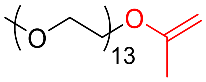

Following the general procedure A (0.2 M substrate, 2 mol% LPTS, 24 h), the titled compound was isolated by column chromatography (100:1:1 DCM:MeOH:Et_3N) to give a mixture of **3m** and **3m′** (**3m**:**3m′** = 2:1) as a white solid. ^{1}**H NMR** (400 MHz, $CDCl_3$) δ 3.86 – 3.73 (m, 5H), 3.65 (d, *J* = 4.6 Hz, 42H), 3.55 (m, 3H), 3.38 (s, 3H), 3.20 (s, 1H), 1.83 (s, 2H), 1.35 (s, 2H).

mPEG$_{2000}$-IPPE (3m, n = 45)

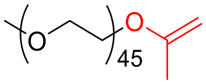

Following the general procedure A (0.05 M substrate, 2 mol% LPTS, 24 h), the titled compound was isolated by column chromatography (100:1:1 DCM:MeOH:Et_3N) to give a mixture of **3m** and **3m′** (**3m**:**3m′** = 5:1) as a white solid. ^{1}**H NMR** (400 MHz, $CDCl_3$) δ 3.86 – 3.80 (m, 5H), 3.64 (s, 200H), 3.55 (d, *J* = 3.0 Hz, 2H), 3.38 (s, 3H), 3.20 (s, 0.5H), 1.83 (s, 2.5H), 1.35 (s, 1H).

mPEG$_{2000}$-PCL$_{1800}$-IPPE (3n)

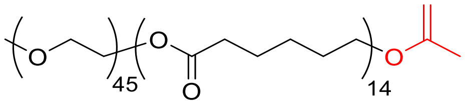

Following the general procedure A (0.02 M substrate, 2 mol% LPTS, 24 h), the titled compound was isolated by column chromatography (100:1:1 to 100:10:1 DCM:MeOH:Et_3N) to give a mixture of **3n** and **3n′** (**3n**:**3n′** = 6:1) as a white solid. ^{1}**H NMR** (400 MHz, $CDCl_3$) δ 4.25 – 4.20 (m, 2H), 4.06 (t, *J* = 6.7 Hz, 26H), 3.81 (d, *J* = 7.0 Hz, 2H), 3.66 (s, 155H), 3.38 (s, 3H), 3.18 (s, 0.4H) 2.31 (t, *J* = 7.5 Hz, 28H), 1.81 (s, 2.6H), 1.65 (m, 56H), 1.44 – 1.32 (m, 28H).

(3*S*,8*S*,9*S*,10*R*,13*R*,14*S*,17*R*)-10,13-dimethyl-17-((*R*)-6-methylheptan-2-yl)-3-(prop-1-en-2-yloxy)-2,3,4,7,8,9,10,11,12,13,14,15,16,17-tetradecahydro-1*H*-cyclopenta[*a*]phenanthrene (3o)

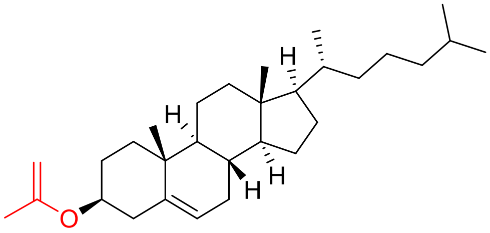

Following the general procedure A (0.5 mol% LPTS, 12 h), the titled compound was isolated by column chromatography (100:1 PE:Et_3N) as a white solid in 92% yield. ^{1}**H NMR** (400 MHz, C_6D_6) δ 5.33 – 5.39 (m, 1H), 3.93 – 4.05 (m, 3H), 2.68 (m, 1H), 2.40 – 2.50 (m, 1H), 2.00 – 2.09 (m, 2H), 1.93 (m, 1H), 1.86 (s, 3H), 1.50 – 1.73 (m, 6H), 1.36 – 1.46 (m, 6H), 0.98 – 1.31 (m, 14H), 0.92 – 0.95 (m, 9H), 0.67 (s, 3H). ^{13}**C NMR** (100 MHz, C_6D_6) δ 157.9, 140.4, 121.9, 81.6, 75.7,

56.7, 56.2, 50.2, 42.3, 39.9, 39.6, 38.5, 37.2, 36.8, 36.4, 35.9, 32.0, 31.9, 28.3, 28.1, 28.0, 24.3, 24.0, 22.7, 22.4, 21.5, 21.1, 19.1, 18.7, 11.7. **HRMS** (ESI): m/z calcd for $C_{30}H_{50}O$ [M+Na]$^+$: 449.3754; found 449.3759.

(*Z*)-1-(prop-1-en-2-yloxy)octadec-9-ene (3p)

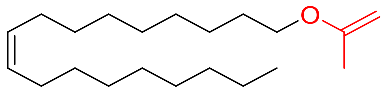

Following the general procedure A (0.5 mol% LPTS, 12 h), the titled compound was isolated by column chromatography (100:1 PE:Et_3N) as a colorless oil in 83% yield. **^{1}H NMR** (400 MHz, C_6D_6) δ 5.43 – 5.56 (m, 2H), 3.92 (d, *J* = 5.9 Hz, 2H), 3.55 (t, *J* = 6.5 Hz, 2H), 2.00 – 2.20 (m, 4H), 1.83 (s, 3H), 1.58 (m, 2H), 1.32 (m, 22H), 0.91 (t, *J* = 6.8 Hz, 3H). **^{13}C NMR** (100 MHz, C_6D_6) δ 159.9, 129.9, 129.8, 80.9, 67.1, 31.9, 29.9, 29.8, 29.6, 29.5, 29.4, 29.3, 29.1, 27.3, 26.2, 22.7, 20.9, 14.0. **HRMS** (ESI): m/z calcd for $C_{21}H_{40}O$ [M+H]$^+$: 309.3152; found 309.3150.

(1*S*,2*R*,4*R*)-1-isopropyl-4-methyl-2-(prop-1-en-2-yloxy)cyclohexane (3q)

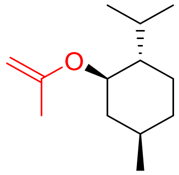

Following the general procedure A (0.5 mol% LPTS, 12 h), the titled compound was isolated by column chromatography (100:1 PE:Et_3N) as a colorless oil in 92% yield. On a large-scale preparation using 50 mmol **1q**, the titled compound was isolated by distillation (20 mbar, 120 ^{o}C) in 94% yield. **^{1}H NMR** (400 MHz, C_6D_6) δ 3.96 (d, *J* = 4.5 Hz, 2H), 3.79 (m, 1H), 2.20 – 2.35 (m, 2H), 1.80 (s, 3H), 1.48 – 1.56 (m, 2H), 1.42 (m, 1H), 1.13 – 1.25 (m, 1H), 0.93 (m, 5H), 0.83 (m, 6H), 0.79 – 0.69 (m, 1H). **^{13}C NMR** (100 MHz, C_6D_6) δ 158.2, 80.7, 75.7, 47.9, 39.6, 34.6, 31.1, 26.4, 23.9, 22.0, 21.4, 20.5, 16.7. **HRMS** (EI): m/z calcd for $C_{13}H_{24}O$ [M-CH_3]$^+$: 181.1587; found 181.1587.

7-(prop-1-en-2-yloxy)hept-1-yne (3r)

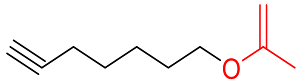

Following the general procedure A (0.5 mol% LPTS, 12 h), the titled compound was isolated by column chromatography (100:1 PE:Et_3N) as a colorless liquid in 84% yield. **^{1}H NMR** (400 MHz, C_6D_6) δ 3.91 (s, 1H), 3.86 (s, 1H), 3.43 (t, *J* = 6.4 Hz, 2H), 1.89 (m, 2H), 1.80 (d, *J* = 0.7 Hz, 3H), 1.76 (t, *J* = 2.7 Hz, 1H), 1.42 (m, 2H), 1.27 (m, 4H). **^{13}C NMR** (100 MHz, C_6D_6) δ 159.8, 83.9, 81.0, 68.6, 66.8, 28.5, 28.1, 25.3, 20.9, 18.2. **HRMS** (EI): m/z calcd for $C_{10}H_{16}O$ [M-CH_3]$^+$: 137.0961; found 137.0960.

2-(4-(prop-1-en-2-yloxy)butyl)oxirane (3s)

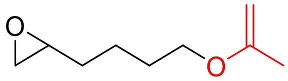

Following the general procedure A (2 mol% LPTS, 24 h), the titled compound was isolated by column chromatography (100:1 PE:Et_3N) as a colorless oil in 82% yield. **^{1}H NMR** (400 MHz, C_6D_6) δ 3.70 (d, *J* = 16.1 Hz, 2H), 3.26 (t, *J* = 6.3 Hz, 2H), 2.38 – 2.30 (m, 1H), 2.12 (dd, *J* = 5.3, 3.9 Hz, 1H), 1.85 (dd, *J* = 5.3, 2.6 Hz, 1H), 1.61 (s, 3H), 1.33 – 1.26 (m, 2H), 1.13 (dd, *J* = 9.8, 5.0 Hz, 2H), 1.05 (dd, *J* = 7.4, 4.7 Hz, 2H). **^{13}C NMR** (100 MHz, C_6D_6) δ 159.9, 81.0, 66.8, 51.3, 45.9, 32.1, 28.7, 22.7, 20.8. **HRMS** (EI): m/z calcd for $C_9H_{16}O_2$ $[M-CH_3]^+$: 141.0910; found 141.0908.

3-(prop-1-en-2-yloxy)propyl acrylate (3t)

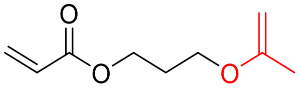

Following the general procedure A (2 mol% LPTS, 24 h), the titled compound was isolated by column chromatography (100:1 PE:Et_3N) as a colorless oil in 62% yield. **^{1}H NMR** (400 MHz, C_6D_6) δ 6.27 (dd, *J* = 17.3, 1.6 Hz, 1H), 5.92 (dd, *J* = 17.3, 10.4 Hz, 1H), 5.22 (dd, *J* = 10.4, 1.6 Hz, 1H), 4.12 (t, *J* = 6.5 Hz, 2H), 3.90 – 3.77 (m, 2H), 3.45 (t, *J* = 6.2 Hz, 2H), 1.78 – 1.66 (m, 5H). **^{13}C NMR** (100 MHz, C_6D_6) δ 165.3, 159.6, 129.8, 128.4, 81.3, 63.4, 61.2, 28.3, 20.6. **HRMS** (EI): m/z calcd for $C_9H_{14}O_3$ $[M-CH_3]^+$: 155.0703; found 155.0707.

1-bromo-6-(prop-1-en-2-yloxy)hexane (3u)

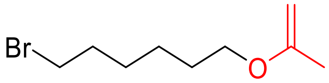

Following the general procedure A (0.5 mol% LPTS, 12 h), the titled compound was isolated by column chromatography (100:1 PE:Et_3N) as a faint yellow oil in 70% yield. **^{1}H NMR** (400 MHz, C_6D_6) δ 3.93 (s, 1H), 3.89 (s, 1H), 3.45 (t, *J* = 6.4 Hz, 2H), 2.91 (t, *J* = 6.8 Hz, 2H), 1.81 (s, 3H), 1.42 (m, *J* = 13.0, 6.5 Hz, 4H), 1.08 (m, *J* = 6.9, 3.3 Hz, 4H). **^{13}C NMR** (100 MHz, C_6D_6) δ 159.9, 81.0, 66.8, 33.1, 32.5, 28.7, 27.7, 25.2, 20.8. **HRMS** (ESI): m/z calcd for $C_9H_{17}BrO$ $[M+H]^+$: 221.0536; found 221.0533.

1,4-bis(prop-1-en-2-yloxy)cyclohexane (3v)

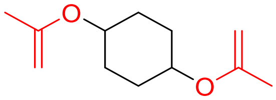

Following the general procedure A (0.5 mol% LPTS, 12 h), the titled compound was isolated by column chromatography (100:1 PE:Et_3N) as a colorless oil in 66% yield. **^{1}H NMR** (400 MHz, C_6D_6) δ 3.82 – 3.98 (m, 3H), 1.91 (m, 2H), 1.78 (s, 3H), 1.39 – 1.50 (m, 2H). **^{13}C NMR** (100 MHz,

C_6D_6) δ 157.7, 81.8, 72.4, 27.3, 26.8, 21.4. **HRMS** (EI): m/z calcd for $C_{12}H_{20}O_2$ $[M-CH_3]^+$: 181.1223; found 181.1222.

1-(6-(prop-1-en-2-yloxy)hexyl)-1*H*-pyrrole-2,5-dione (3w)

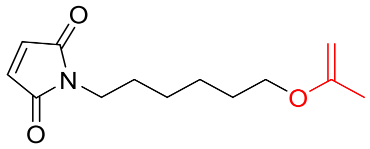

Following the general procedure A (2 mol% LPTS, 24 h), the titled compound was isolated by column chromatography (100:1 PE:Et_3N) as a white solid in 83% yield. **^{1}H NMR** (400 MHz, C_6D_6) δ 5.78 (s, 2H), 3.88 (d, *J* = 14.1 Hz, 2H), 3.45 (t, *J* = 6.4 Hz, 2H), 3.26 (t, *J* = 7.2 Hz, 2H), 1.79 (s, 3H), 1.46 – 1.32 (m, 4H), 1.16 (m, 2H), 1.06 – 0.97 (m, 2H). **^{13}C NMR** (100 MHz, C_6D_6) δ 170.1, 159.9, 133.1, 81.0, 66.9, 37.3, 28.9, 28.4, 26.4, 25.6, 20.9. **HRMS** (ESI): m/z calcd for $C_{13}H_{19}NO_3$ $[M+Na]^+$: 260.1257; found 260.1259.

2-((prop-1-en-2-yloxy)methyl)furan (3x)

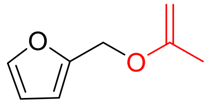

Following the general procedure A (2 mol% LPTS, 24 h), the titled compound was isolated by column chromatography (100:1 PE:Et_3N) as a colorless oil in 50% yield. **^{1}H NMR** (400 MHz, C_6D_6) δ 7.05 (s, 1H), 6.05 (m, 2H), 4.46 (s, 2H), 3.88 (d, *J* = 8.6 Hz, 2H), 1.73 (s, 3H). **^{13}C NMR** (100 MHz, C_6D_6) δ 159.4, 150.9, 142.5, 110.2, 109.3, 81.7, 61.5, 20.6. **HRMS** (EI): m/z calcd for $C_8H_{10}O_2$ $[M-CH_3]^+$: 123.0441; found 123.0441.

5-(prop-1-en-2-yloxy)bicyclo[2.2.1]hept-2-ene (3y)

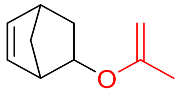

Following the general procedure A (0.5 mol% LPTS, 12 h), the titled compound was isolated by column chromatography (100:1 PE:Et_3N) as a colorless oil (mixture of *endo* and *exo* isomers) in 85% yield. **^{1}H NMR** (400 MHz, C_6D_6) δ 6.18 (m, 1H, major), 6.10 (m, 1H, major), 6.00 (m, 1H, minor), 5.75 (m, 1H, minor), 4.48 (m, H, major), 3.94 (s, 2H, major, minor), 3.88 (m, H, minor), 3.06 (s, H, major), 2.97 (s, 1H, minor), 2.58 (s, 1H, minor), 2.55 (s, 1H, major), 1.79 (s, 3H, minor), 1.77 (m, 1H, major), 1.73 (s, 3H, major), 1.55 (m, 1H, minor), 1.47 – 1.45 (m, 1H, minor), 1.36 – 1.31 (m, 1H, major), 1.04 – 0.97 (m, 2H, major), 0.91 – 0.83 (m, 2H, minor). **^{13}C NMR** (100 MHz, C_6D_6) δ 158.9 (major), 158.7 (minor), 140.7 (minor), 137.5 (major), 132.6 (minor), 132.2 (major), 82.3 (major, minor), 76.9 (major), 76.9 (minor), 47.1 (major), 47.0 (minor), 46.4 (minor), 45.8 (major), 42.3 (major), 40.6 (minor), 34.7 (minor), 34.6 (major), 21.1 (minor), 21.0 (major). **HRMS** (EI): m/z calcd for $C_{10}H_{14}O$ $[M+H]^+$: 151.1117; found 151.1117.

(*Z*)-5-(prop-1-en-2-yloxy)cyclooct-1-ene (3z)

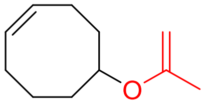

Following the general procedure A (0.5 mol% LPTS, 12 h), the titled compound was isolated by column chromatography (100:1 PE:Et_3N) as a colorless oil in 93% yield. **^{1}H NMR** (400 MHz, C_6D_6) δ 5.60 – 5.46 (m, 2H), 4.02 – 4.09 (m, 1H), 3.99 (s, 1H), 3.83 (s, 1H), 2.10 – 2.21 (m, 1H), 1.96 (m, 4H), 1.80 (s, 3H), 1.68 (m, 2H), 1.40 – 1.48 (m, 1H), 1.25 – 1.38 (m, 2H). **^{13}C NMR** (100 MHz, C_6D_6) δ 157.8, 129.9, 129.5, 81.8, 76.7, 34.4, 32.9, 25.7, 25.3, 22.2, 21.4. **HRMS** (EI): m/z calcd for $C_{11}H_{18}O$ $[M-CH_3]^+$: 151.1117; found 151.1117.

(3*s*,5*s*,7*s*)-1-(prop-1-en-2-yloxy)adamantane (3aa)

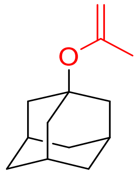

Following the general procedure A (0.5 mol% LPTS, 12 h), the titled compound was isolated by column chromatography (100:1 PE:Et_3N) as a colorless oil in 91% yield. **^{1}H NMR** (400 MHz, C_6D_6) δ 4.28 (d, *J* = 18.7 Hz, 2H), 2.00 (d, *J* = 2.5 Hz, 6H), 1.96 (s, 3H), 1.80 (s, 3H), 1.46 (m, 6H). **^{13}C NMR** (100 MHz, C_6D_6) δ 155.7, 91.7, 76.1, 42.1, 36.3, 30.8, 23.8. **HRMS** (ESI): m/z calcd for $C_{13}H_{20}O$ $[M+H]^+$:193.1587; found 193.1583.

6-(prop-1-en-2-yloxy)hexyl acetate (3ab)

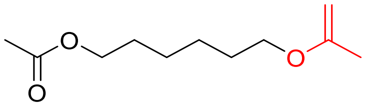

Following the general procedure A (2 mol% LPTS, 24 h), the titled compound was isolated by column chromatography (100:1 PE:Et_3N) as a colorless oil in 77% yield. **^{1}H NMR** (400 MHz, C_6D_6) δ 3.86 – 3.98 (m, 4H), 3.48 (t, *J* = 6.4 Hz, 2H), 1.81 (s, 3H), 1.69 (s, 3H), 1.44 – 1.53 (m, 2H), 1.33 – 1.42 (m, 2H), 1.15 (m, 4H). **^{13}C NMR** (100 MHz, C_6D_6) δ 169.7, 159.9, 81.0, 66.8, 63.9, 28.9, 28.5, 25.8, 25.6, 20.8, 20.2. **HRMS** (EI): m/z calcd for $C_{11}H_{20}O_3$ $[M-CH_3]^+$: 185.1172; found 185.1171.

1-(prop-1-en-2-yloxy)dodecane (3ac)

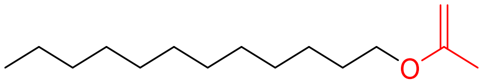

Following the general procedure A (0.5 mol% LPTS, 12 h), the titled compound was isolated by column chromatography (100:1 PE:Et_3N) as a white solid in 89% yield. **^{1}H NMR** (400 MHz, C_6D_6) δ 3.92 (d, *J* = 4.8 Hz, 2H), 3.56 (t, *J* = 6.5 Hz, 2H), 1.82 (s, 3H), 1.64 – 1.55 (m, 2H), 1.27 (d, *J* = 11.5 Hz, 18H), 0.94 – 0.89 (m, 3H). **^{13}C NMR** (100 MHz, C_6D_6) δ 160.0, 81.0, 67.1, 32.0, 30.0,

29.7, 29.5, 29.2, 26.3, 22.8, 20.9, 14.0. **HRMS** (ESI): m/z calcd for $C_{15}H_{30}O$ $[M+H]^+$: 227.2369; found 227.2367.

4-((prop-1-en-2-yloxy)methyl)benzonitrile (3ad)

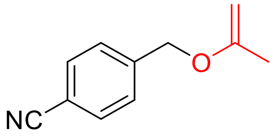

Following the general procedure A (2 mol% LPTS, 24 h), the titled compound was isolated by column chromatography (100:1 PE:Et_3N) as a colorless oil in 81% yield. **^{1}H NMR** (400 MHz, C_6D_6) δ 7.01 (d, *J* = 8.1 Hz, 2H), 6.78 (d, *J* = 8.0 Hz, 2H), 4.26 (s, 2H), 3.91 (s, 1H), 3.77 (s, 1H), 1.75 (s, 3H). **^{13}C NMR** (100 MHz, C_6D_6) δ 159.1, 142.0, 131.7, 127.1, 118.5, 111.5, 82.6, 67.9, 20.5. **HRMS** (EI): m/z calcd for $C_{11}H_{11}NO$ $[M-CH_3]^+$: 158.0600; found 158.0601.

(((6-(prop-1-en-2-yloxy)hexyl)oxy)methyl)benzene (3ae)

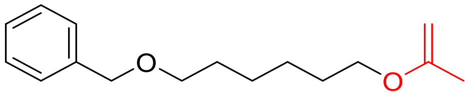

Following the general procedure A (2 mol% LPTS, 24 h), the titled compound was isolated by column chromatography (100:1 PE:Et_3N) as a colorless oil in 75% yield. **^{1}H NMR** (400 MHz, C_6D_6) δ 7.31 (d, *J* = 7.5 Hz, 2H), 7.19 (d, *J* = 7.2 Hz, 2H), 7.10 (t, *J* = 7.3 Hz, 1H), 4.33 (s, 2H), 3.90 (d, *J* = 11.3 Hz, 2H), 3.51 (t, *J* = 6.4 Hz, 2H), 3.27 (t, *J* = 6.4 Hz, 2H), 1.81 (s, 3H), 1.53 (m, 4H), 1.28 (m, 4H). **^{13}C NMR** (100 MHz, C_6D_6) δ 159.9, 139.3, 128.2, 127.4, 127.2, 81.0, 72.6, 70.1, 67.0, 29.8, 29.1, 26.1, 20.9. **HRMS** (EI): m/z calcd for $C_{16}H_{24}O_2$ $[M-CH_3]^+$: 233.1536; found 233.1535.

6-(prop-1-en-2-yloxy)hex-1-ene (3af)

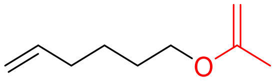

Following the general procedure A (0.5 mol% LPTS, 12 h), the titled compound was isolated by column chromatography (100:1 PE:Et_3N) as a colorless liquid in 70% yield. **^{1}H NMR** (400 MHz, C_6D_6) δ 5.62 – 5.77 (m, 1H), 4.92 – 5.03 (m, 2H), 3.90 (d, *J* = 13.3 Hz, 2H), 3.49 (t, *J* = 6.4 Hz, 2H), 1.91 (m, 2H), 1.80 (s, 3H), 1.48 – 1.57 (m, 2H), 1.36 (m, 2H). **^{13}C NMR** (100 MHz, C_6D_6) δ 159.9, 138.4, 114.4, 80.9, 66.8, 33.4, 28.5, 25.4, 20.8. **HRMS** (EI): m/z calcd for $C_9H_{16}O$ $[M-CH_3]^+$: 125.0961; found 125.0961.

(*Z*)-3,7-dimethyl-1-(prop-1-en-2-yloxy)octa-2,6-diene (3ag)

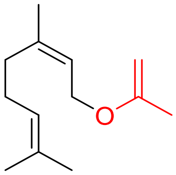

Following the general procedure A (0.5 mol% LPTS, 12 h), the titled compound was isolated by column chromatography (100:1 PE:Et_3N) as a colorless oil in 83% yield. **^{1}H NMR** (400 MHz, C_6D_6) δ 5.54 (t, *J* = 6.5 Hz, 1H), 5.12 (t, *J* = 7.0 Hz, 1H), 4.27 (d, *J* = 6.6 Hz, 2H), 3.95 (d, *J* = 2.6 Hz, 2H), 2.08 – 1.97 (m, 4H), 1.83 (s, 3H), 1.63 (d, *J* = 6.1 Hz, 6H), 1.51 (s, 3H). **^{13}C NMR** (100 MHz, C_6D_6) δ 159.8, 139.6, 131.4, 124.0, 121.4, 81.2, 64.0, 32.2, 26.6, 25.4, 23.1, 20.9, 17.3. **HRMS** (EI): m/z calcd for $C_{13}H_{22}O$ $[M\text{-}CH_3]^+$: 179.1430; found 179.1431.

1-nitro-4-((prop-1-en-2-yloxy)methyl)benzene (3ah)

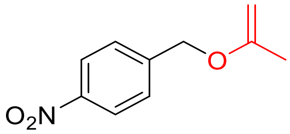

Following the general procedure A (2 mol% LPTS, 24 h), the titled compound was isolated by column chromatography (100:1 PE:Et_3N) as a white solid in 60% yield. **^{1}H NMR** (400 MHz, C_6D_6) δ 7.81 (d, *J* = 8.7 Hz, 2H), 7.15 (s, 1H), 6.79 (d, *J* = 8.7 Hz, 2H), 4.25 (s, 2H), 3.91 (s, 1H), 3.76 (s, 1H), 1.76 (s, 3H). **^{13}C NMR** (100 MHz, C_6D_6) δ 159.0, 147.3, 143.9, 127.1, 123.2, 82.6, 67.6, 20.5. **HRMS** (ESI): m/z calcd for $C_{10}H_{11}NO_3$ $[M+H]^+$: 194.0812; found 194.0814.

(3*r*,5*r*,7*r*)-1-((prop-1-en-2-yloxy)methyl)adamantane (3ai)

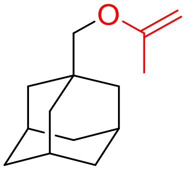

Following the general procedure A (2 mol% LPTS, 24 h), the titled compound was isolated by column chromatography (100:1 PE:Et_3N) as a white solid in 58% yield. **^{1}H NMR** (400 MHz, C_6D_6) δ 3.92 (d, *J* = 4.0 Hz, 2H), 3.22 (s, 2H), 1.91 (s, 3H), 1.82 (s, 3H), 1.61 – 1.70 (m, 6H), 1.58 (d, *J* = 2.3 Hz, 6H). **^{13}C NMR** (100 MHz, C_6D_6) δ 160.1, 80.9, 77.5, 39.4, 37.1, 33.2, 28.3, 20.7. **HRMS** (ESI): m/z calcd for $C_{14}H_{22}O$ $[M+H]^+$: 207.1743; found 207.1745.

(1*r*,4*r*)-1,4-bis((prop-1-en-2-yloxy)methyl)cyclohexane (3aj)

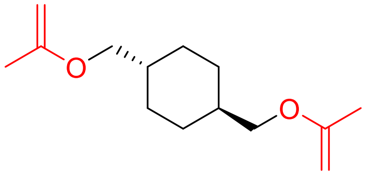

Following the general procedure A (0.5 mol% LPTS, 12 h), the titled compound was isolated by column chromatography (100:1 PE:Et_3N) as a white solid in 58% yield. The spectra for **3aj** matched previously reported data.[21] **^{1}H NMR** (400 MHz, C_6D_6) δ 3.91 (d, *J* = 9.1 Hz, 4H), 3.37 (d, *J* = 6.3 Hz, 4H), 1.81 (s, 6H), 1.76 (d, *J* = 6.8 Hz, 4H), 1.48 – 1.59 (m, 2H), 0.84 – 0.95 (m, 4H). **^{13}C NMR** (100 MHz, C_6D_6) δ 159.9, 81.0, 72.5, 37.5, 29.2, 20.8. **HRMS** (EI): m/z calcd for $C_{14}H_{24}O_2$ $[M-CH_3]^+$: 209.1536; found 209.1535.

methyl (*S*)-3-(prop-1-en-2-yloxy)butanoate (3ak)

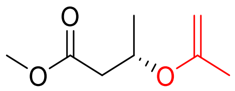

Following the general procedure A (2 mol% LPTS, 24 h), the titled compound was isolated by column chromatography (100:1:1 PE:EtOAc:Et_3N) as a colorless oil in 85% yield. **^{1}H NMR** (400 MHz, C_6D_6) δ 4.54 (m, 1H), 3.94 (s, 2H), 3.29 (s, 3H), 2.66 (m, 1H), 2.19 (m, 1H), 1.71 (s, 3H), 1.13 (d, *J* = 6.1 Hz, 3H). **^{13}C NMR** (100 MHz, C_6D_6) δ 170.5, 157.6, 82.2, 68.7, 50.7, 40.6, 21.1, 19.0. **HRMS** (EI): m/z calcd for $C_8H_{14}O_3$ $[M+H]^+$: 159.1016; found 159.1014.

(1*r*,3*r*,5*r*,7*r*)-2-(prop-1-en-2-yloxy)adamantane (3al)

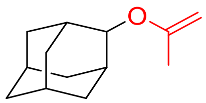

Following the general procedure A (0.5 mol% LPTS, 12 h), the titled compound was isolated by column chromatography (100:1 PE:Et_3N) as a colorless oil in 90% yield. **^{1}H NMR** (400 MHz, C_6D_6) δ 4.14 (s, 1H), 4.00 (s, 1H), 3.92 (s, 1H), 2.22 (d, *J* = 12.4 Hz, 2H), 2.16 (s, 2H), 1.85 (s, 3H), 1.73 (d, *J* = 13.9 Hz, 2H), 1.68 (s, 2H), 1.62 (s, 2H), 1.53 (d, *J* = 11.7 Hz, 2H), 1.46 (d, *J* = 11.5 Hz, 2H). **^{13}C NMR** (100 MHz, C_6D_6) δ 157.6, 81.8, 78.1, 37.4, 36.2, 31.7, 31.2, 27.4, 21.4. **HRMS** (EI): m/z calcd for $C_{13}H_{20}O$ $[M+H]^+$: 193.1587; found 193.1588.

(1-(prop-1-en-2-yloxy)ethyl)benzene (3am)

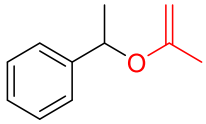

Following the general procedure A (2 mol% LPTS, 24 h), the titled compound was isolated by column chromatography (100:1 PE:Et_3N) as a colorless oil in 80% yield. **^{1}H NMR** (400 MHz, C_6D_6) δ 7.16 (d, *J* = 15.1 Hz, 5H), 4.91 (q, *J* = 6.4 Hz, 1H), 3.86 (d, *J* = 30.8 Hz, 2H), 1.81 (s, 3H), 1.39 (d, *J* = 6.5 Hz, 3H). **^{13}C NMR** (100 MHz, C_6D_6) δ 157.8, 143.8, 128.3, 127.0, 125.3, 84.0, 74.7, 23.9, 21.2. **HRMS** (EI): m/z calcd for $C_{11}H_{14}O$ $[M-CH_3]^+$: 147.0804; found 147.0803.

(3*S*,8*R*,9*S*,10*R*,13*S*,14*S*)-10,13-dimethyl-3-(prop-1-en-2-yloxy)-1,2,3,4,7,8,9,10,11,12,13,14,15,16-tetradecahydro-17*H*-cyclopenta[*a*]phenanthren-17-one (3an)

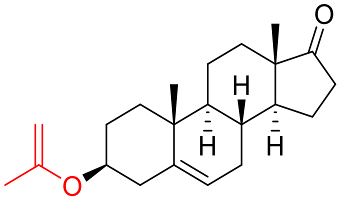

Following the general procedure A (0.5 mol% LPTS, 12 h), the titled compound was isolated by column chromatography (100:1 PE:Et_3N) as a white solid in 89% yield. **^{1}H NMR** (400 MHz, C_6D_6) δ 5.26– 5.30 (m, 1H), 4.02 (d, *J* = 3.5 Hz, 2H), 3.90 – 3.98 (m, 1H), 2.66 (m, 1H), 2.41 (m, 1H), 2.12 (m, 1H), 1.98 – 2.06 (m, 1H), 1.87 – 1.94 (m, 1H), 1.86 (s, 3H), 1.71 – 1.82(m, 2H), 1.51 – 1.62 (m, 2H), 1.13 – 1.45(m, 6H), 1.02 (m, 1H), 0.87 (m, 2H), 0.81 (s, 3H), 0.74 (m, 1H), 0.58 (s, 3H). **^{13}C NMR** (100 MHz, C_6D_6) δ 217.4, 157.9, 140.6, 121.1, 81.6, 75.5, 51.4, 50.2, 46.9, 38.4, 37.0, 36.8, 35.3, 31.7, 31.2, 30.7, 27.8, 21.5, 20.2, 19.0, 13.1. **HRMS** (ESI): m/z calcd for $C_{22}H_{32}O_2$ $[M+NH_4]^+$: 346.2741; found 346.2736.

EST-3-TBS-17-IPPE (3ao)

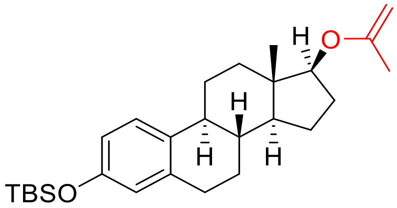

Substrate EST-3-TBS was synthesized as previously reported.[25] Following the general procedure A (0.5 mol% LPTS, 12 h), the titled compound was isolated by column chromatography (100:1:1 PE:EtOAc:Et_3N) as a white solid in 87% yield. **^{1}H NMR** (400 MHz, C_6D_6) δ 7.13 (s, 1H), 6.82 (d, *J* = 10.0 Hz, 2H), 4.00 (d, *J* = 4.5 Hz, 2H), 3.86 (t, *J* = 8.0 Hz, 1H), 2.74 (m, 2H), 1.99 – 2.15 (m, 4H), 1.83 (s, 3H), 1.63 – 1.71 (m, 1H), 1.27 – 1.51 (m, 5H), 1.17 (m, 3H), 1.05 (s, 9H), 0.85 (s, 3H), 0.19 (s, 6H). **^{13}C NMR** (100 MHz, C_6D_6) δ 159.2, 153.6, 137.7, 133.4, 126.5, 120.2, 117.5, 85.2, 82.2, 49.5, 44.0, 43.3, 38.4, 37.6, 29.7, 28.0, 27.3, 26.3, 25.6, 23.4, 21.1, 18.1, 12.0, -4.6. **HRMS** (ESI): m/z calcd for $C_{27}H_{42}O_2Si$ $[M+H]^+$: 427.3027; found 427.3027.

(1*R*,3a*S*,7a*R*,*E*)-1-((2*R*,5*R*,*E*)-5,6-dimethylhept-3-en-2-yl)-7a-methyl-4-((*Z*)-2-((*S*)-2-methylene-5-(prop-1-en-2-yloxy)cyclohexylidene)ethylidene)octahydro-1*H*-indene (3ap)

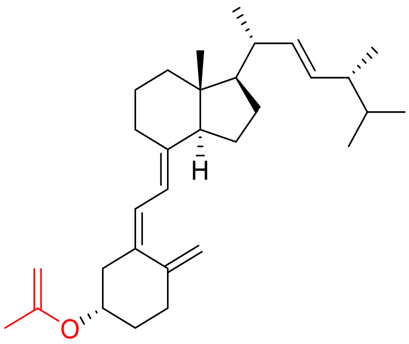

Following the general procedure A (0.5 mol% LPTS, 12 h), the titled compound was isolated by column chromatography (100:1 PE:Et_3N) as a faint yellow oil in 94% yield. **^{1}H NMR** (400 MHz, C_6D_6) δ 6.42 (s, 2H), 5.20 – 5.27 (m, 2H), 5.09 (s, 2H), 4.13 (dt, *J* = 11.8, 3.8 Hz, 1H), 3.98 (s, 1H), 3.91 (s, 1H), 2.83 (d, *J* = 13.2 Hz, 1H), 2.71 (d, *J* = 13.3 Hz, 1H), 2.31 – 2.60 (m, 2H), 1.98 – 2.07 (m, 2H), 1.86– 1.97 (m, 4H), 1.77 (s, 3H), 1.43 – 1.70 (m, 7H), 1.09 – 1.38 (m, 4H), 1.05 (d, *J* = 6.6 Hz, 3H), 0.99 (d, *J* = 6.8 Hz, 3H), 0.90 (dd, *J* = 6.7, 2.8 Hz, 6H), 0.59 (s, 3H). **^{13}C NMR** (100 MHz, C_6D_6) δ 157.8, 145.5, 141.5, 135.8, 135.6, 131.9, 122.3, 118.2, 112.4, 81.9, 73.1, 56.4, 56.3, 45.6, 43.0, 42.1, 40.5, 40.4, 33.1, 32.3, 31.9, 29.0, 27.9, 23.6, 22.3, 21.4, 21.1, 19.9, 19.6, 17.6, 12.1. **HRMS** (ESI): m/z calcd for $C_{31}H_{48}O$ $[M+H]^+$: 437.3778; found 437.3780.

(1*r*,3*r*,5*r*,7*r*)-2-methyl-2-(prop-1-en-2-yloxy)adamantane (3aq)

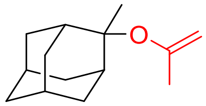

Following the general procedure A (2 mol% LPTS, 24 h), the titled compound was isolated by column chromatography (100:1 PE: Et_3N) as a colorless oil in 60% yield. **^{1}H NMR** (400 MHz, C_6D_6) δ 4.06 (s, 1H), 3.99 (s, 1H), 2.32 (d, *J* = 12.2 Hz, 2H), 2.15 (s, 2H), 1.79 (s, 3H), 1.77 (s, 1H), 1.63 (q, *J* = 18.4, 16.0 Hz, 7H), 1.50 (s, 1H), 1.46 (s, 4H). **^{13}C NMR** (100 MHz, C_6D_6) δ 154.6, 86.2, 81.9, 38.1, 36.7, 34.5, 33.0, 27.6, 27.0, 23.0, 21.3. **HRMS** (ESI): m/z calcd for $C_{14}H_{22}O$ $[M+H]^+$: 207.1743; found 207.1744.

(8*R*,9*S*,10*R*,13*S*,14*S*,17*S*)-10,13-dimethyl-17-((1-phenylvinyl)oxy)-1,2,6,7,8,9,10,11,12,13,14,15,16,17-tetradecahydro-3*H*-cyclopenta[*a*]phenanthren-3-one (5a)

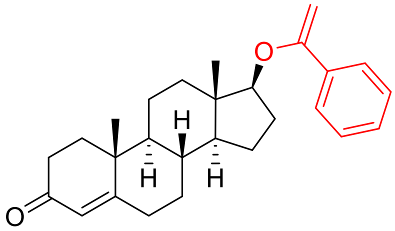

Following the general procedure B, with the modification of using (1-methoxyvinyl)benzene, the titled compound was isolated by column chromatography (100:1 PE: Et_3N) as a white solid in 72%

yield. **^{1}H NMR** (400 MHz, C_6D_6) δ 7.78 (d, *J* = 7.2 Hz, 2H), 7.20 (s, 2H), 7.12 (d, *J* = 7.4 Hz, 1H), 5.83 (s, 1H), 4.78 (d, *J* = 2.4 Hz, 1H), 4.26 (d, *J* = 2.4 Hz, 1H), 3.96 – 3.90 (m, 1H), 2.31 – 2.05 (m, 3H), 1.97 – 1.84 (m, 3H), 1.58 – 1.37 (m, 4H), 1.25 – 1.06 (m, 5H), 0.91 (s, 4H), 0.71 (s, 3H), 0.67 – 0.48 (m, 3H). **^{13}C NMR** (100 MHz, C_6D_6) δ 196.7, 168.1, 159.5, 137.4, 128.3, 125.5, 124.2, 85.7, 83.4, 53.6, 49.9, 43.2, 38.1, 37.2, 35.6, 35.0, 33.9, 32.2, 31.4, 27.9, 23.6, 20.4, 16.7, 12.2. **HRMS** (ESI): m/z calcd for $C_{27}H_{34}O_2$ $[M+H]^+$: 391.2632; found 391.2634.

(8*R*,9*S*,10*R*,13*S*,14*S*,17*S*)-17-((1-(4-methoxyphenyl)vinyl)oxy)-10,13-dimethyl-1,2,6,7,8,9,10,11,12,13,14,15,16,17-tetradecahydro-3*H*-cyclopenta[*a*]phenanthren-3-one (5b)

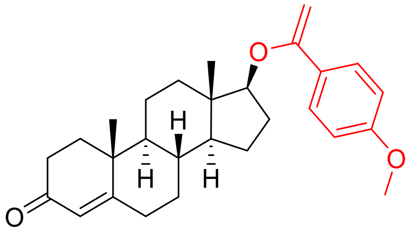

Following the general procedure B, with the modification of using 1-methoxy-4-(1-methoxyvinyl)benzene, the titled compound was isolated by column chromatography (100:1 PE:Et_3N) as a white solid in 65% yield. **^{1}H NMR** (400 MHz, $CDCl_3$) δ 7.56 (d, *J* = 8.8 Hz, 2H), 6.86 (d, *J* = 8.8 Hz, 2H), 5.74 (s, 1H), 4.55 (s, 1H), 4.11 (s, 1H), 4.04 (t, *J* = 8.0 Hz, 1H), 3.81 (s, 3H), 2.43 – 2.26 (m, 5H), 2.04 (dd, *J* = 8.9, 3.1 Hz, 2H), 1.94 – 1.86 (m, 2H), 1.76 – 1.56 (m, 6H), 1.50 – 1.38 (m, 2H), 1.21 (m, 4H), 1.12 – 1.07 (m, 1H), 0.99 (s, 3H). **^{13}C NMR** (100 MHz, $CDCl_3$) δ 199.5, 171.2, 159.9, 159.0, 129.8, 126.7, 124.0, 113.5, 85.7, 81.9, 55.3, 54.0, 50.4, 43.4, 38.7, 37.4, 35.8, 35.5, 34.0, 32.9, 31.7, 28.0, 24.0, 20.7, 17.5, 12.5. **HRMS** (ESI): m/z calcd for $C_{28}H_{36}O_3$ $[M+H]^+$: 421.2737; found 421.2738.

(8*R*,9*S*,10*R*,13*S*,14*S*,17*S*)-10,13-dimethyl-17-((1-(naphthalen-2-yl)vinyl)oxy)-1,2,6,7,8,9,10,11,12,13,14,15,16,17-tetradecahydro-3*H*-cyclopenta[*a*]phenanthren-3-one (5c)

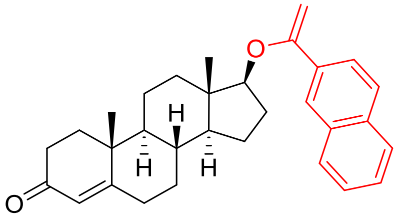

Following the general procedure B, with the modification of using 2-(1-methoxyvinyl)naphthalene, the titled compound was isolated by column chromatography (100:1 PE:Et_3N) as a white solid in 73% yield. **^{1}H NMR** (400 MHz, $CDCl_3$) δ 8.09 (s, 1H), 7.86 – 7.77 (m, 3H), 7.73 – 7.68 (m, 1H), 7.49 – 7.44 (m, 2H), 5.75 (s, 1H), 4.82 (d, *J* = 2.6 Hz, 1H), 4.31 (d, *J* = 2.5 Hz, 1H), 4.11 (t, *J* = 8.0 Hz, 1H), 2.44 – 2.26 (m, 5H), 2.07 – 1.85 (m, 4H), 1.80 – 1.56 (m, 6H), 1.47 (m, 2H), 1.22 (s, 4H), 1.14 – 1.08 (m, 1H), 1.06 (s, 3H). **^{13}C NMR** (100 MHz, $CDCl_3$) δ 199.6, 171.1, 159.2, 134.3, 133.4, 133.2, 128.5, 127.7, 127.6, 126.1, 124.4, 124.0, 123.6, 85.9, 84.2, 54.0, 50.4, 43.5, 38.7, 37.5, 35.8, 35.5, 34.0, 32.9, 31.7, 28.0, 24.0, 20.8, 17.5, 12.6. **HRMS** (ESI): m/z calcd for $C_{31}H_{36}O_2$ $[M+H]^+$: 441.2788; found 441.2789.

(8*R*,9*S*,10*R*,13*S*,14*S*,17*S*)-10,13-dimethyl-17-((1-(*p*-tolyl)vinyl)oxy)-1,2,6,7,8,9,10,11,12,13,14,15,16,17-tetradecahydro-3*H*-cyclopenta[*a*]phenanthren-3-one (5d)

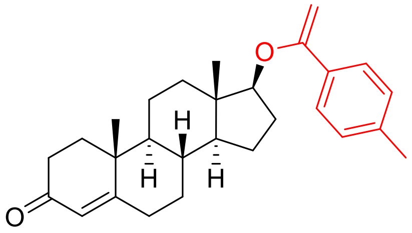

Following the general procedure B, with the modification of using 1-(1-methoxyvinyl)-4-methylbenzene, the titled compound was isolated by column chromatography (100:1 PE:Et_3N) as a white solid in 85% yield. **^{1}H NMR** (400 MHz, $CDCl_3$) δ 7.51 (d, *J* = 8.2 Hz, 2H), 7.13 (d, *J* = 8.0 Hz, 2H), 5.74 (s, 1H), 4.62 (d, *J* = 2.4 Hz, 1H), 4.14 (d, *J* = 2.4 Hz, 1H), 4.08 – 3.98 (m, 1H), 2.48 – 2.23 (m, 8H), 2.08 – 1.96 (m, 2H), 1.92 – 1.84 (m, 1H), 1.77 – 1.55 (m, 5H), 1.54 – 1.35 (m, 2H), 1.21 (s, 4H), 1.12 – 1.05 (m, 2H), 0.99 (s, 3H), 0.95 (m, 1H). **^{13}C NMR** (100 MHz, $CDCl_3$) δ 199.5, 171.2, 159.3, 138.2, 134.3, 128.8, 125.4, 124.0, 85.7, 82.7, 54.0, 50.4, 43.4, 38.7, 37.4, 35.8, 35.5, 34.0, 32.9, 31.7, 28.0, 24.0, 21.2, 20.7, 17.5, 12.5. **HRMS** (ESI): m/z calcd for $C_{28}H_{36}O_2$ $[M+H]^+$: 405.2788; found 405.2791.

(8*R*,9*S*,10*R*,13*S*,14*S*,17*S*)-17-(cyclohex-1-en-1-yloxy)-10,13-dimethyl-1,2,6,7,8,9,10,11,12,13,14,15,16,17-tetradecahydro-3*H*-cyclopenta[*a*]phenanthren-3-one (5e)

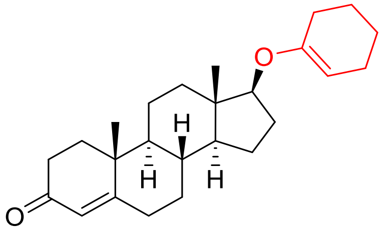

Following the general procedure B, with the modification of using 1-methoxycyclohex-1-ene, the titled compound was isolated by column chromatography (100:1 PE:Et_3N) as a white solid in 75% yield. **^{1}H NMR** (400 MHz, C_6D_6) δ 5.83 (s, 1H), 4.70 (t, *J* = 3.7 Hz, 1H), 3.83 (t, *J* = 8.1 Hz, 1H), 2.28 – 2.05 (m, 7H), 1.95 – 1.82 (m, 3H), 1.60 – 1.38 (m, 8H), 1.27 – 1.06 (m, 5H), 1.00 – 0.92 (m, 1H), 0.89 (s, 3H), 0.70 (s, 3H), 0.69 – 0.60 (m, 2H), 0.52 (m, 1H). **^{13}C NMR** (100 MHz, C_6D_6) δ 197.1, 168.6, 154.3, 124.4, 95.0, 84.3, 53.8, 50.3, 43.1, 38.3, 37.5, 35.8, 35.2, 34.2, 32.5, 31.7, 28.6, 28.5, 24.0, 23.9, 23.3, 23.1, 20.7, 16.9, 12.3. **HRMS** (ESI): m/z calcd for $C_{25}H_{36}O_2$ $[M+H]^+$: 369.2788; found 369.2792.

(8*R*,9*S*,10*R*,13*S*,14*S*,17*S*)-17-((4,4-dimethylcyclohex-1-en-1-yl)oxy)-10,13-dimethyl-1,2,6,7,8,9,10,11,12,13,14,15,16,17-tetradecahydro-3*H*-cyclopenta[*a*]phenanthren-3-one (5f)

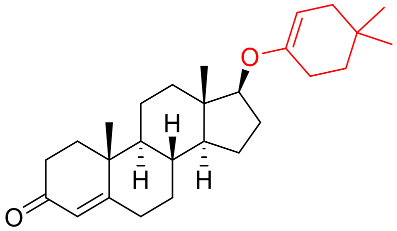

Following the general procedure B, with the modification of using 1-methoxy-4,4-dimethylcyclohex-1-ene, the titled compound was isolated by column chromatography (100:1 PE:Et_3N) as a white solid in 67% yield. **^{1}H NMR** (400 MHz, $CDCl_3$) δ 5.72 (s, 1H), 4.51 (d, *J* = 3.6 Hz, 1H), 3.84 (t, *J* = 8.1 Hz, 1H), 2.44 – 2.23 (m, 4H), 2.18 – 2.07 (m, 1H), 2.03 (m, 3H), 1.92 – 1.78 (m, 4H), 1.74 – 1.53 (m, 4H), 1.51 – 1.25 (m, 6H), 1.19 (s, 3H), 1.12 (m, 1H), 0.90 (s, 8H), 0.82 (s, 3H). **^{13}C NMR** (100 MHz, $CDCl_3$) δ 199.5, 171.3, 152.9, 123.9, 94.6, 84.3, 54.0, 50.5, 43.0, 38.7, 37.7, 37.4, 35.8, 35.7, 35.5, 34.0, 32.9, 31.7, 28.9, 28.3, 28.1, 28.0, 25.5, 23.8, 20.7, 17.5, 12.0. **HRMS** (ESI): m/z calcd for $C_{27}H_{40}O_2$ $[M+H]^+$: 397.3101; found 397.3101.

2,3-dimethoxy-5-methyl-6-(10-((1-phenylvinyl)oxy)decyl)cyclohexa-2,5-diene-1,4-dione (5g)

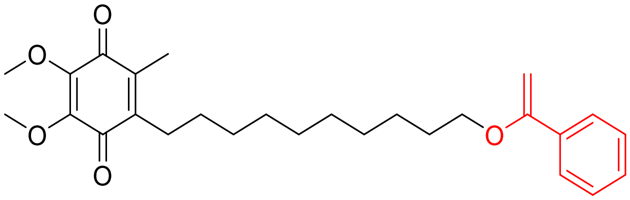

Following the general procedure B, with the modification of using (1-methoxyvinyl)benzene, the titled compound was isolated by column chromatography (100:30:1 DCM:PE:Et_3N) as an orange oil in 68% yield. **^{1}H NMR** (400 MHz, C_6D_6) δ 7.78 (d, *J* = 8.7 Hz, 2H), 7.18 (d, *J* = 7.1 Hz, 2H), 7.12 (d, *J* = 7.3 Hz, 1H), 4.72 (d, *J* = 2.5 Hz, 1H), 4.18 (d, *J* = 2.4 Hz, 1H), 3.66 (t, *J* = 6.3 Hz, 2H), 3.60 (s, 6H), 2.34 – 2.27 (m, 2H), 1.77 (s, 3H), 1.68 – 1.62 (m, 2H), 1.35 (m, 4H), 1.24 (s, 10H). **^{13}C NMR** (100 MHz, C_6D_6) δ 184.0, 183.6, 160.3, 144.7, 142.4, 138.1, 137.0, 128.3, 128.1, 125.5, 81.9, 67.5, 60.3, 29.9, 29.6, 29.5, 29.4, 28.7, 26.3, 26.2, 11.4. **HRMS** (ESI): m/z calcd for $C_{27}H_{36}O_5$ $[M+H]^+$: 441.2636; found 441.2639.

(3*S*,8*R*,9*S*,10*R*,13*S*,14*S*)-10,13-dimethyl-3-((1-phenylvinyl)oxy)-1,2,3,4,7,8,9,10,11,12,13,14,15,16-tetradecahydro-17*H*-cyclopenta[*a*]phenanthren-17-one (5h)

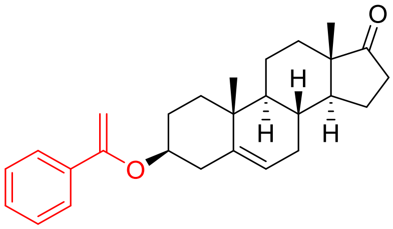

Following the general procedure B, with the modification of using (1-methoxyvinyl)benzene, the titled compound was isolated by column chromatography (100:1 DCM:Et_3N) as a white solid in 72% yield. **^{1}H NMR** (400 MHz, C_6D_6) δ 7.81 (d, J = 7.1 Hz, 2H), 7.21 (d, J = 7.1 Hz, 3H), 5.30 (d, J = 5.1 Hz, 1H), 4.81 (d, J = 2.5 Hz, 1H), 4.30 (d, J = 2.4 Hz, 1H), 4.06 (m, 1H), 2.69 (m, 1H), 2.53 – 2.41 (m, 1H), 2.14 (dd, J = 19.1, 8.4 Hz, 1H), 2.05 (m, 1H), 1.95 – 1.87 (m, 1H), 1.84 – 1.72 (m, 2H), 1.61 (m, 2H), 1.48 – 1.26 (m, 5H), 1.25 – 1.14 (m, 2H), 1.03 (m, 1H), 0.92 – 0.75 (m, 6H), 0.61 (s, 3H). **^{13}C NMR** (100 MHz, C_6D_6) δ 217.6, 158.4, 140.5, 137.4, 128.4, 125.7, 121.3, 83.1, 76.2, 51.3, 50.2, 47.0, 38.3, 37.0, 36.8, 35.3, 31.7, 31.2, 30.8, 27.8, 21.5, 20.2, 19.1, 13.1. **HRMS** (ESI): m/z calcd for $C_{27}H_{34}O_2$ $[M+H]^+$: 391.2632; found 391.2634.

_N_-(6-((1-phenylvinyl)oxy)hexyl)acrylamide (5i)

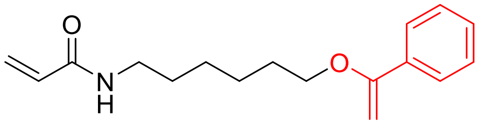

Following the general procedure B, with the modification of using (1-methoxyvinyl)benzene, the titled compound was isolated by column chromatography (100:1 DCM:Et_3N) as a white solid in 78% yield. **^{1}H NMR** (400 MHz, C_6D_6) δ 7.77 (d, J = 7.2 Hz, 2H), 7.19 (d, J = 7.1 Hz, 2H), 7.10 (t, J = 7.3 Hz, 1H), 6.38 (dd, J = 16.9, 1.8 Hz, 1H), 5.61 (dd, J = 16.9, 10.2 Hz, 1H), 5.23 (dd, J = 10.2, 1.8 Hz, 1H), 4.72 (d, J = 2.5 Hz, 1H), 4.51 (s, 1H), 4.17 (d, J = 2.5 Hz, 1H), 3.60 (t, J = 6.3 Hz, 2H), 3.07 (m, 2H), 1.57 – 1.50 (m, 2H), 1.27 – 1.15 (m, 4H), 1.05 (m, 2H). **^{13}C NMR** (100 MHz, C_6D_6) δ 164.4, 160.4, 137.2, 131.4, 128.6, 125.7, 125.5, 82.2, 67.6, 39.3, 29.8, 29.1, 26.7, 26.1. **HRMS** (ESI): m/z calcd for $C_{17}H_{23}NO_2$ $[M+H]^+$: 274.1802; found 274.1802.

2-(10-(cyclohex-1-en-1-yloxy)decyl)-5,6-dimethoxy-3-methylcyclohexa-2,5-diene-1,4-dione (5j)

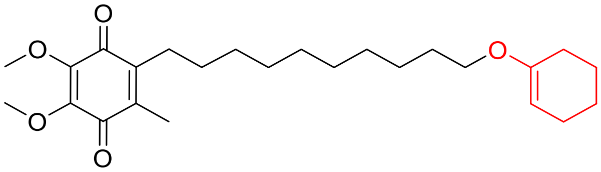

Following the general procedure B, with the modification of using 1-methoxycyclohex-1-ene, the titled compound was isolated by column chromatography (100:1 PE:Et_3N) as an orange oil in 78% yield. **^{1}H NMR** (400 MHz, C_6D_6) δ 4.62 (t, J = 3.4 Hz, 1H), 3.60 (d, J = 7.8 Hz, 8H), 2.34 – 2.27 (m, 2H), 2.20 (d, J = 6.2 Hz, 2H), 2.11 – 2.05 (m, 2H), 1.77 (s, 3H), 1.65 (m, 2H), 1.57 (dd, J = 11.1, 5.9 Hz, 2H), 1.48 (m, 2H), 1.41 – 1.29 (m, 4H), 1.23 (m, 10H). **^{13}C NMR** (100 MHz, C_6D_6) δ 184.4, 184.0, 155.2, 145.1, 142.8, 138.5, 93.5, 66.3, 60.6, 30.2, 30.0, 29.9, 29.8, 29.0, 28.5, 26.8, 26.6, 24.0, 23.4, 23.3, 11.7. **HRMS** (ESI): m/z calcd for $C_{25}H_{38}O_5$ $[M+H]^+$: 419.2792; found 419.2800.

(3*S*,8*R*,9*S*,10*R*,13*S*,14*S*)-3-(cyclohex-1-en-1-yloxy)-10,13-dimethyl-1,2,3,4,7,8,9,10,11,12,13,14,15,16-tetradecahydro-17*H*-cyclopenta[*a*]phenanthren-17-one (5k)

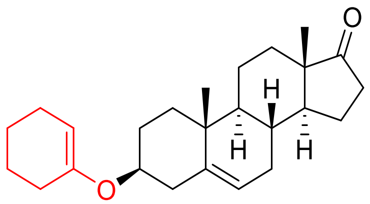

Following the general procedure B, with the modification of using 1-methoxycyclohex-1-ene, the titled compound was isolated by column chromatography (100:1 PE:Et_3N) as a white solid in 75% yield. **^{1}H NMR** (400 MHz, C_6D_6) δ 5.35 – 5.28 (m, 1H), 4.79 – 4.73 (m, 1H), 3.97 (m, 1H), 2.68 (d, J = 11.5 Hz, 1H), 2.45 (t, J = 12.3 Hz, 1H), 2.23 (t, J = 6.4 Hz, 2H), 2.17 – 2.00 (m, 4H), 1.94 – 1.87 (m, 1H), 1.83 – 1.71 (m, 2H), 1.68 – 1.55 (m, 4H), 1.53 – 1.31 (m, 6H), 1.27 – 1.16 (m, 2H), 1.10 – 0.99 (m, 1H), 0.97 – 0.78 (m, 6H), 0.60 (s, 3H). **^{13}C NMR** (100 MHz, C_6D_6) δ 217.7, 152.9, 140.9, 127.5, 121.0, 94.3, 74.4, 51.4, 50.3, 47.0, 38.9, 37.1, 36.9, 35.3, 31.7, 31.3, 30.7, 28.6, 28.3, 23.8, 23.1, 23.0, 21.5, 20.2, 19.1, 13.1. **HRMS** (ESI): m/z calcd for $C_{25}H_{36}O_2$ $[M+H]^+$: 369.2788; found 369.2790.

***N*-(6-(cyclohex-1-en-1-yloxy)hexyl)acrylamide (5l)**

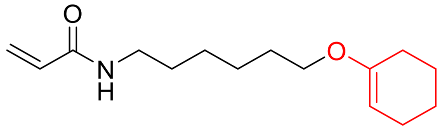

Following the general procedure B, with the modification of using 1-methoxycyclohex-1-ene, the titled compound was isolated by column chromatography (100:50:1 PE: EtOAc:Et_3N) as a white solid in 70% yield. **^{1}H NMR** (400 MHz, C_6D_6) δ 6.40 (d, J = 16.9 Hz, 1H), 5.96 (dd, J = 17.0, 10.2 Hz, 1H), 5.86 (s, 1H), 5.29 (d, J = 10.3 Hz, 1H), 4.60 (t, J = 4.0 Hz, 1H), 3.53 (t, J = 6.4 Hz, 2H), 3.18 (q, J = 6.7 Hz, 2H), 2.17 (d, J = 6.1 Hz, 2H), 2.10 – 2.04 (m, 2H), 1.58 – 1.53 (m, 4H), 1.50 – 1.45 (m, 2H), 1.28 (m, 4H), 1.16 – 1.09 (m, 2H). **^{13}C NMR** (100 MHz, C_6D_6) δ 164.8, 154.8, 131.5, 125.2, 93.2, 65.9, 39.3, 29.6, 29.3, 28.1, 26.7, 26.0, 23.7, 23.0. **HRMS** (ESI): m/z calcd for $C_{15}H_{25}NO_2$ $[M+H]^+$: 252.1958; found 252.1961.

8) Elucidating Mechanism of Organocatalytic Transisopropenylation

Monitoring the Reaction Process

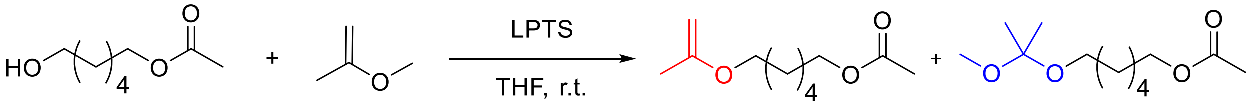

1ab **2a** **3ab** **3ab'**

To a Schlenk flask (10 mL) was added **1ab** (1 mmol) and LPTS (2 mol%). Then THF (2 mL) and **2a** (16 mmol) was added to the flask, and stirred at room temperature. At predetermined intervals, 0.2 mL solution was sampled and quenched with Et_3N. Solvent and excess of **2a** were removed under reduced pressure. The composition of residual mixture was determined by comparing the integral area of characteristic peaks (**3ab′** at 3.17 ppm, **3ab** at 1.79 ppm).

Organocatalytic Synthesis of 3a from 3a′

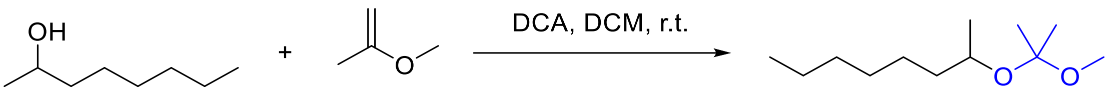

1a **2a** **3a'**

To a Schlenk flask (100 mL) was added **1a** (1.3 g, 10 mmol) and DCA (26 mg, 2 mol%). Then DCM (20 mL) and **2a** (1.9 mL, 20 mmol) was added to the flask, and stirred at room temperature. After the reaction was complete, 0.2 mL Et_3N was added to quench the reaction. Solvent and excess of **2a** were removed under reduced pressure. The resulting residue was purified by column chromatography (100:1 PE:Et_3N) to give the **3a′**. **^{1}H NMR** (400 MHz, $CDCl_3$) δ 3.82 (m, 1H), 3.23 (s, 3H), 1.58 – 1.38 (m, 2H), 1.35 (s, 6H), 1.28 (s, 8H), 1.13 (d, *J* = 6.2 Hz, 3H), 0.92 – 0.85 (m, 3H).

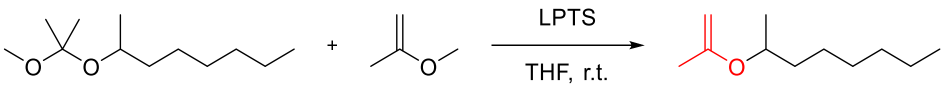

3a′ **2a** **3a**

To a Schlenk flask (10 mL) was added **3a′** (1 mmol) and LPTS (0.5 mol%). Then THF (2 mL) and **2a** (16 mmol) was added to the flask, and stirred at room temperature for 1 h. After the reaction was complete, 0.2 mL Et_3N was added to quench the reaction. Solvent and excess of **2a** were removed under reduced pressure. According to ^{1}H NMR, 95% of **3a'** was converted to **3a**. Moreover, methoxypropyl ketal of methanol was detected.

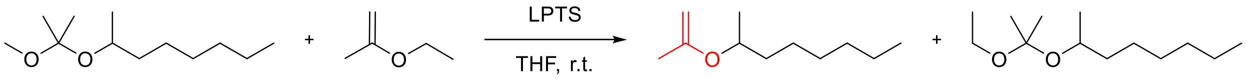

3a' **2b** **3a**

To a Schlenk flask (10 mL) was added **3a′** (1 mmol) and LPTS (0.5 mol%). Then THF (2 mL) and 2-ethoxypropene **2b** (16 mmol) was added to the flask, and stirred at room temperature for 1 h. After the reaction was complete, 0.2 mL Et_3N was added to quench the reaction. Solvent and excess of **2b** were removed under reduced pressure. According to ^{1}H NMR, **3a′** was converted to **3a** and a new ethoxyl ketal. The ratio of **3a** to the ethoxyl ketal was determined as 88:12.

The Influence of Stoichiometric Ratios on Yield of IPPEs

To a Schlenk flask was added **3a** (1 mmol) and LPTS (0.5 mol%). Then THF (2 mL) and **2a** was added to the flask, and stirred at room temperature. After the reaction was complete, 0.2 mL Et_3N was added to quench the reaction. Solvent and excess of **2a** were removed under reduced pressure. The composition of residual mixture was determined by comparing the integral area of characteristic peaks of **3a** and **3a′**. In fact, the data was also shown in Supplementary Table 3.

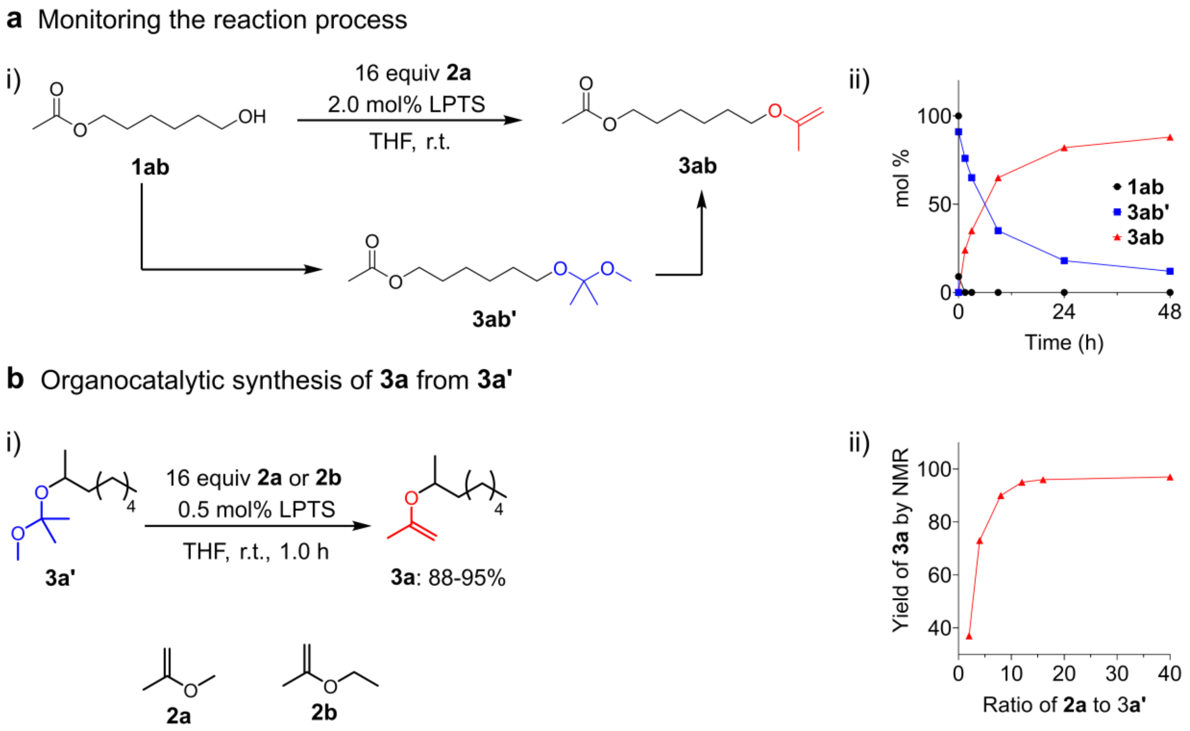

Supplementary Figure 17. Control experiments.

To elucidate the mechanism, we monitored some reactions of **1ab** and **2a** using ^{1}H NMR (Supplementary Figure 17a-i). Kinetic curves showed that 91% of **1ab** was converted to **3ab′** in 1 min, and ketal **3ab′** was then gradually transformed to **3ab** (Supplementary Figure 17a-ii).

To verify whether IPPE was truly converted from ketal, **3a′** was prepared and reacted with **2a** or 2-ethoxypropene (2-EPE, **2b**) under similar conditions (Supplementary Figure 17b-i). As a result, reaction of **3a′** with **2a** (16 equiv.) afforded **3a** with 95% yield, and methoxypropyl ketal of methanol was detected; whereas reaction of **3a′** with **2b** produced **3a** (16 equiv.) with 88% yield,

and ethoxypropyl ketal of methanol was detected. In reaction of **3a′** with **2a**, the yield of **3a** at equilibrium was strongly influenced by the stoichiometric ratio of **2a** to **3a′**(Supplementary Figure 17b-ii).

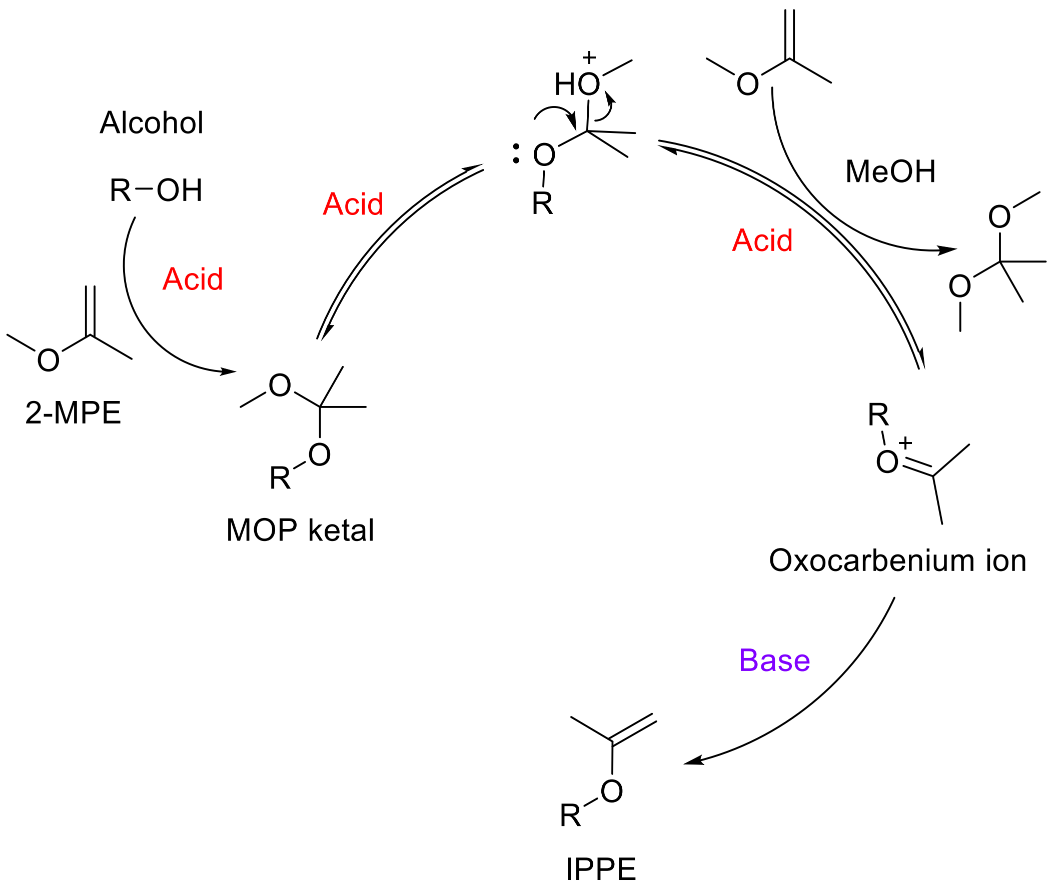

Supplementary Figure 18. The mechanism. Alcohol reacts with 2-MPE to form MOP ketal, and then the excess of 2-MPE drives the elimination of methanol from MOP ketal to produce the oxocarbenium ion, which finally rearranges into a new IPPE with the help of base.

9) Synthesis of Ketal-Linked Prodrugs

Synthesis of HSA-K-DEX

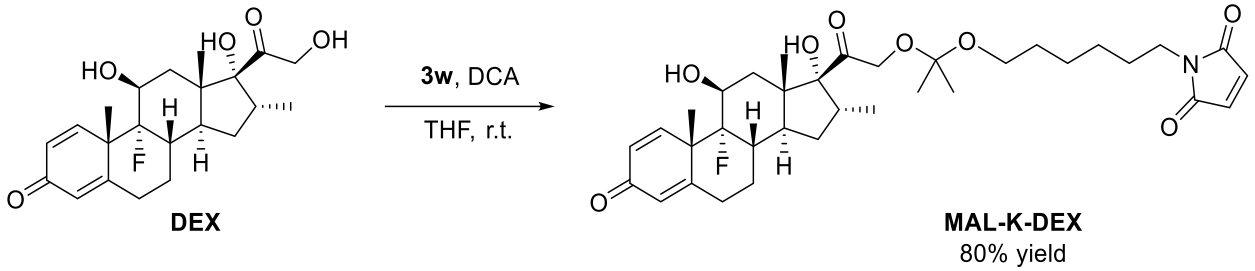

To a Schlenk flask was added dexamethasone (100 mg, 0.26 mmol) and **3w** (360 mg, 1.56 mmol). Then dry THF (2 mL) and DCA (6.6 mg, 0.05 mmol) was added to the flask, and stirred at room temperature for 6 h. After the reaction was completed (monitored by TLC), 0.1 mL Et_3N was added to quench the reaction. Solvent was removed under reduced pressure. The resulting residue was purified by column chromatography (33:66:1 PE:EtOAc:Et_3N) to afford MAL-K-DEX as a white solid (128 mg, 80% yield). **^{1}H NMR** (400 MHz, $CDCl_3$) δ 7.21 (d, J = 10.1 Hz, 1H), 6.69 (s, 2H), 6.33 (d, J = 10.6 Hz, 1H), 6.11 (s, 1H), 4.52 (d, J = 17.9 Hz, 1H), 4.38 (d, J = 11.7 Hz, 1H), 4.18 (d, J = 18.2 Hz, 1H), 3.51 (t, J = 7.1 Hz, 2H), 3.39 (t, J = 6.4 Hz, 2H), 3.08 (s, 1H), 2.92 (s, 1H), 2.61 (m, 1H), 2.38 (d, J = 13.6 Hz, 3H), 2.28 – 2.18 (m, 1H), 2.02 (s, 1H), 1.88 – 1.70 (m, 2H), 1.60 (s, 1H), 1.57 – 1.40 (m, 8H), 1.38 (s, 6H), 1.36 – 1.21 (m, 5H), 1.06 (s, 3H), 0.91 (d, J = 7.2 Hz, 3H). **^{13}C NMR** (100 MHz, $CDCl_3$) δ 208.6, 186.6, 171.2, 166.1, 152.0, 134.2, 123.0, 125.2, 100.8, 91.1, 72.3, 66.4, 61.1, 48.5, 48.2, 43.8, 37.8, 37.2, 36.1, 34.3, 34.1, 32.4, 31.1, 29.7, 28.4, 27.4, 26.5, 25.9, 25.0, 24.9, 23.1, 17.3, 14.9. **HRMS** (ESI): m/z calcd for $C_{35}H_{48}FNO_8$ $[M+Na]^+$: 652.3256; found 652.3257.

To prepare HSA-K-DEX, 5 mg HSA was dissolved in 2 mL PBS (6.7 mM, pH 8.0, containing 5% v/v DMSO), followed by addition of MAL-K-DEX (0.7 mg, 15 equiv.). The solution was gently shaken in an incubator at 25 °C for 2 h. The solution was concentrated in Millipore 10 kDa ultrafiltration tube and washed five times with 10 mL PBS buffer (6.7 mM, pH 8.0, containing 3% v/v DMSO), concentrating between each wash. The isolated HSA-K-DEX was resuspended in 1 mL solution, and the concentration of protein was determined as 4.8 mg/mL by using UV spectroscopy. The molecular weight of conjugate was measured by MALDI-TOF. In addition, 200 μL HSA-K-DEX solution was diluted to 800 μL with 0.2 M hydrochloric acid until complete hydrolyzation of HSA-K-DEX. The hydrolyzed DEX concentration was analyzed using HPLC. The degree of derivatization was approximately 0.7.

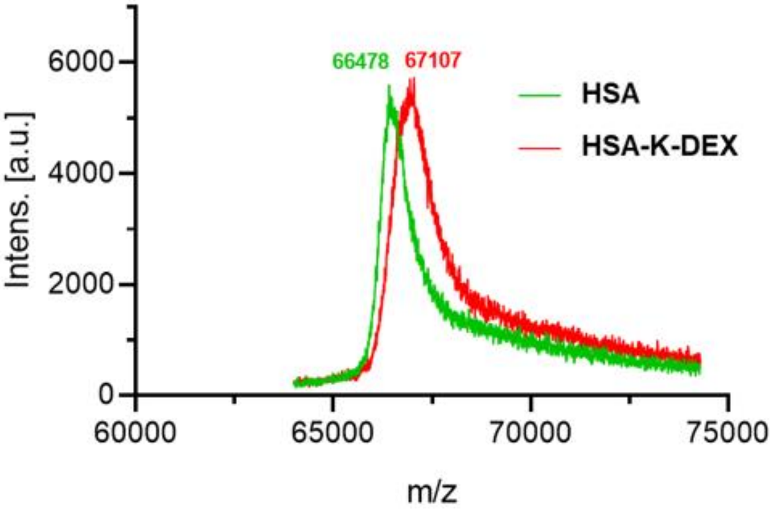

Supplementary Figure 19. MALDI-TOF spectra of HSA and HSA-K-DEX.

Synthesis of PEG-K-DEX

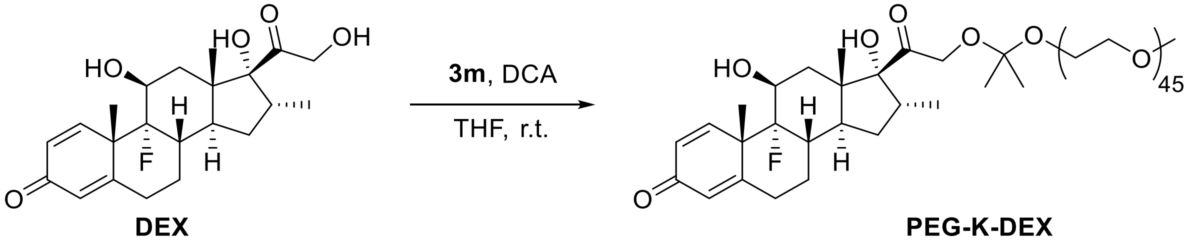

To a Schlenk flask (25 mL) was added dexamethasone (0.1 g, 0.26 mmol) and **3m** (3.1 g, 1.56 mmol, M_n = 2,000 g/mol). The flask was evacuated and refilled with N_2. Then dry THF (4 mL) and DCA (20 mg, 0.16 mmol) was added to the flask, and stirred at room temperature for 6 h. After the reaction was completed (monitored by TLC), 0.2 mL Et_3N was added to quench the reaction. Solvent was removed under reduced pressure. The resulting residue was purified by reversed-phase column chromatography (60:40:0.1 H_2O:ACN:Et_3N) afford PEG-K-DEX as a white solid (370 mg, 60% yield). Spectra of PEG-K-DEX matched previously reported data.[26] **^{1}H NMR** (400 MHz, $CDCl_3$) δ 7.22 (d, *J* = 10.1 Hz, 1H), 6.35 (d, *J* = 10.1 Hz, 1H), 6.10 (s, 1H), 4.60 (d, *J* = 18.0 Hz, 1H), 4.34 (d, *J* = 11.8 Hz, 1H), 4.24 (d, *J* = 18.0 Hz, 1H), 3.85 –3.41 (m, 198H), 3.38 (s, 3H), 3.15 – 3.05 (m, 1H), 2.67 – 2.54 (m, 1H), 2.42 – 2.27 (m, 3H), 2.27 – 2.12 (m, 1H), 1.63 – 1.57 (m, 1H), 1.54 (s, 3H), 1.41 (s, 6H), 1.36 – 1.32 (m, 1H), 1.28 – 1.20 (m, 1H), 1.04 (s, 3H), 0.90 (d, *J* = 7.3 Hz, 3H). **^{13}C NMR** (100 MHz, $CDCl_3$) δ 208.2, 186.5, 166.2, 152.2, 129.8, 125.1, 100.7, 91.1, 72.0, 70.6, 66.2, 60.6, 60.2, 59.0, 48.3, 43.9, 36.9, 35.7, 34.4, 34.2, 32.4, 31.1, 27.4, 24.8, 23.1, 17.1, 14.9.

Synthesis of LA-K-CAPME

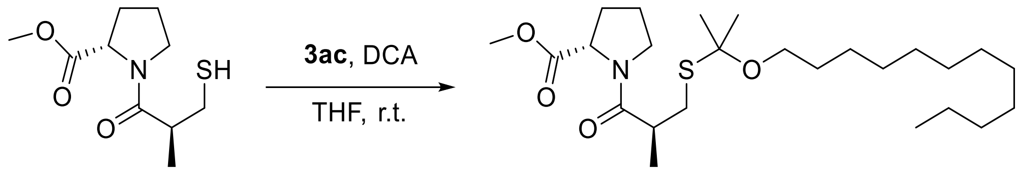

CAPME **LA-K-CAPME**
77% yield

Captopril methyl ester (CAPME) was prepared according to the reported procedure.[27] To a Schlenk flask was added CAPME (150 mg, 0.65 mmol) and IPPE **3ac** (881 mg, 3.9 mmol). Then dry THF (5 mL) and DCA (17 mg, 0.13 mmol) was added to the flask, and stirred at room temperature for 6 h. After the reaction was completed (monitored by TLC), 0.2 mL Et_3N was added to quench the reaction. Solvent was removed under reduced pressure. The resulting residue was purified by column chromatography (100:20:1 PE:EtOAc:Et_3N) to afford LA-K-CAPME as a white solid (230 mg, 77% yield). **^{1}H NMR** (400 MHz, $CDCl_3$) δ 4.53 (dd, *J* = 8.6, 3.9 Hz, 1H), 3.71 (s, 3H), 3.65 (t, *J* = 6.6 Hz, 2H), 3.45 (t, *J* = 7.4 Hz, 2H), 2.87 (m, 1H), 2.75 (m, 1H), 2.52 (dd, *J* = 12.1, 6.8 Hz, 1H), 2.20 (m, 1H), 2.10 – 1.96 (m, 3H), 1.50 (s, 6H), 1.26 (m, 23H), 0.88 (t, *J* = 6.7 Hz, 3H). **^{13}C NMR** (100 MHz, $CDCl_3$) δ 174.0, 172.9, 85.5, 62.7, 58.6, 52.1, 46.9, 38.8, 31.9, 31.1, 30.0, 29.7, 29.6, 29.6, 29.4, 29.1, 28.7, 28.6, 26.4, 24.8, 22.7, 17.3, 14.1. **HRMS** (ESI): m/z calcd for $C_{25}H_{47}NO_4S$ $[M+Na]^+$: 480.3118; found 480.3120.

Synthesis of LA-K-BUF

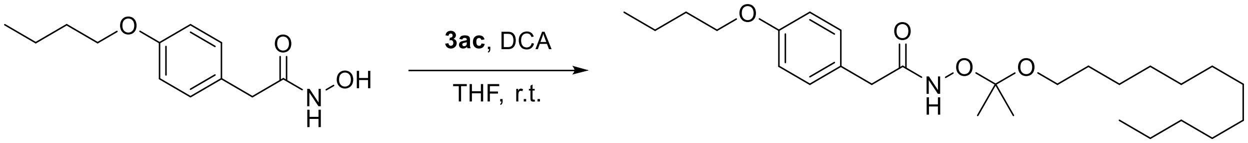

BUF **LA-K-BUF**
85% yield

To a Schlenk flask (25 mL) was added bufexamac (200 mg, 0.9 mmol) and IPPE **3ac** (1.2 g, 5.4 mmol). Then dry THF (6 mL) and DCA (23 mg, 0.18 mmol) was added to the flask, and stirred at room temperature for 6 h. After the reaction was completed (monitored by TLC), 0.2 mL Et_3N was added to quench the reaction. Solvent was removed under reduced pressure. The resulting residue was purified by column chromatography (100:20:1 PE:EtOAc:Et_3N) to afford LA-K-BUF as a white solid (340 mg, 85% yield). **^{1}H NMR** (400 MHz, $CDCl_3$) δ 7.69 (s, 1H), 7.18 (d, *J* = 8.5 Hz, 2H), 6.86 (d, *J* = 8.3 Hz, 2H), 3.94 (t, *J* = 6.5 Hz, 2H), 3.45 (d, *J* = 39.5 Hz, 4H), 1.76 (m, 2H), 1.53 – 1.45 (m, 2H), 1.38 (s, 6H), 1.27 (m, 20H), 0.97 (t, *J* = 7.4 Hz, 3H), 0.88 (t, *J* = 6.7 Hz, 3H). **^{13}C NMR** (100 MHz, $CDCl_3$) δ 168.3, 158.6, 130.3, 125.6, 115.1, 106.2, 67.8, 62.1, 40.7, 32.0, 31.4, 29.8, 29.7, 29.7, 29.6, 29.5, 29.4, 26.2, 23.1, 22.7, 19.3, 14.1, 13.9. **HRMS** (ESI): m/z calcd for $C_{27}H_{47}NO_4$ $[M+Na]^+$: 472.3397; found 472.3398.

Synthesis of PTX-7-K-EG_3

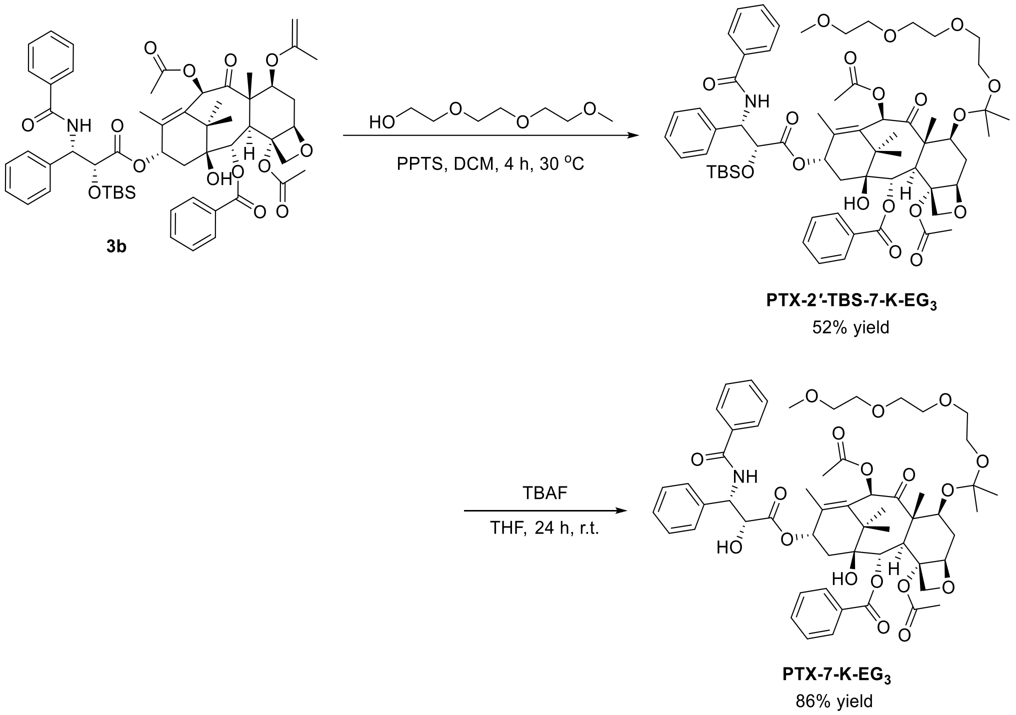

To a solution of PTX-2′-TBS-7-IPPE **3b** (75.6 mg, 75.0 μmol) and triethylene glycol monomethyl ether (8.2 mg, 50.0 μmol) in anhydrous DCM (2 mL) was added PPTS (2.5 μmol, 64 μL of 10.0 mg/mL in DCM) under N_2 atmosphere, and the resulting mixture was stirred for 4 h at 30 °C. The reaction mixture was quenched with Et_3N (1 mL) and concentrated in vacuo. The residue was chromatographed on silica gel column with EtOAc/PE (1:10 to 1:3, v/v) containing 0.5% Et_3N as an eluent to offer PTX-2′-TBS-7-K-EG_3 as white solid (45.6 mg, 52.0%).

To a solution of PTX-2′-TBS-7-K-EG_3 (37.5 mg, 32.0 μmol) in anhydrous THF (0.5 mL) was added TBAF (64 μL of 1.0 M THF solution, 64.0 μmol) under N_2 atmosphere, and the resulting mixture was stirred for 24 h at room temperature. The reaction mixture was quenched with saturated $NaHCO_3$ and diluted with EtOAc, then washed with water. The combined organic layer was washed with brine, dried over anhydrous $MgSO_4$ and concentrated in vacuo. The residue was chromatographed on silica gel column with EtOAc/MeOH (40:1 to 20:1, v/v) containing 0.5% Et_3N as an eluent to offer PTX-7-K-EG_3 as white solid (29.0 mg, 86.0%). **^{1}H NMR** (400 MHz, CD_3CN) δ 8.12 – 8.07 (m, 2H), 7.86 – 7.81 (m, 2H), 7.69 – 7.64 (m, 1H), 7.63 – 7.51 (m, 4H), 7.47 (td, *J* = 6.8, 6.2, 1.6 Hz, 3H), 7.43 – 7.38 (m, 2H), 7.33 – 7.27 (m, 1H), 6.35 (s, 1H), 6.12 – 6.03 (m, 1H), 5.61 (dd, *J* = 8.7, 4.4 Hz, 1H), 5.57 (d, *J* = 7.1 Hz, 1H), 4.92 (dd, *J* = 9.9, 2.1 Hz, 1H), 4.72 (d, *J* = 4.5 Hz, 1H), 4.40 (dd, *J* = 10.8, 6.5 Hz, 1H), 4.18 – 4.09 (m, 2H), 3.78 (d, *J* = 7.0 Hz, 1H), 3.59 – 3.40 (m, 12H), 3.27 (s, 3H), 2.83 (ddd, *J* = 15.5, 9.9, 6.4 Hz, 2H), 2.36 (s, 4H), 2.28 (dd, *J* = 15.5, 9.3 Hz, 2H), 2.11 (s, 3H), 2.02 – 1.96 (m, 1H), 1.87 (d, *J* = 1.5 Hz, 3H), 1.80 (ddd, *J* = 14.9, 10.8, 2.4 Hz, 1H), 1.67 (s, 3H), 1.44 (s, 3H), 1.27 (s, 6H), 1.16 (s, 3H), 1.12 (s, 6H). **^{13}C NMR** (100 MHz, CD_3CN) δ 204.4, 175.3, 173.1, 171.8, 169.5, 168.4, 142.1, 141.6, 136.8, 136.3, 135.9, 134.1, 132.6, 132.4, 131.2, 131.0, 130.9, 130.2, 129.8, 103.5, 86.3, 83.1, 80.4, 78.4,

77.2, 77.1, 76.2, 75.1, 74.1, 73.6, 72.7, 72.6, 72.6, 72.5, 62.7, 60.3, 59.7, 58.6, 49.6, 45.6, 38.1, 37.1, 28.4, 26.9, 25.8, 24.7, 23.4, 22.5, 16.4, 13.0. **HRMS** (MALDI): m/z calcd for $C_{57}H_{71}NO_{18}$ $[M+Na]^+$: 1080.4563; found: 1080.4567. Purity of PTX-7-K-EG_3 was examined using HPLC.

Synthesis of PK3F

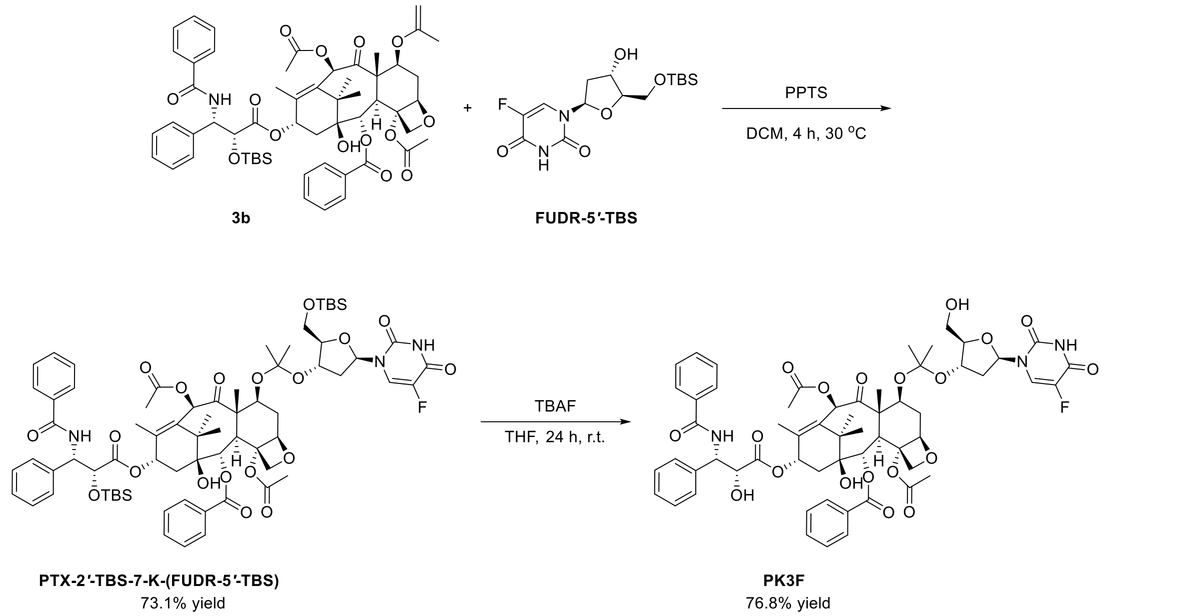

FUDR-5′-TBS was synthesized as previously reported.[24] To a solution of **3b** (75.6 mg, 75.0 μmol) and FUDR-5′-TBS (9.0 mg, 25.0 μmol) in anhydrous DCM (2 mL) was added PPTS (1.3 μmol, 32.7 μL of 10 mg/mL in DCM) under N_2 atmosphere, and the resulting reaction mixture was stirred for 4 h at 30 °C. The reaction mixture was quenched with Et_3N (0.2 mL) and concentrated in vacuo. The residue was chromatographed on silica gel column with EtOAc/PE (1:10 to 1:3, v/v) containing 0.5% Et_3N as an eluent to offer PTX-2′-TBS-7-K-(FUDR-5′-TBS) as white solid (25.0 mg, 73.1%). **^{1}H NMR** (400 MHz, CD_3CN) δ 8.14 – 8.09 (m, 2H), 7.87 (d, *J* = 6.9 Hz, 1H), 7.79 – 7.76 (m, 2H), 7.70 – 7.62 (m, 1H), 7.61 – 7.37 (m, 10H), 7.33 – 7.25 (m, 1H), 6.36 (s, 1H), 6.09 (td, *J* = 8.1, 7.0, 4.0 Hz, 2H), 5.72 (dd, *J* = 9.3, 4.3 Hz, 1H), 5.59 (d, *J* = 7.1 Hz, 1H), 4.95 (dd, *J* = 9.8, 2.2 Hz, 1H), 4.78 (d, *J* = 4.3 Hz, 1H), 4.53 – 4.38 (m, 2H), 4.24 – 4.11 (m, 2H), 4.02 (q, *J* = 2.3 Hz, 1H), 3.83 (d, *J* = 2.6 Hz, 3H), 2.88 – 2.74 (m, 2H), 2.56 (s, 3H), 2.43 – 2.17 (m, 4H), 2.11 (s, 3H), 1.86 (d, *J* = 1.5 Hz, 3H), 1.68 (s, 3H), 1.49 (s, 3H), 1.18 (s, 3H), 1.12 (d, *J* = 1.7 Hz, 6H), 0.94 (s, 9H), 0.81 (s, 9H), 0.15 (d, *J* = 2.9 Hz, 6H), -0.02 (s, 3H), -0.16 (s, 3H). **^{13}C NMR** (100 MHz, CD_3CN) δ 202.3, 172.7, 171.3, 170.2, 167.7, 166.7, 158.0, 157.7, 149.7, 142.5, 140.5, 140.2, 139.3, 135.3, 134.6, 134.2, 132.3, 130.8, 130.8, 129.4, 129.3, 129.2, 128.6, 128.2, 127.9, 124.8, 124.5, 102.5, 87.9, 86.2, 84.3, 81.3, 78.7, 76.7, 76.2, 75.4, 75.4, 73.4, 72.3, 72.0, 63.9, 57.9, 57.0, 47.7, 43.9, 40.0, 36.5, 36.4, 34.5, 32.0, 26.9, 26.0, 25.9, 25.8, 25.7, 24.1, 23.4, 23.1, 22.8, 21.9, 20.8, 18.8, 18. 6, 14.8, 14.8, 14.1, 11.3, -5.2, -5.4, -5.5, -5.6. **HRMS** (MALDI): m/z calcd for $C_{71}H_{94}FN_3O_{19}Si_2$ $[M+Na]^+$: 1390.5896; found: 1390.5899.

To a solution of PTX-2′-TBS-7-K-(FUDR-5′-TBS) (21.9 mg, 16.0 μmol) in anhydrous THF (0.5 mL) was added TBAF (50 μL of 1.0 M THF solution, 50.0 μmol) under N_2 atmosphere, and the resulting reaction mixture was stirred for 24 h at room temperature. The reaction mixture was quenched with saturated $NaHCO_3$ and diluted with EtOAc, then washed with water. The combined organic layer was washed with brine, dried over anhydrous $MgSO_4$ and concentrated in vacuo. The residue was chromatographed on silica gel column with EtOAc/MeOH (40:1 to 20:1, v/v) containing 0.5% Et_3N as an eluent to offer PK3F as white solid (14.0 mg, 76.8%). **^{1}H NMR** (400 MHz, CD_3CN) δ 8.12 – 8.06 (m, 2H), 7.95 (d, *J* = 7.1 Hz, 1H), 7.83 (dd, *J* = 14.6, 8.0 Hz, 3H), 7.66 (t, *J* = 7.3 Hz, 1H), 7.61 – 7.36 (m, 9H), 7.28 (t, *J* = 7.4 Hz, 1H), 6.35 (s, 1H), 6.08 (qd, *J* = 9.6, 8.8, 4.9 Hz, 2H), 5.67 – 5.50 (m, 2H), 4.98 – 4.85 (m, 1H), 4.73 (d, *J* = 4.7 Hz, 1H), 4.43 (dd, *J* = 11.0, 6.3 Hz, 2H), 4.21 – 4.08 (m, 2H), 3.94 (q, *J* = 2.7 Hz, 1H), 3.84 – 3.60 (m, 7H), 2.93 – 2.71 (m, 2H), 2.35 (s, 3H), 2.31 – 2.16 (m, 2H), 2.11 (s, 3H), 1.90 – 1.86 (m, 3H), 1.74 (ddd, *J* = 14.0, 10.9, 2.3 Hz, 1H), 1.68 (s, 3H), 1.45 (s, 3H), 1.18 (s, 3H), 1.12 (s, 6H). **^{13}C NMR** (100 MHz, CD_3CN) δ 202.5, 173.4, 171.1, 170.0, 167.5, 166.4, 157.8, 157.5, 149.7, 142.3, 140.3, 140.0, 139.6, 134.8, 134.3, 134.1, 132.2, 130.6, 130.5, 129.3, 129.1, 129.0, 128.3, 127.9, 125.2, 124.9, 102.4, 87.8, 85.9, 84.1, 81.1, 76.5, 75.3, 75.2, 74.3, 73.3, 72.1, 71.6, 61.9, 57.8, 56.7, 47.6, 46.5, 39.3, 36.3, 36.2, 26.5, 26.4, 24.1, 22.8, 21.5, 20.6, 10.4. **HRMS** (MALDI): m/z calcd for $C_{59}H_{66}FN_3O_{19}$ $[M+Na]^+$: 1162.4167; found: 1162.4172. Purity of PK3F was examined using HPLC.

Synthesis of PK5F

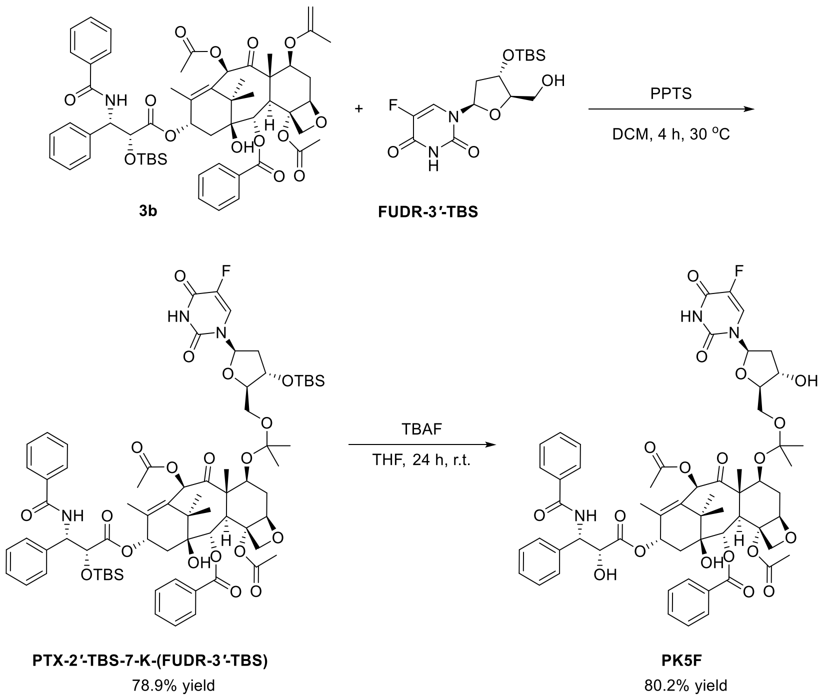

FUDR-3′-TBS was synthesized as previously reported.[28] To a solution of **3b** (75.6 mg, 75.0 μmol) and FUDR-3′-TBS (9.0 mg, 25.0 μmol) in anhydrous DCM (2 mL) was added PPTS (1.3 μmol, 32.7 μL of 10 mg/mL in DCM) under N_2 atmosphere, and the resulting reaction mixture was stirred

for 4 h at 30 ^{o}C. The reaction mixture was quenched with Et_3N (0.2 mL) and concentrated in vacuo. The residue was chromatographed on silica gel column with EtOAc/PE (1:10 to 1:3, v/v) containing 0.5% Et_3N as an eluent to offer PTX-2′-TBS-7-K-(FUDR-3′-TBS) as white solid (27.0 mg, 78.9%). **^{1}H NMR** (400MHz, $CDCl_3$) δ 8.19 – 8.07 (m, 2H), 7.95 (dd, *J* = 6.5, 1.4 Hz, 1H), 7.80 – 7.67 (m, 2H), 7.60 (t, *J* = 7.4 Hz, 1H), 7.56 – 7.46 (m, 3H), 7.44 – 7.30 (m, 7H), 7.07 (d, *J* = 9.0 Hz, 1H), 6.44 (s, 1H), 6.34 – 6.23 (m, 1H), 6.18 (t, *J* = 5.9 Hz, 1H), 5.75 (d, *J* = 9.4 Hz, 1H), 5.69 (d, *J* = 7.1 Hz, 1H), 4.87 (d, *J* = 9.1 Hz, 1H), 4.69 (d, *J* = 2.1 Hz, 1H), 4.50 (dd, *J* = 10.7, 6.3 Hz, 1H), 4.37 – 4.22 (m, 1H), 4.18 (d, *J* = 8.4 Hz, 1H), 3.92 – 3.83 (m, 2H), 3.78 (dd, *J* = 11.1, 2.8 Hz, 1H), 3.70 – 3.53 (m, 2H), 2.43-2.23 (m, 8H), 2.19 (s, 3H), 2.17 – 1.98 (m, 2H), 1.98 (s, 3H), 1.76 (s, 3H), 1.64 (s, 3H), 1.29 (s, 3H), 1.20 (d, *J* = 3.2 Hz, 6H), 0.86 (s, 9H), 0.80 (s, 9H), 0.05 (s, 6H), -0.03 (s, 3H), -0.29 (s, 3H). **^{13}C NMR** (100MHz, $CDCl_3$) δ 201.8, 171.6, 170.0, 169.5, 167.0, 151.3, 142.4, 140.0, 138.2, 134.1, 133.7, 133.6, 131.8, 130.3, 129.2, 128.8, 128.0, 127.0, 126.4, 123.1, 123.1, 101.8, 85.6, 84.9, 84.1, 81.0, 78.7, 77.2, 76.4, 75.1, 74.8, 74.7, 72.8, 71.4, 70.9, 59.8, 57.4, 55.7, 52.9, 46.9, 43.3, 41.3, 35.6, 34.6, 29.7, 26.5, 25.7, 25.6, 25.5, 25.1, 23.7, 23.1, 21.6, 20.9, 18.1, 17.9, 14.5, 11.2, 8.1, -4.9, -5.2, -5.8. **HRMS** (MALDI): m/z calcd for $C_{71}H_{94}FN_3O_{19}Si_2$ $[M+Na]^+$: 1390.5896; found: 1390.5898.

To a solution of PTX-2′-TBS-7-K-(FUDR-3′-TBS) (24.0 mg, 17.5 μmol) in anhydrous THF (0.5 mL) was added TBAF (53 μL of 1.0 M THF solution, 53.0 μmol) under N_2 atmosphere, and the resulting reaction mixture was stirred for 24 h at room temperature. The reaction mixture was quenched with saturated $NaHCO_3$ and diluted with EtOAc, then washed with water. The combined organic layer was washed with brine, dried over anhydrous $MgSO_4$ and concentrated in vacuo. The residue was chromatographed on silica gel column with EtOAc/MeOH (40:1 to 20:1, v/v) containing 0.5% Et_3N as an eluent to offer PK5F as white solid (16.0 mg, 80.2%). **^{1}H NMR** (400 MHz, $CDCl_3$) δ 8.14 – 8.07 (m, 2H), 7.96 (d, *J* = 6.5 Hz, 1H), 7.82 – 7.74 (m, 2H), 7.61 (t, *J* = 7.4 Hz, 1H), 7.55 – 7.44 (m, 5H), 7.44 – 7.36 (m, 4H), 7.32 (t, *J* = 7.4 Hz, 1H), 7.24 (d, *J* = 9.0 Hz, 1H), 6.41 (s, 1H), 6.17 (q, *J* = 8.7, 6.9 Hz, 2H), 5.78 (dd, *J* = 8.9, 2.9 Hz, 1H), 5.67 (d, *J* = 6.9 Hz, 1H), 4.86 (d, *J* = 9.5 Hz, 1H), 4.79 (d, *J* = 3.0 Hz, 1H), 4.42 (dd, *J* = 10.4, 6.6 Hz, 1H), 4.30 (dd, *J* = 12.1, 7.0 Hz, 2H), 4.16 (d, *J* = 8.5 Hz, 1H), 3.94 (d, *J* = 4.5 Hz, 1H), 3.88 – 3.62 (m, 8H), 2.77 (dq, *J* = 14.3, 7.5, 6.3 Hz, 1H), 2.36 (s, 3H), 2.34 – 2.24 (m, 2H), 2.18 (s, 3H), 2.10 (dd, *J* = 13.7, 6.7 Hz, 1H), 1.87 (s, 3H), 1.75 (s, 3H), 1.55 (s, 3H), 1.33 (s, 3H), 1.19 (d, *J* = 7.4 Hz, 6H). **^{13}C NMR** (100 MHz, CD_3CN) δ 173.5, 171.2, 170.0, 167.7, 166.5, 157.7, 157.5, 149.4, 142.3, 140.4, 139.9, 139.7, 134.9, 134.4, 134.2, 132.3, 130.7, 130.6, 129.4, 129.2, 129.1, 128.4, 128.0, 127.9, 125.5, 125.1, 102.2, 86.2, 85.5, 84.3, 81.2, 78.5, 76.6, 75.4, 75.3, 74.4, 73.5, 71.8, 70.5, 60.8, 57.9, 56.8, 43.8, 40.4, 36.3, 35.3, 26.6, 25.1, 23.8, 22.9, 21.6, 20.7, 14.7, 14.6. **HRMS** (MALDI): m/z calcd for $C_{59}H_{66}FN_3O_{19}$ $[M+Na]^+$: 1162.4167; found: 1162.4170. Purity of PK5F was examined using HPLC.

Synthesis of PK5E

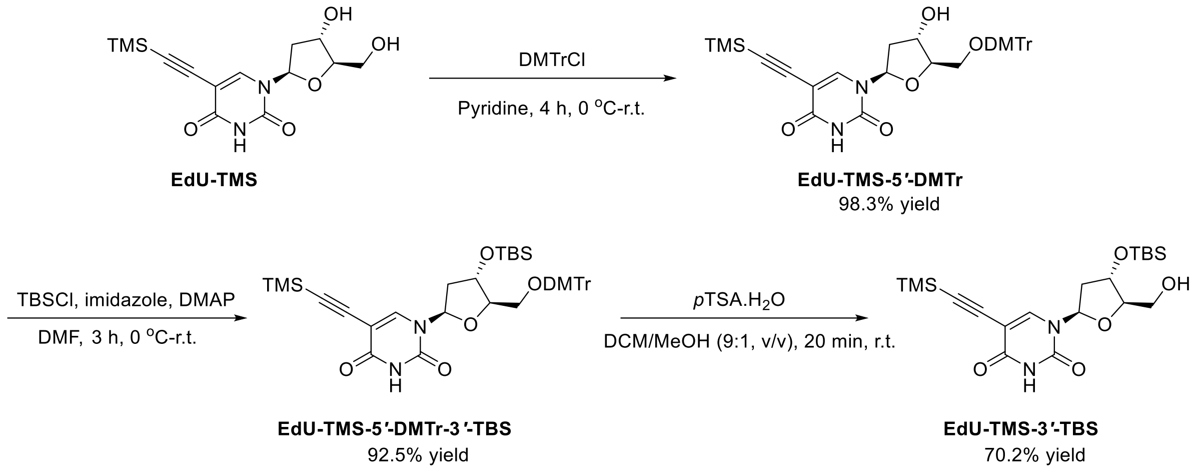

EdU-TMS was prepared according to reported procedures.[29] To a solution of EdU-TMS (5.3 g, 16.4 mmol) in anhydrous pyridine (20 mL) under N_2 atmosphere was added DMtrCl (5.7 g, 16.9 mmol in 10 mL anhydrous pyridine) dropwise at 0 °C. After that, the reaction mixture was warmed to temperature, and stirred for 4 h. The reaction mixture was quenched with saturated NaCl, and pyridine was removed with rotary evaporator. Then the residue was diluted with DCM. The solution was washed with water, brine, dried over anhydrous $MgSO_4$ and concentrated in vacuo. The residue was chromatographed on silica gel column with EtOAc/PE (from 1:5 to 2:1, v/v) as an eluent to offer EdU-TMS-5′-DMTr as white solid (10.1 g, 98.3%). **^{1}H NMR** (400 MHz, $CDCl_3$) δ 7.96 (s, 1H), 7.45 – 7.34 (m, 2H), 7.31 – 7.08 (m, 8H), 6.84 – 6.72 (m, 4H), 6.22 (dd, *J* = 7.9, 5.6 Hz, 1H), 4.39 (dt, *J* = 5.6, 2.6 Hz, 1H), 4.04 (q, *J* = 3.3 Hz, 1H), 3.72 (s, 6H), 3.35 (dd, *J* = 10.7, 3.3 Hz, 1H), 3.25 (dd, *J* = 10.6, 3.8 Hz, 1H), 2.45 (ddd, *J* = 13.8, 5.7, 2.4 Hz, 1H), 2.15 (ddd, *J* = 13.7, 8.0, 6.0 Hz, 1H), -0.05 (d, *J* = 0.9 Hz, 9H). **^{13}C NMR** (100 MHz, $CDCl_3$) δ 159.0, 144.8, 143.0, 135.9, 135.9, 130.4, 130.3, 113.7, 101.0, 100.1, 95.2, 87.3, 86.9, 86.2, 72.8, 63.9, 60.8, 55.6, 41.8, 21.4, 14.6.

EdU-TMS-5′-DMTr (10.1 g, 16.1 mmol) was added to a solution of imidazole (3.3 g, 48.6 mmol) and DMAP (195.5 mg, 1.6 mmol) in anhydrous DMF (60 mL) under N_2 atmosphere, and the resulting reaction mixture was cooled to 0 °C, added TBSCl (2.6 g, 17.0 mmol) and warmed to temperature. After stirred for 3 h, the reaction mixture was quenched with saturated NaCl and diluted with EtOAc. The organic layer was washed with water, brine, dried over anhydrous $MgSO_4$ and concentrated in vacuo. The residue was chromatographed on silica gel column with EtOAc/PE (from 1:5 to 1:1, v/v) as an eluent to offer EdU-TMS-5′-DMTr-3′-TBS as white solid (11.1 g, 92.5%). **^{1}H NMR** (400 MHz, $CDCl_3$) δ 8.08 (s, 1H), 7.48 – 7.40 (m, 2H), 7.39 – 7.27 (m, 5H), 7.25 – 7.17 (m, 1H), 6.91 – 6.80 (m, 4H), 6.25 (dd, *J* = 7.9, 5.6 Hz, 1H), 4.33 (dt, *J* = 5.4, 2.4 Hz, 1H), 3.99 (q, *J* = 3.0 Hz, 1H), 3.79 (s, 6H), 3.43 (dd, *J* = 10.8, 3.0 Hz, 1H), 3.22 (dd, *J* = 10.8, 3.6 Hz, 1H), 2.95 (s, 1H), 2.88 (s, 1H), 2.35 (ddd, *J* = 13.2, 5.7, 2.4 Hz, 1H), 2.11 (ddd, *J* = 13.5, 8.0, 5.9 Hz, 1H), 0.83 (s, 9H), -0.00 (s, 3H), -0.03 (s, 9H), -0.05 (s, 3H). **^{13}C NMR** (100 MHz, $CDCl_3$)

δ 159.1, 159.0, 149.3, 144.9, 143.2, 136.0, 135.9, 130.4, 130.3, 128.5, 128.4, 127.3, 113.8, 113.7, 100.9, 100.0, 95.2, 87.8, 87.3, 86.3, 73.0, 63.5, 55.6, 42.4, 36.9, 31.8, 26.1, 18.3, -4.3, -4.5.

EdU-TMS-5′-DMTr-3′-TBS (11.1 g, 15.0 mmol) was added to a mixture solution of DCM and MeOH (9:1, v/v), then was added with *p*TSA.H_2O (2.9 g, 15.0 mmol), the reaction mixture was stirred for 20 min at room temperature. After the reaction was finished, saturated $NaHCO_3$ was added to quench the reaction, remove DCM with rotary evaporator, then the residue was diluted with EtOAc, the organic layer was washed with water, brine, dried over anhydrous $MgSO_4$ and concentrated in vacuo. The residue was reprecipitation with hexane and afford EdU-TMS-3′-TBS as white solid (4.6 g, 70.2%). **^{1}H NMR** (400 MHz, $CDCl_3$) δ 7.92 (s, 1H), 6.13 (d, *J* = 6.5 Hz, 1H), 4.64 – 4.33 (m, 1H), 4.07 – 3.85 (m, 2H), 3.82 – 3.70 (m, 1H), 2.28 (ddd, *J* = 6.7, 5.2, 1.9 Hz, 2H), 0.89 (s, 9H), 0.23 (s, 9H), 0.09 (s, 6H). **^{13}C NMR** (100 MHz, $CDCl_3$) δ 161.1, 149.2, 144.5, 100.4, 99.8, 95.4, 88.0, 87.4, 71.6, 62.1, 41.2, 25.8, 18.1, -4.6, -4.7. **HRMS** (MALDI): m/z calcd for $C_{20}H_{34}N_2O_5Si_2$ $[M+Na]^+$: 461.1898; found: 461.1895.

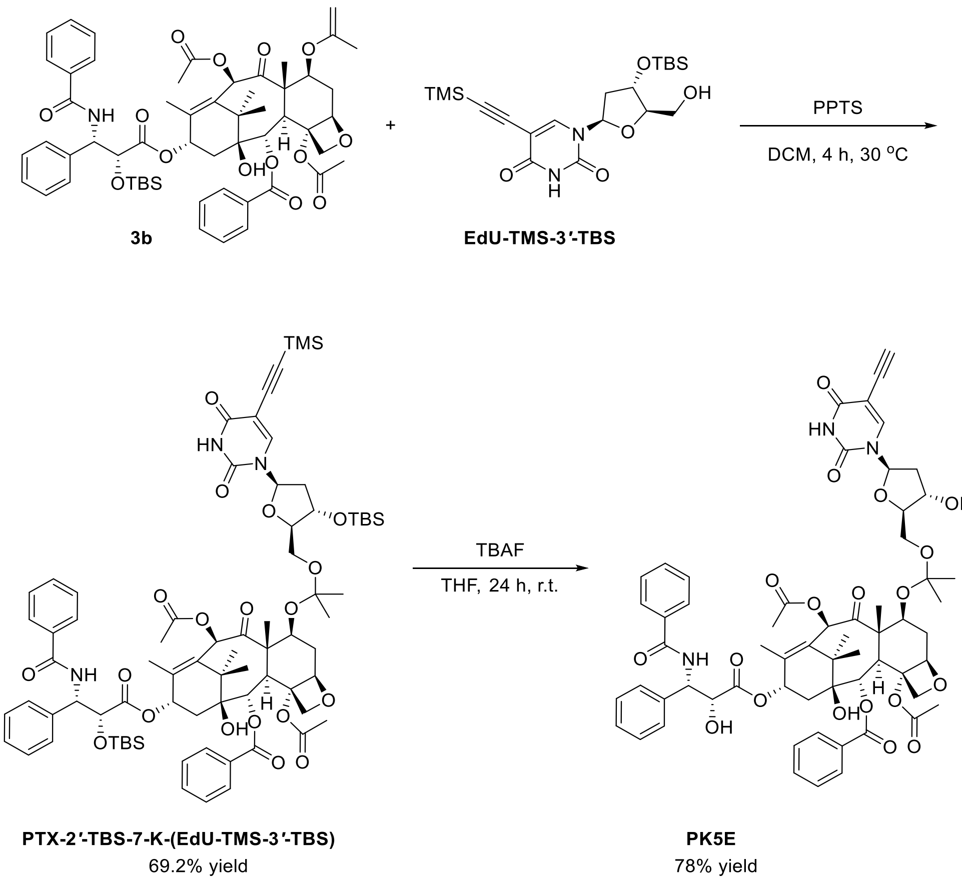

To a solution of **3b** (75.6 mg, 75.0 μmol) and EdU-TMS-3′-TBS (11.0 mg, 25.0 μmol) in anhydrous DCM (2 mL) was added PPTS (1.3 mmol, 32.7 μL of 10.0 mg/mL in DCM) under N_2 atmosphere, and the resulting mixture was stirred for 4 h at 30 °C. The reaction mixture was quenched with Et_3N (0.2 mL) and concentrated in vacuo. The residue was chromatographed on silica gel column with EtOAc/PE (1:10 to 1:3, v/v) containing 0.5% Et_3N as an eluent to offer

PTX-2′-TBS-7-K-(EdU-TMS-3′-TBS) as white solid (25.0 mg, 69.2%). **^{1}H NMR** (400 MHz, CD_3CN) δ 8.17 – 8.07 (m, 2H), 7.86 (s, 1H), 7.81 – 7.74 (m, 2H), 7.71 – 7.63 (m, 1H), 7.60 – 7.37 (m, 11H), 7.35 – 7.23 (m, 1H), 6.40 (s, 1H), 6.19 – 6.00 (m, 2H), 5.72 (dd, *J* = 9.4, 4.1 Hz, 1H), 5.58 (d, *J* = 7.1 Hz, 1H), 4.87 (dd, *J* = 9.9, 2.2 Hz, 1H), 4.74 (d, *J* = 4.1 Hz, 1H), 4.44 (dd, *J* = 10.7, 6.4 Hz, 1H), 4.33 (q, *J* = 5.8 Hz, 1H), 4.14 (d, *J* = 3.2 Hz, 2H), 3.89 – 3.81 (m, 2H), 3.76 (dd, *J* = 11.0, 3.1 Hz, 1H), 3.45 (dd, *J* = 11.0, 5.4 Hz, 1H), 2.90 – 2.72 (m, 1H), 2.54 (s, 3H), 2.41 – 2.13 (m, 3H), 2.11 (s, 3H), 1.88-1.70 (m, 5H), 1.69 (s, 3H), 1.59 (s, 3H), 1.23 (s, 3H), 1.12 (s, 6H), 0.87 (s, 9H), 0.80 (s, 9H), 0.22 (s, 9H), 0.08 (d, *J* = 2.3 Hz, 6H), -0.02 (s, 3H), -0.18 (s, 3H). **^{13}C NMR** (100 MHz, CD_3CN) δ 202.4, 172.5, 170.9, 169.9, 167.6, 166.5, 161.9, 149.9, 144.5, 140.4, 139.1, 135.2, 134.5, 134.1, 132.1, 130.7, 130.6, 129.3, 129.1, 129.1, 128.4, 127.9, 127.8, 101.9, 99.4, 98.3, 97.5, 86.4, 86.0, 84.3, 81.2, 78.6, 76.5, 76.0, 75.3, 75.1, 73.5, 71.9, 71.3, 60.0, 57.8, 56.8, 47.4, 43.8, 41.2, 36.2, 35.2, 26.5, 25.7, 25.6, 25.2, 24.3, 23.3, 21.8, 20.6, 18.4, 18.2, 14.7, 11.6, -0.3, -4.9, -5.1, -5.2, -5.5. **HRMS** (MALDI): m/z calcd for $C_{76}H_{103}N_3O_{19}Si_3$ $[M+Na]^+$: 1468.6386; found: 1468.6389.

To a solution of PTX-2′-TBS-7-K-(EdU-TMS-3′-TBS) (23.1 mg, 16.0 μmol) in anhydrous THF (0.5 mL) was added TBAF (64 μL of 1.0 M THF solution, 64.0 μmol) under N_2 atmosphere, and the resulting mixture was stirred for 24 h at room temperature. The reaction mixture was quenched with saturated $NaHCO_3$ and diluted with EtOAc, then washed with water. The combined organic layer was washed with brine, dried over anhydrous $MgSO_4$ and concentrated in vacuo. The residue was chromatographed on silica gel column with EtOAc/MeOH (40:1 to 20:1, v/v) containing 0.5% Et_3N as an eluent to offer PK5E as white solid (14.3 mg, 78.0%). **^{1}H NMR** (400 MHz, CD_3CN) δ 8.16 – 8.04 (m, 2H), 7.87 (dd, *J* = 13.1, 8.1 Hz, 2H), 7.66 (t, *J* = 7.4 Hz, 1H), 7.60 – 7.35 (m, 9H), 7.29 (t, *J* = 7.3 Hz, 1H), 6.37 (s, 1H), 6.06 (q, *J* = 7.1, 6.0 Hz, 2H), 5.68 – 5.49 (m, 2H), 4.90 (d, *J* = 9.1 Hz, 1H), 4.73 (d, *J* = 4.7 Hz, 1H), 4.43 (dd, *J* = 10.7, 6.5 Hz, 1H), 4.22 (q, *J* = 5.0 Hz, 1H), 4.13 (s, 2H), 3.90 (q, *J* = 3.7 Hz, 1H), 3.80 (d, *J* = 7.0 Hz, 1H), 3.71 (dd, *J* = 11.4, 2.7 Hz, 1H), 3.53 (dd, *J* = 11.3, 4.4 Hz, 1H), 3.45 (s, 1H), 3.24 (q, *J* = 7.2 Hz, 1H), 2.86 (ddd, *J* = 15.6, 10.0, 6.7 Hz, 1H), 2.35 (s, 3H), 2.24 (dt, *J* = 11.1, 7.9 Hz, 3H), 2.12 (s, 3H), 1.94-1.92 (m, 6H), 1.88 (s, 3H), 1.68 (s, 3H), 1.56 (s, 3H), 1.21 (s, 3H), 1.12 (s, 6H). **^{13}C NMR** (100 MHz, CD_3CN) δ 202.7, 173.6, 171.4, 170.1, 167.9, 166.7, 162.3, 150.2, 145.2, 140.6, 140.0, 135.2, 134.7, 134.26, 132.3, 130.9, 130.8, 129.5, 129.3, 129.2, 128.5, 128.2, 128.1, 102.4, 98.5, 87.0, 86.4, 84.6, 82.6, 81.5, 78.7, 76.8, 76.5, 75.5, 75.4, 74.6, 73.7, 71.8, 71.3, 61.2, 59.3, 58.2, 57.0, 47.8, 44.0, 41.2, 36.5, 35.4, 26.8, 25.4, 24.1, 23.1, 21.7, 20.8, 14.7, 11.3, 8.3. **HRMS** (MALDI): m/z calcd for $C_{61}H_{67}N_3O_{19}$ $[M+Na]^+$: 1168.4261; found: 1168.4265. Purity of PK5E was examined using HPLC.

10) In Vitro and In Vivo Characterizations of PTX Prodrugs

Purity Examination for PTX Prodrugs

PTX-7-K-EG_3: HPLC analyses were performed using Agilent 1260 with C18 column (Agilent, Poroshell 120 EC-C18, 4.6 mm × 100 mm, 2.7 µm). Samples were injected at a flow rate of 1.0 mL/min, column temperature at 25 ^{o}C, and UV detection wavelength at 227 nm. An aqueous solution of ACN/H_2O (65/35) was used as the mobile phase.

PK3F, PK5F, and PK5E: HPLC analyses were performed using Agilent 1260 with C18 column (Agilent, Poroshell 120 EC-C18, 4.6 mm × 100 mm, 2.7 µm). Samples were injected at a flow rate of 1.0 mL/min, column temperature at 25 ^{o}C, and UV detection wavelength at 227 nm. HPLC eluent condition: 20% [v/v] ACN, 1.0 min 20% [v/v] ACN, 4.0 min 80% [v/v] ACN, 7.0 min 80% [v/v] ACN, 7.5 min 20% [v/v] ACN, 10 min 20% [v/v] ACN.

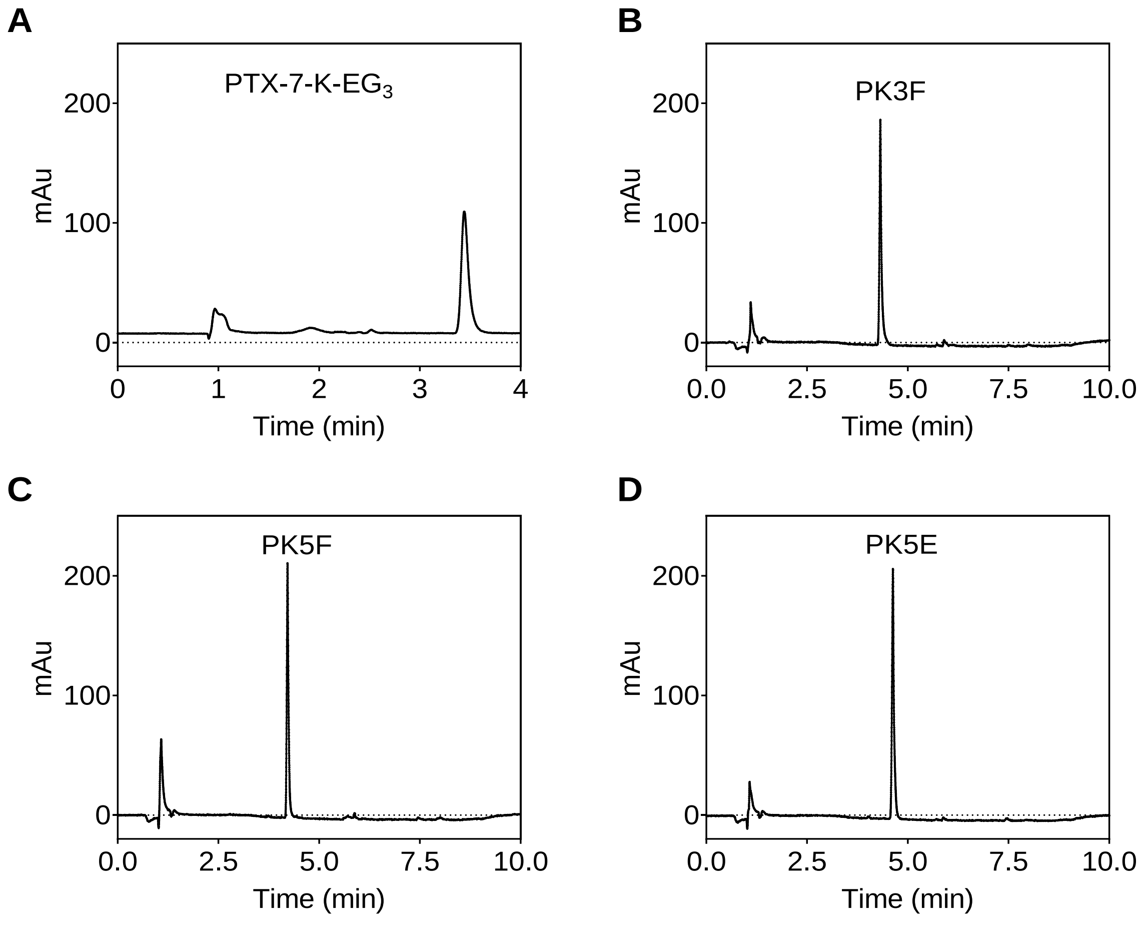

Supplementary Figure 20. HPLC chromatograms of (A) PTX-7-K-EG_3, (B) PK3F, (C) PK5F and (D) PK5E.

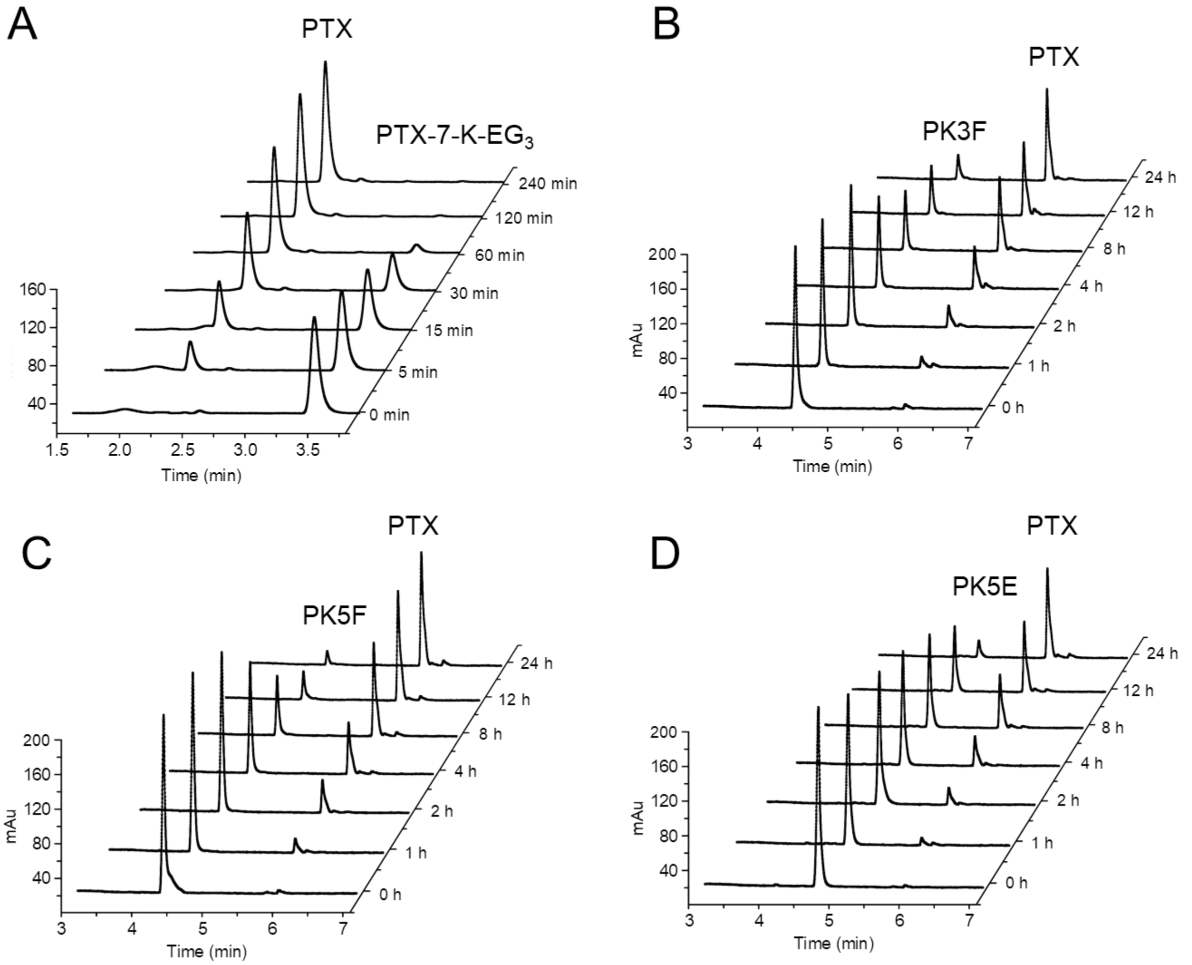

Supplementary Figure 21. HPLC chromatograms of (A) PTX-7-K-EG_3, (B) PK3F, (C) PK5F and (D) PK5E at pH 5.0. Due to the significant difference in polarity and solubility between EdU/FUDR, PTX, and heterodimers, we still could not obtain sharp peaks, excellent separation, and accurate integrations for three compounds in the same chromatogram after comprehensive optimization of HPLC conditions. Besides, the unassigned fluctuations in the chromatograms were due to the change of gradient.

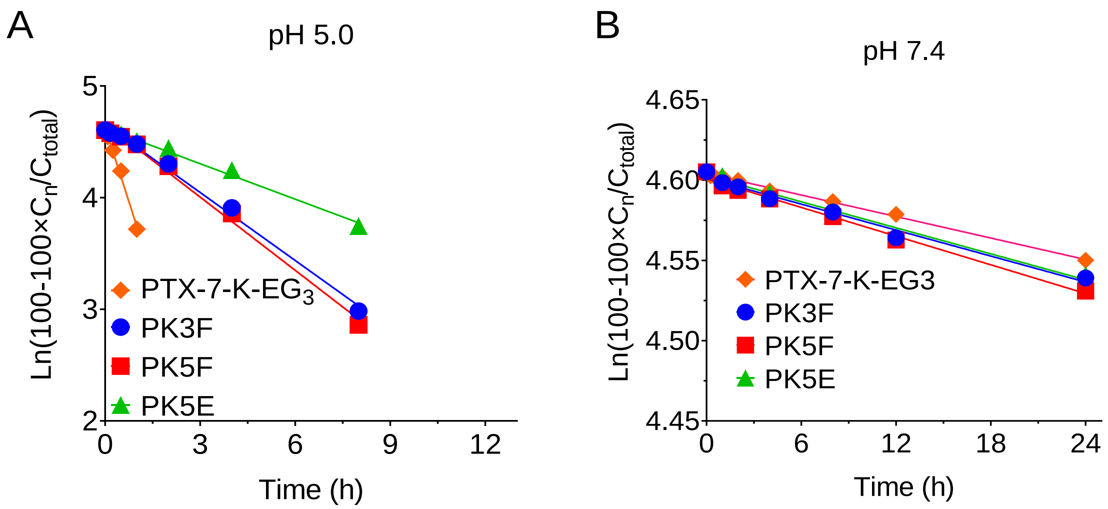

Supplementary Figure 22. Hydrolysis rate of the heterodimeric prodrugs at 37 °C at pH (A) 5.0 and (B) 7.4. "Liner regression" model was fitted to each data set. Data are means ± SD; n = 4. The hydrolysis reactions followed pseudo-first-order kinetics, and the prodrug half-lives ($t_{1/2}$) were thus calculated from the kinetic rate constants (0.693/k). Source data are provided as a Source Data file.

Supplementary Table 9. Hydrolysis parameters of PTX prodrugs

pH	Compound	$t_{1/2}$ (h)	k (h^{-1})	R^2
5.0	PTX-7-K-EG_3	0.78	0.885	0.992
	PK3F	3.41	0.203	0.993
	PK5F	3.16	0.219	0.993
	PK5E	6.54	0.106	0.991
7.4	PTX-7-K-EG_3	311	$2.23×10^{-3}$	0.996
	PK3F	256.7	$2.70×10^{-3}$	0.985
	PK5F	232.6	$2.98×10^{-3}$	0.992
	PK5E	256.7	$2.70×10^{-3}$	0.981

Supplementary Table 10. Characterization of drug-loaded micelles

	DL (wt%)[a]	LE (%)[a]	Size (nm)[b]	PDI[b]	Zeta potential (mV)[c]
PTX	9.3	92.0	25.0	0.21	-1.0
PK5F	8.9	73.8	20.9	0.28	-1.8
PK3F	8.8	74.2	23.7	0.18	-1.5
PK5E	8.9	73.7	21.6	0.21	-0.8

[a] Determined using HPLC; [b] determined using Zetasizer Nano ZS90.

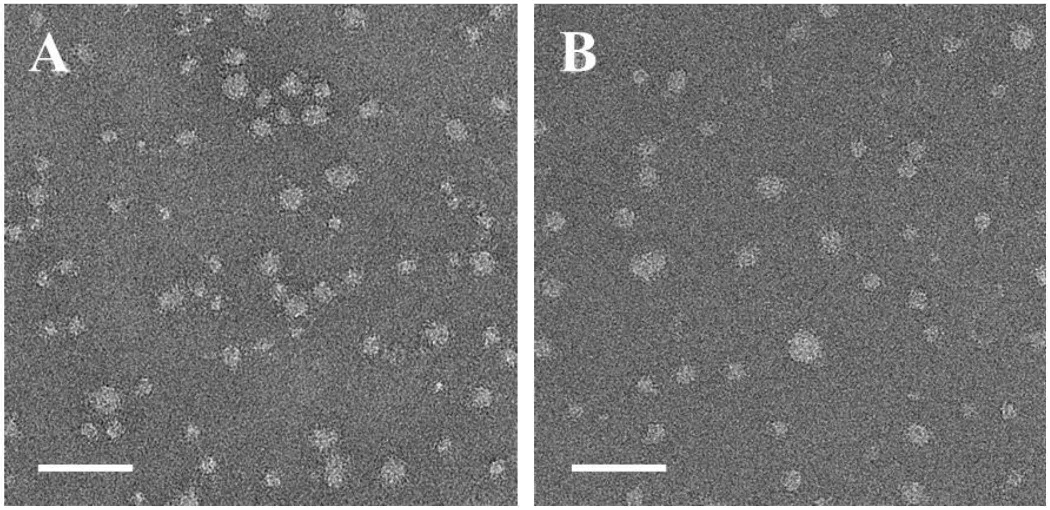

Supplementary Figure 23. Transmission electron microscope images of (A) PK3F-loaded micelles and (B) PK5E-loaded micelles. Scar bar is 50 nm. Experiments were performed three times independently, and representative images are shown.

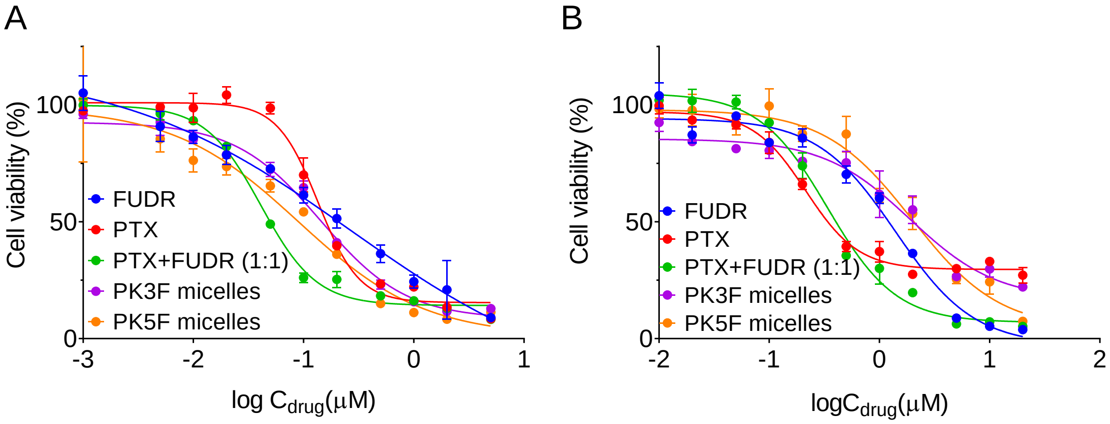

Supplementary Figure 24. Representative dose-dependent cytotoxicity of drugs for (A) HCT116 cells and (B) 3T3 cells. Data are means ± SD (n = 3 independent biological samples). Log(inhibitor) vs. response -- Variable slope (four parameters) model was fitted to each data set using the nonlinear regression analysis program of GraphPad Prism 7.0. Source data are provided as a Source Data file.

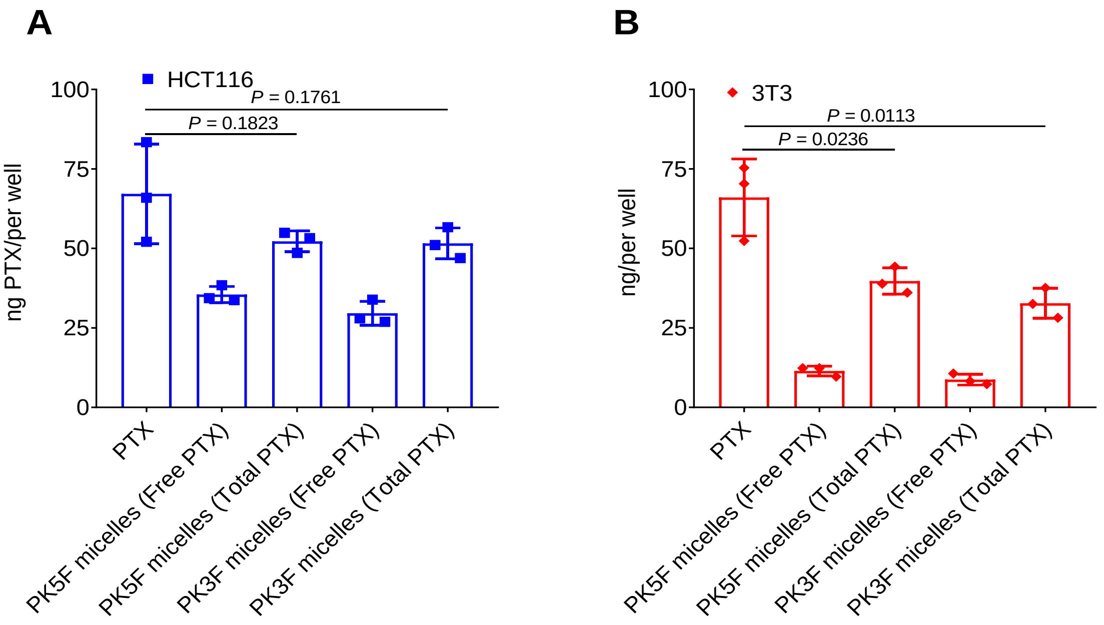

Supplementary Figure 25. Cellular uptake of drugs in (A) HCT116 cells and (B) 3T3 cells. Cells were incubated with drugs at 20 μM for 6 h. Free PTX indicates the liberated PTX from dimers. Data are means ± SD (*n* = 3 independent experiments). Unpaired two-tailed Student's t-test was used for statistical analysis. Source data are provided as a Source Data file.

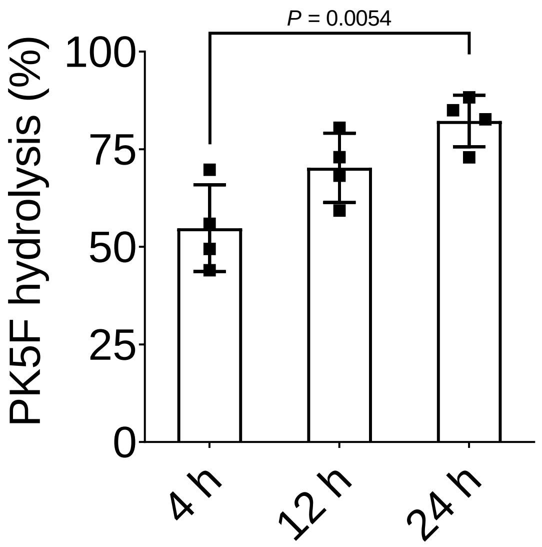

Supplementary Figure 26. Hydrolysis degree of PK5F in HCT116 tumors after single injection of PK5F-loaded micelles. Data are means ± SD (*n* = 4 biologically independent animals). Unpaired two-tailed Student's t-test was used for statistical analysis. Source data are provided as a Source Data file.

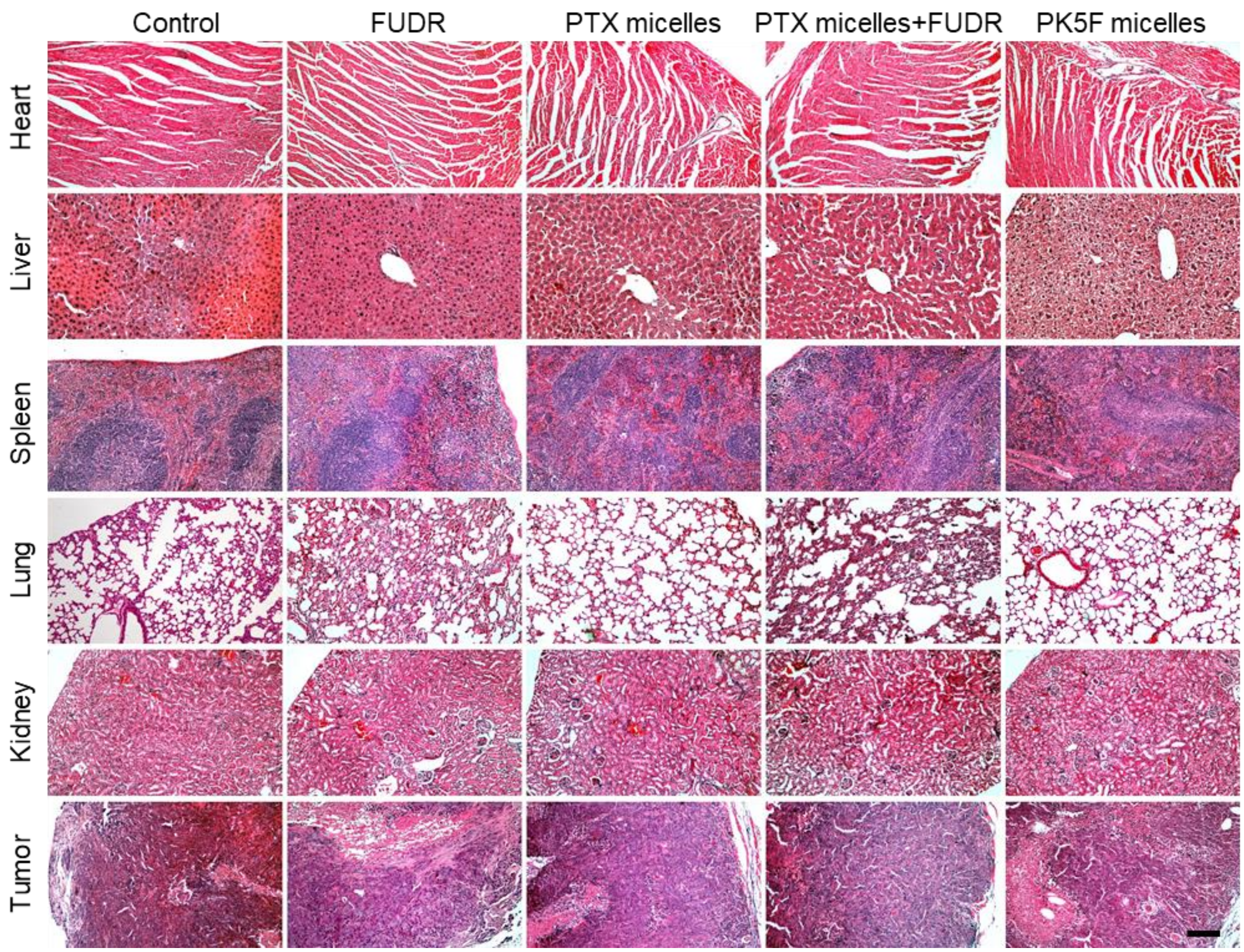

Supplementary Figure 27. Representative H&E staining of major organs and tumor at the end of the treatment. Scale bar = 200 μm. Experiments were performed five times independently, and representative images are shown.

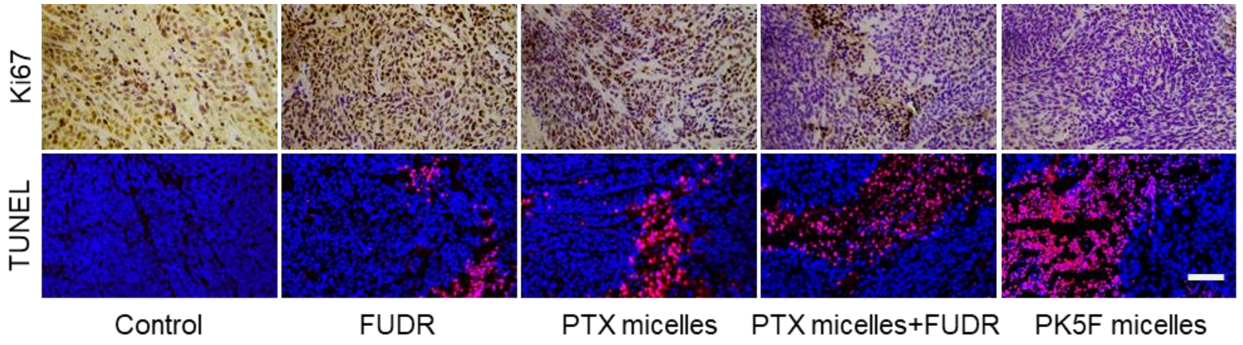

Supplementary Figure 28. Representative TUNEL (terminal deoxynucleotidyl transferase dUTP nick end labeling) and Ki67 (a proliferation marker) staining of tumor sections after treatments. Scale bar = 200 μm. Experiments were performed five times independently, and representative images are shown. Treatment with the PK5F-loaded micelles resulted in the fewest proliferating cells and the highest level of apoptosis in the tumor.

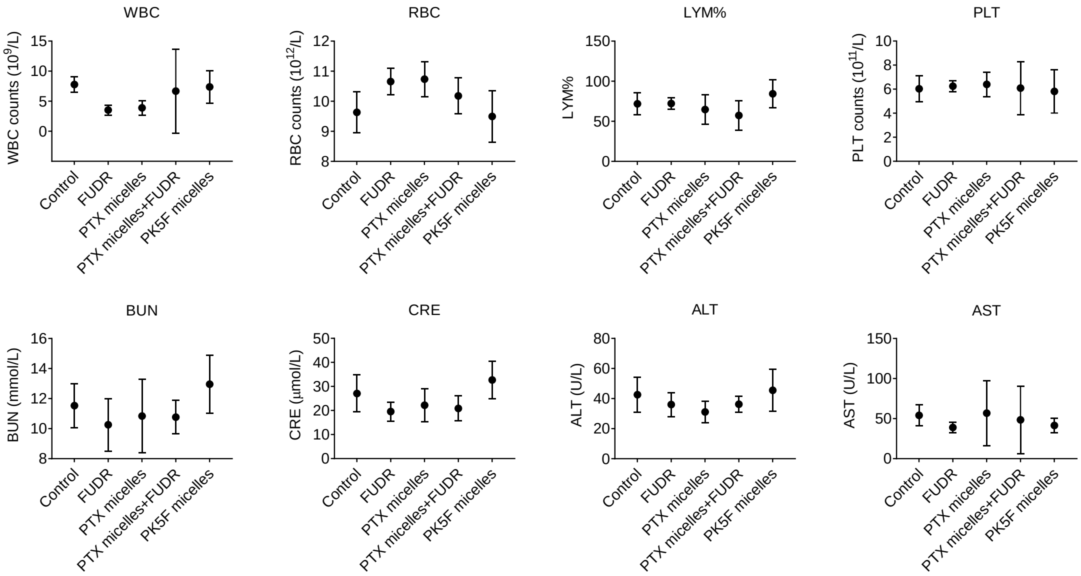

Supplementary Figure 29. Blood count test (top panel) and serum biochemical analysis (low panel) of mice at the end of the treatment. Data are means ± SD (n = 6 biologically independent animals). Source data are provided as a Source Data file.

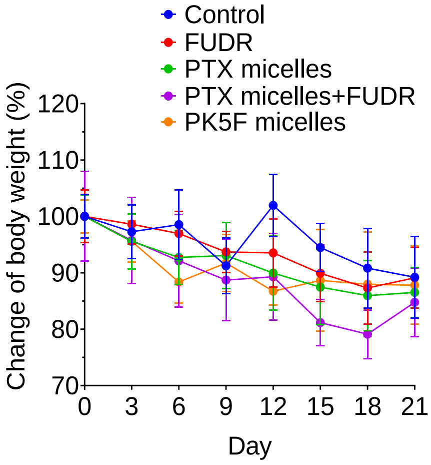

Supplementary Figure 30. Body weight change of the mice as a function of days. Data are means ± SD (n = 6 biologically independent animals). Source data are provided as a Source Data file.

11) Synthesis of Ketal-Linked Biomaterials

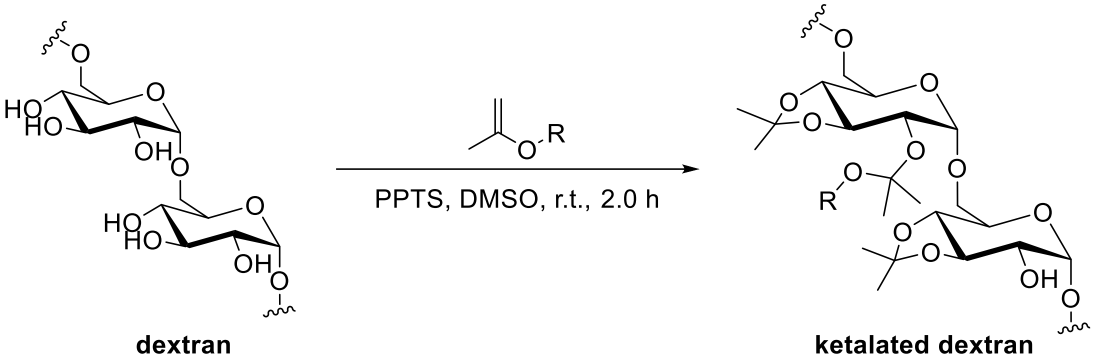

General Procedure

To a dried Schlenk flask (25 mL) was added dextran (M_w = 10,000 g/mol) and PPTS. The flask was evacuated and refilled with N_2. Then DMSO was added to the flask and stirred until complete dissolution of the dextran. Finally, IPPE was added, and the mixture was stirred at room temperature for 2 h. After the reaction was complete, 1 vol% of Et_3N was added to quench the reaction, and the ketalated dextran was precipitated by water (pH 8.0, 0.1‰ Et_3N). The crude product was centrifuged at 15,000 g for 15 min, and the resulting pellet was redissolved in acetone (containing 1% Et_3N) and drip into the water of pH 8. The sample was centrifuged in the same manner again, and the resulting pellet was dissolved in acetone and dried by anhydrous Na_2SO_4. The organic phase was then filtered and evaporated under reduced pressure to minimal volume and precipitated in hexane (containing 1% Et_3N). The precipitate was washed by hexane twice and dried under a high vacuum to yield the final product. The degree of substitution and the ratio of acyclic ketals to cyclic ketals were determined in a mixture of D_2O and CD_3CN (2/1), according to the reported method.[30]

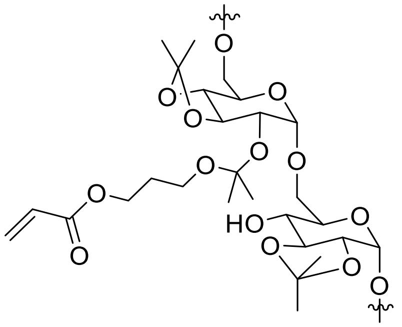

Dextran was reacted with **3t** following the general procedure (0.015 mmol dextran, 0.009 mmol PPTS, 5.55 mmol **3t** and 3 mL DMSO) and the final compound was isolated as a white solid (230 mg). **^{1}H NMR** (400 MHz, $CDCl_3$) δ 6.39 (d, acrylate), 6.11 (dd, acrylate), 5.82 (d, acrylate), 5.16 (br, dextran), 5.04 – 4.74 (br, dextran), 4.23, 4.16 – 3.10, 1.91, 1.58 – 1.28. The degree of substitution is 63%; ratio of acyclic ketals to cyclic ketals is 1:1.2.

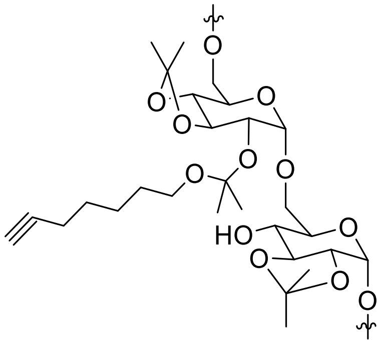

Dextran was reacted with **3r** following the general procedure (0.015 mmol dextran, 0.009 mmol PPTS, 5.55 mmol **3r** and 3 mL DMSO), and the final compound was isolated as a white solid (260 mg). **^{1}H NMR** (400 MHz, $CDCl_3$) δ 5.18 (br, dextran), 4.92 (br, dextran), 4.27 – 3.10, 2.18 (td, alkyne), 1.95 (d, alkyne), 1.67 – 1.29. The degree of substitution is 68%; ratio of acyclic ketals to cyclic ketals is 1:1.9.

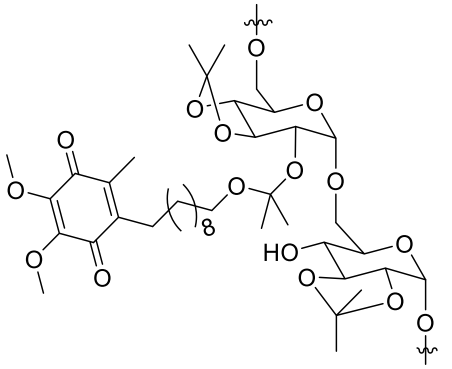

Dextran was reacted with **3k** following the general procedure (0.015 mmol dextran, 0.054 mmol PPTS, 5.55 mmol **3k** and 6 mL DMSO), and the final compound was isolated as an orange solid (180 mg). **^{1}H NMR** (400 MHz, $CDCl_3$) δ 5.15 (br, dextran), 4.85 (br, dextran), 4.10, 3.98 (s, Idebenone), 3.80, 3.43, 2.43, 2.01 (s, Idebenone), 1.60 – 1.14. The degree of substitution is 71%; ratio of acyclic ketals to cyclic ketals is 1:1.8.

12) NMR Spectra

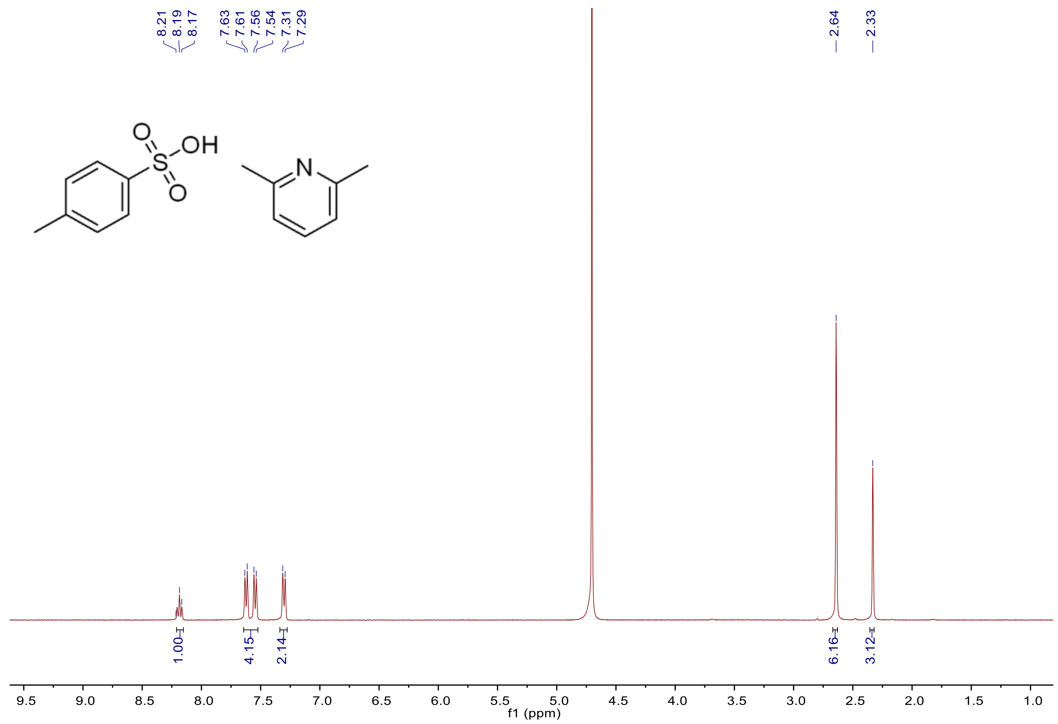

Supplementary Figure 31. ^{1}H NMR spectrum of **LPTS** (400 MHz, D_2O)

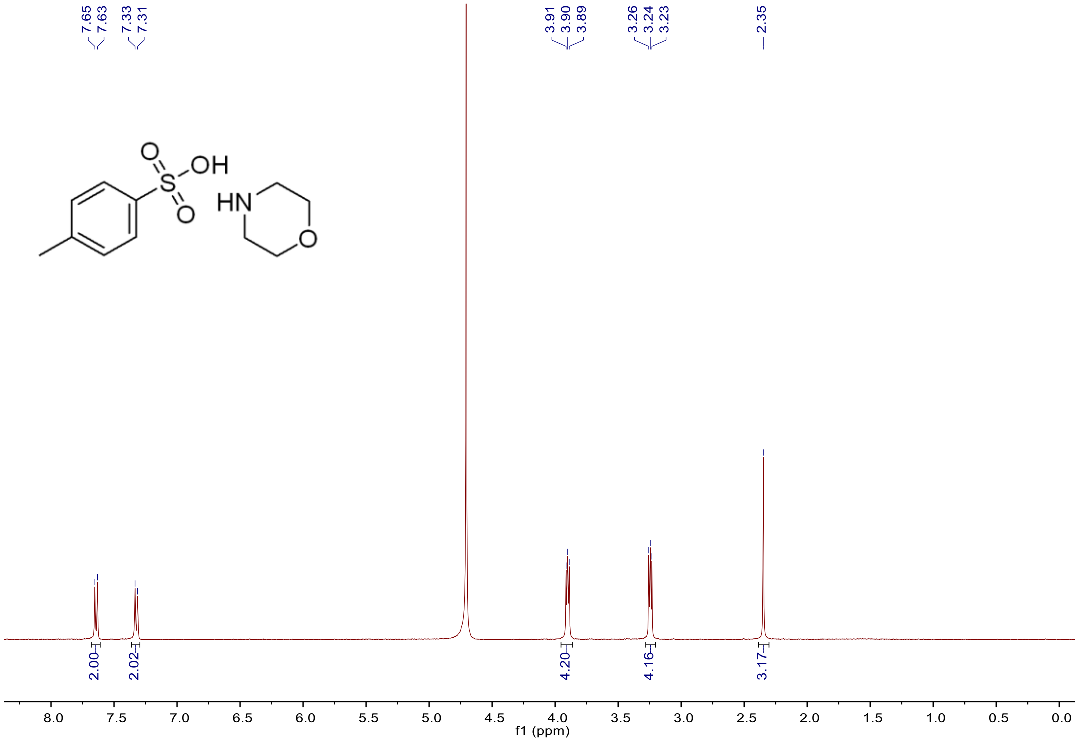

Supplementary Figure 32. ^{1}H NMR spectrum of **MPTS** (400 MHz, D_2O)

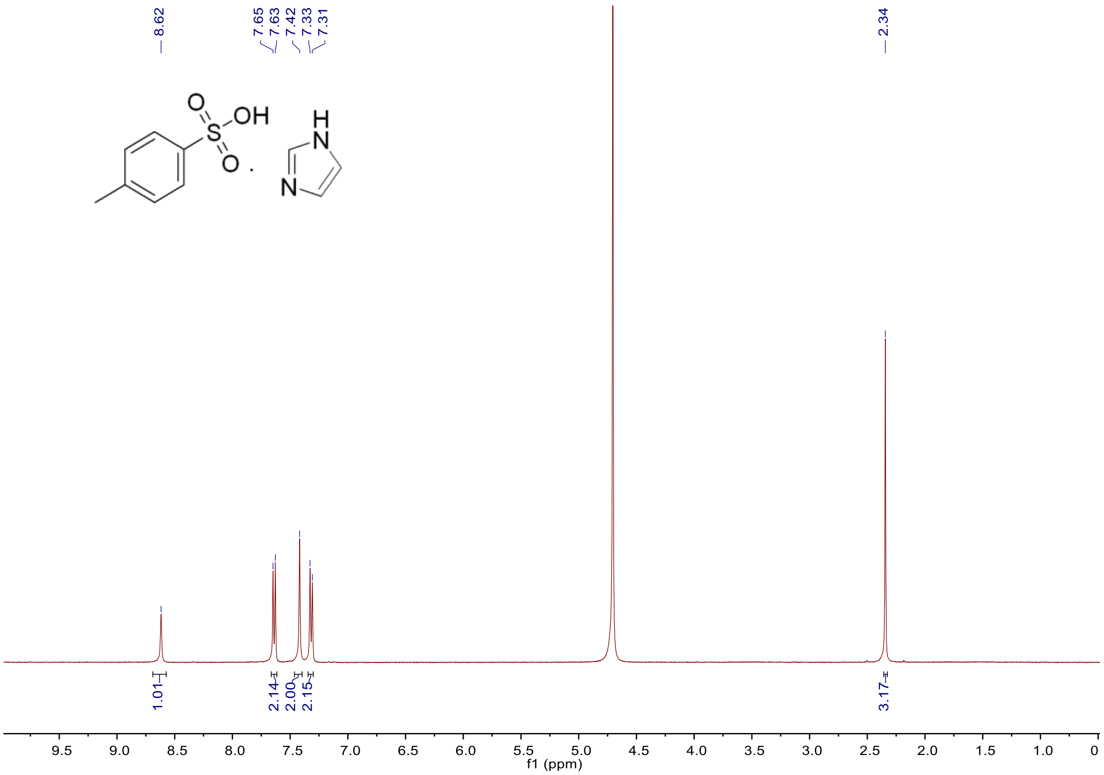

Supplementary Figure 33. ^{1}H NMR spectrum of **IPTS** (400 MHz, D_2O)

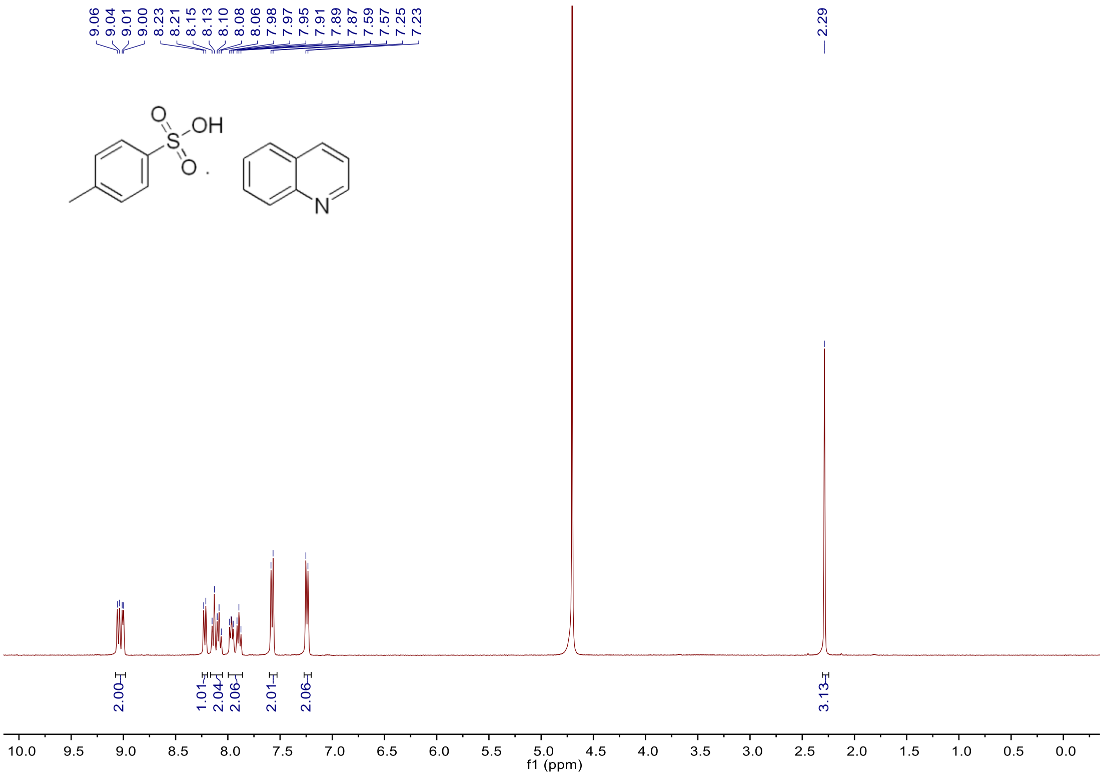

Supplementary Figure 34. ^{1}H NMR spectrum of **QPTS** (400 MHz, D_2O)

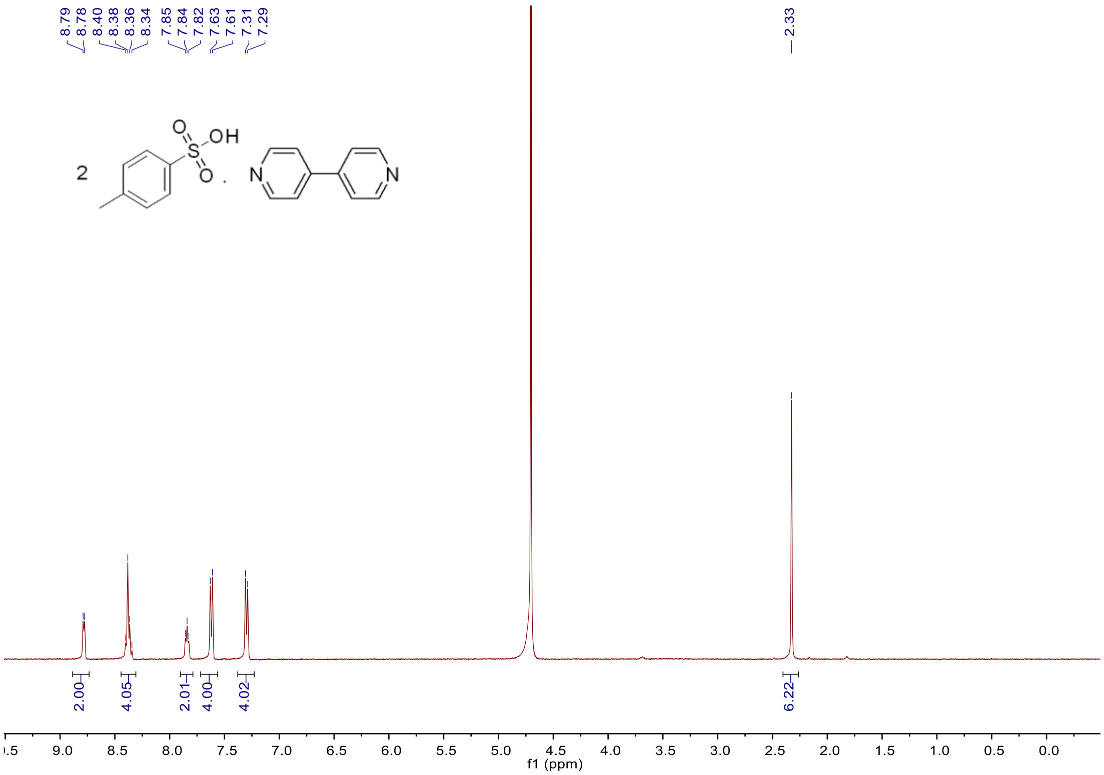

Supplementary Figure 35. ^{1}H NMR spectrum of **BPPTS** (400 MHz, D_2O)

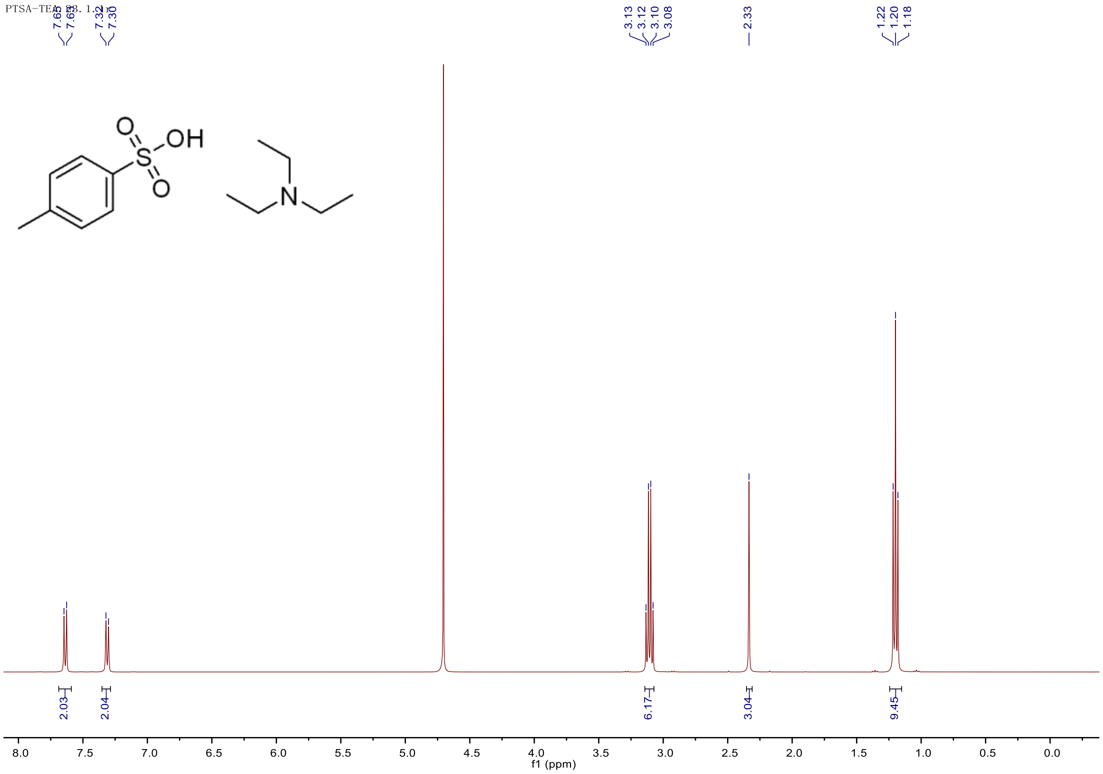

Supplementary Figure 36. ^{1}H NMR spectrum of **TPTS** (400 MHz, D_2O)

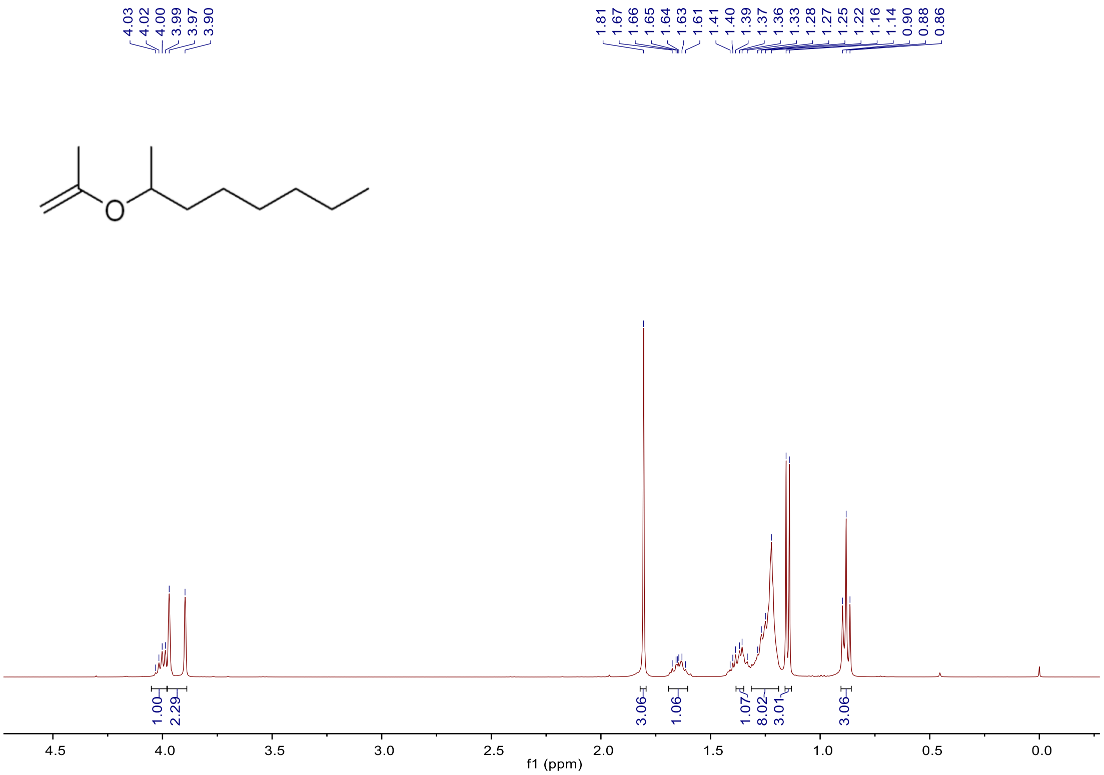

Supplementary Figure 37. ^{1}H NMR spectrum of compound **3a** (400 MHz, C_6D_6)

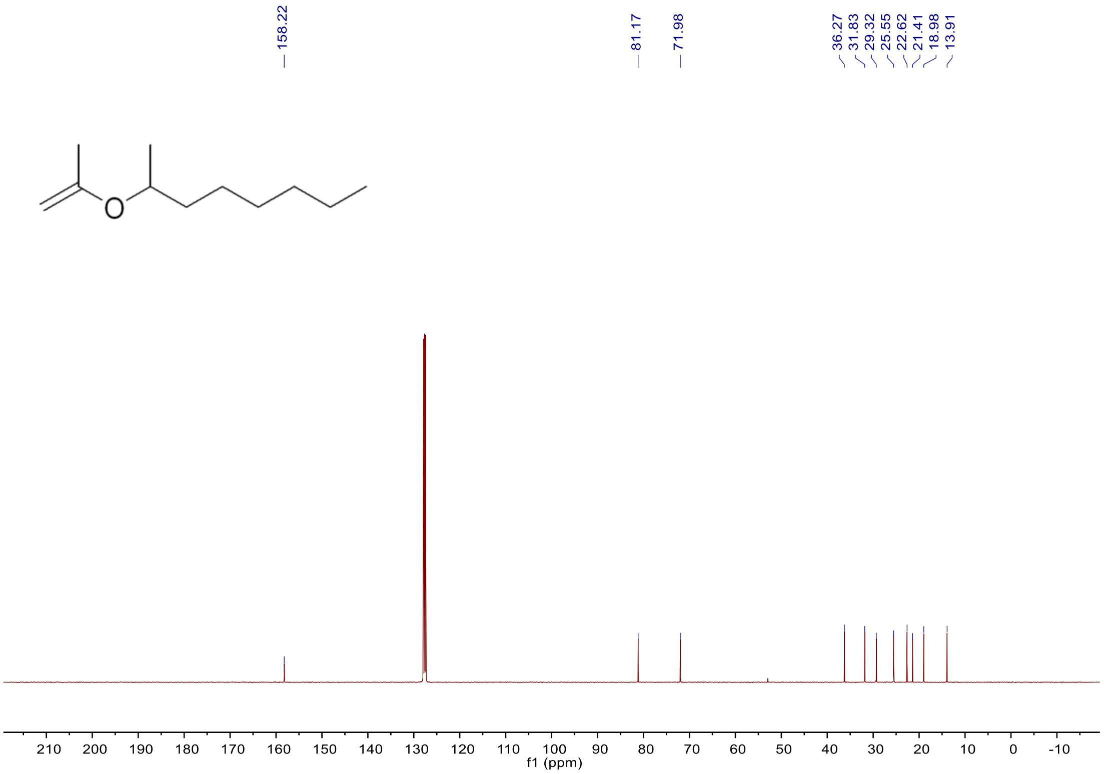

Supplementary Figure 38. ^{13}C NMR spectrum of compound **3a** (100 MHz, C_6D_6)

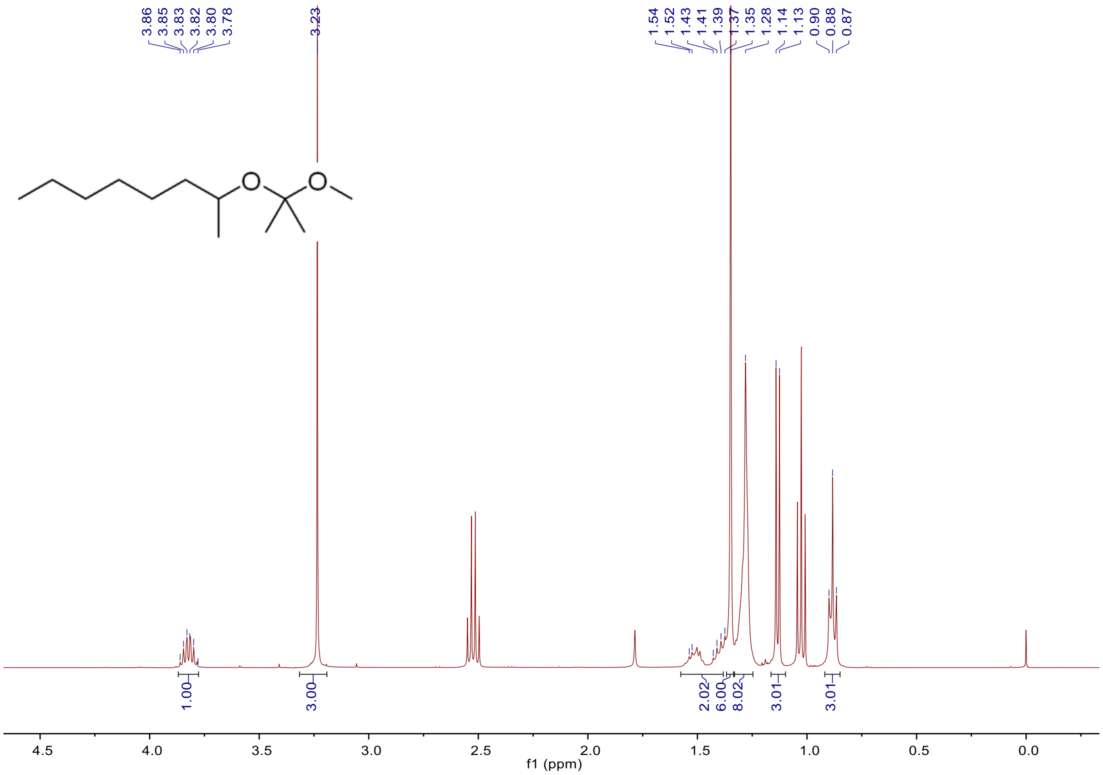

Supplementary Figure 39. ^{1}H NMR spectrum of **3a′** (400 MHz, $CDCl_3$)

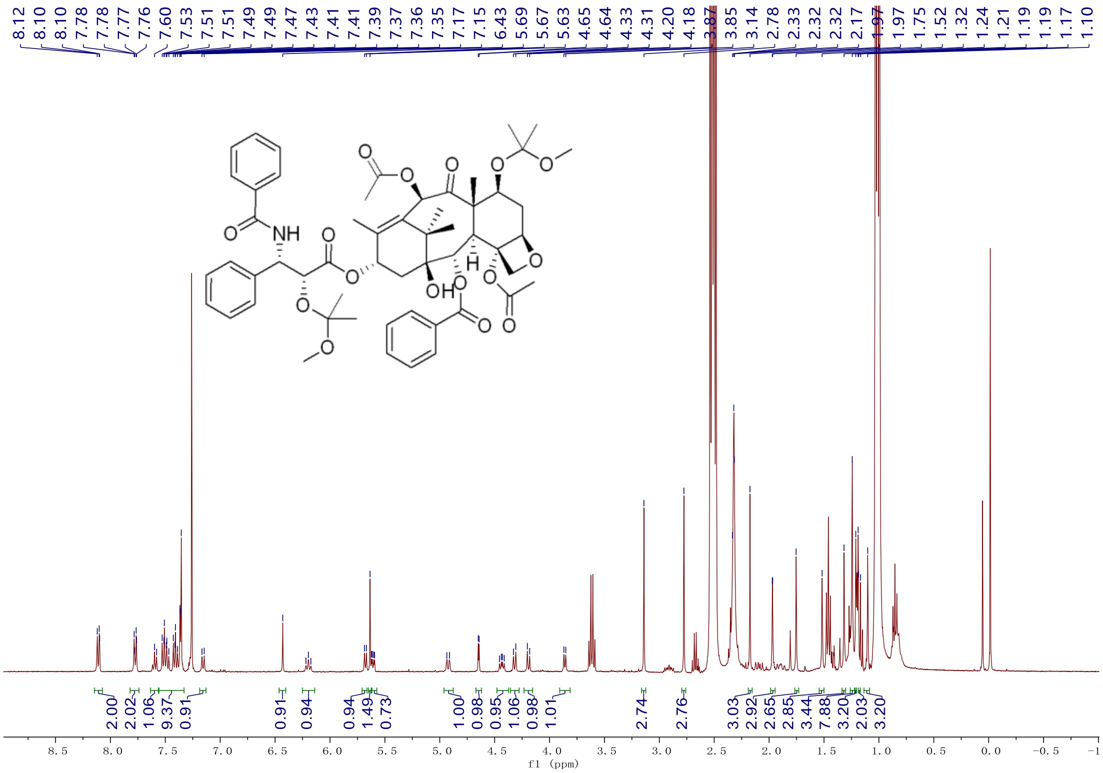

Supplementary Figure 40. ^{1}H NMR spectrum of compound **PTX-diMOP** (400 MHz, $CDCl_3$)

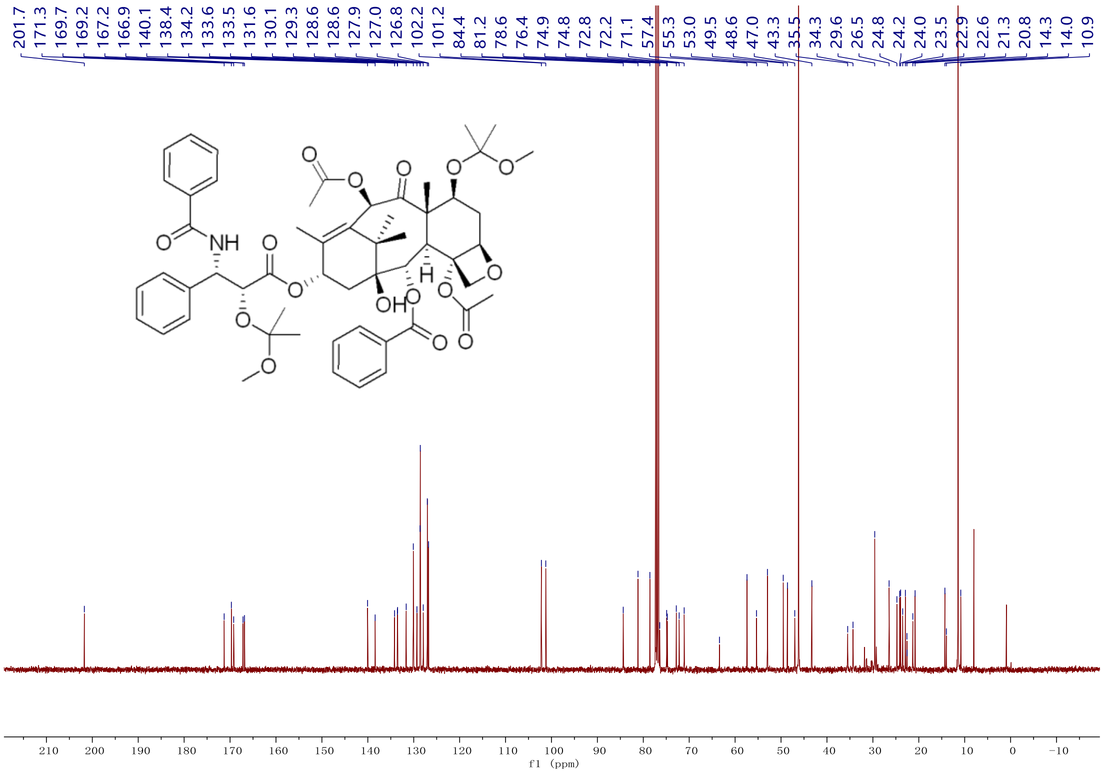

Supplementary Figure 41. ^{13}C NMR spectrum of compound **PTX-diMOP** (100 MHz, $CDCl_3$)

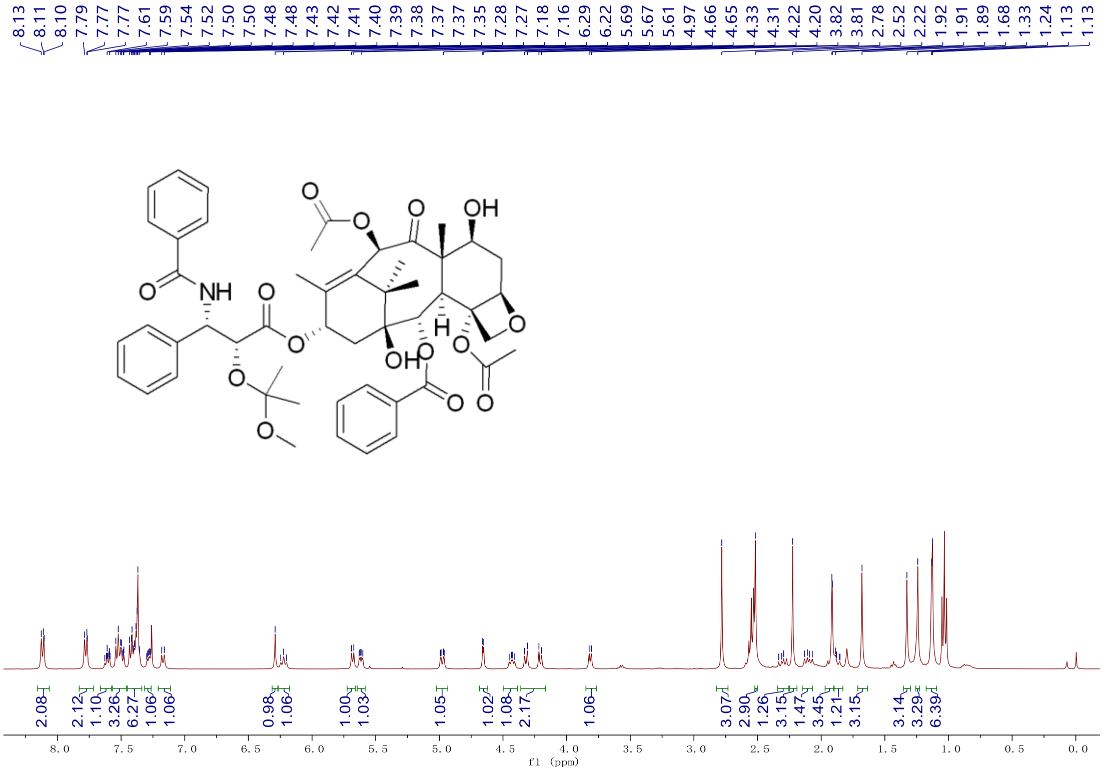

Supplementary Figure 42. ^{1}H NMR spectrum of compound **PTX-2′-MOP** (400 MHz, $CDCl_3$)

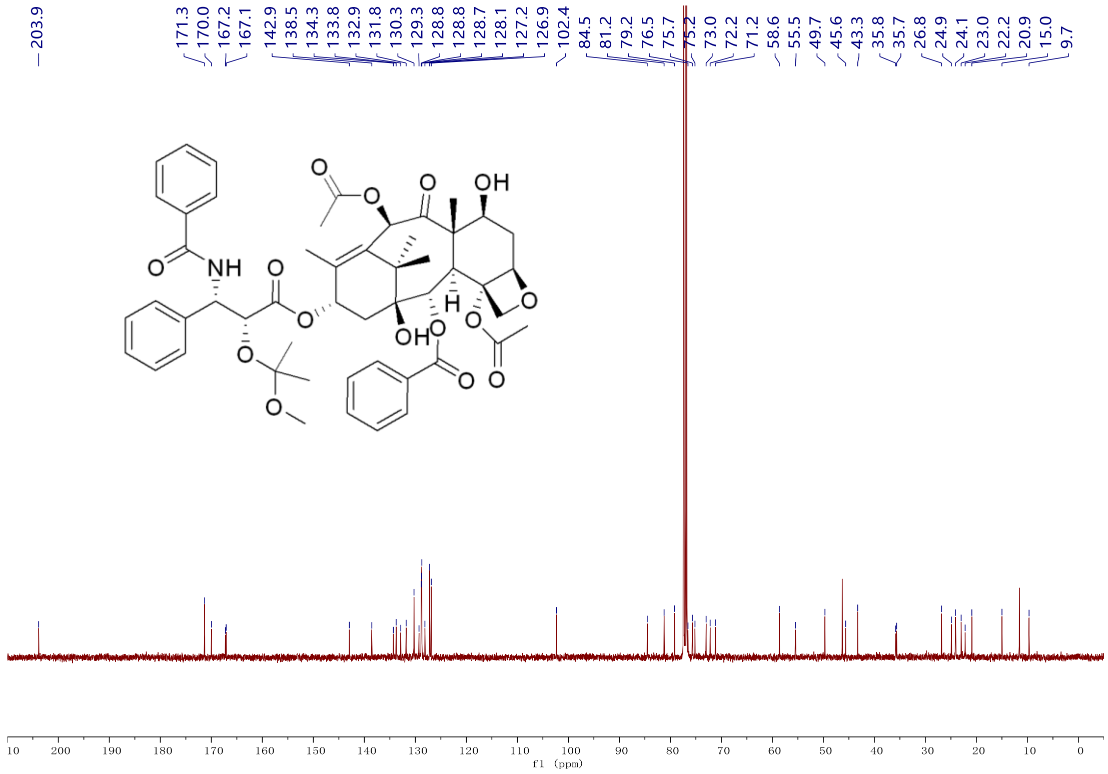

Supplementary Figure 43. ^{13}C NMR spectrum of compound **PTX-2′-MOP** (100 MHz, $CDCl_3$)

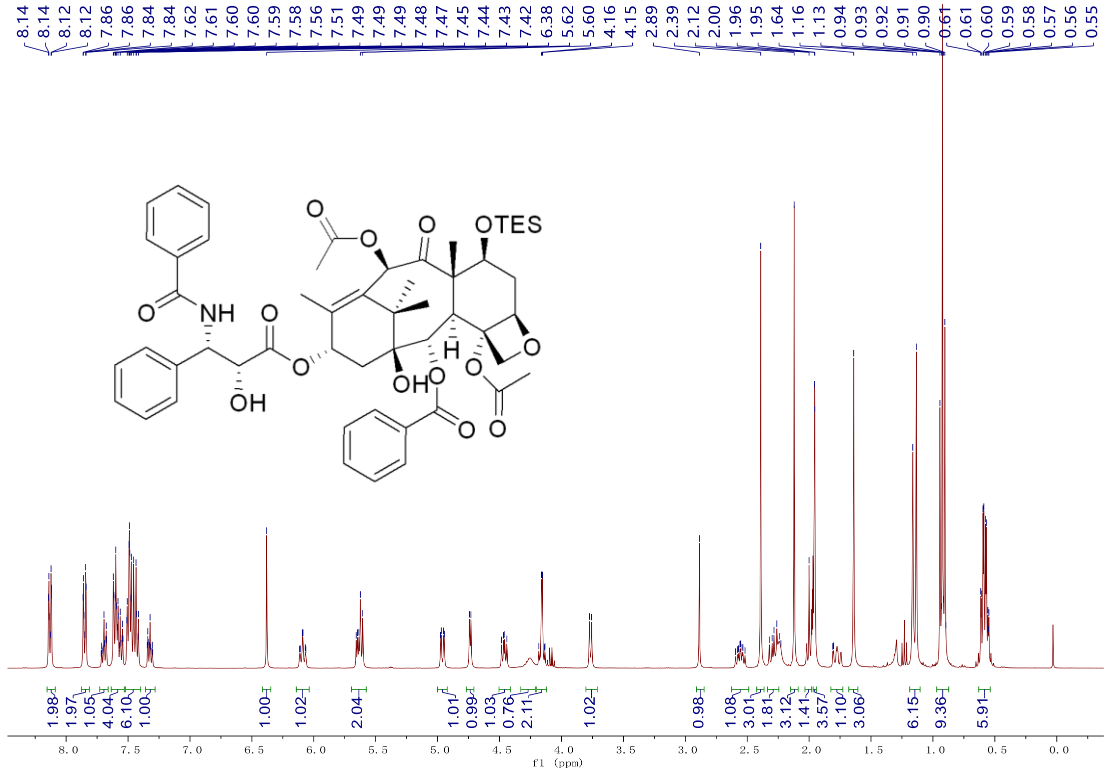

Supplementary Figure 44. ^{1}H NMR spectrum of compound **PTX-7-TES** (400 MHz, CD_3CN)

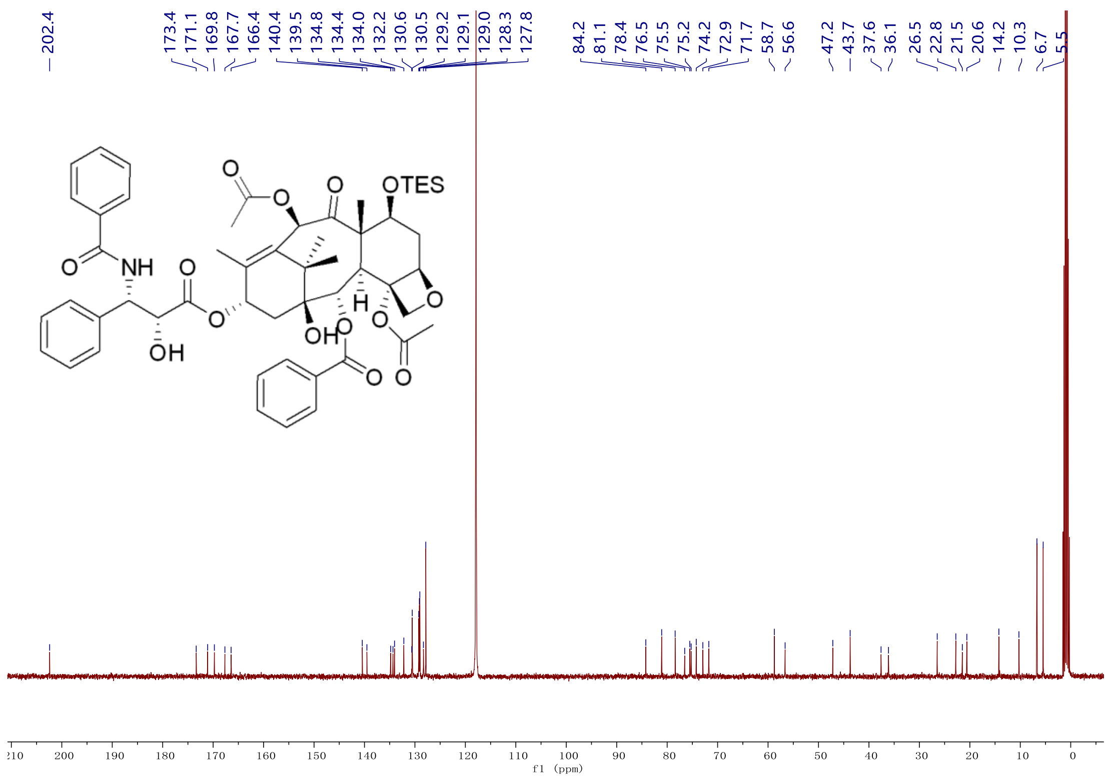

Supplementary Figure 45. ^{13}C NMR spectrum of compound **PTX-7-TES** (100 MHz, CD_3CN)

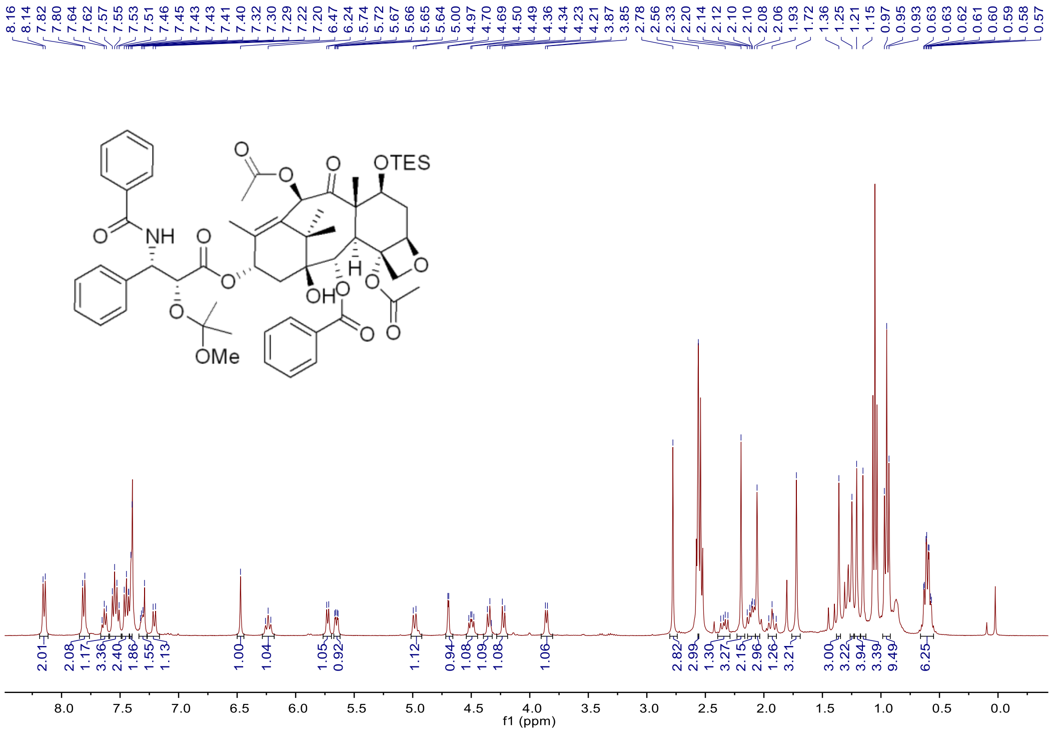

Supplementary Figure 46. ^{1}H NMR spectrum of compound **PTX-2′-MOP-7-TES** (400 MHz, $CDCl_3$)

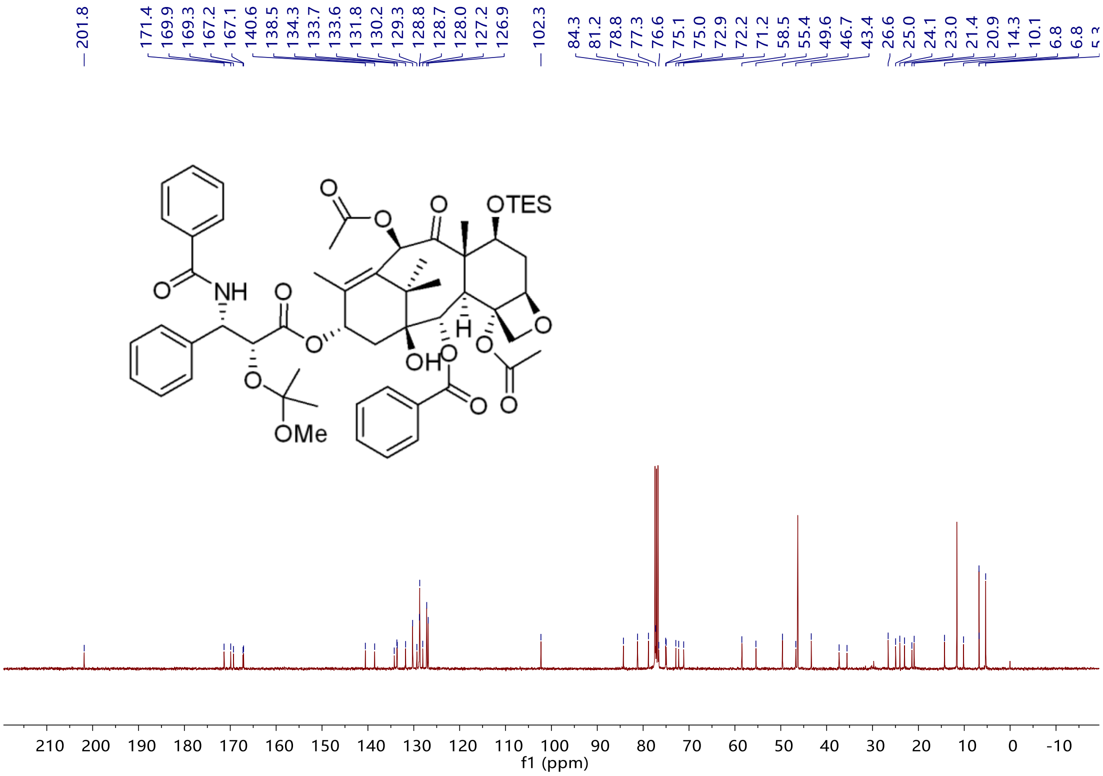

Supplementary Figure 47. ^{13}C NMR spectrum of compound **PTX-2′-MOP-7-TES** (100 MHz, $CDCl_3$)

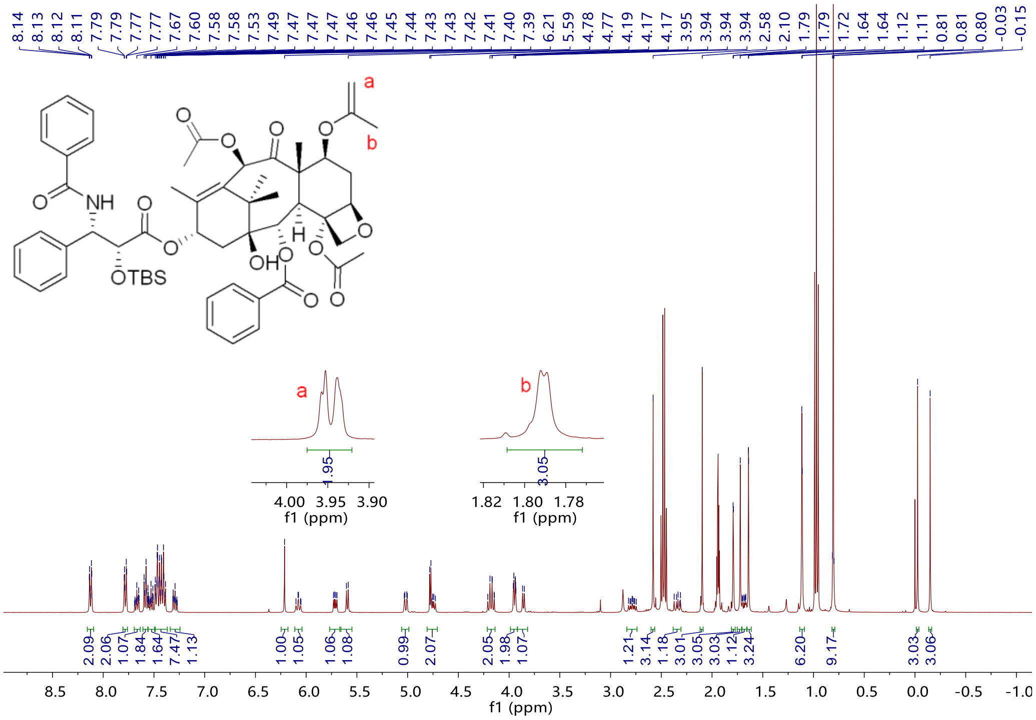

Supplementary Figure 48. ^{1}H NMR spectrum of compound **3b** (400 MHz, CD_3CN)

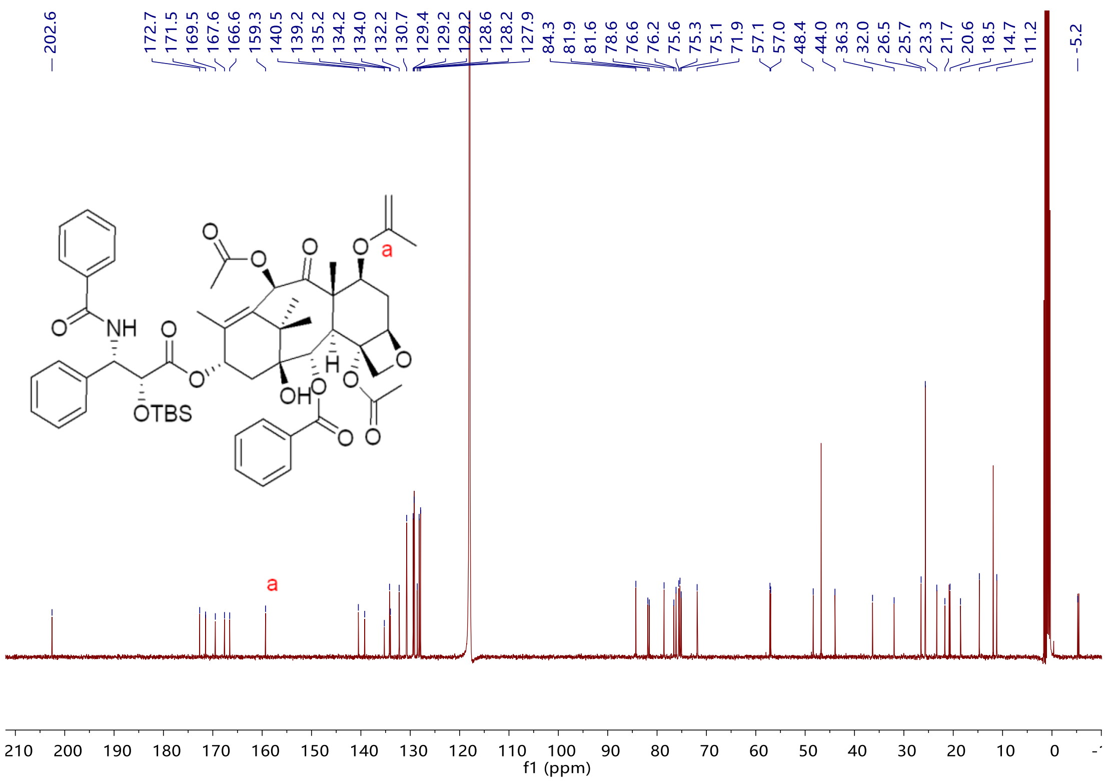

Supplementary Figure 49. ^{13}C NMR spectrum of compound **3b** (100 MHz, CD_3CN)

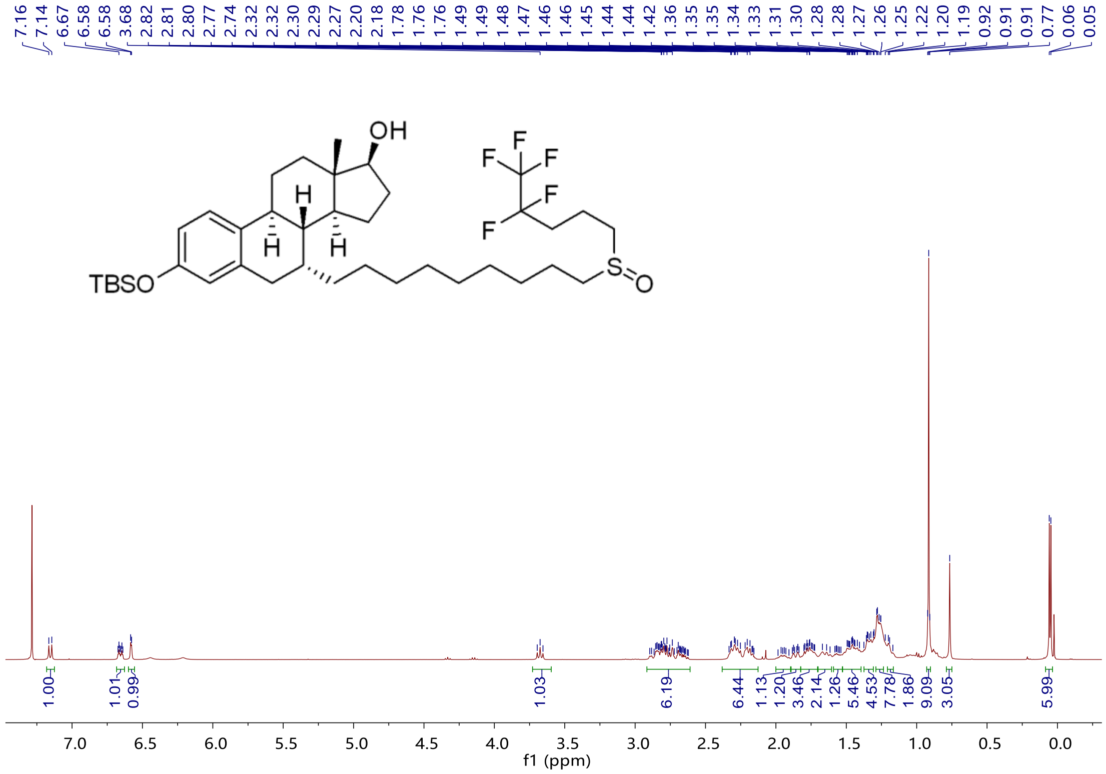

Supplementary Figure 50. ^{1}H NMR spectrum of compound **FUL-3-TBS** (400 MHz, $CDCl_3$)

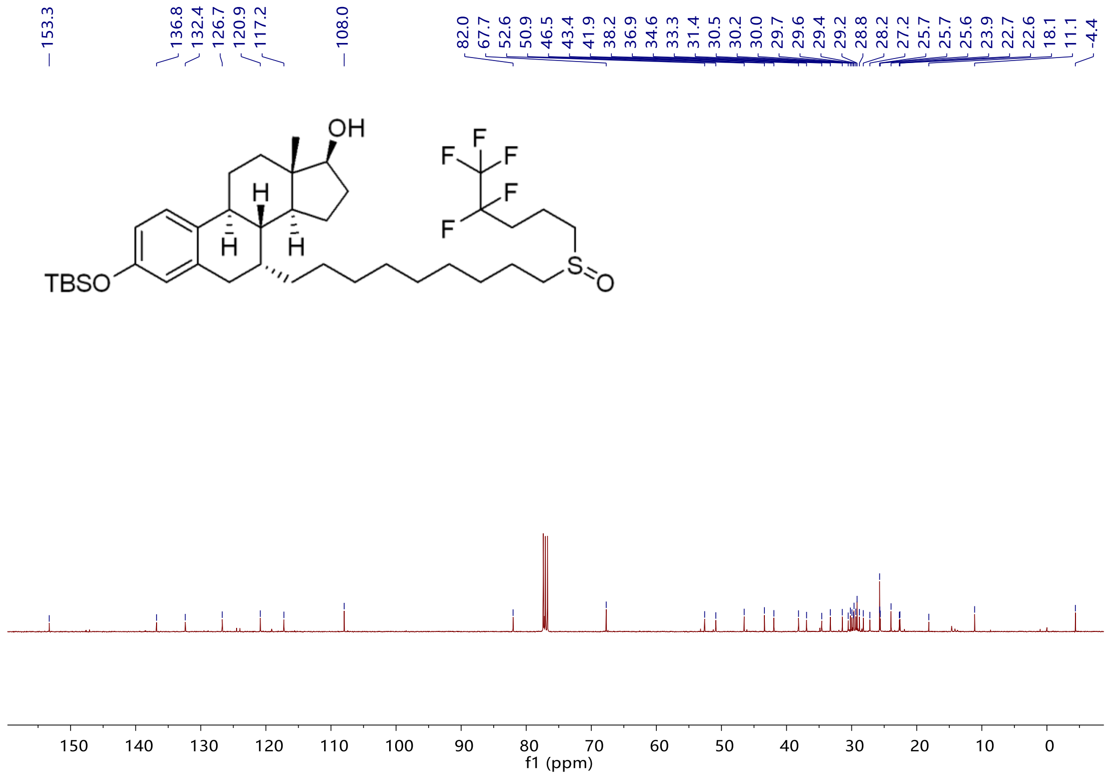

Supplementary Figure 51. ^{13}C NMR spectrum of compound **FUL-3-TBS** (100 MHz, $CDCl_3$)

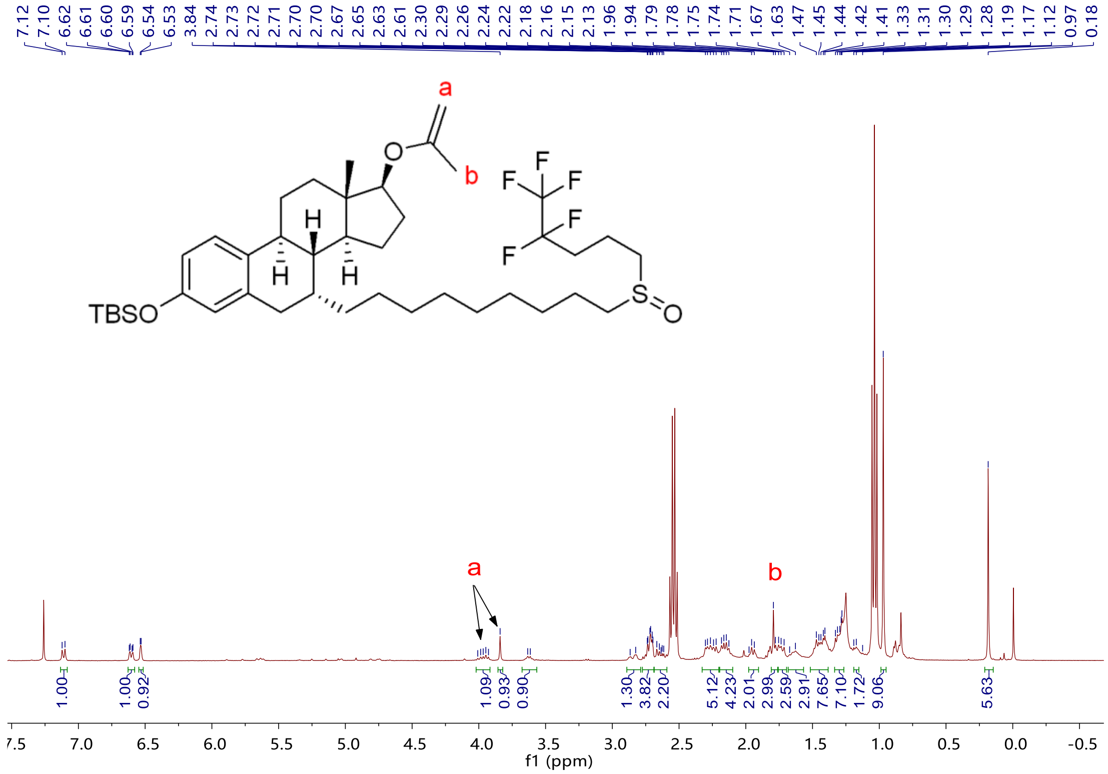

Supplementary Figure 52. ^{1}H NMR spectrum of compound **3c** (400 MHz, $CDCl_3$)

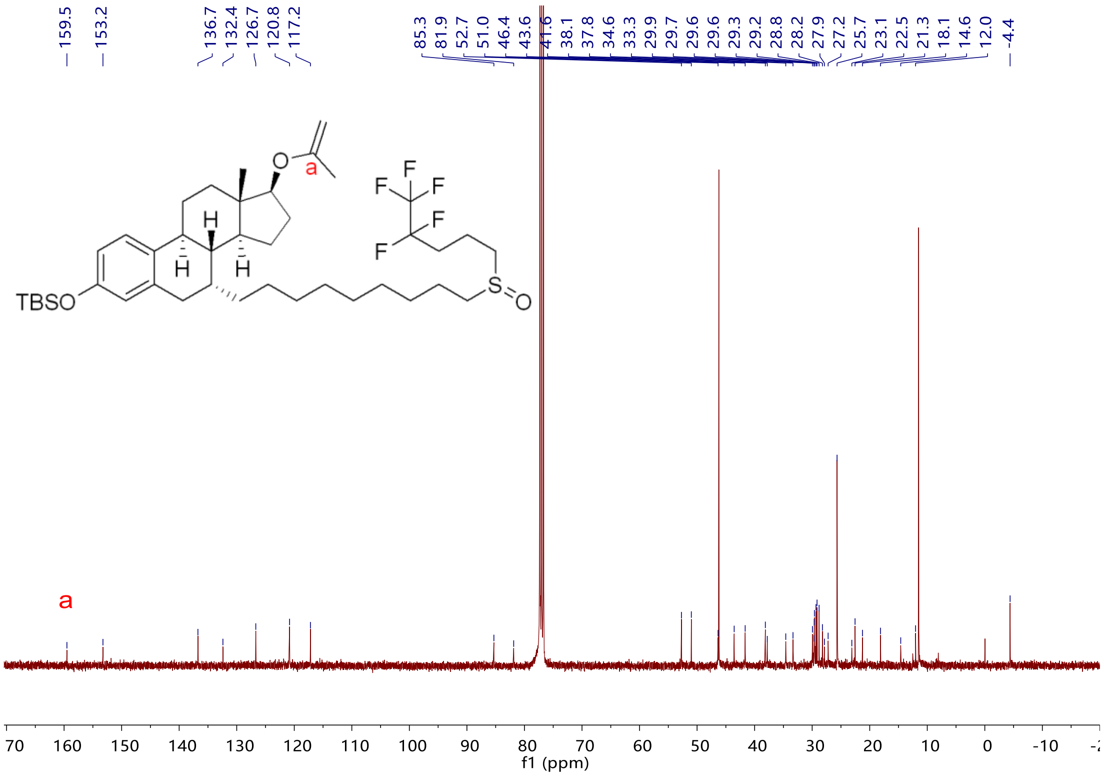

Supplementary Figure 53. ^{13}C NMR spectrum of compound **3c** (100 MHz, $CDCl_3$)

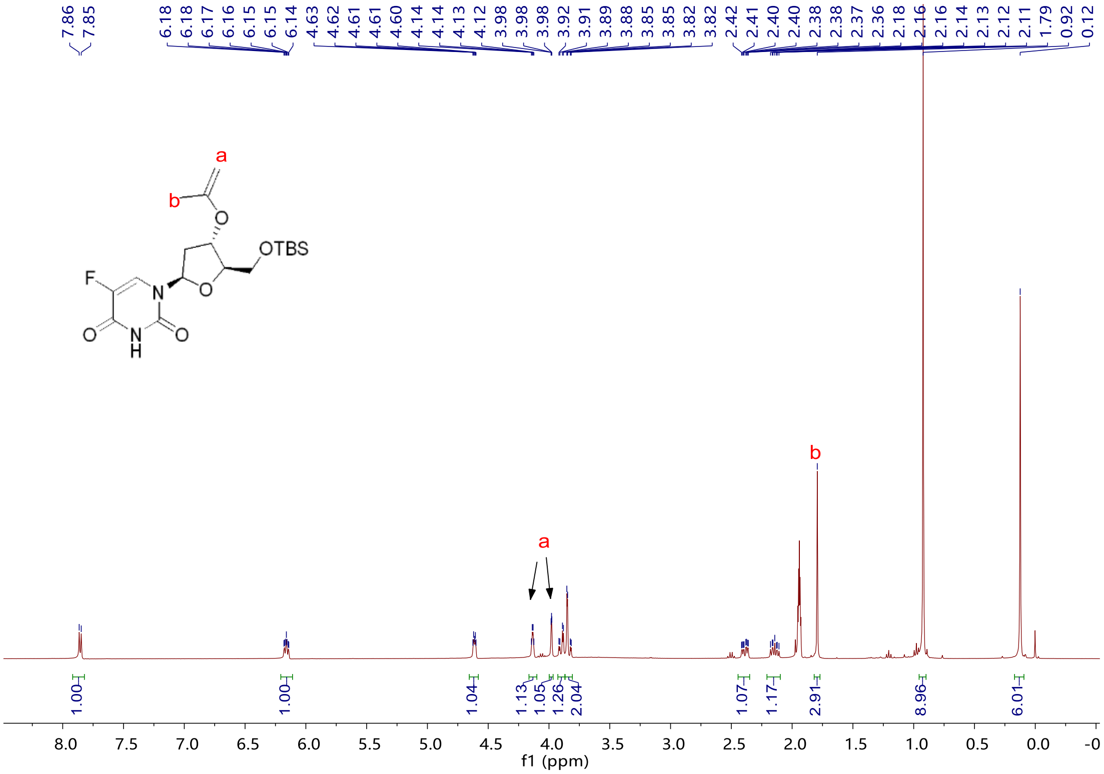

Supplementary Figure 54. ^{1}H NMR spectrum of compound **3d** (400 MHz, CD_3CN)

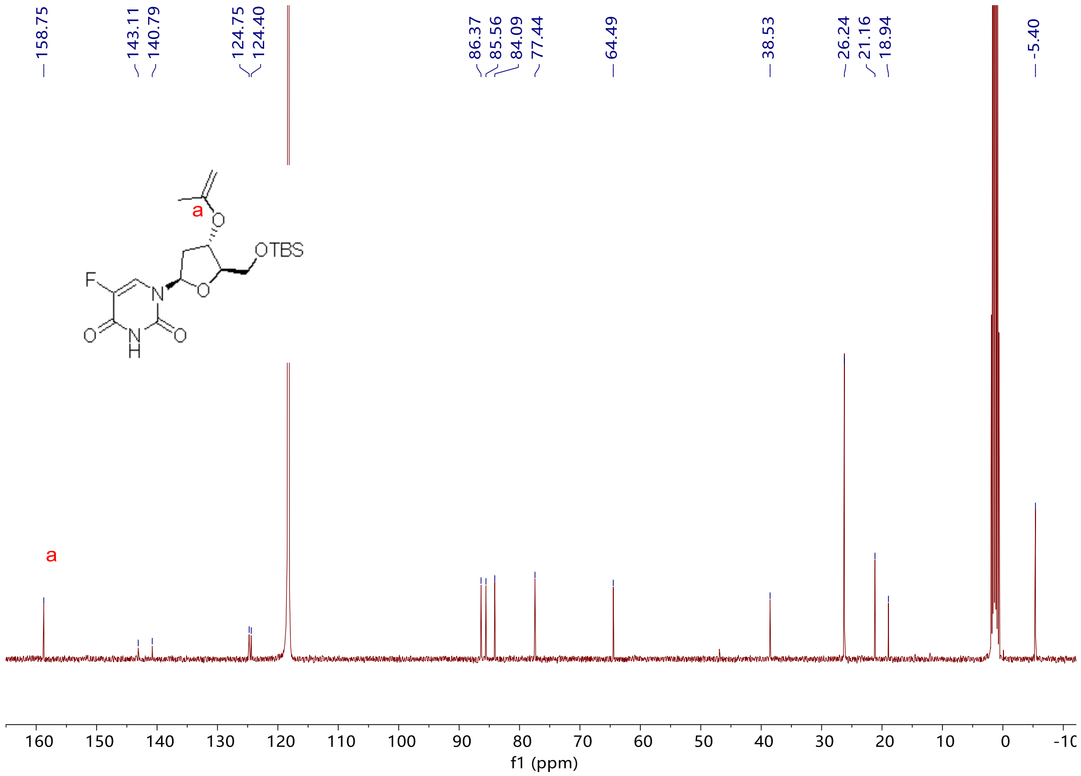

Supplementary Figure 55. ^{13}C NMR spectrum of compound **3d** (100 MHz, CD_3CN)

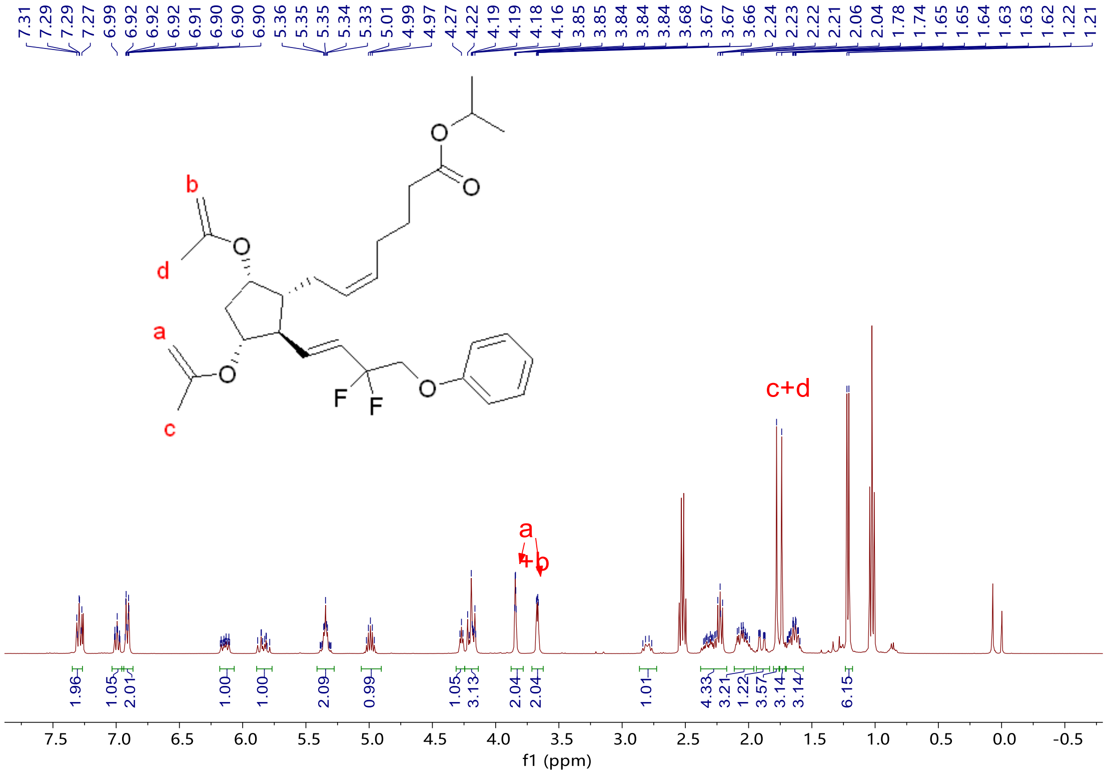

Supplementary Figure 56. ^{1}H NMR spectrum of compound **3e** (400 MHz, $CDCl_3$)

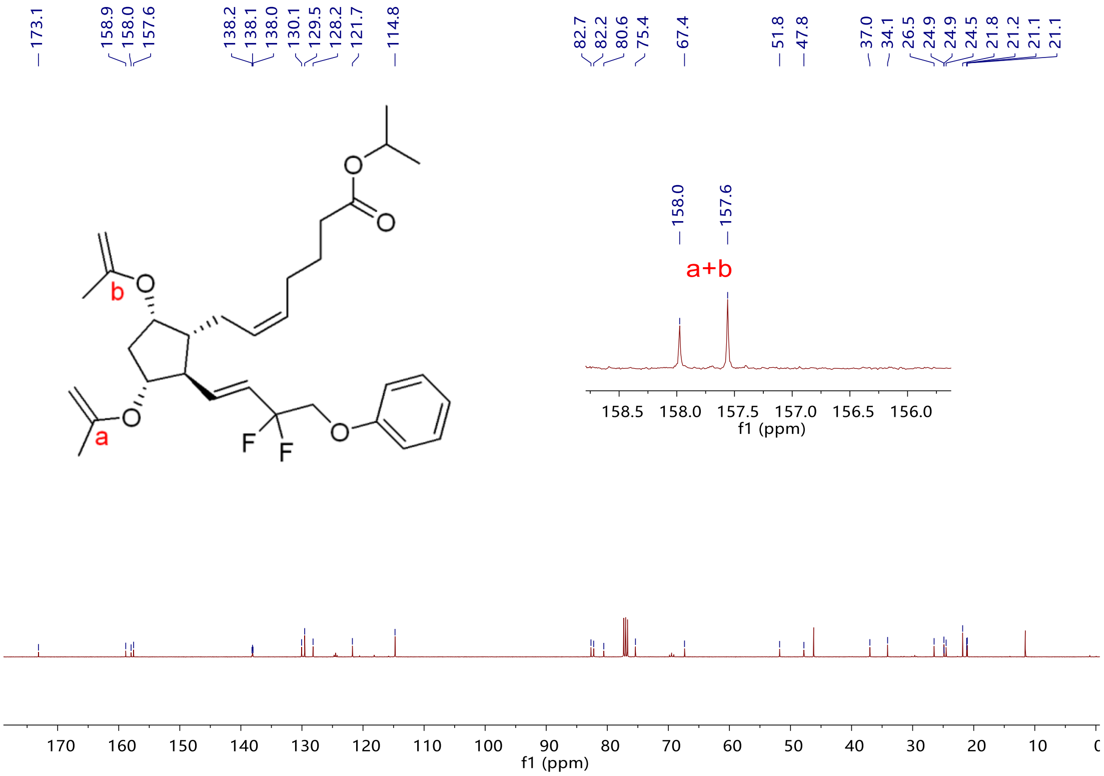

Supplementary Figure 57. ^{13}C NMR spectrum of compound **3e** (100 MHz, $CDCl_3$)

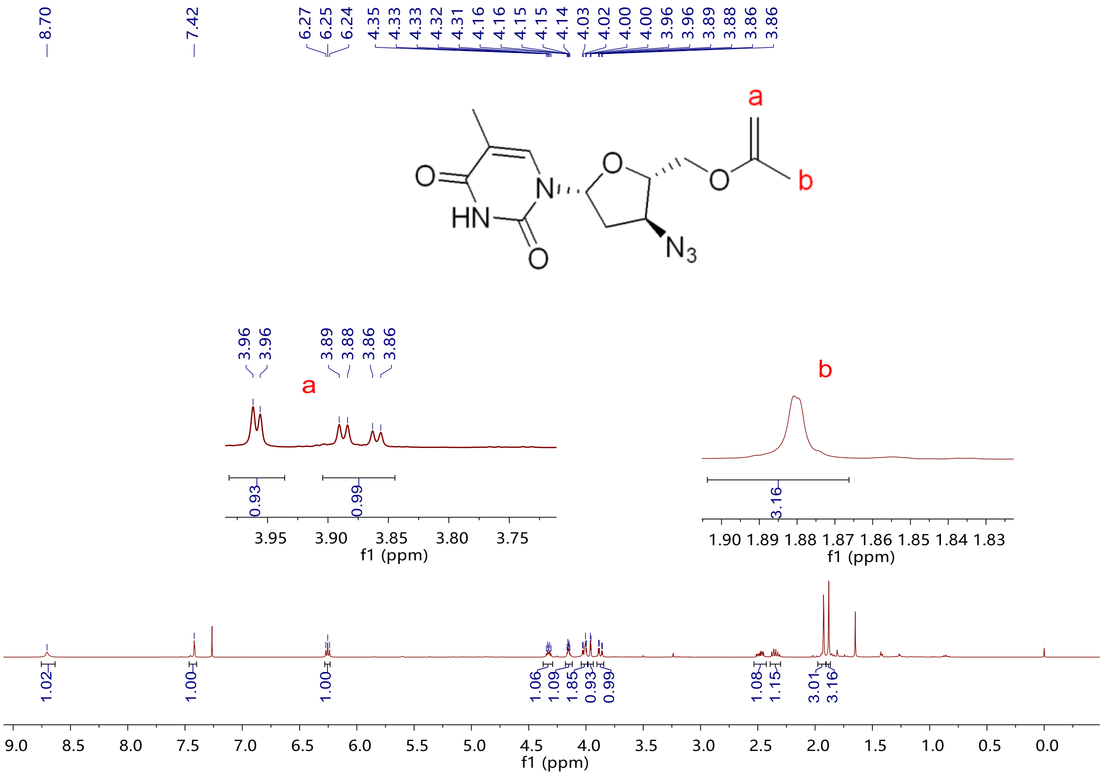

Supplementary Figure 58. ^{1}H NMR spectrum of compound **3f** (400 MHz, $CDCl_3$)

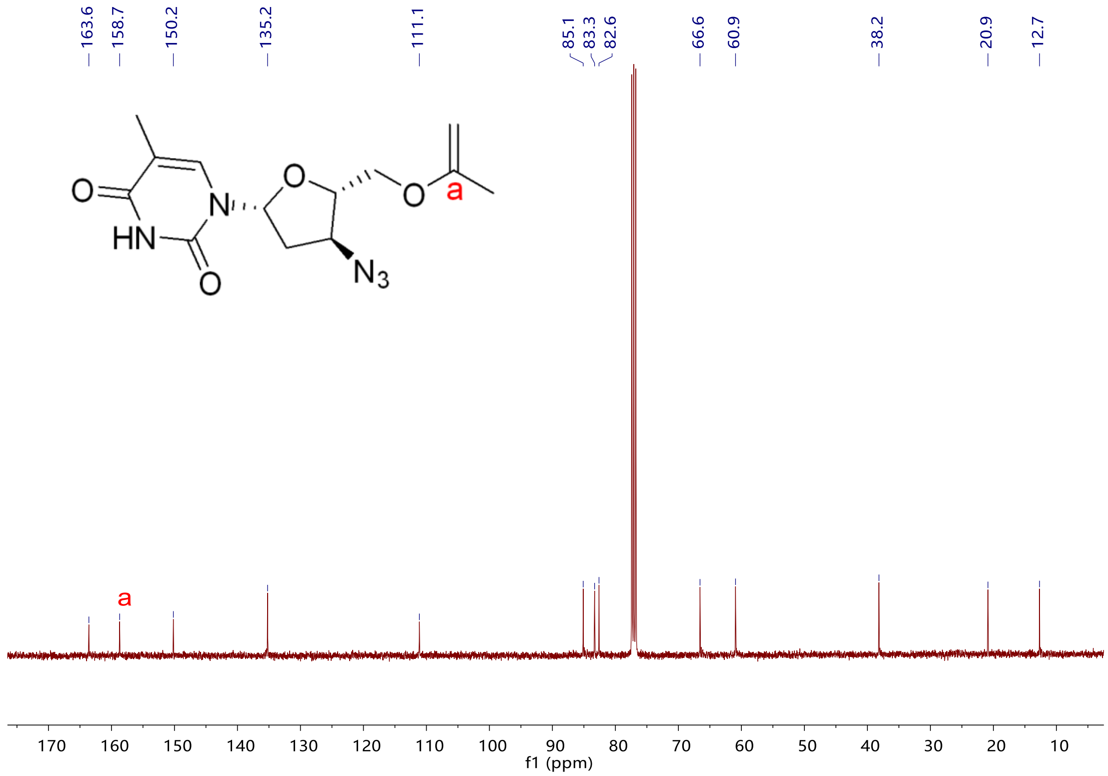

Supplementary Figure 59. ^{13}C NMR spectrum of compound **3f** (100 MHz, $CDCl_3$)

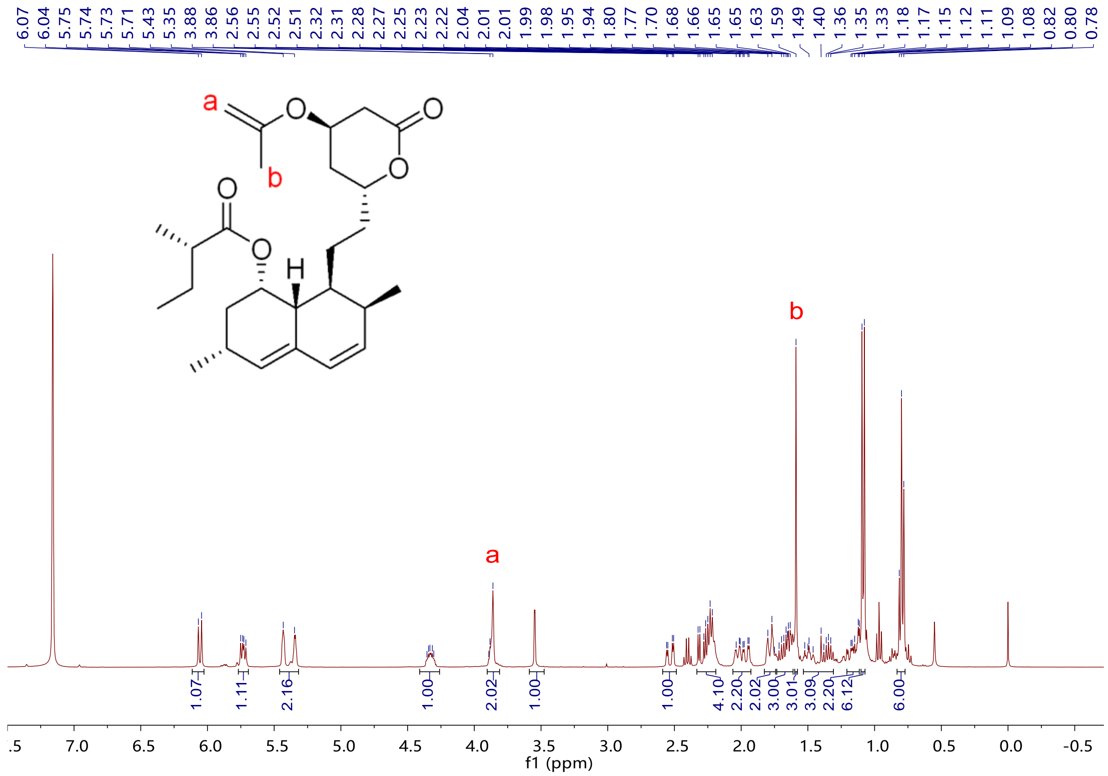

Supplementary Figure 60. ^{1}H NMR spectrum of compound **3g** (400 MHz, C_6D_6)

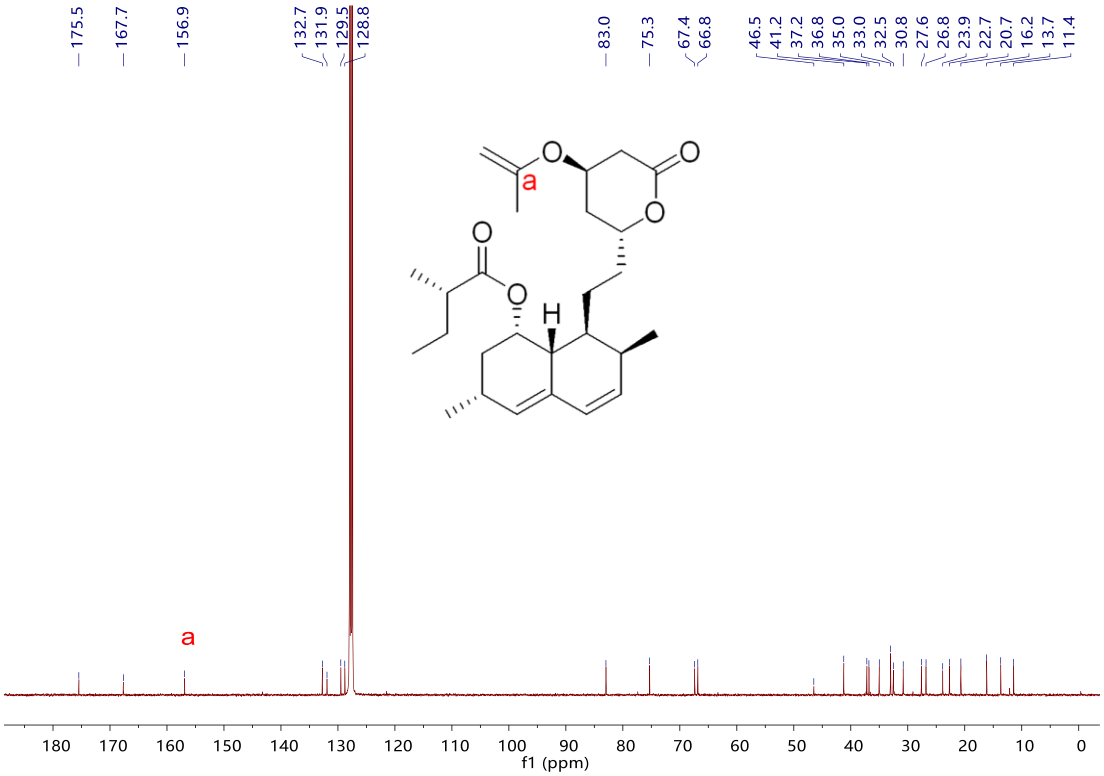

Supplementary Figure 61. ^{13}C NMR spectrum of compound **3g** (100 MHz, C_6D_6)

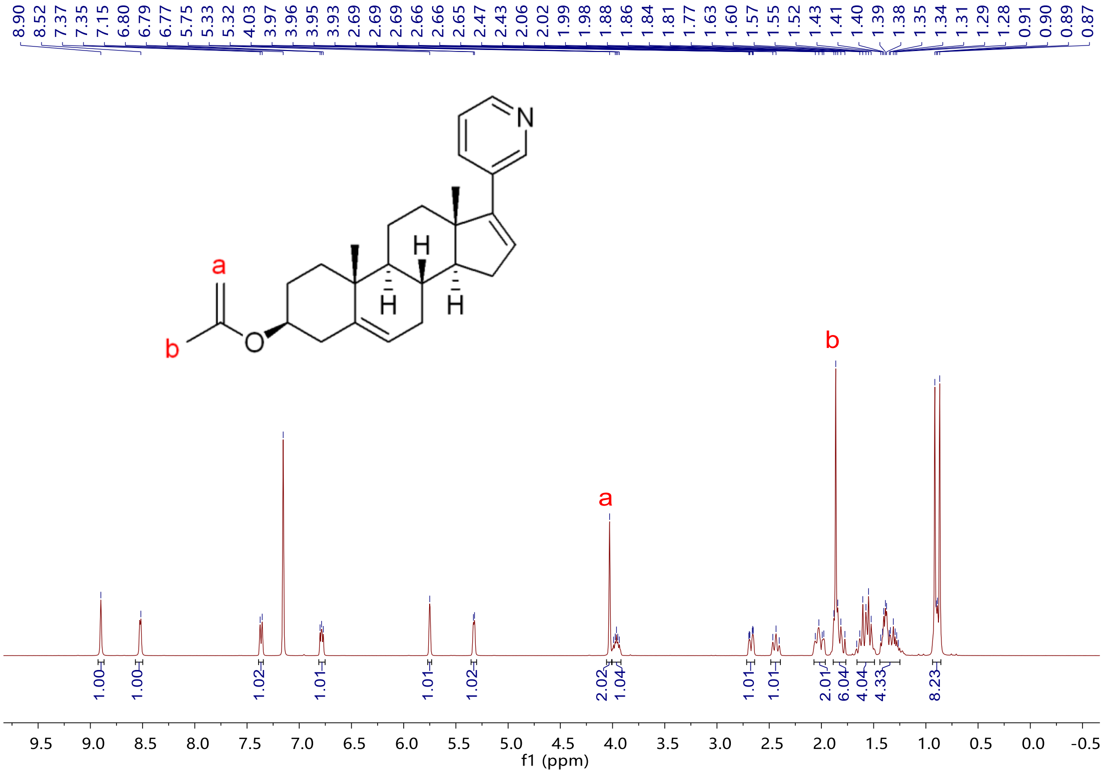

Supplementary Figure 62. ^{1}H NMR spectrum of compound **3h** (400 MHz, C_6D_6)

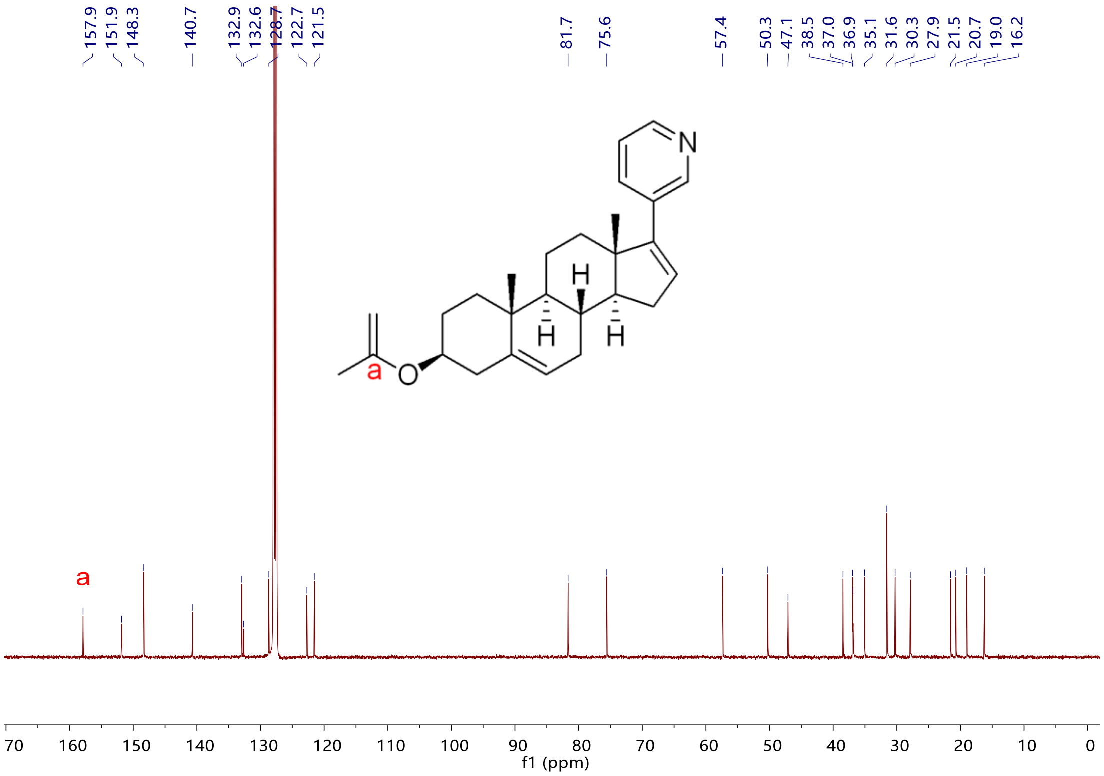

Supplementary Figure 63. ^{13}C NMR spectrum of compound **3h** (100 MHz, C_6D_6)

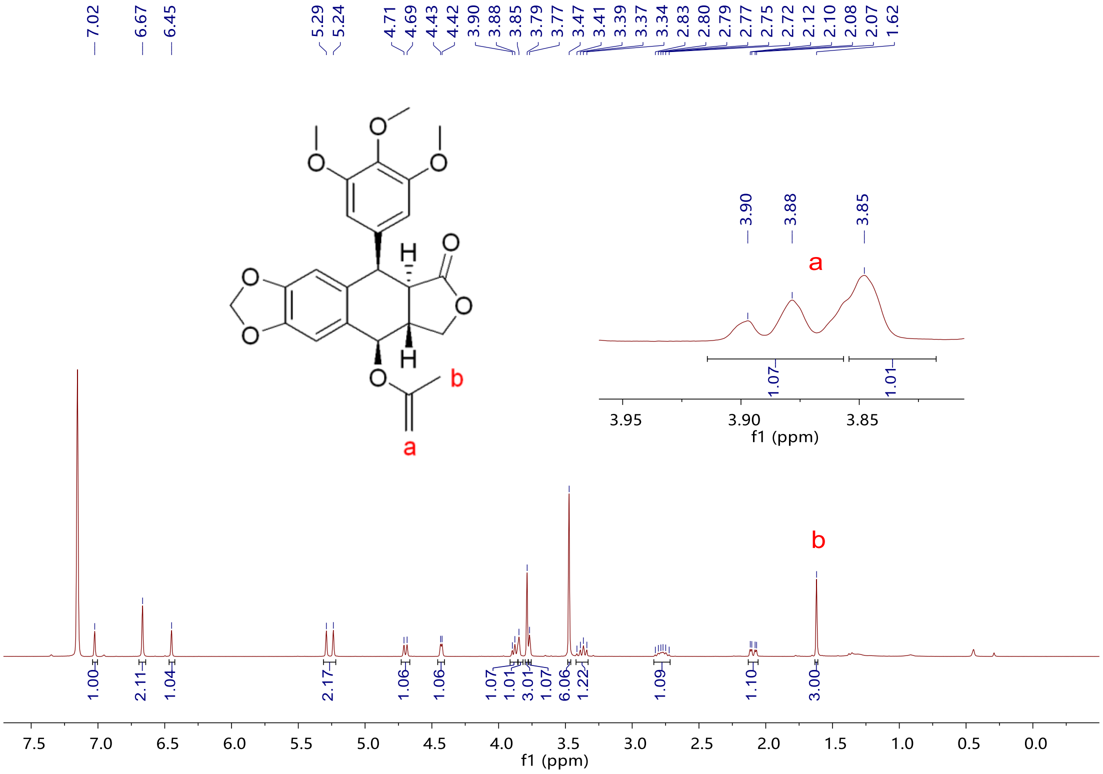

Supplementary Figure 64. ^{1}H NMR spectrum of compound **3i** (400 MHz, C_6D_6)

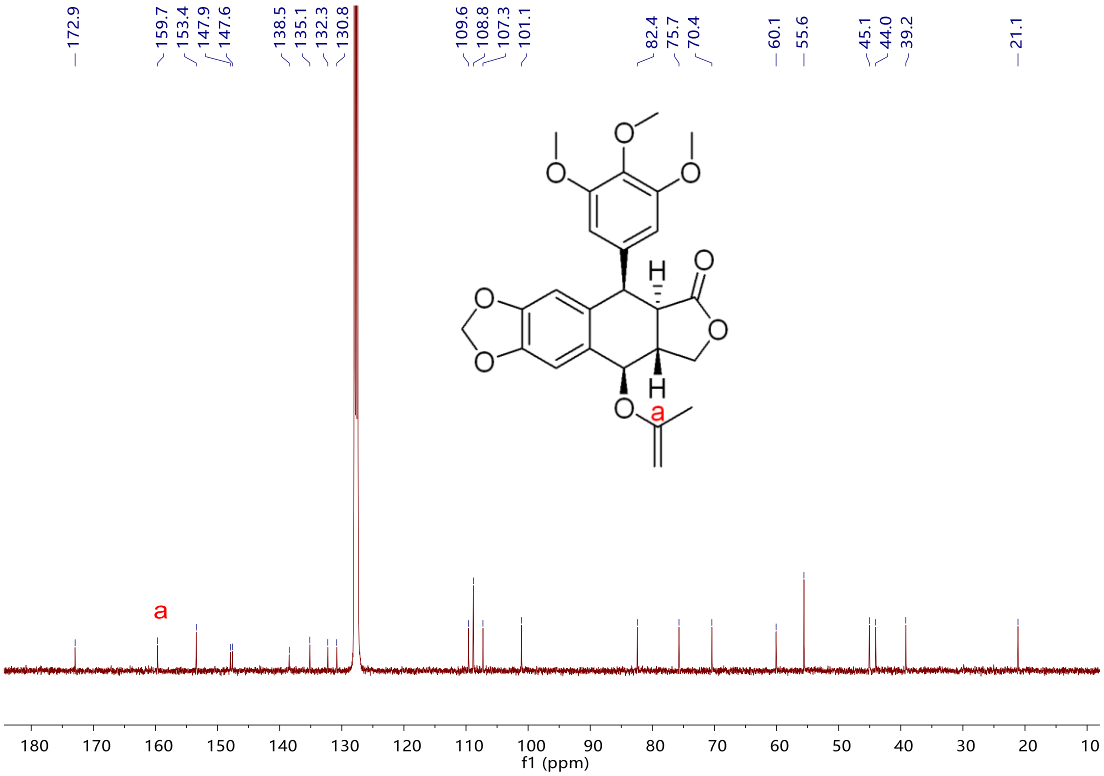

Supplementary Figure 65. ^{13}C NMR spectrum of compound **3i** (100 MHz, C_6D_6)

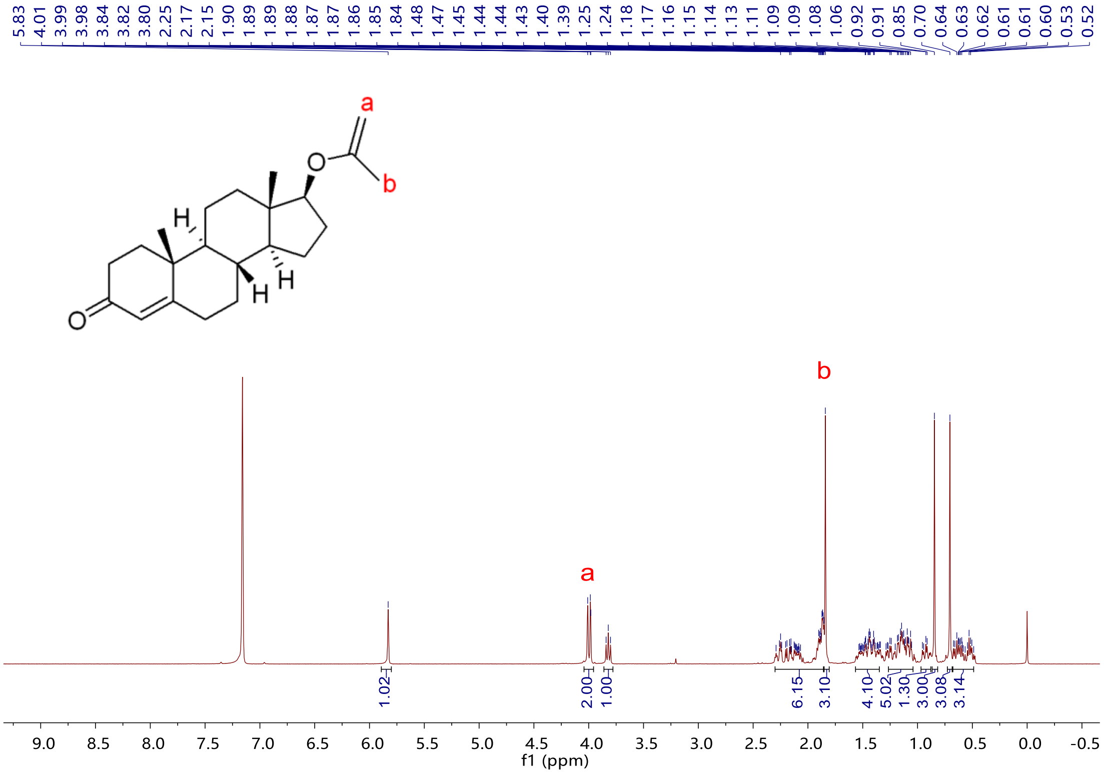

Supplementary Figure 66. ^{1}H NMR spectrum of compound **3j** (400 MHz, C_6D_6)

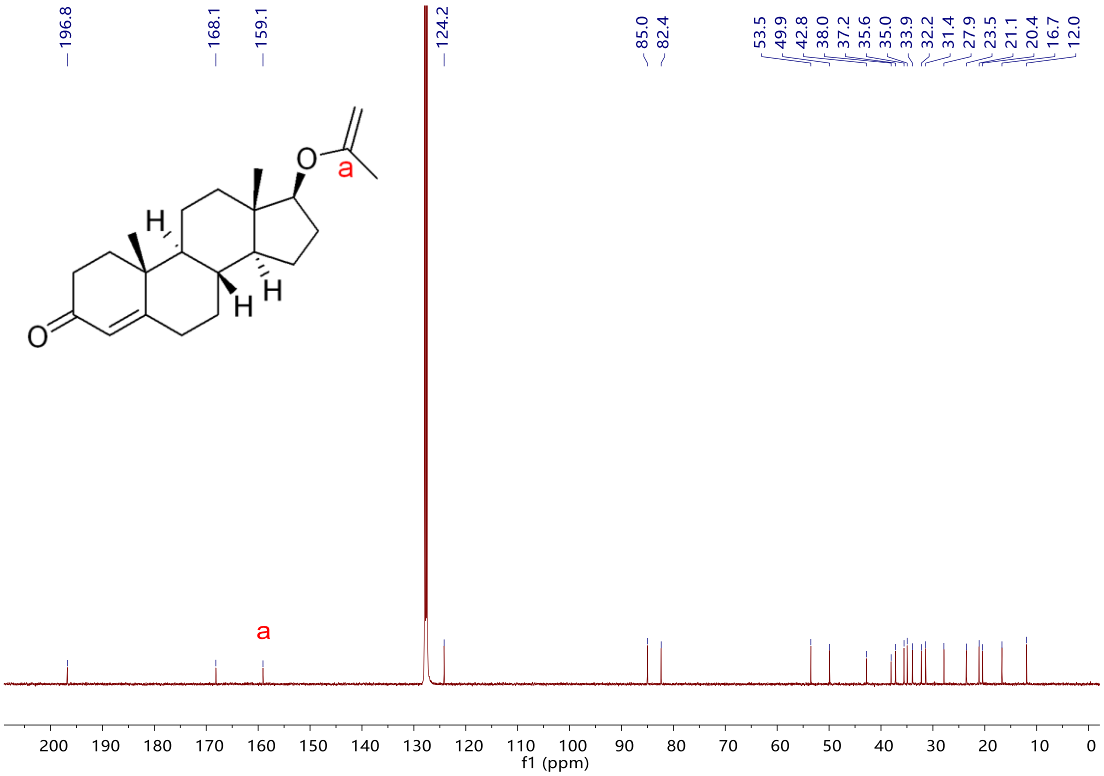

Supplementary Figure 67. ^{13}C NMR spectrum of compound **3j** (100 MHz, C_6D_6)

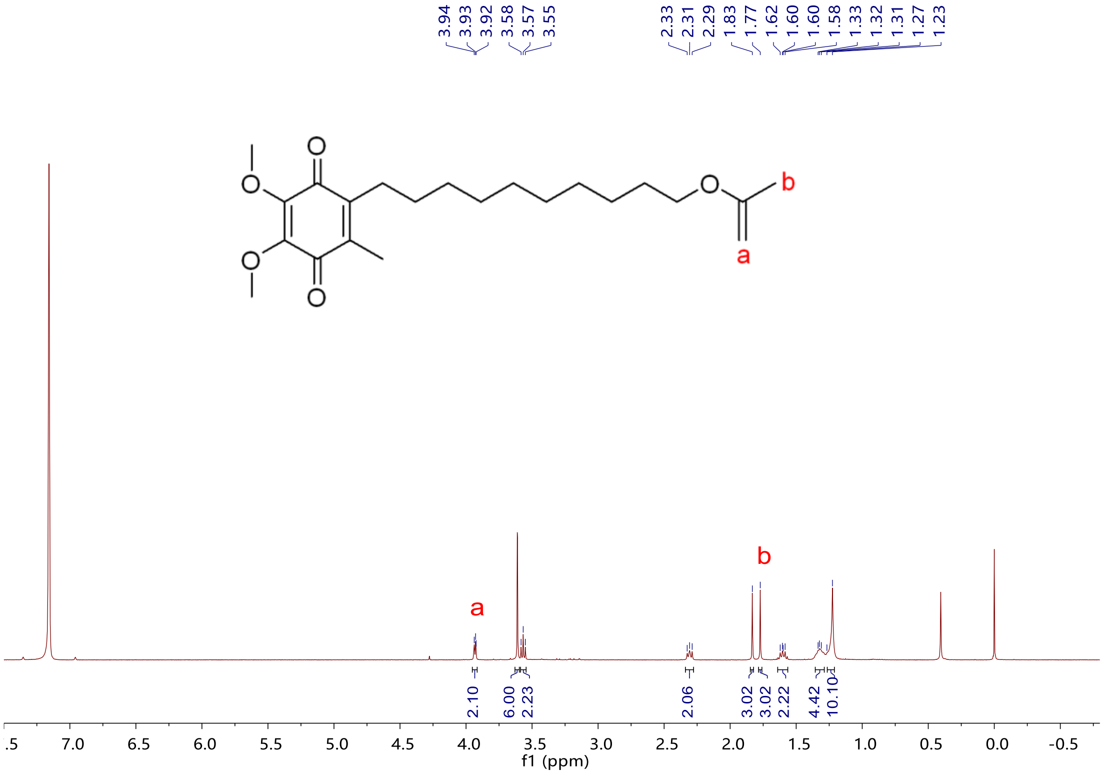

Supplementary Figure 68. ^{1}H NMR spectrum of compound **3k** (400 MHz, C_6D_6)

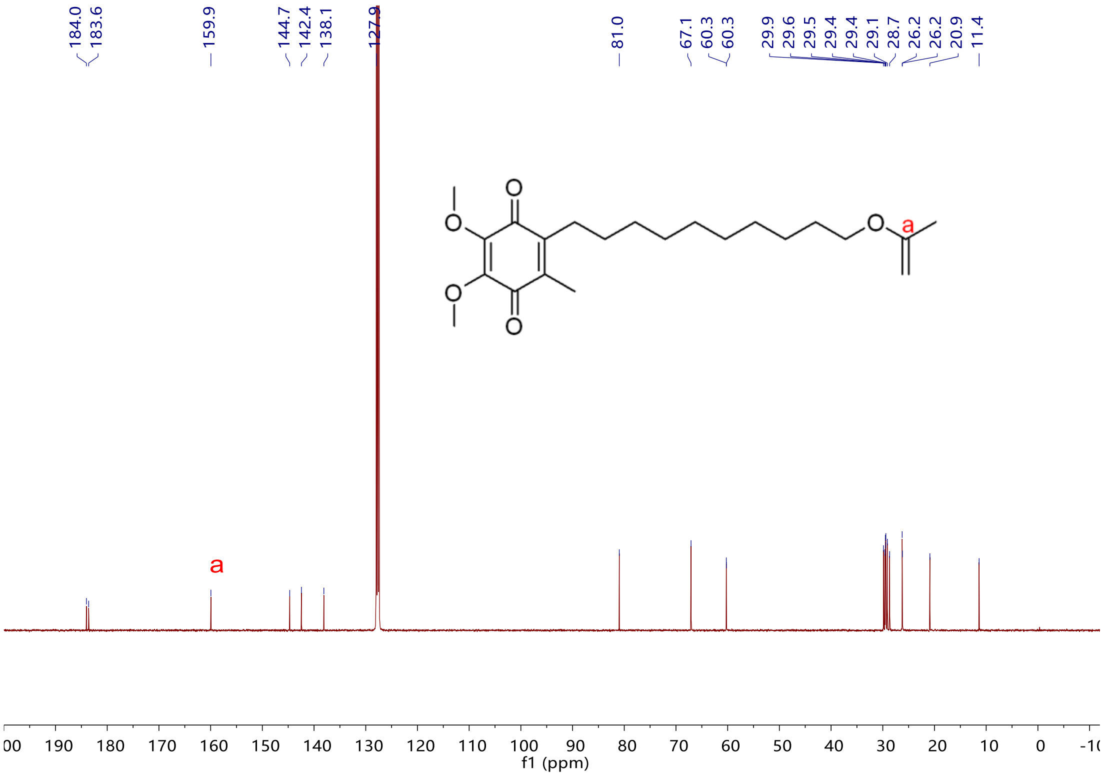

Supplementary Figure 69. ^{13}C NMR spectrum of compound **3k** (100 MHz, C_6D_6)

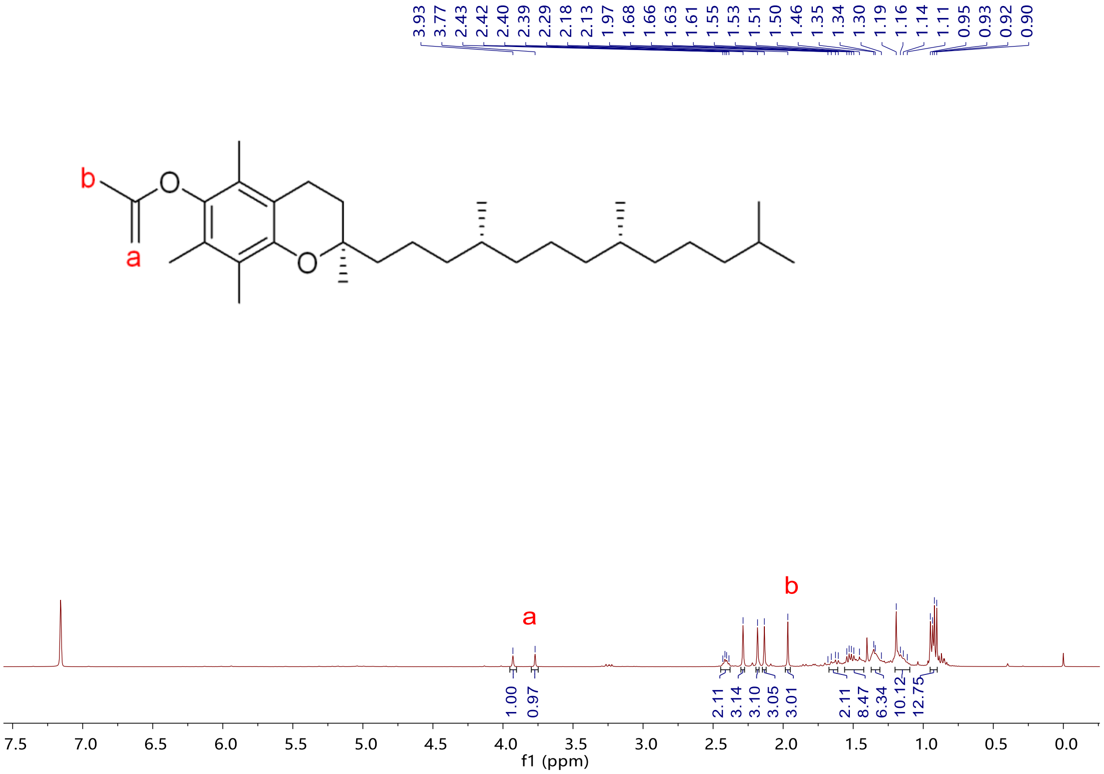

Supplementary Figure 70. ^{1}H NMR spectrum of compound **3l** (400 MHz, C_6D_6)

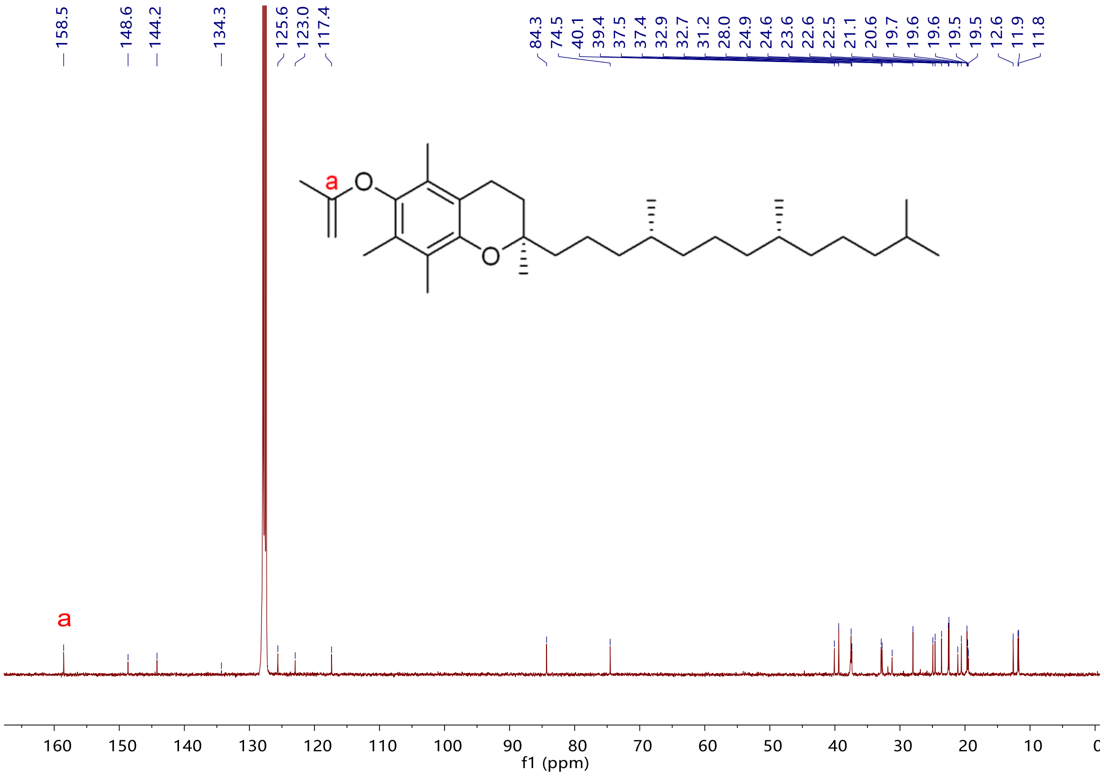

Supplementary Figure 71. ^{13}C NMR spectrum of compound **3l** (100 MHz, C_6D_6)

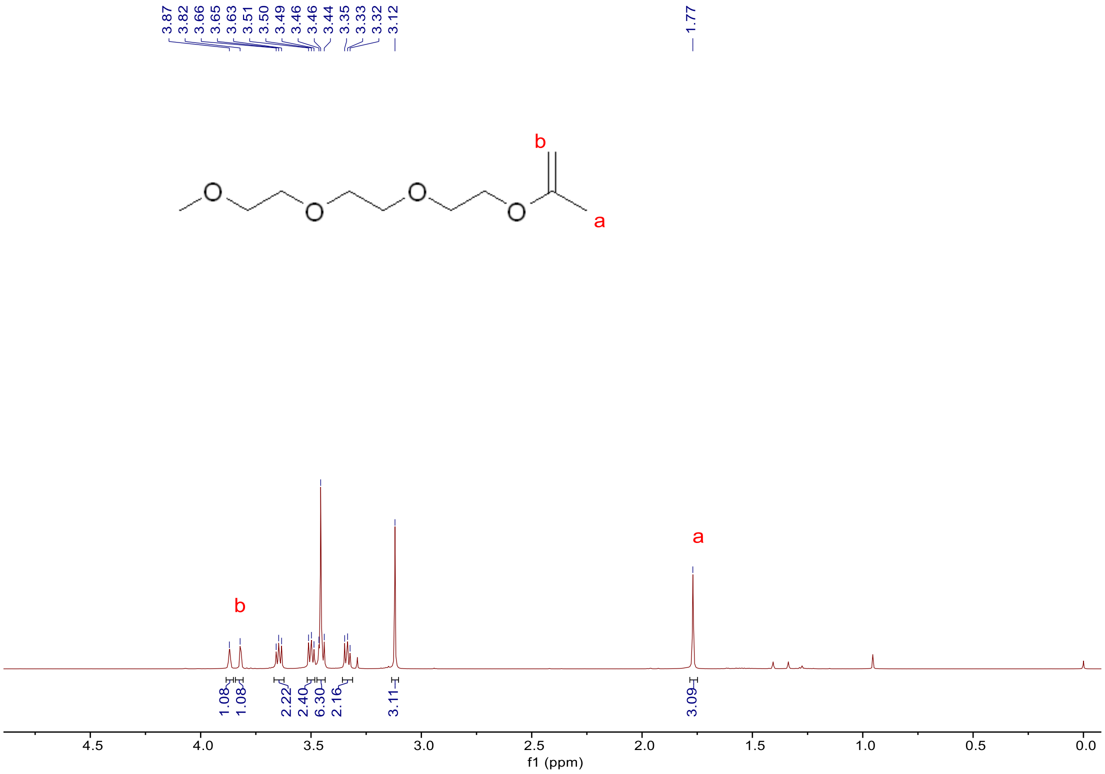

Supplementary Figure 72. ^{1}H NMR spectrum of compound **3m** (n = 3) (400 MHz, C_6D_6)

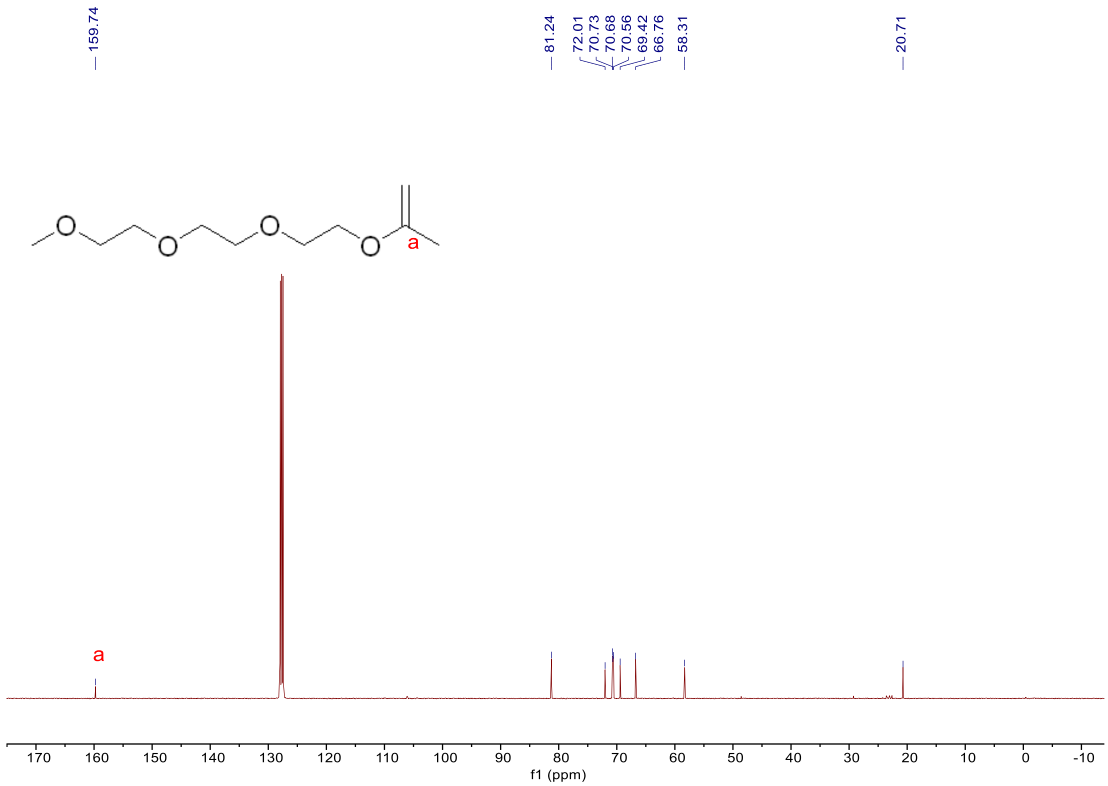

Supplementary Figure 73. ^{13}C NMR spectrum of compound **3m** (n = 3) (100 MHz, C_6D_6)

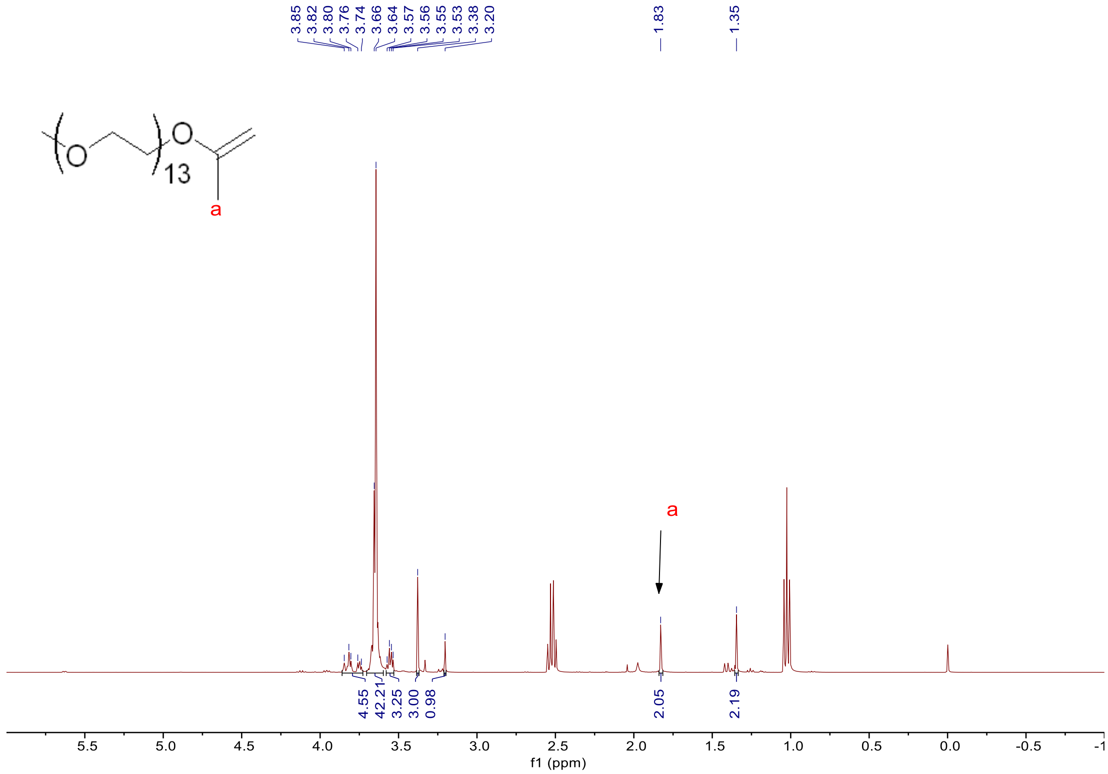

Supplementary Figure 74. ^{1}H NMR spectrum of compound **3m** (n = 13) (400 MHz, $CDCl_3$)

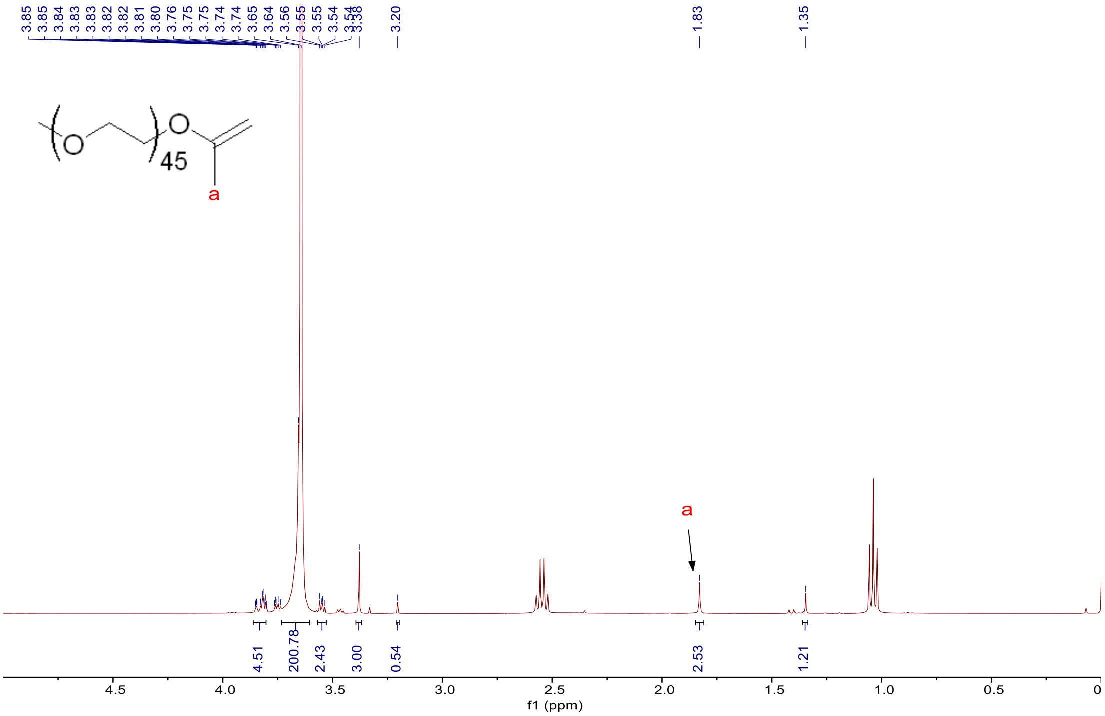

Supplementary Figure 75. ^{1}H NMR spectrum of compound **3m** (n = 45) (400 MHz, $CDCl_3$)

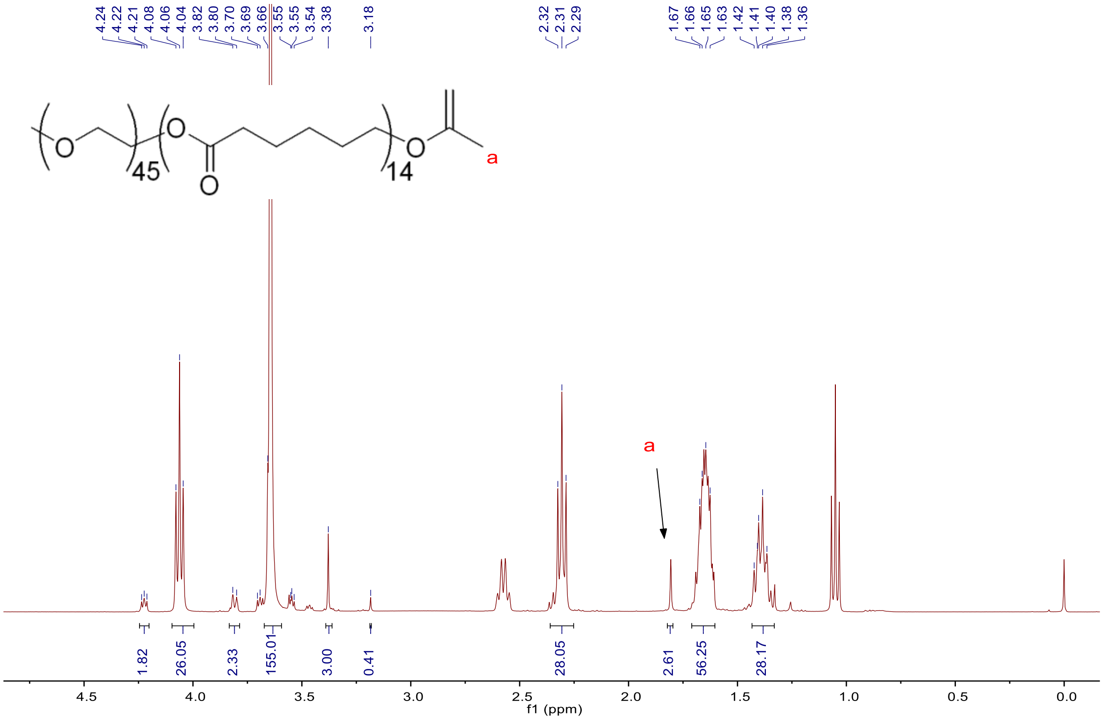

Supplementary Figure 76. ^{1}H NMR spectrum of compound **3n** (400 MHz, $CDCl_3$)

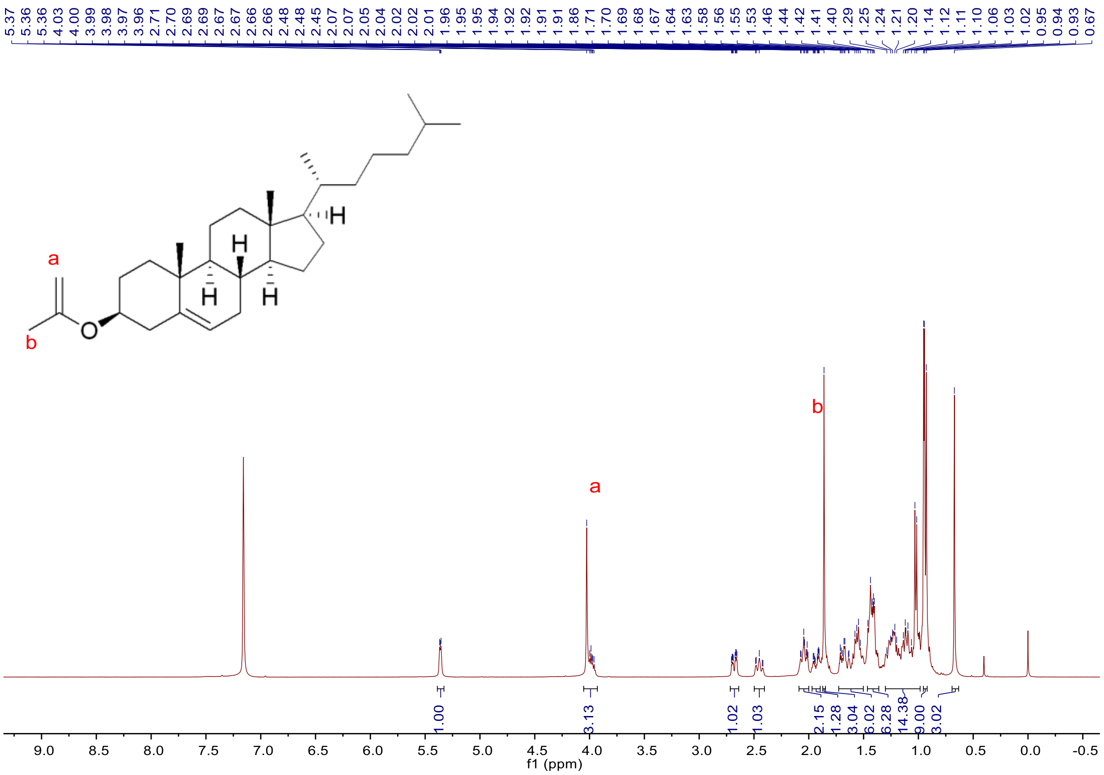

Supplementary Figure 77. ^{1}H NMR spectrum of compound **3o** (400 MHz, C_6D_6)

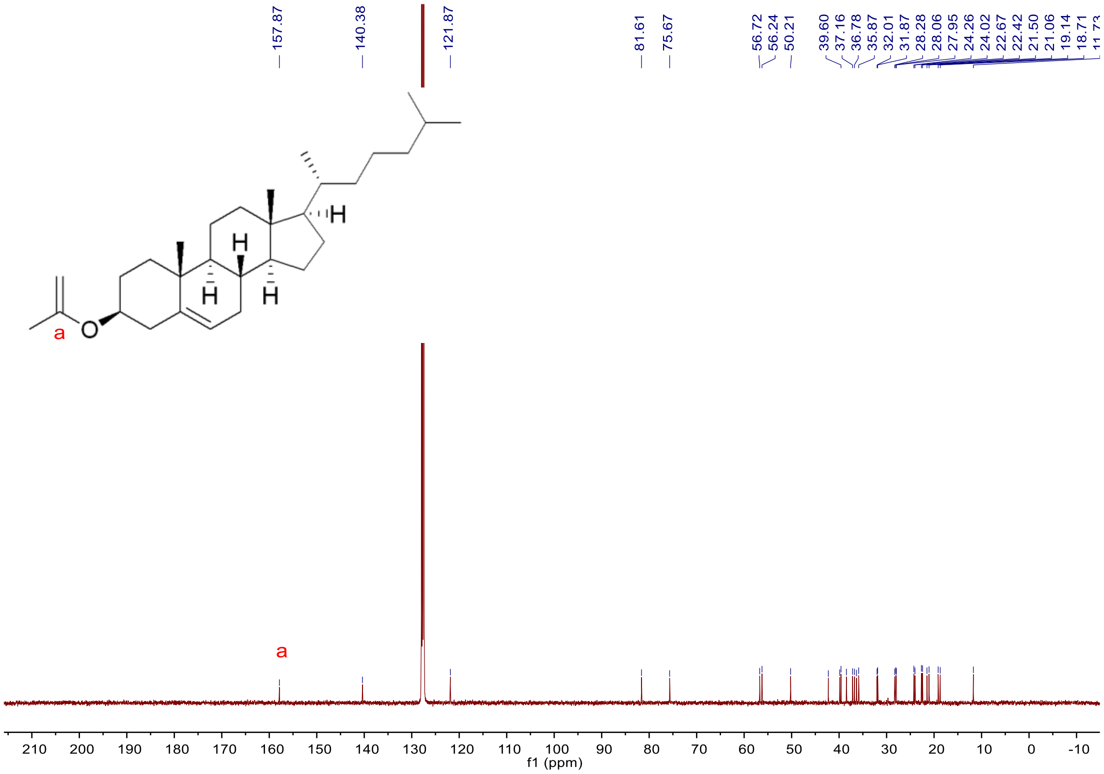

Supplementary Figure 78. ^{13}C NMR spectrum of compound **3o** (100 MHz, C_6D_6)

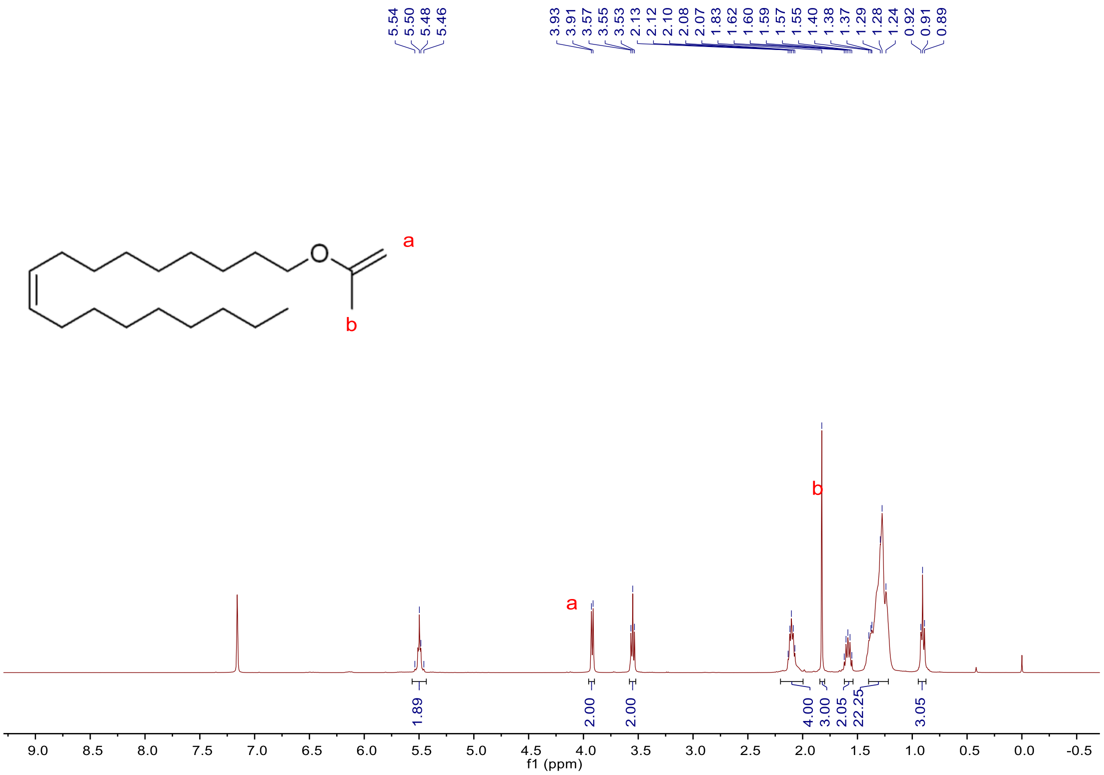

Supplementary Figure 79. ^{1}H NMR spectrum of compound **3p** (400 MHz, C_6D_6)

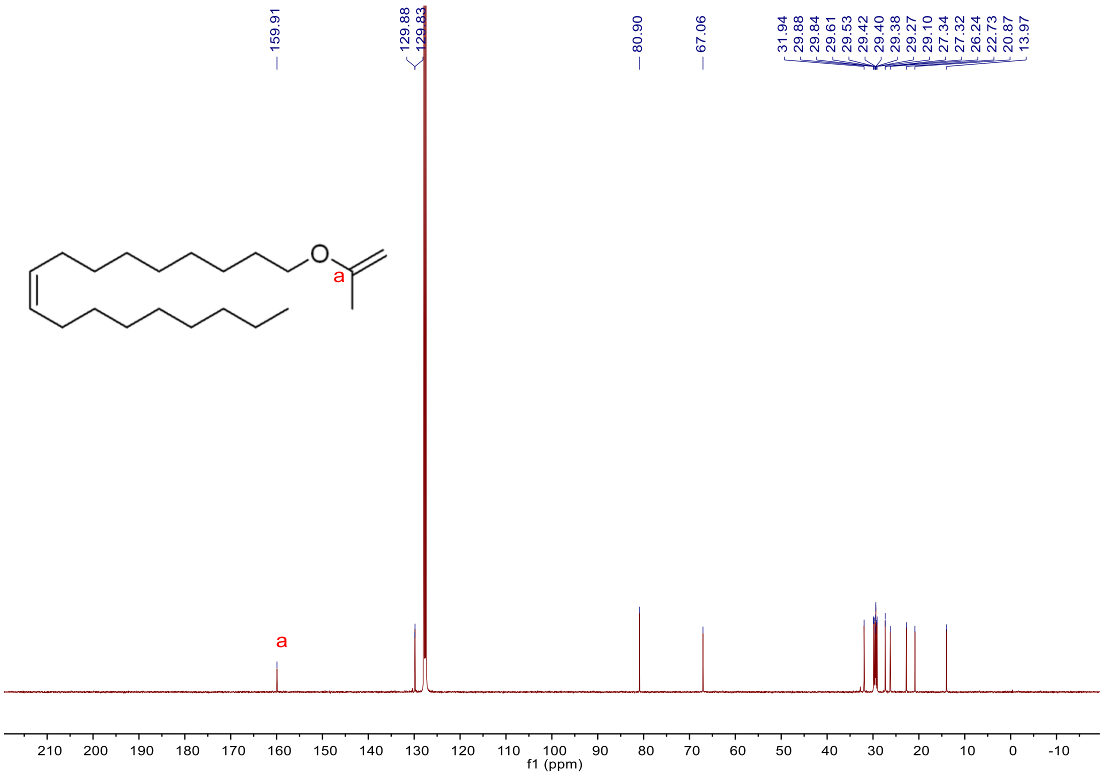

Supplementary Figure 80. ^{13}C NMR spectrum of compound **3p** (100 MHz, C_6D_6)

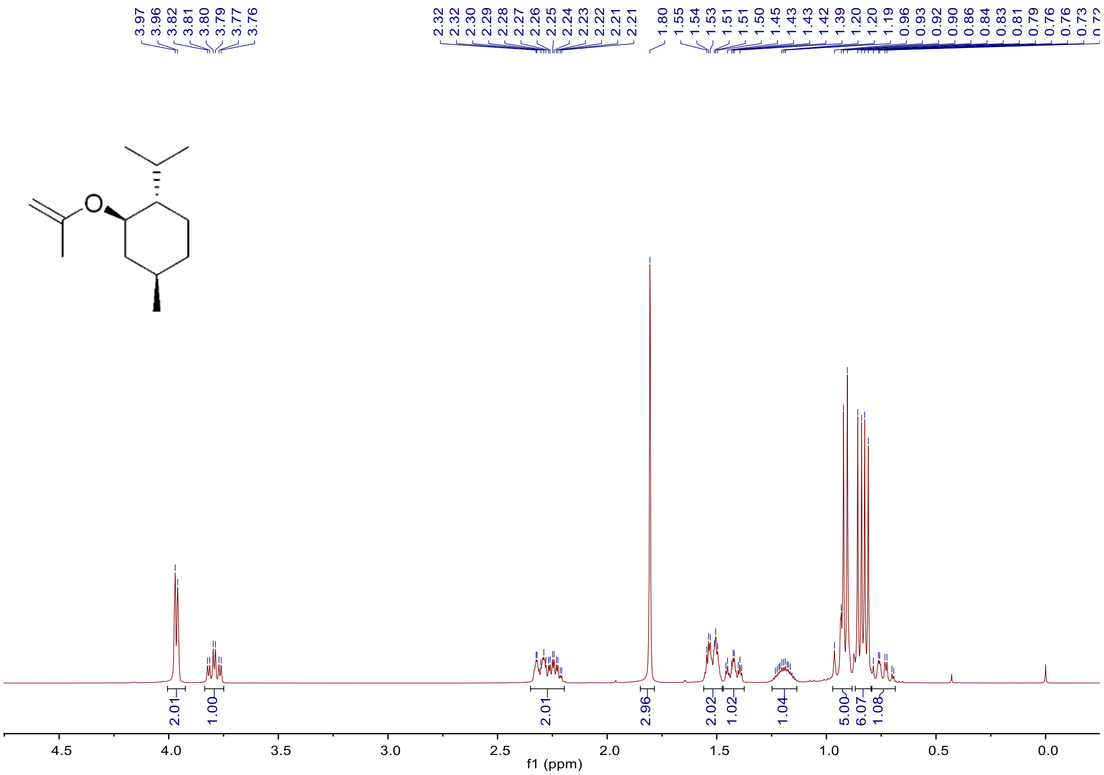

Supplementary Figure 81. ^{1}H NMR spectrum of compound **3q** (400 MHz, C_6D_6)

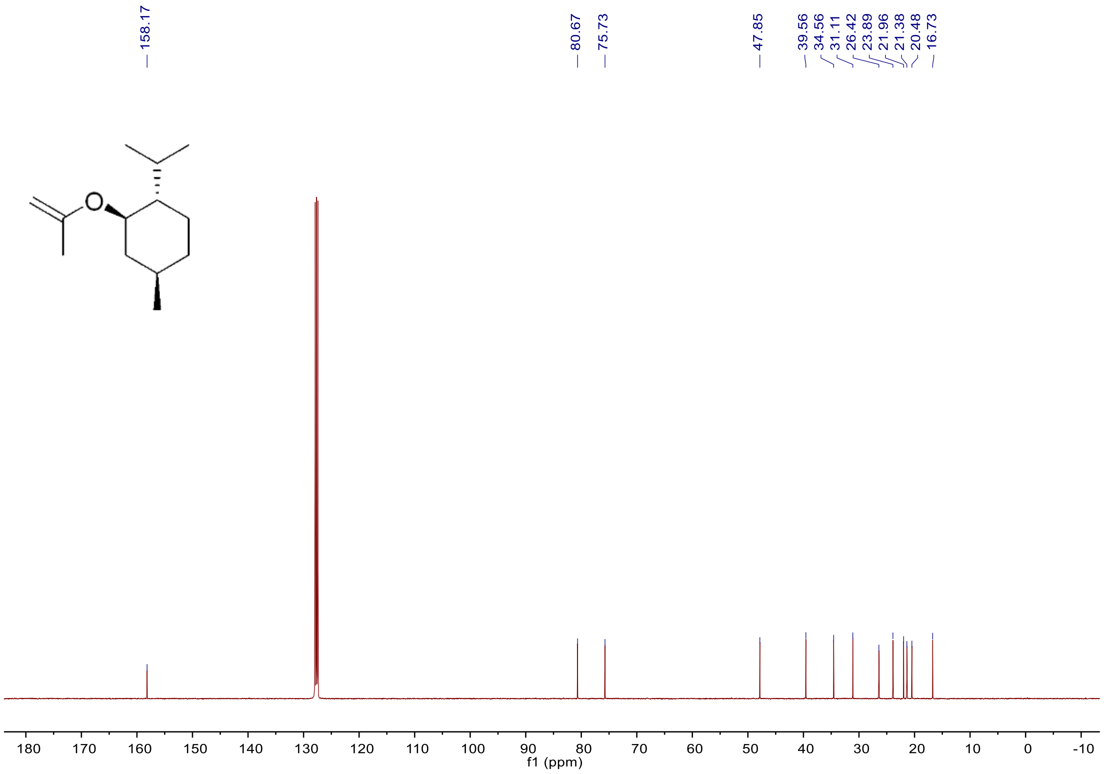

Supplementary Figure 82. ^{13}C NMR spectrum of compound **3q** (100 MHz, C_6D_6)

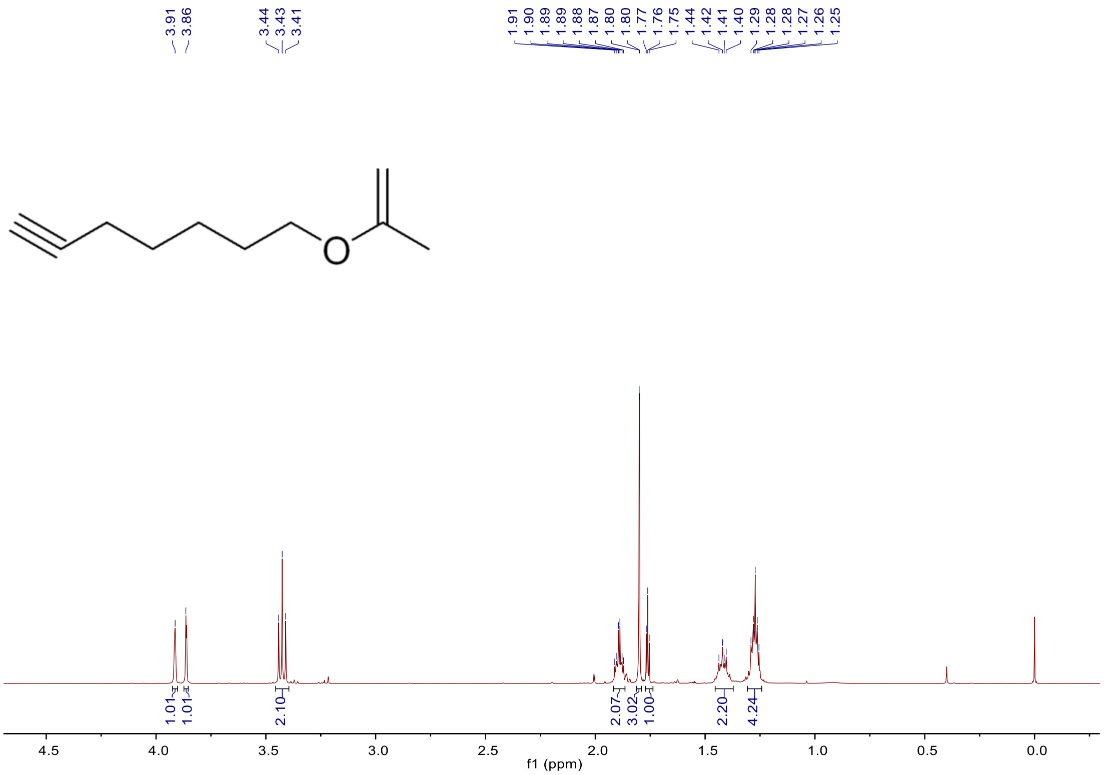

Supplementary Figure 83. ^{1}H NMR spectrum of compound **3r** (400 MHz, C_6D_6)

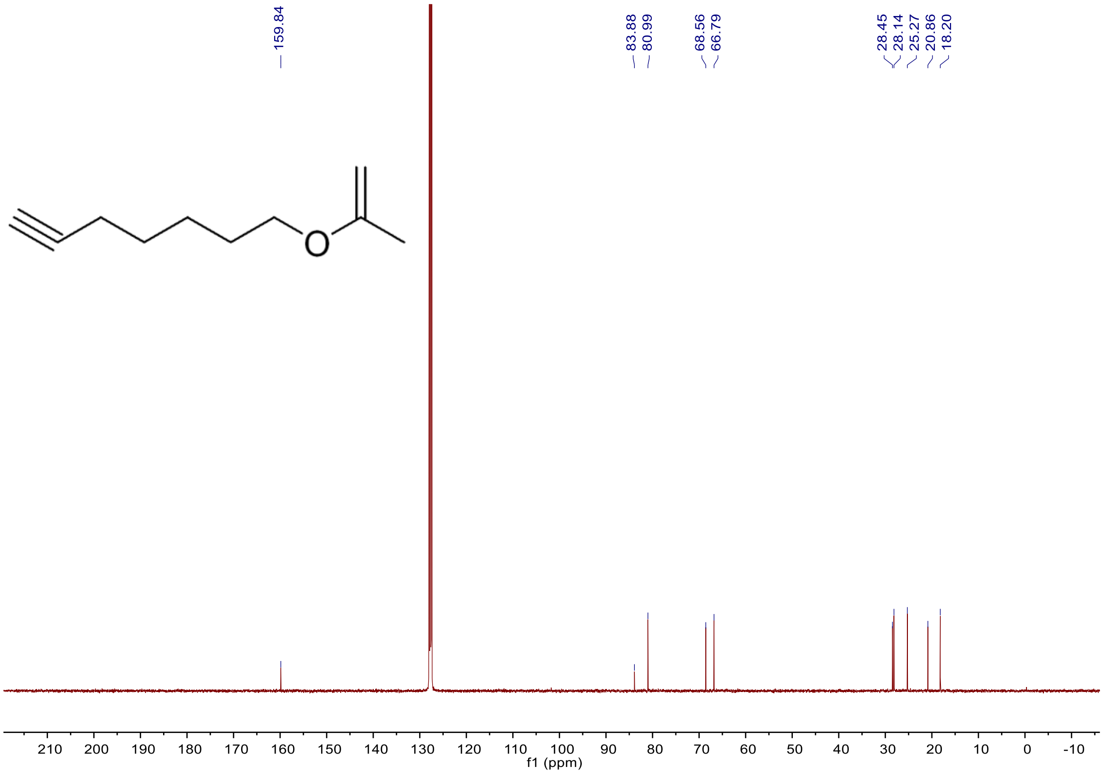

Supplementary Figure 84. ^{13}C NMR spectrum of compound **3r** (100 MHz, C_6D_6)

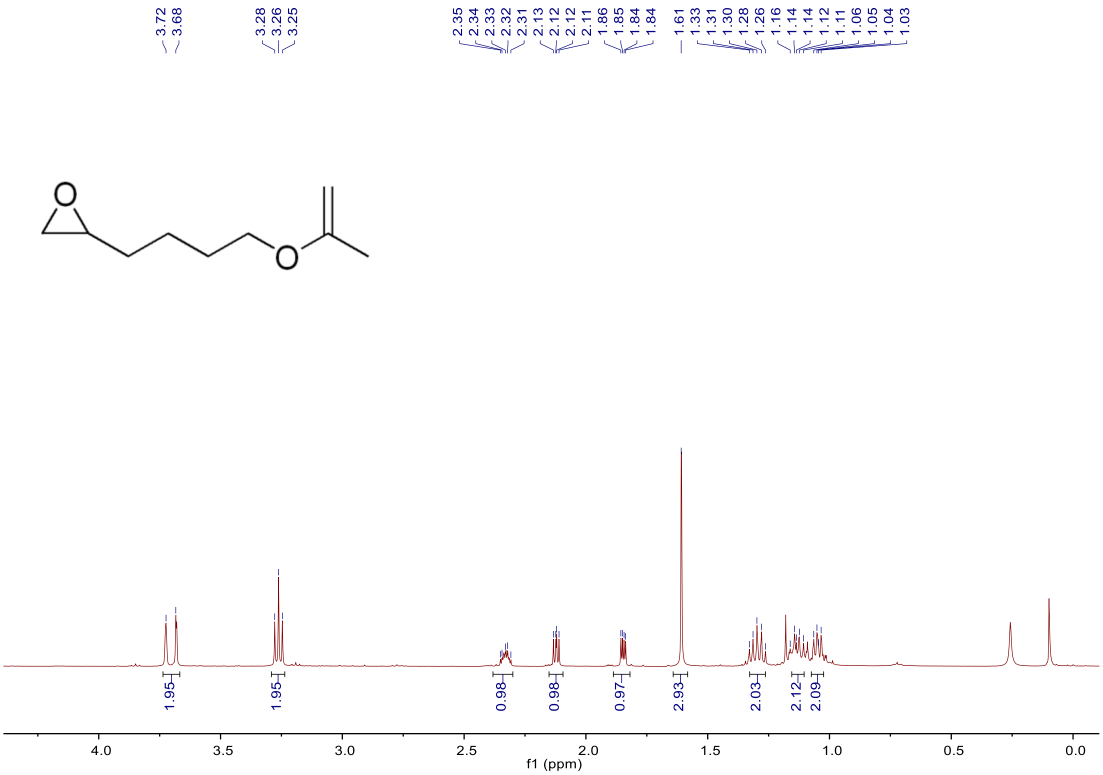

Supplementary Figure 85. ^{1}H NMR spectrum of compound **3s** (400 MHz, C_6D_6)

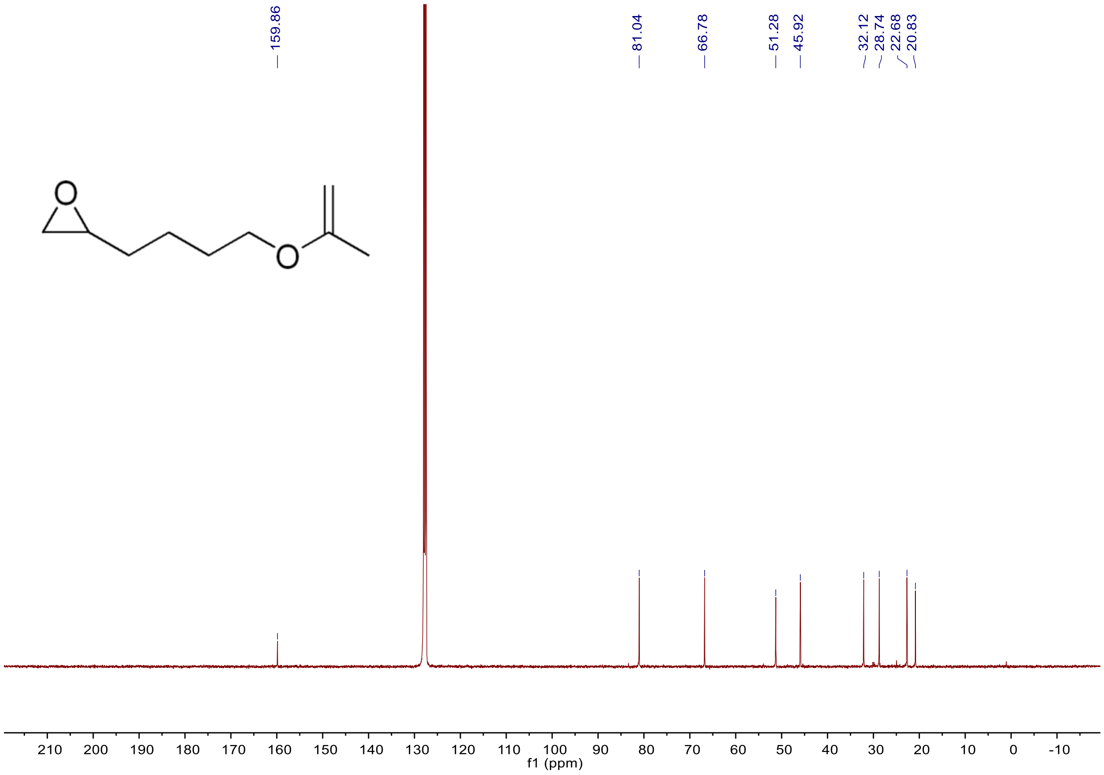

Supplementary Figure 86. ^{13}C NMR spectrum of compound **3s** (100 MHz, C_6D_6)

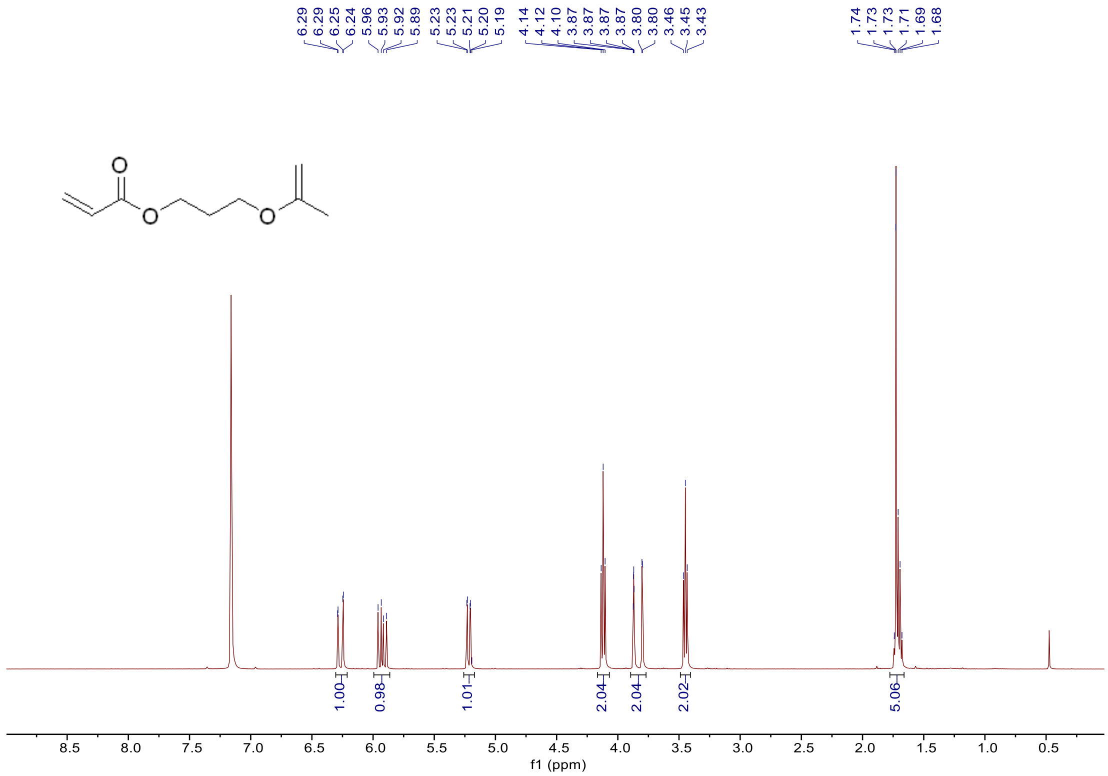

Supplementary Figure 87. ^{1}H NMR spectrum of compound **3t** (400 MHz, C_6D_6)

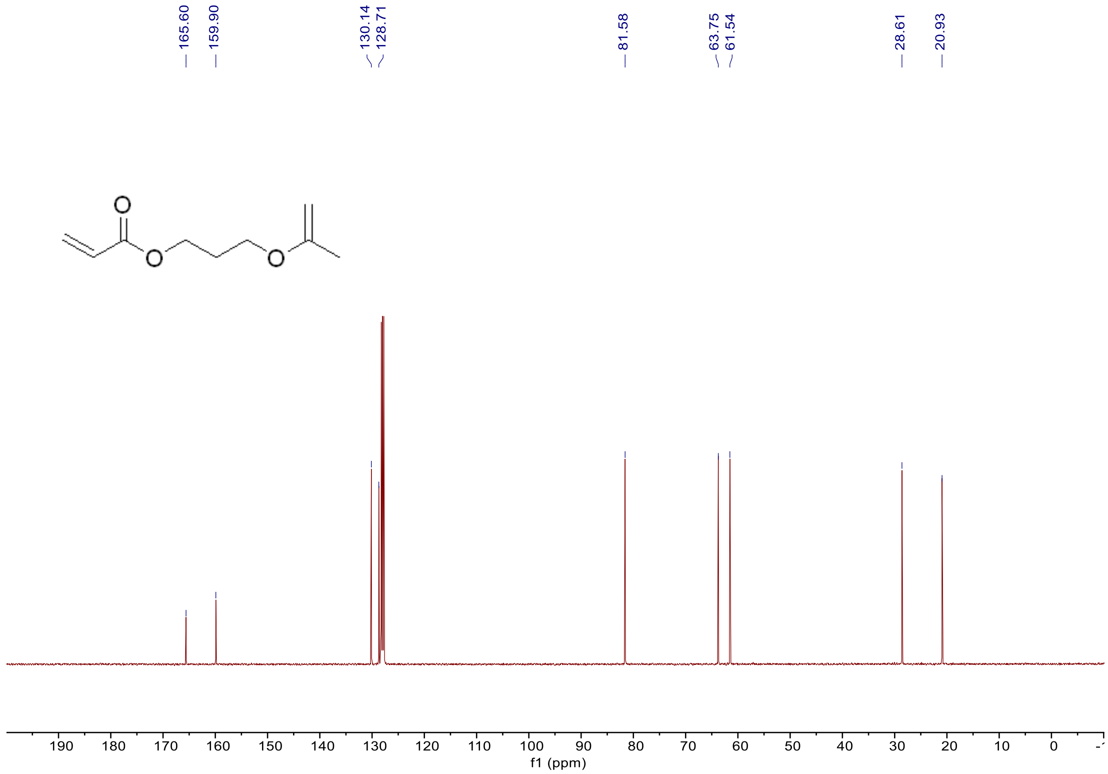

Supplementary Figure 88. ^{13}C NMR spectrum of compound **3t** (100 MHz, C_6D_6)

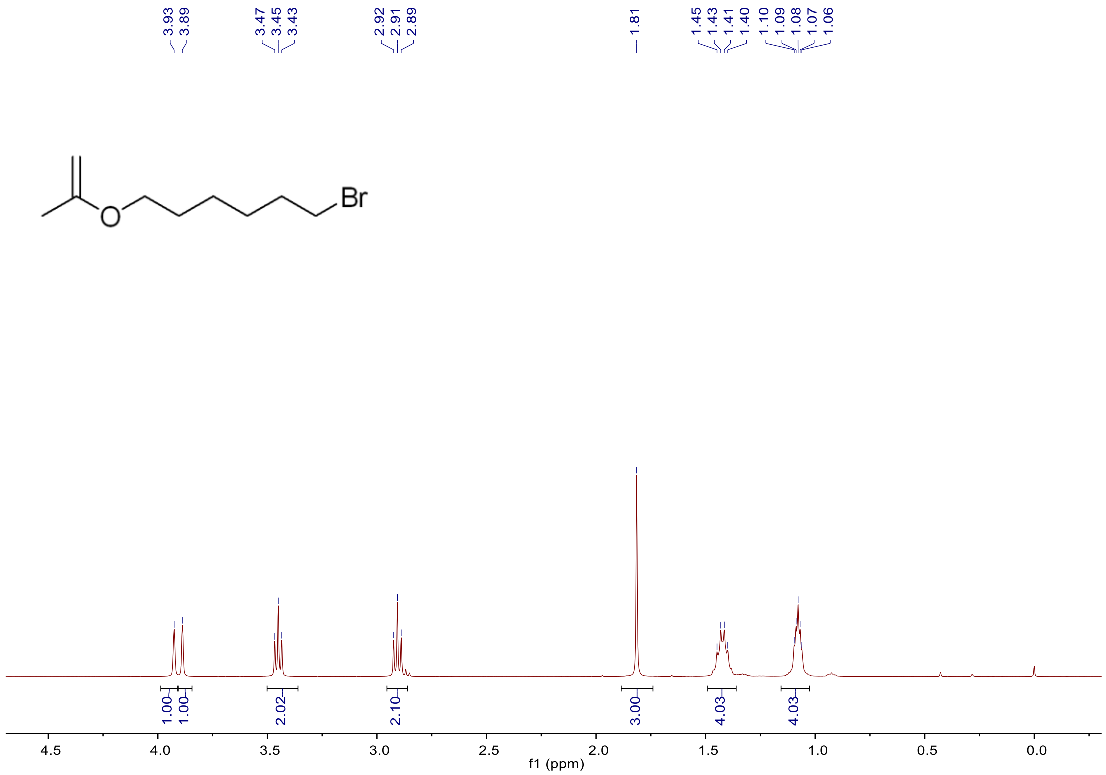

Supplementary Figure 89. ^{1}H NMR spectrum of compound **3u** (400 MHz, C_6D_6)

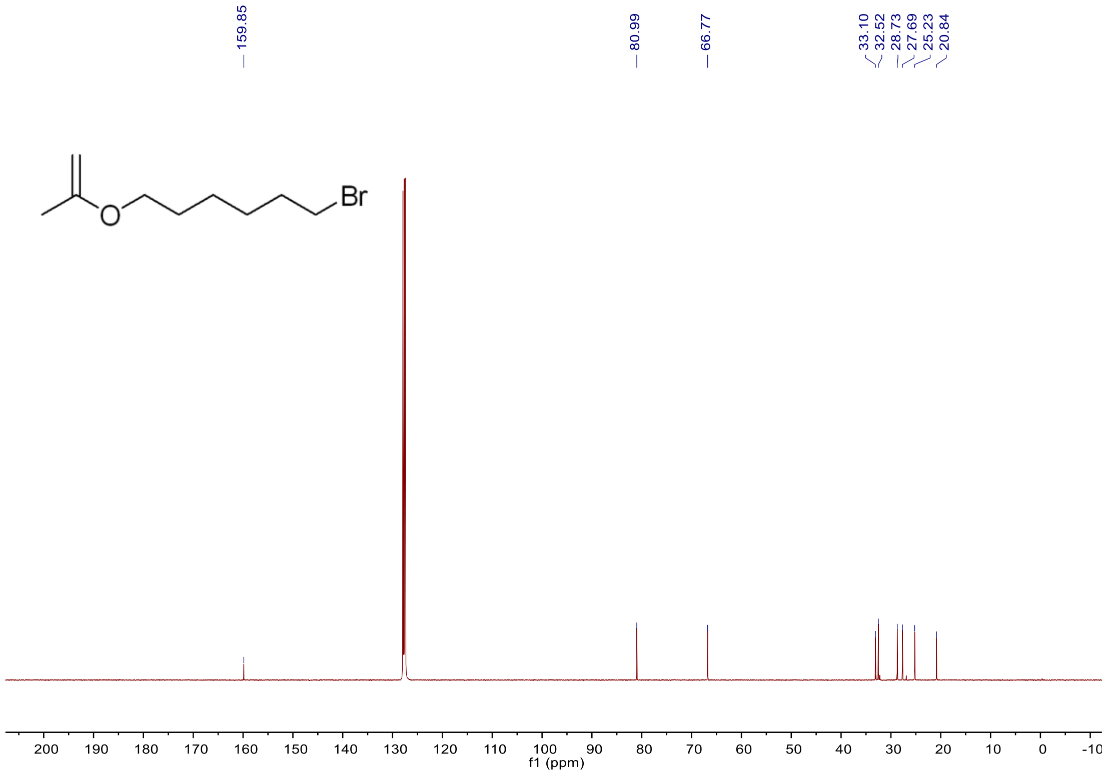

Supplementary Figure 90. ^{13}C NMR spectrum of compound **3u** (100 MHz, C_6D_6)

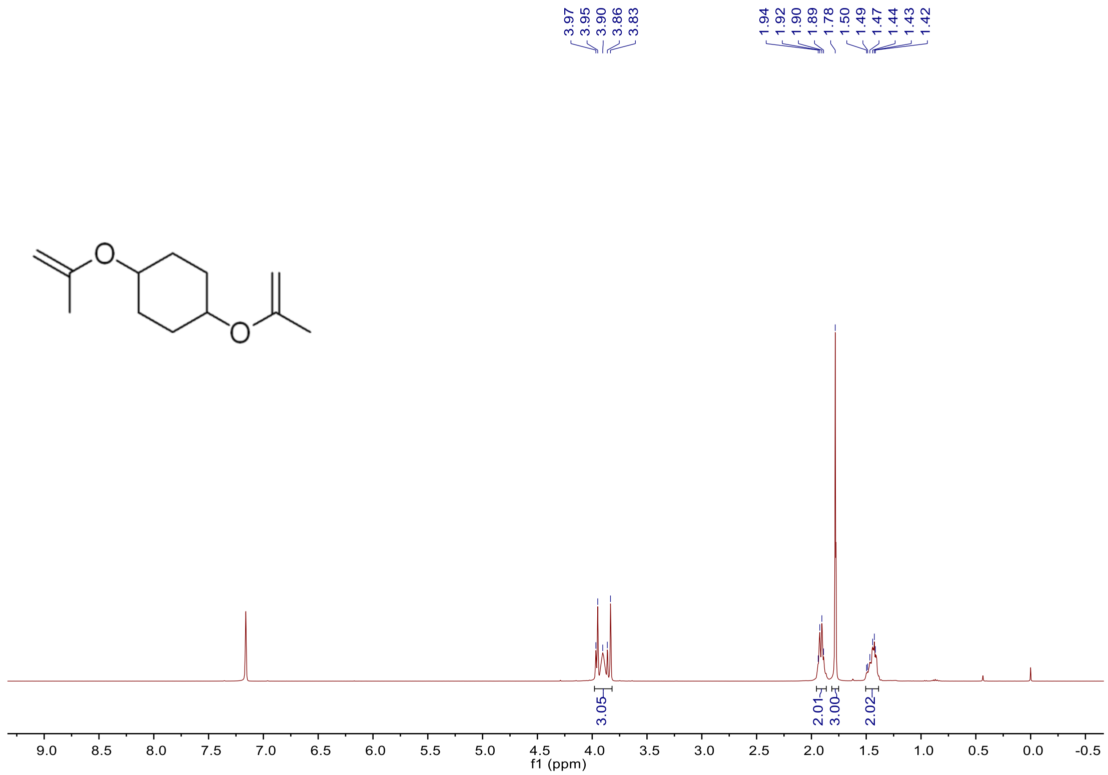

Supplementary Figure 91. ^{1}H NMR spectrum of compound **3v** (400 MHz, C_6D_6)

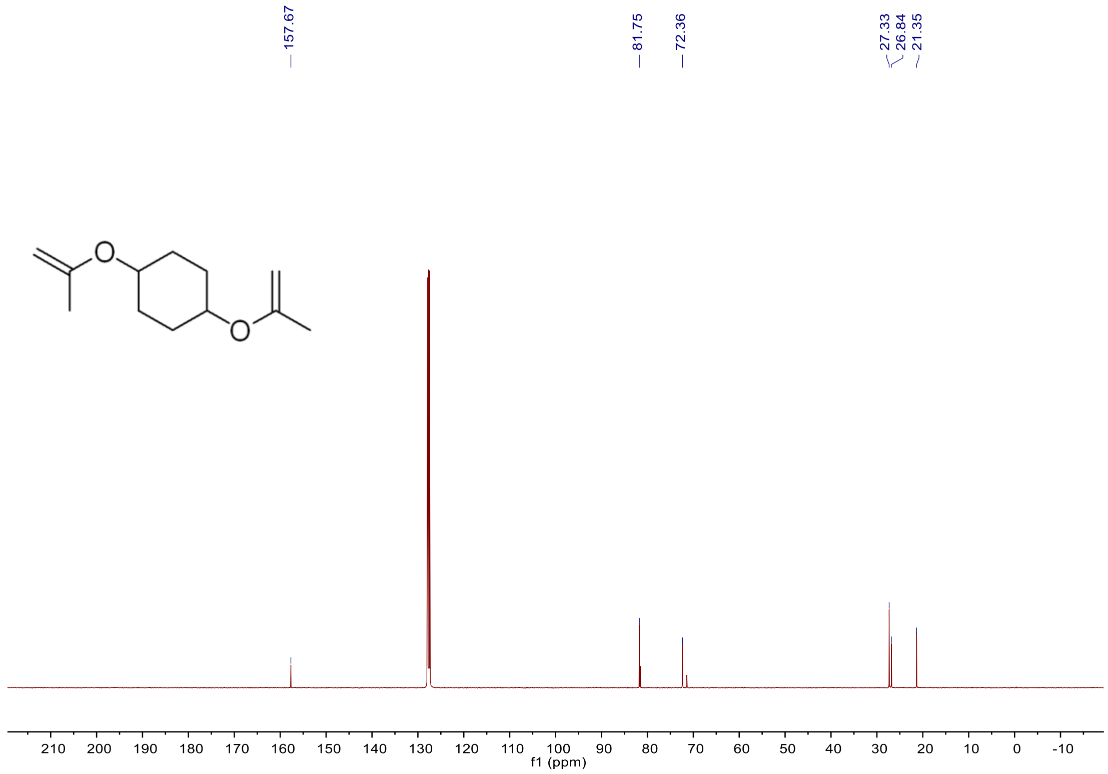

Supplementary Figure 92. ^{13}C NMR spectrum of compound **3v** (100 MHz, C_6D_6)

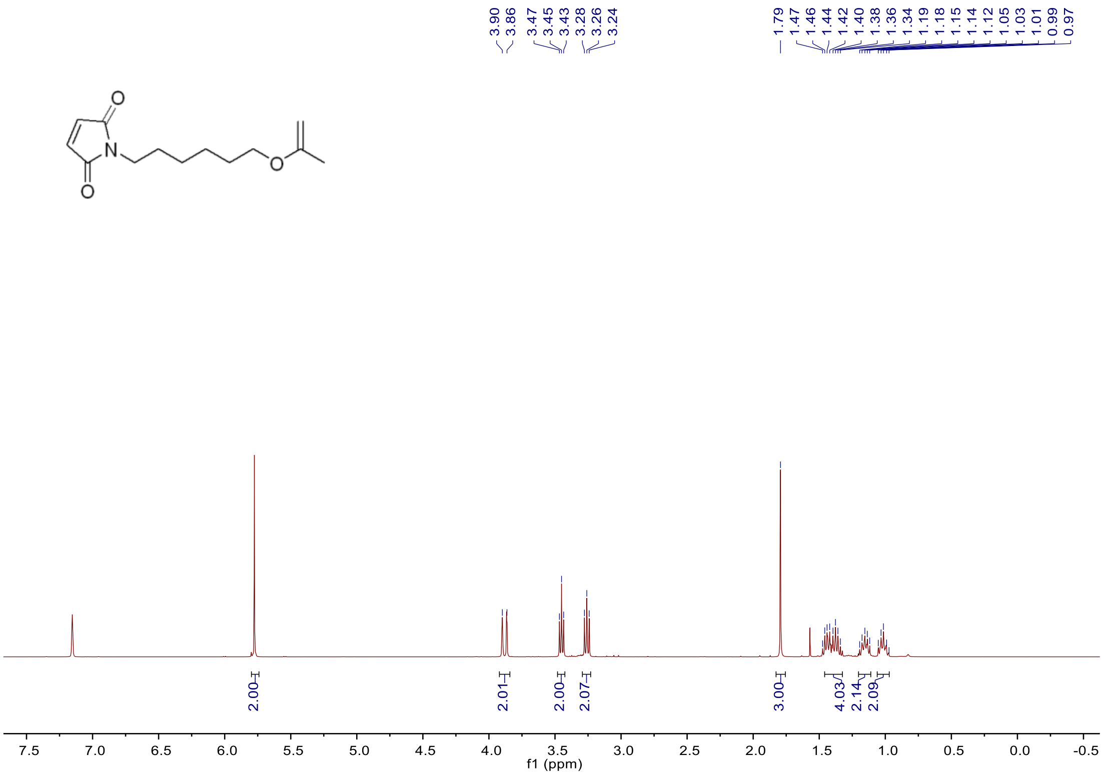

Supplementary Figure 93. ^{1}H NMR spectrum of compound **3w** (400 MHz, C_6D_6)

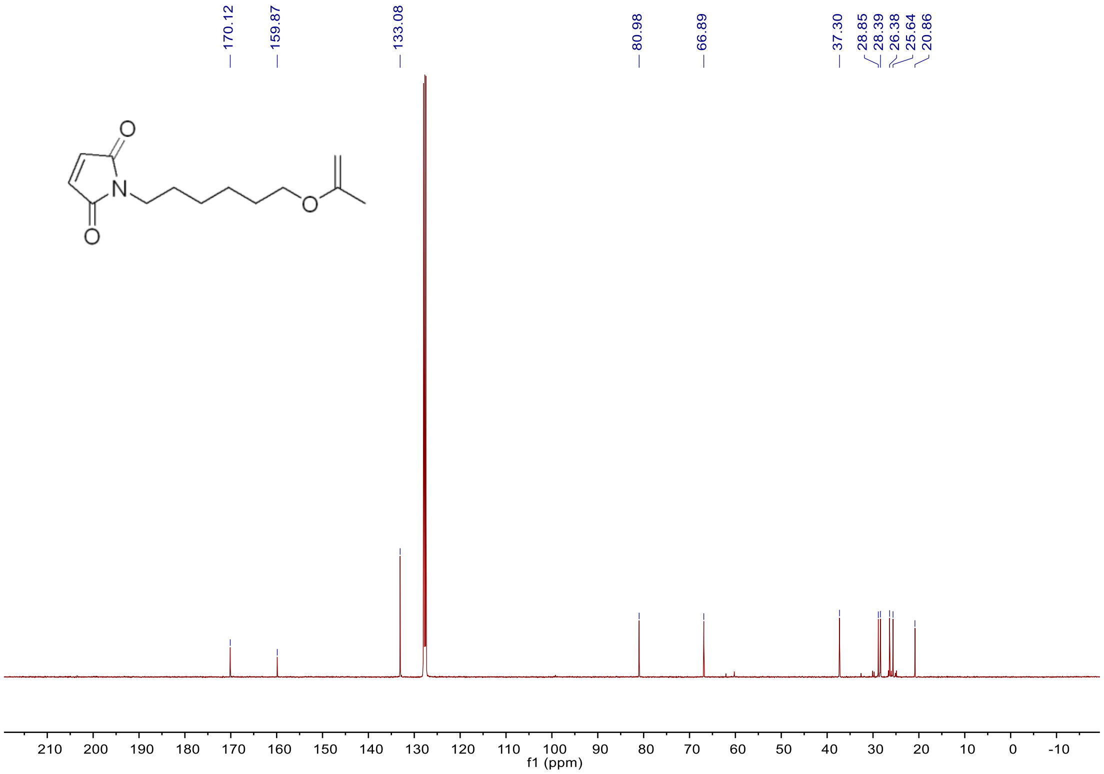

Supplementary Figure 94. ^{13}C NMR spectrum of compound **3w** (100 MHz, C_6D_6)

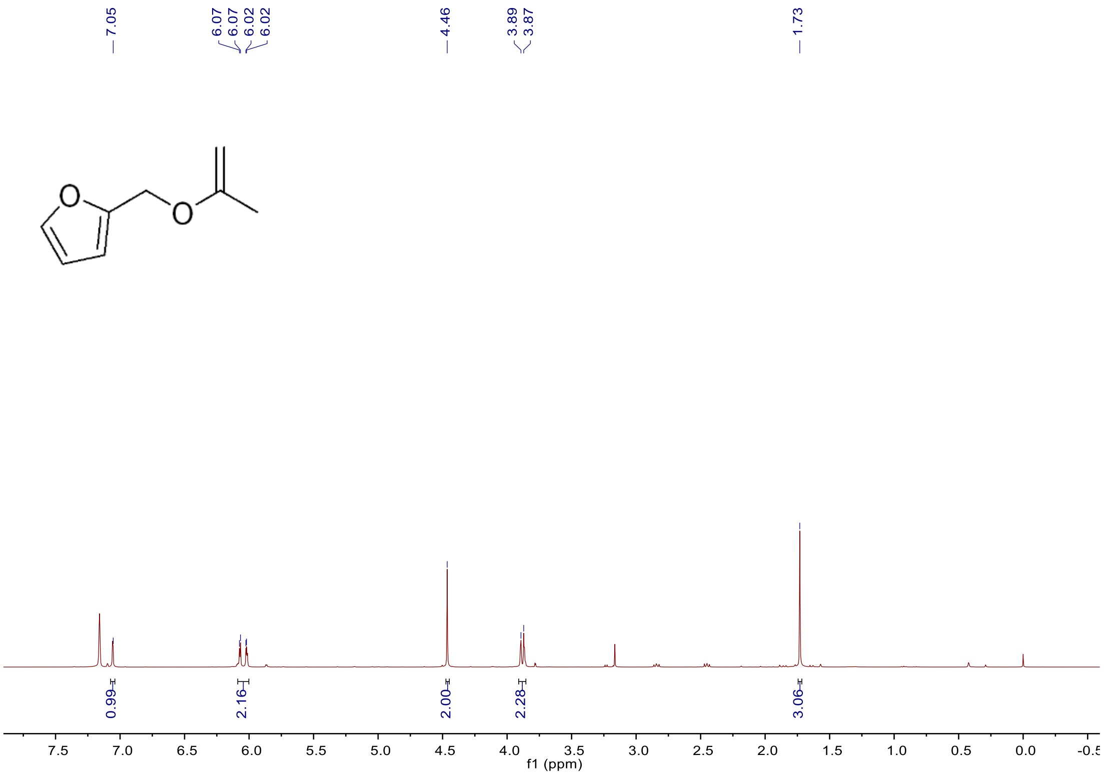

Supplementary Figure 95. ^{1}H NMR spectrum of compound **3x** (400 MHz, C_6D_6)

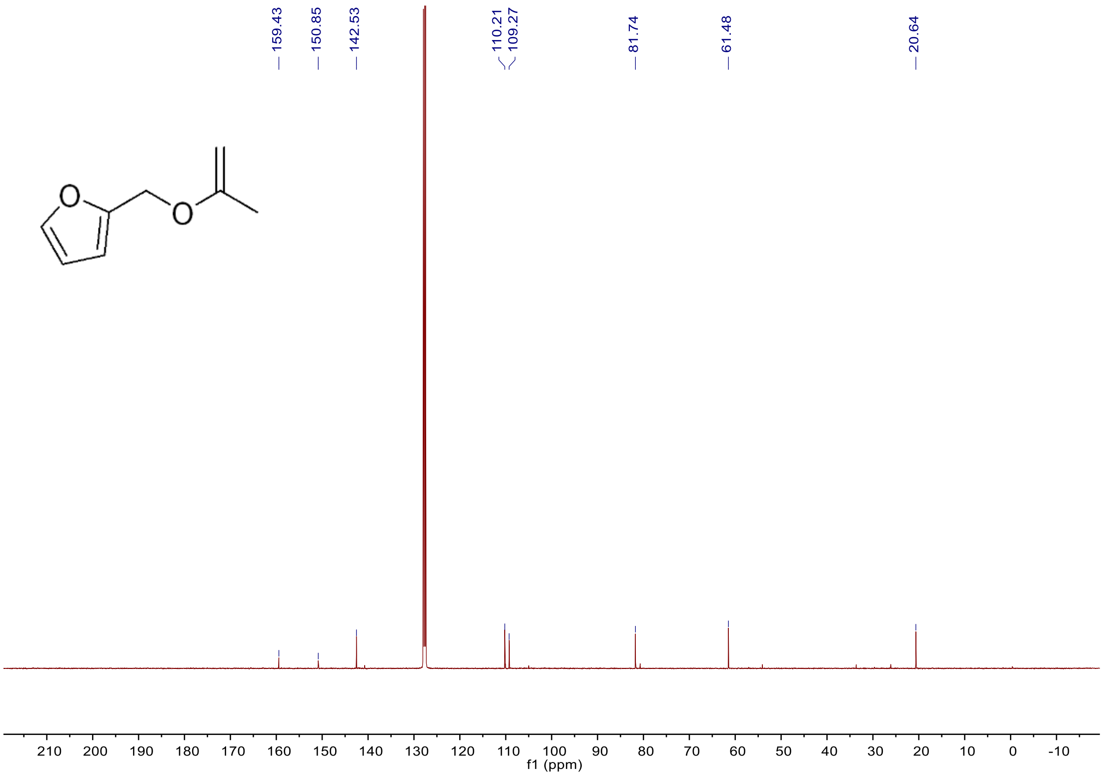

Supplementary Figure 96. ^{13}C NMR spectrum of compound **3x** (100 MHz, C_6D_6)

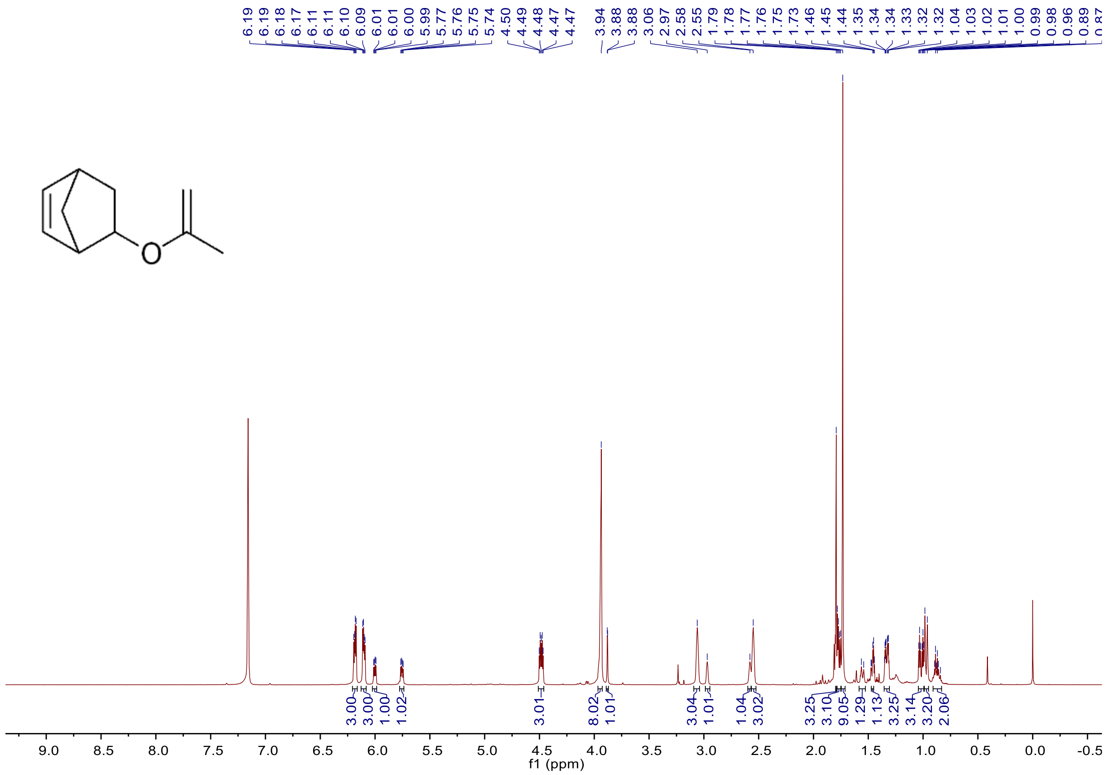

Supplementary Figure 97. ^{1}H NMR spectrum of compound **3y** (400 MHz, C_6D_6)

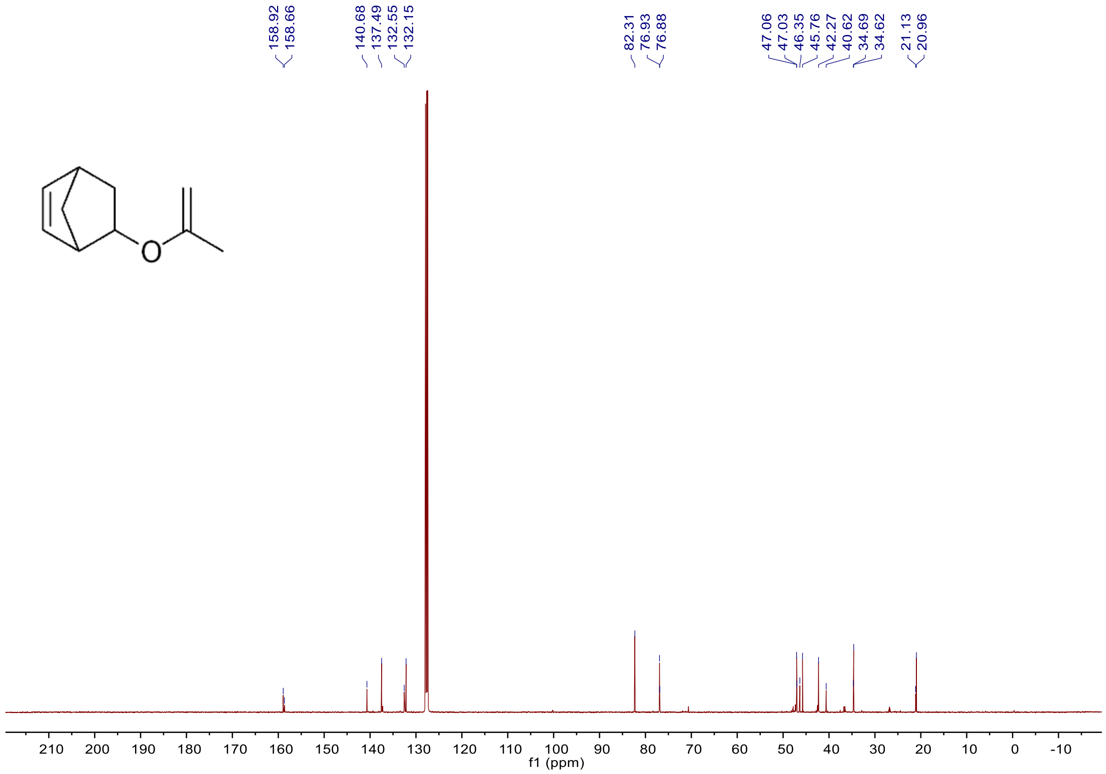

Supplementary Figure 98. ^{13}C NMR spectrum of compound **3y** (100 MHz, C_6D_6)

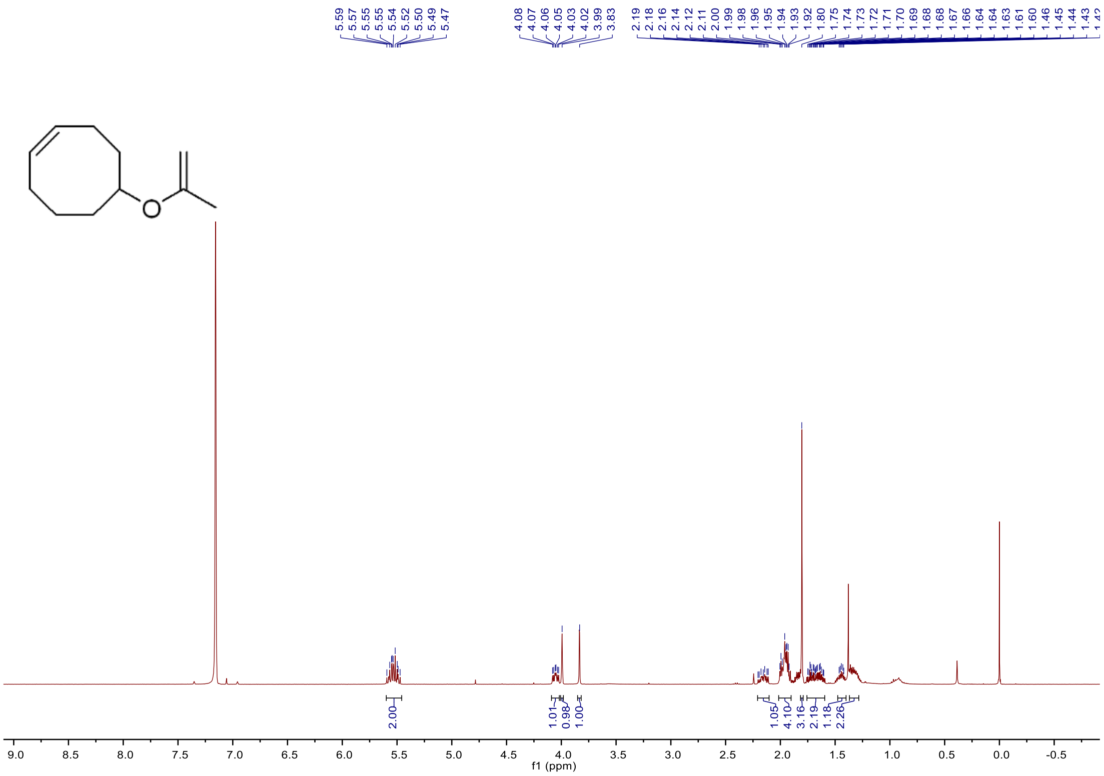

Supplementary Figure 99. ^{1}H NMR spectrum of compound **3z** (400 MHz, C_6D_6)

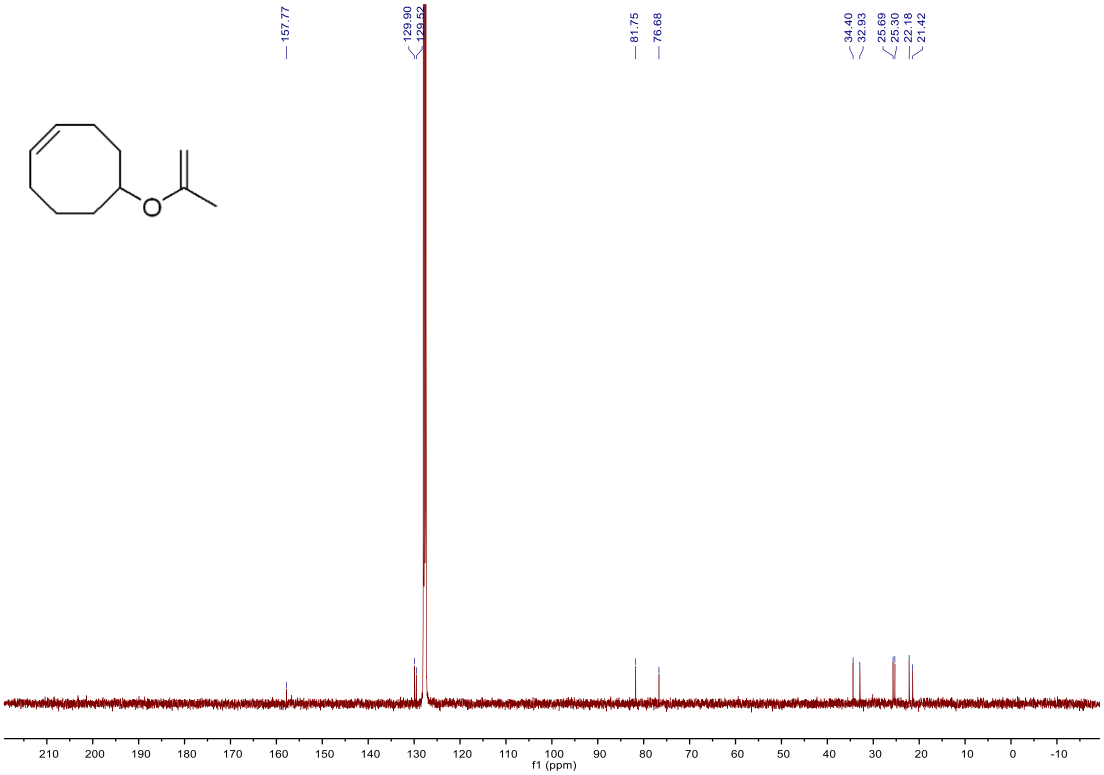

Supplementary Figure 100. ^{13}C NMR spectrum of compound **3z** (100 MHz, C_6D_6)

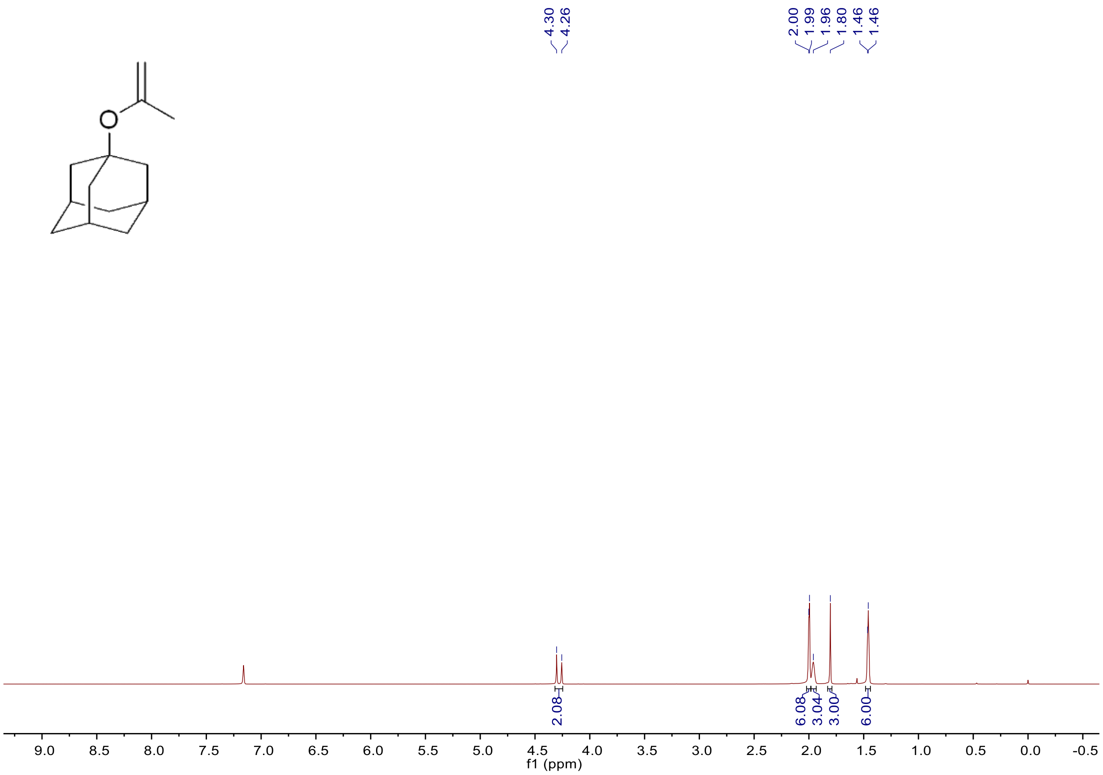

Supplementary Figure 101. ^{1}H NMR spectrum of compound **3aa** (400 MHz, C_6D_6)

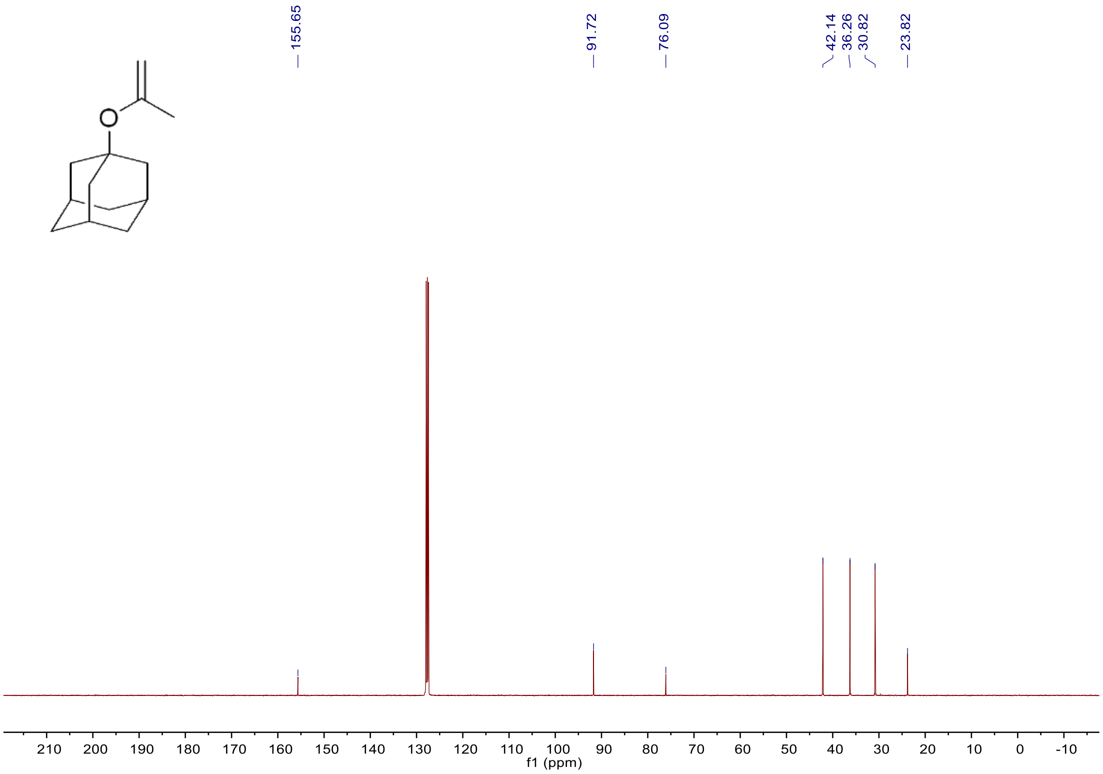

Supplementary Figure 102. ^{13}C NMR spectrum of compound **3aa** (100 MHz, C_6D_6)

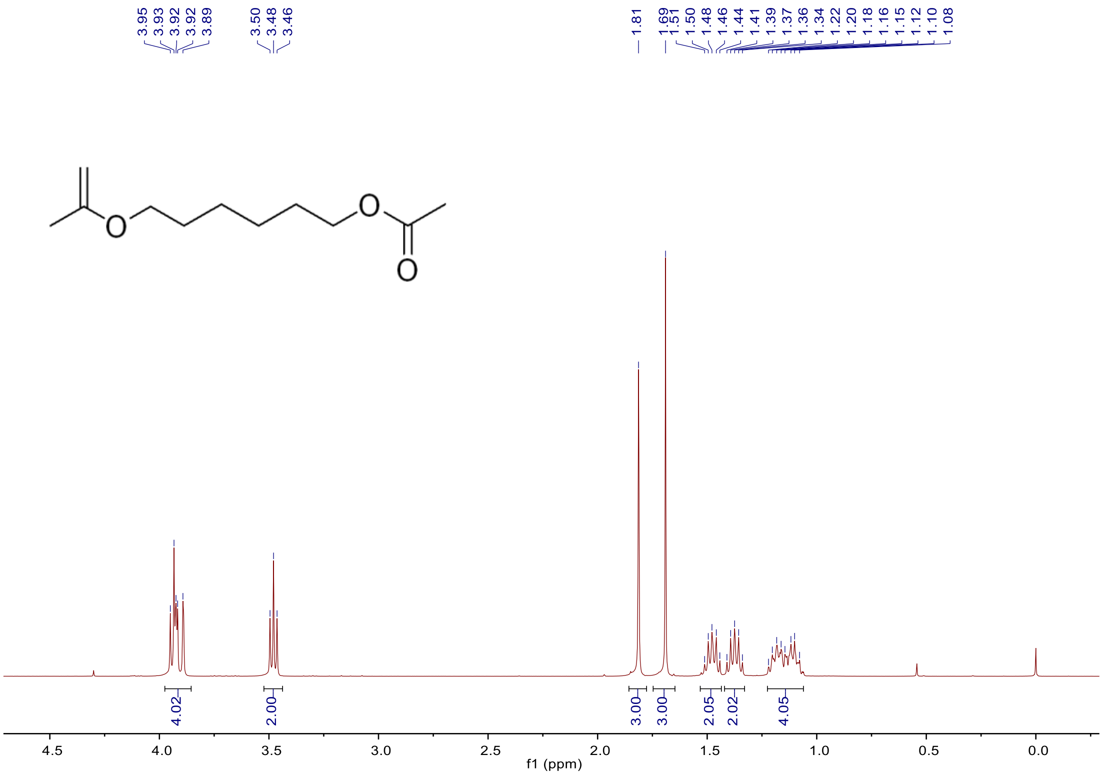

Supplementary Figure 103. ^{1}H NMR spectrum of compound **3ab** (400 MHz, C_6D_6)

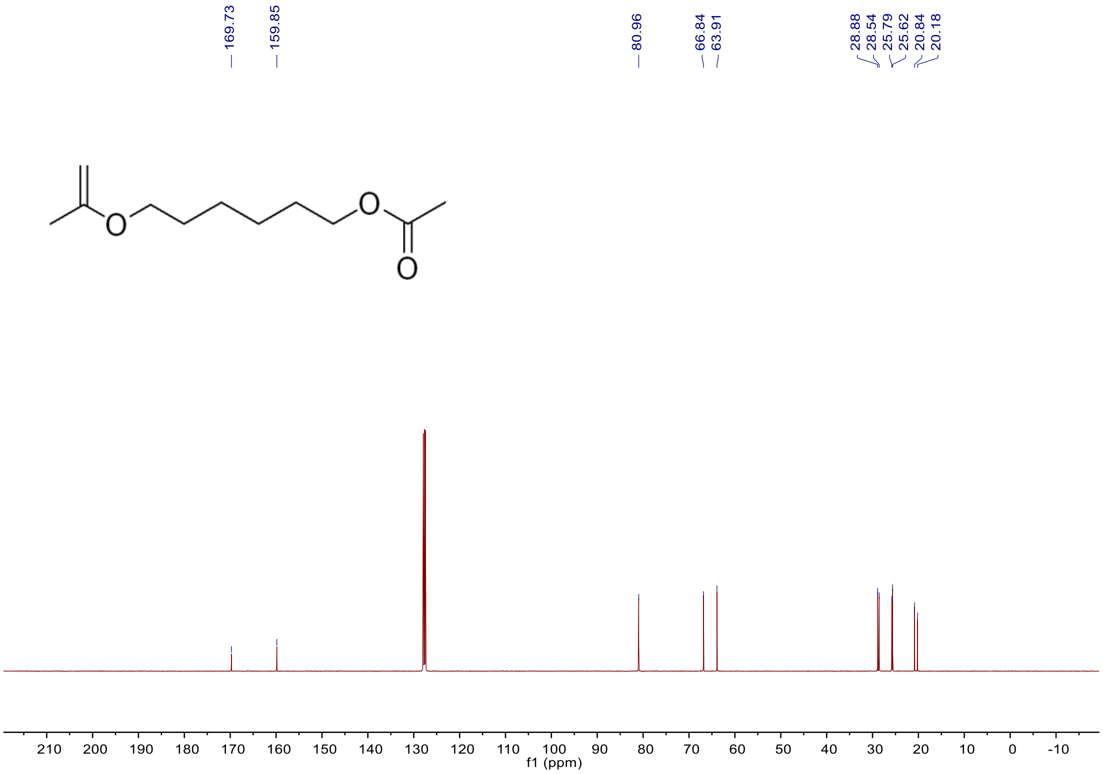

Supplementary Figure 104. ^{13}C NMR spectrum of compound **3ab** (100 MHz, C_6D_6)

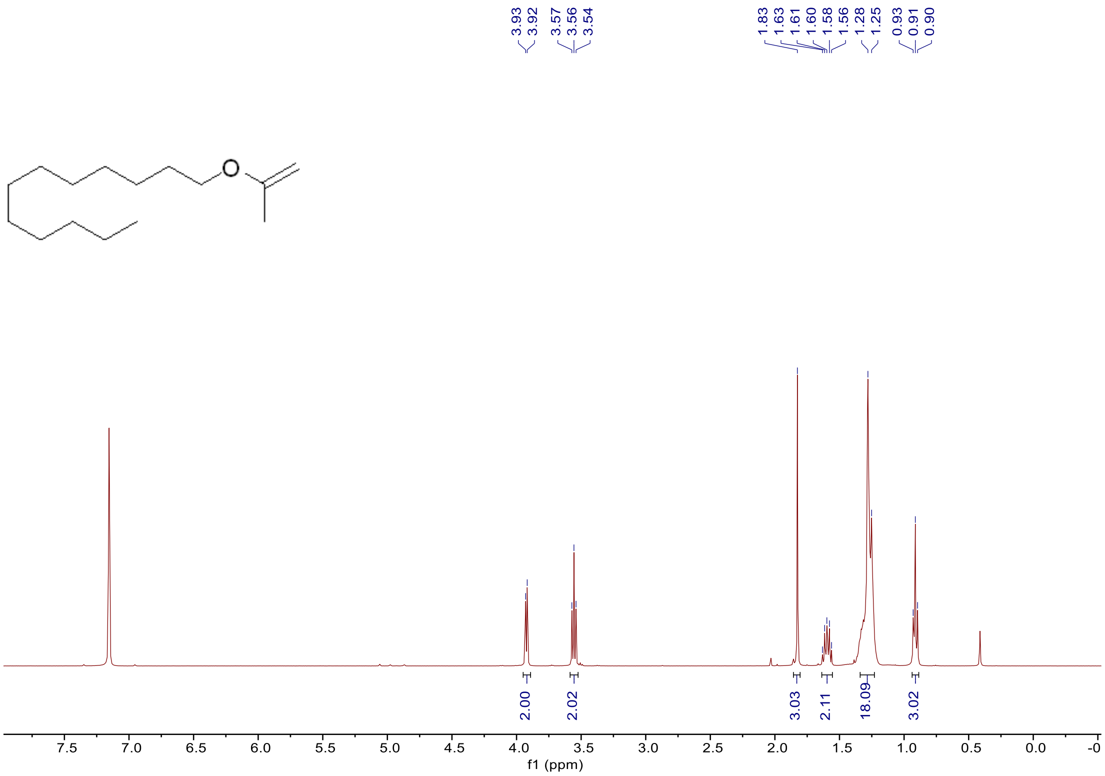

Supplementary Figure 105. ^{1}H NMR spectrum of compound **3ac** (400 MHz, C_6D_6)

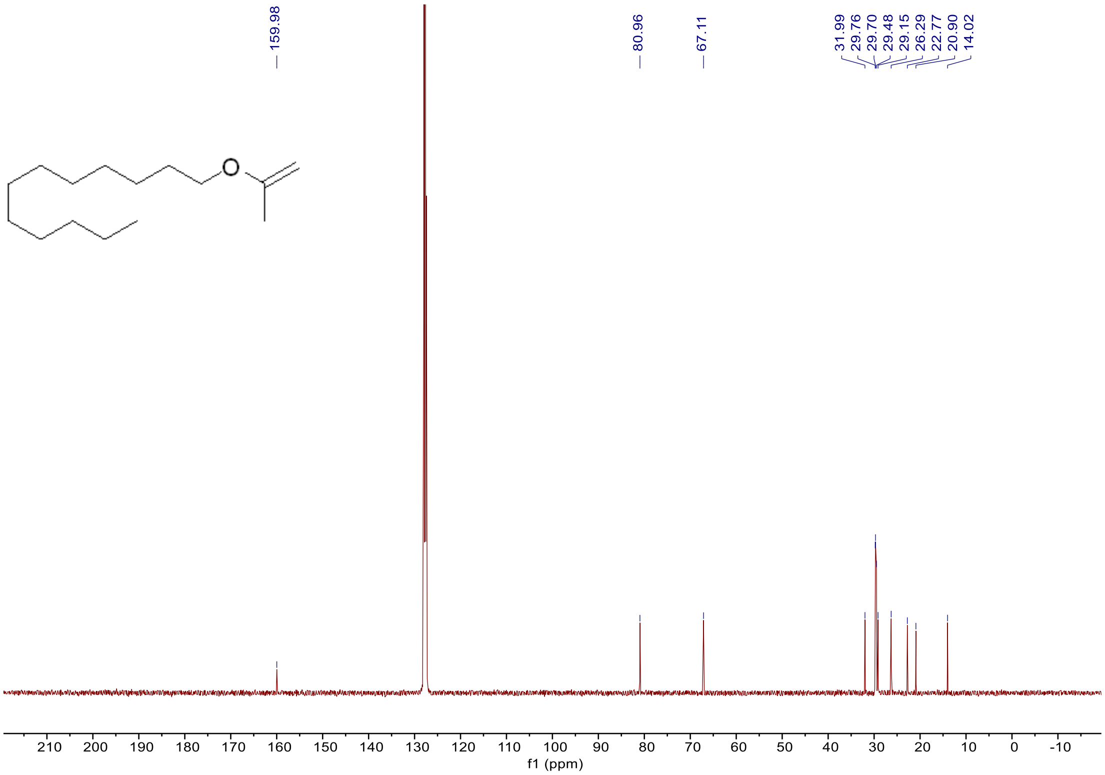

Supplementary Figure 106. ^{13}C NMR spectrum of compound **3ac** (100 MHz, C_6D_6)

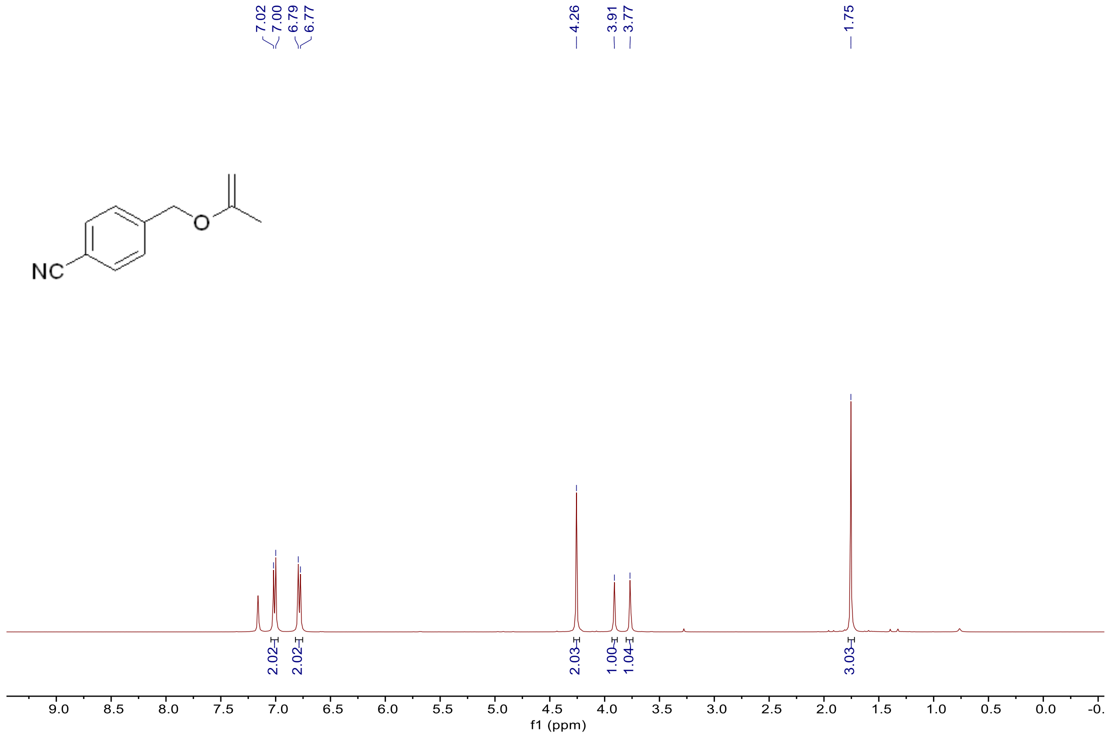

Supplementary Figure 107. ^{1}H NMR spectrum of compound **3ad** (400 MHz, C_6D_6)

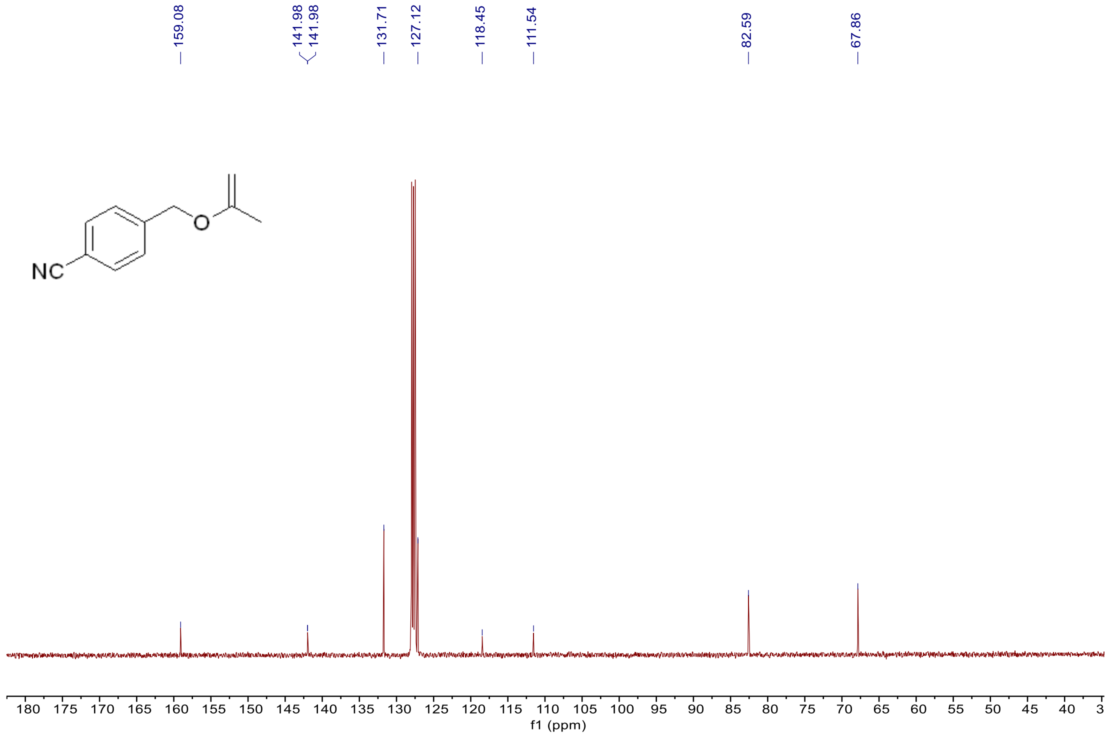

Supplementary Figure 108. ^{13}C NMR spectrum of compound **3ad** (100 MHz, C_6D_6)

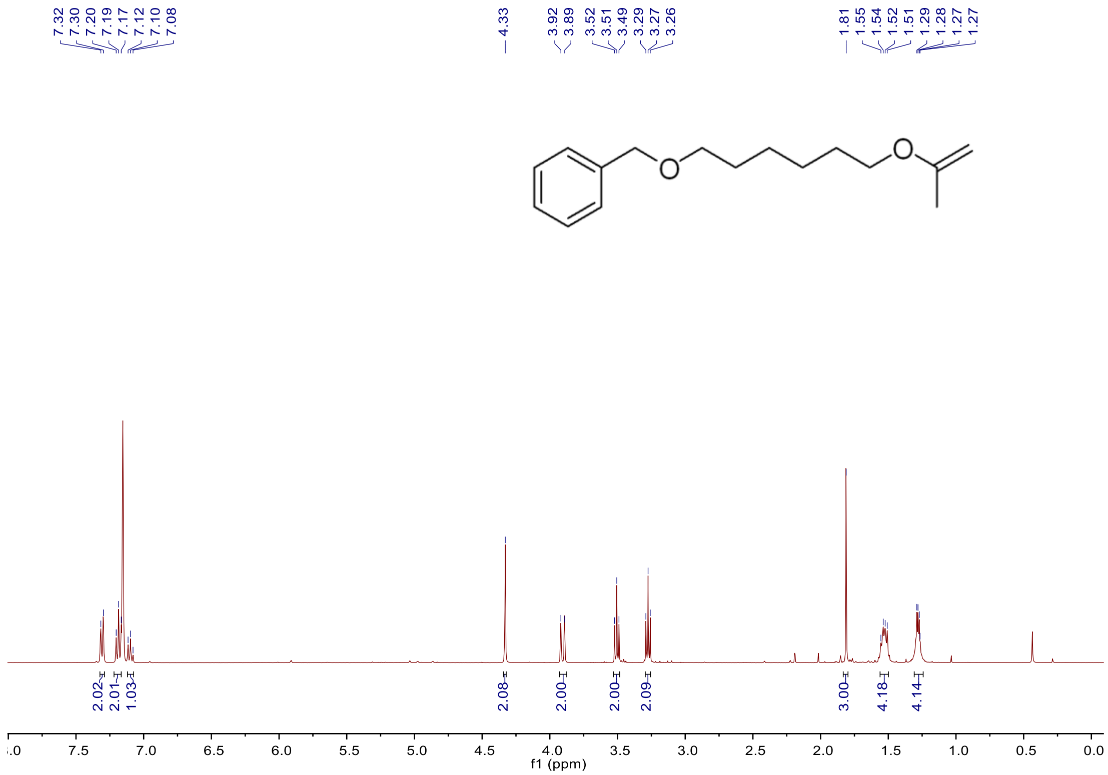

Supplementary Figure 109. ^{1}H NMR spectrum of compound **3ae** (400 MHz, C_6D_6)

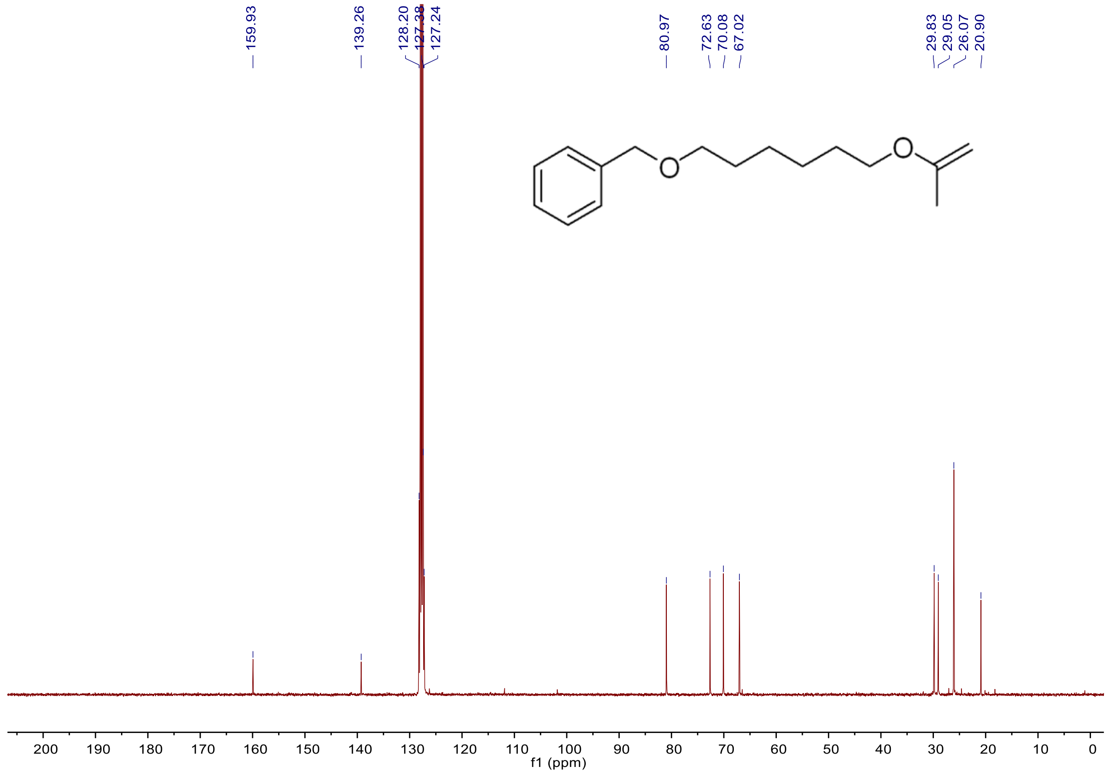

Supplementary Figure 110. ^{13}C NMR spectrum of compound **3ae** (100 MHz, C_6D_6)

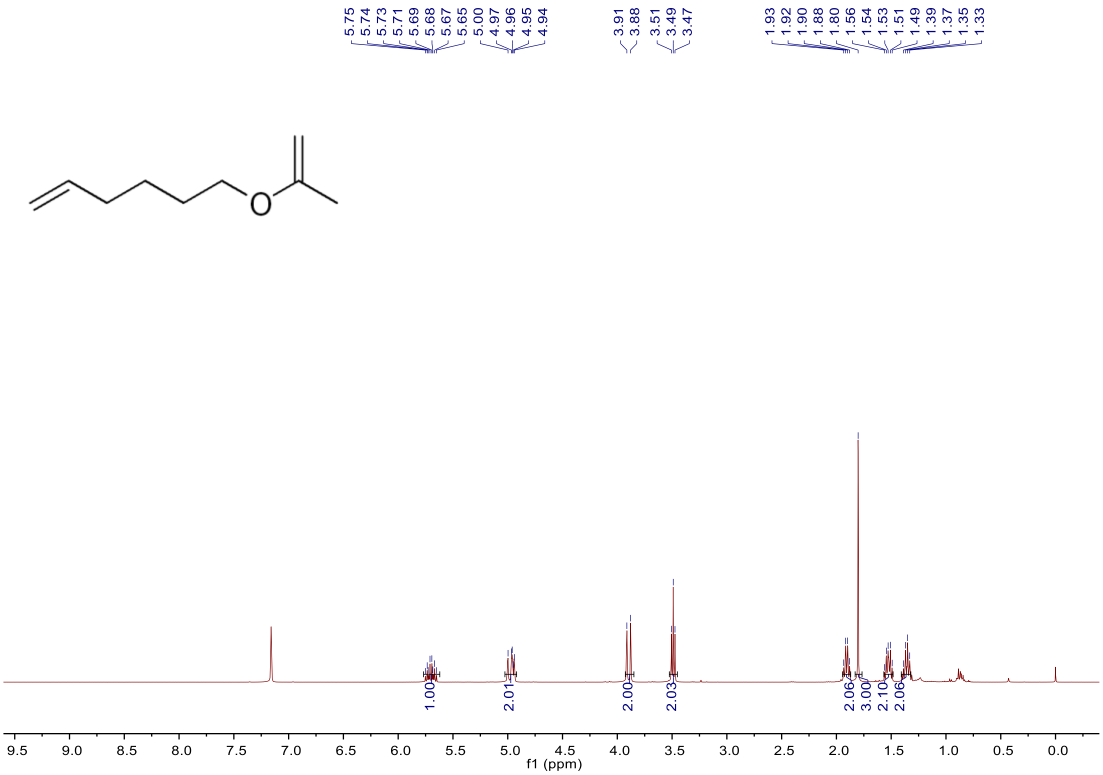

Supplementary Figure 111. ^{1}H NMR spectrum of compound **3af** (400 MHz, C_6D_6)

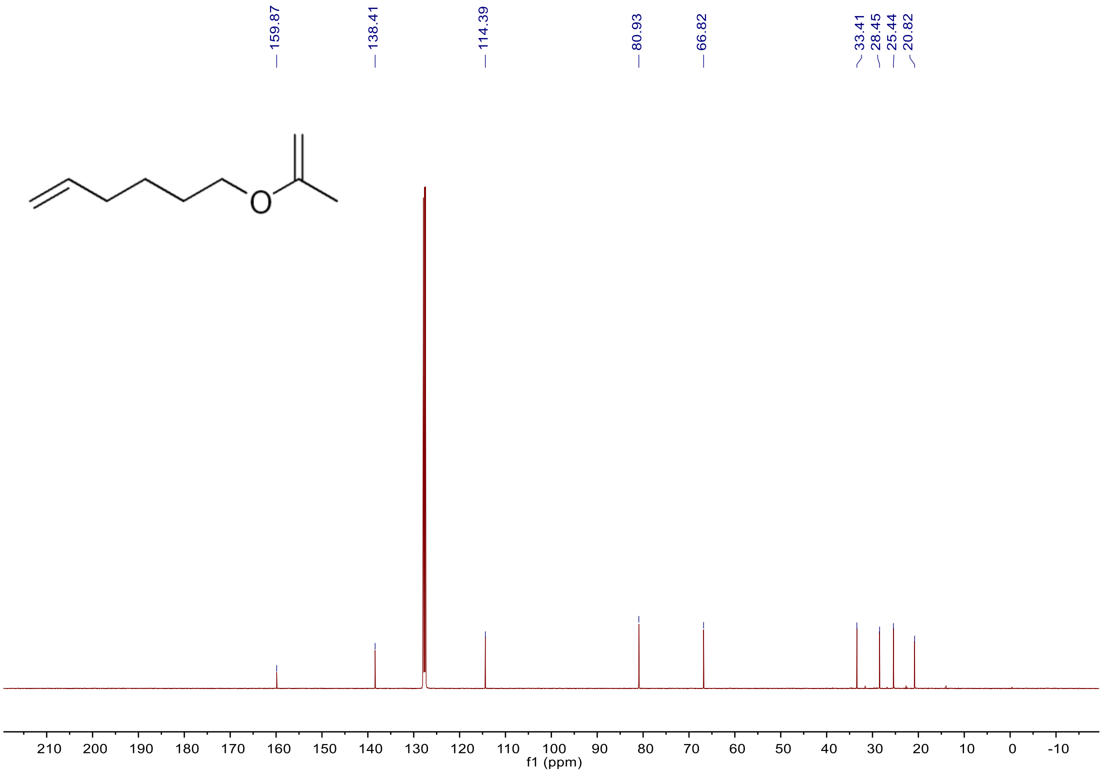

Supplementary Figure 112. ^{13}C NMR spectrum of compound **3af** (100 MHz, C_6D_6)

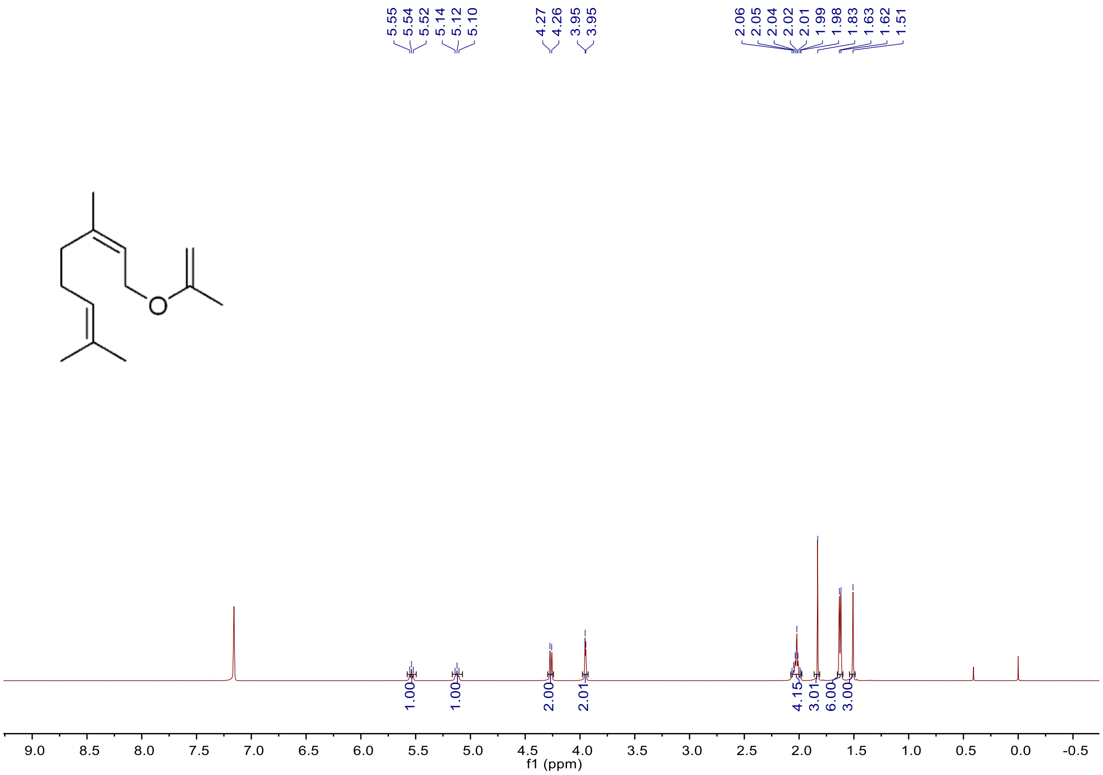

Supplementary Figure 113. ^{1}H NMR spectrum of compound **3ag** (400 MHz, C_6D_6)

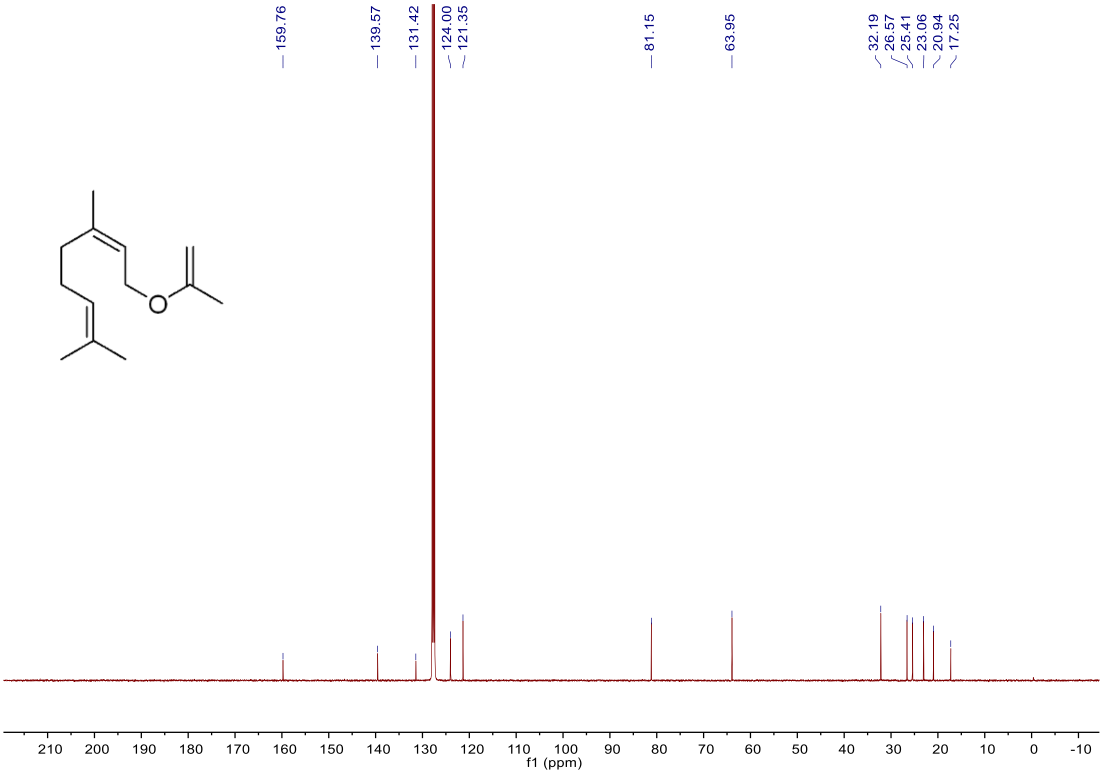

Supplementary Figure 114. ^{13}C NMR spectrum of compound **3ag** (100 MHz, C_6D_6)

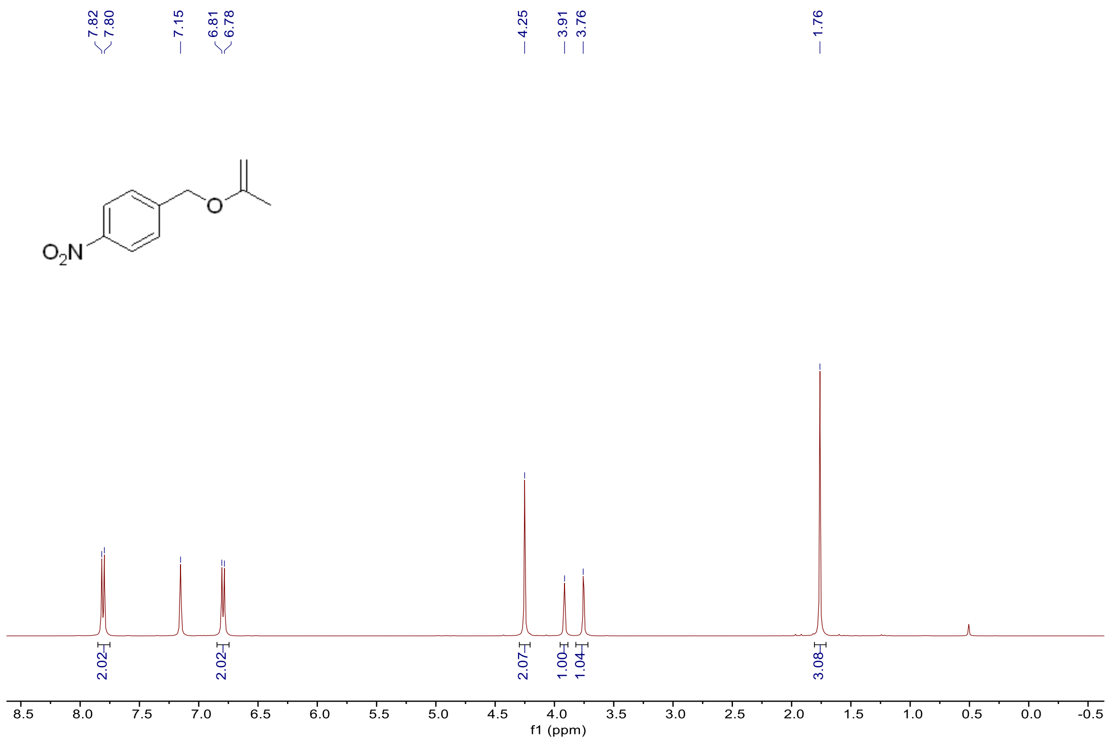

Supplementary Figure 115. ^{1}H NMR spectrum of compound **3ah** (400 MHz, C_6D_6)

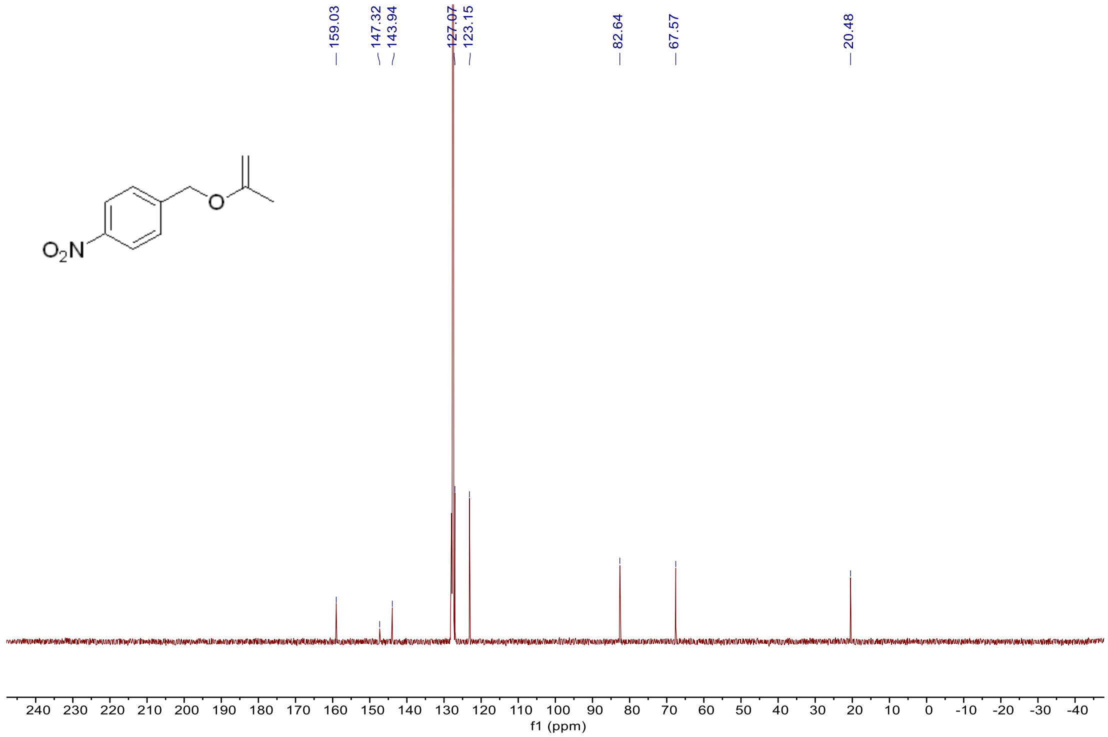

Supplementary Figure 116. ^{13}C NMR spectrum of compound **3ah** (100 MHz, C_6D_6)

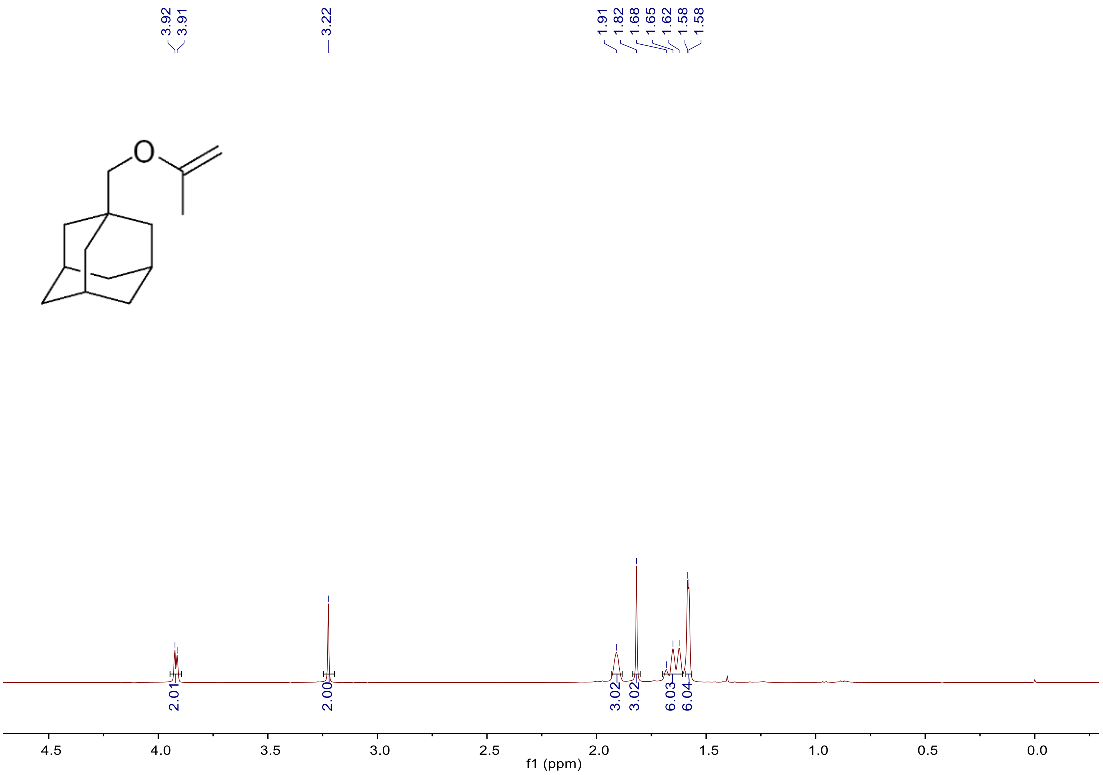

Supplementary Figure 117. ^{1}H NMR spectrum of compound **3ai** (400 MHz, C_6D_6)

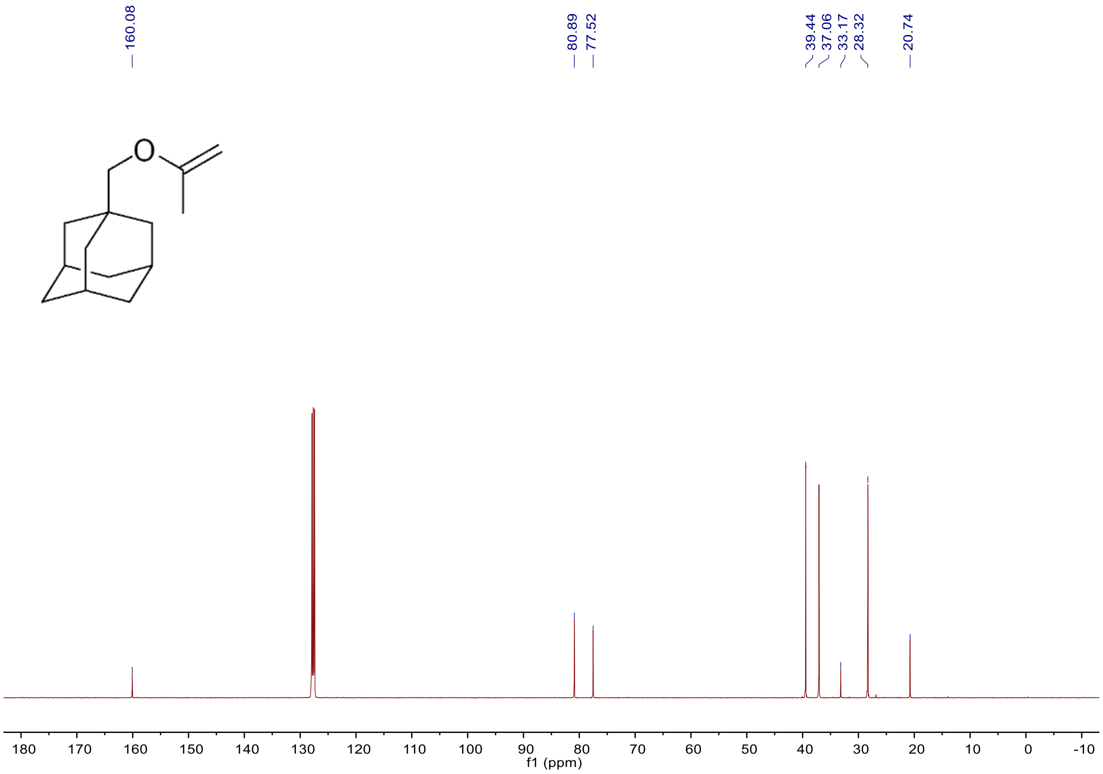

Supplementary Figure 118. ^{13}C NMR spectrum of compound **3ai** (100 MHz, C_6D_6)

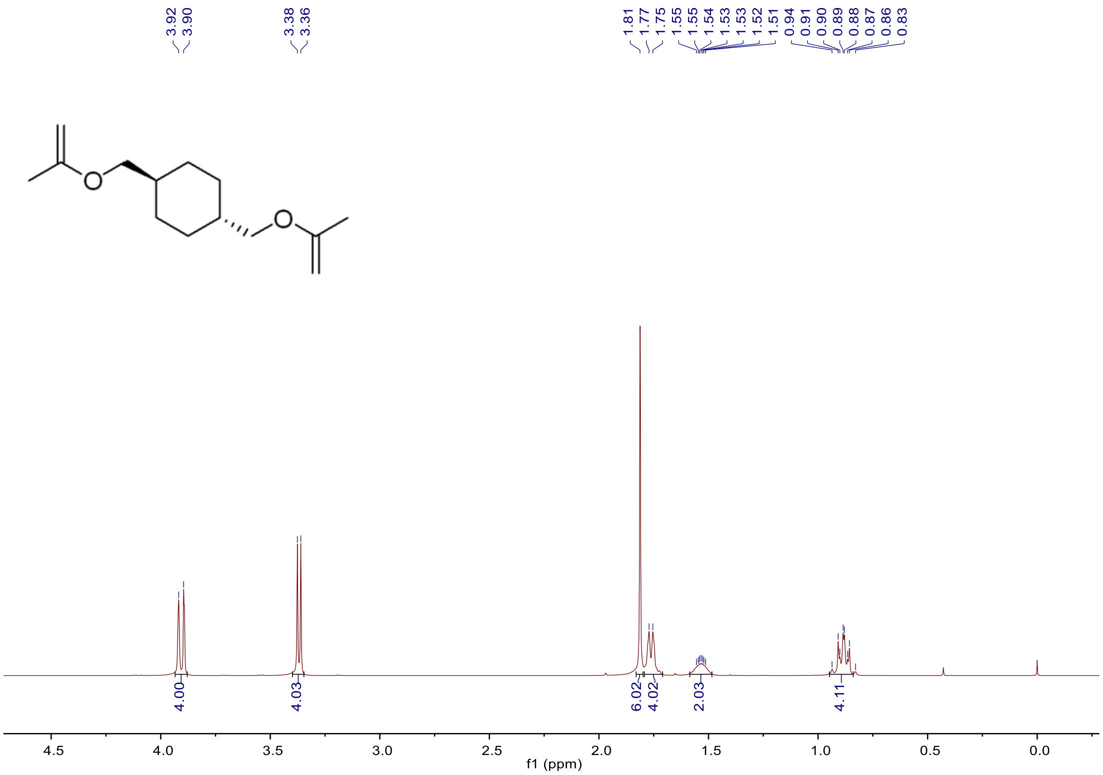

Supplementary Figure 119. ^{1}H NMR spectrum of compound **3aj** (400 MHz, C_6D_6)

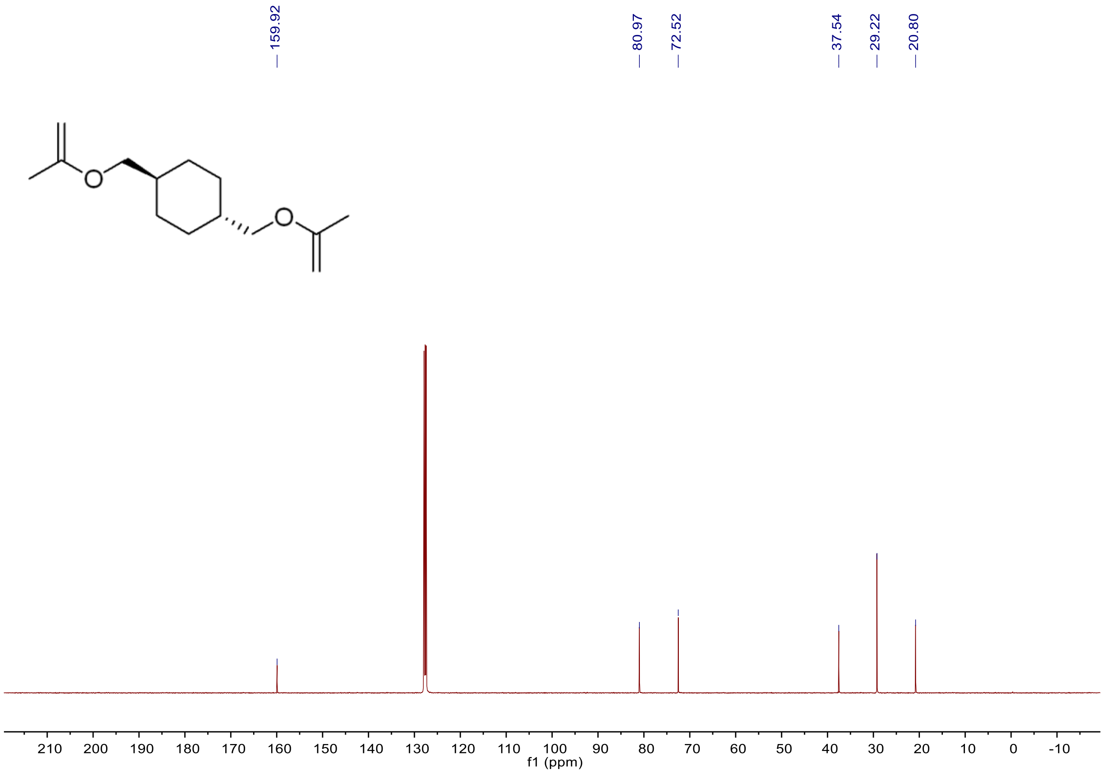

Supplementary Figure 120. ^{13}C NMR spectrum of compound **3aj** (100 MHz, C_6D_6)

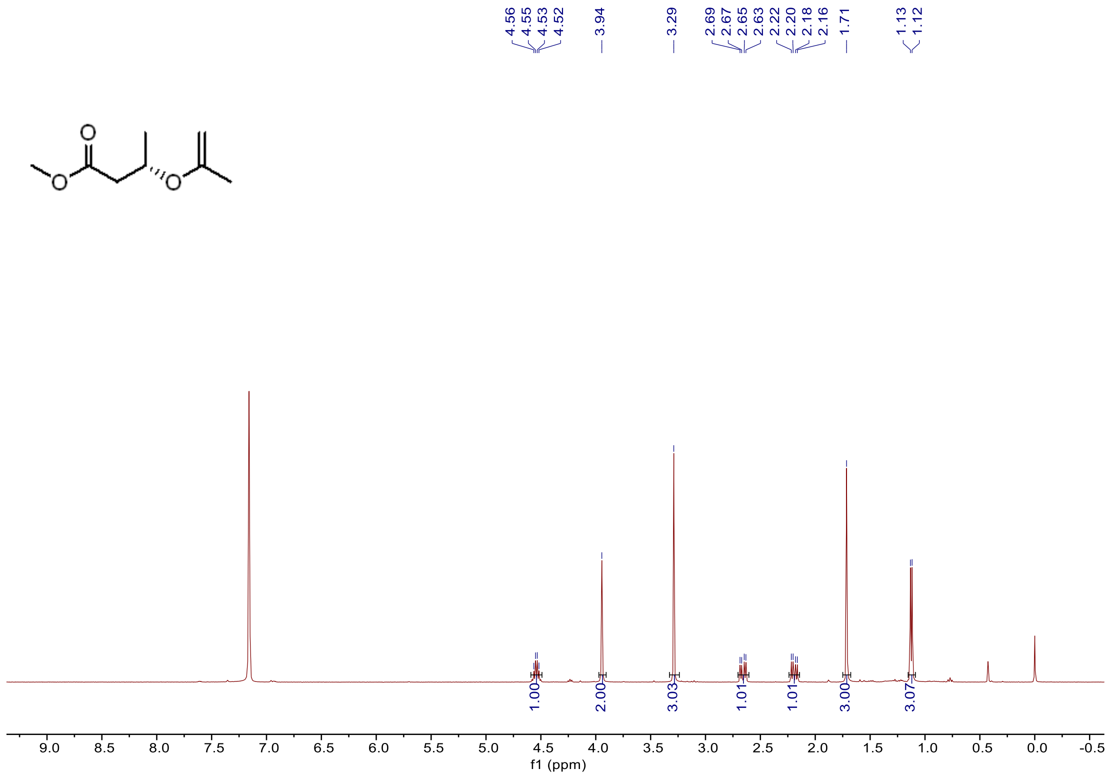

Supplementary Figure 121. ^{1}H NMR spectrum of compound **3ak** (400 MHz, C_6D_6)

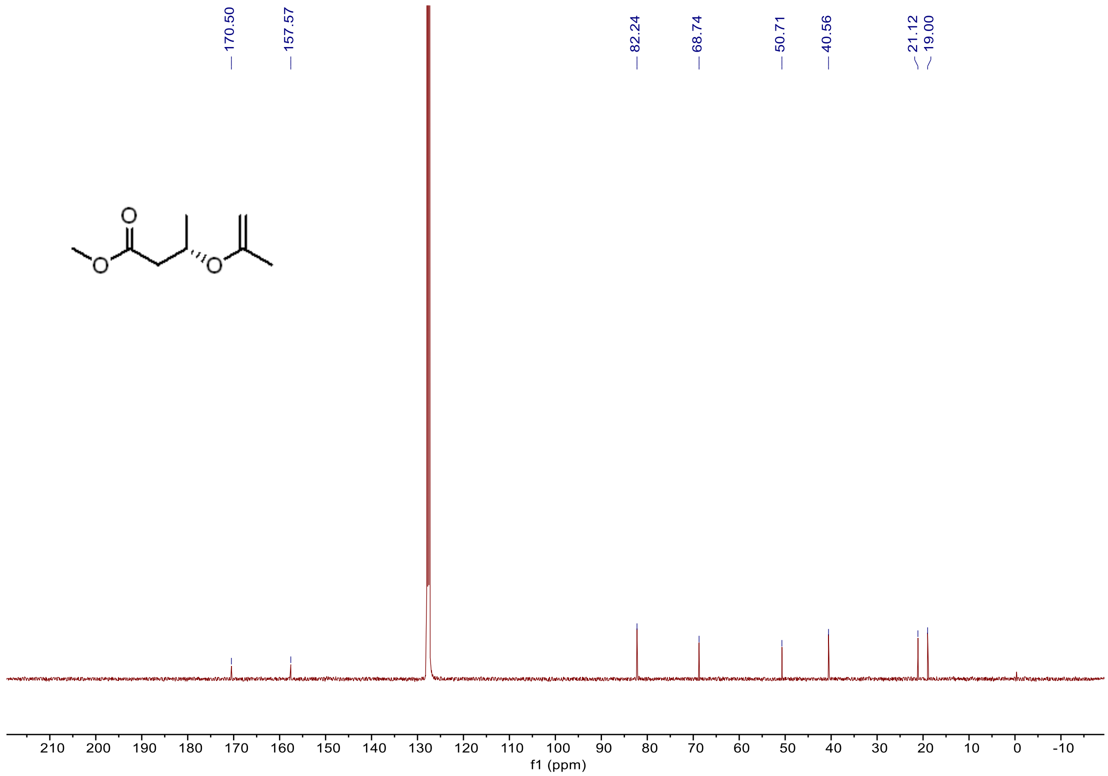

Supplementary Figure 122. ^{13}C NMR spectrum of compound **3ak** (100 MHz, C_6D_6)

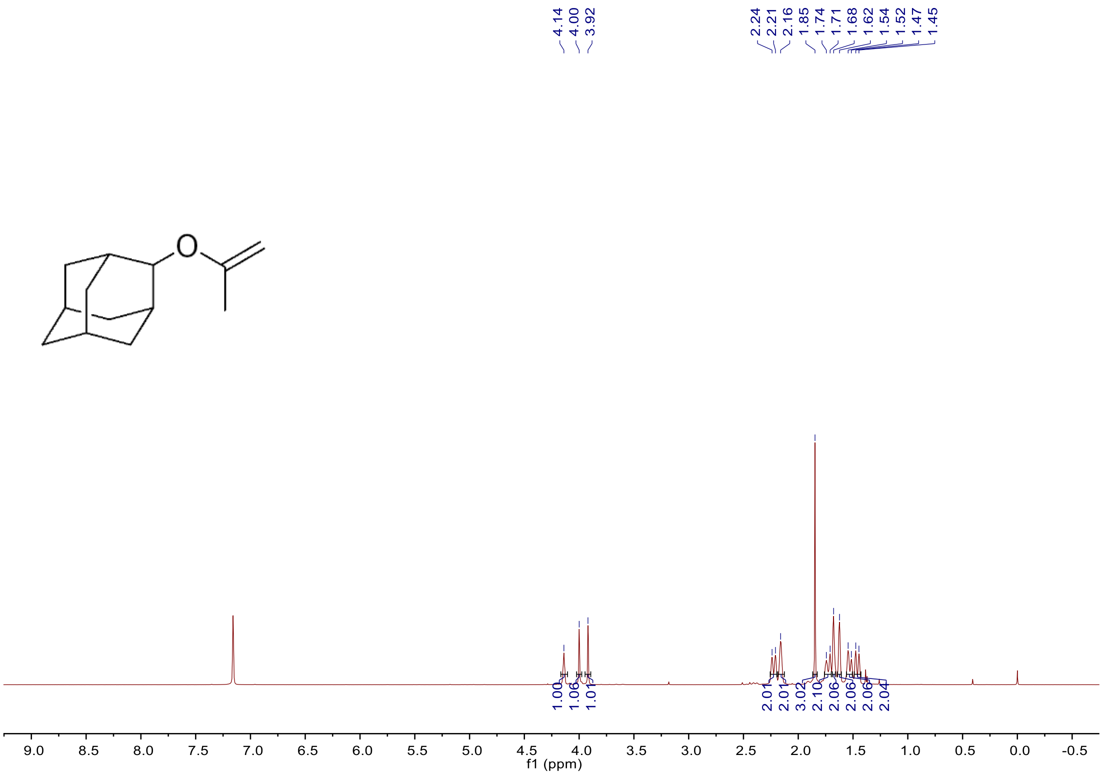

Supplementary Figure 123. ^{1}H NMR spectrum of compound **3al** (400 MHz, C_6D_6)

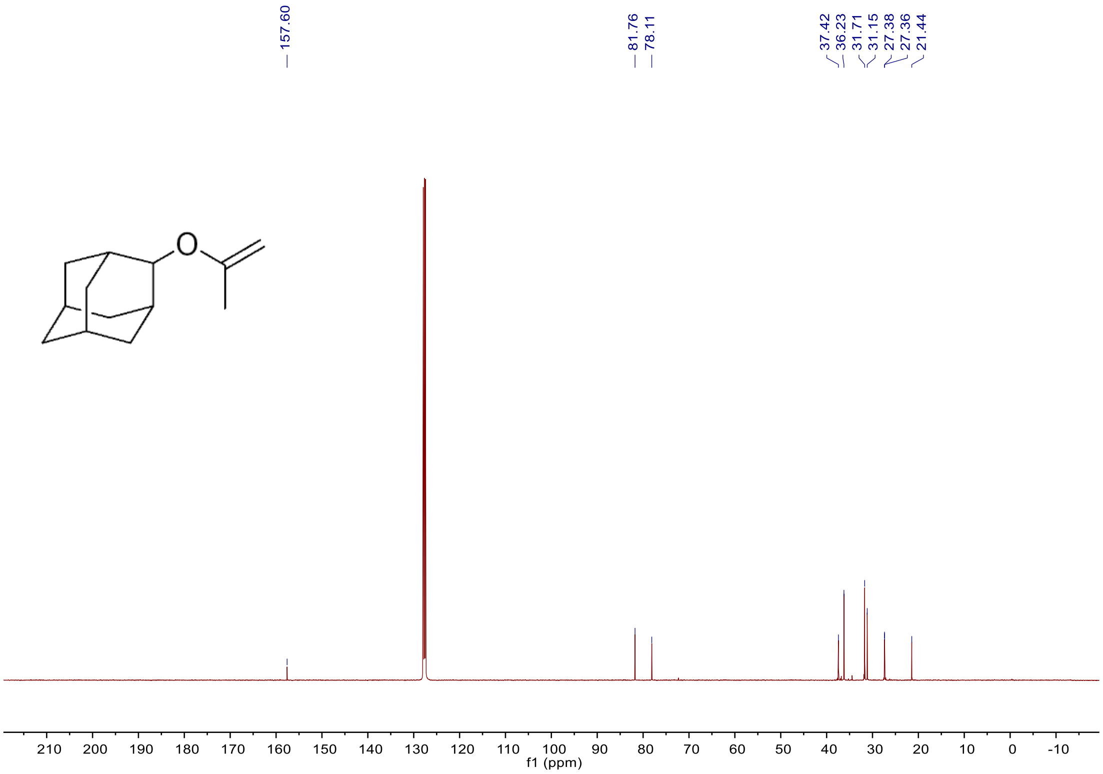

Supplementary Figure 124. ^{13}C NMR spectrum of compound **3al** (100 MHz, C_6D_6)

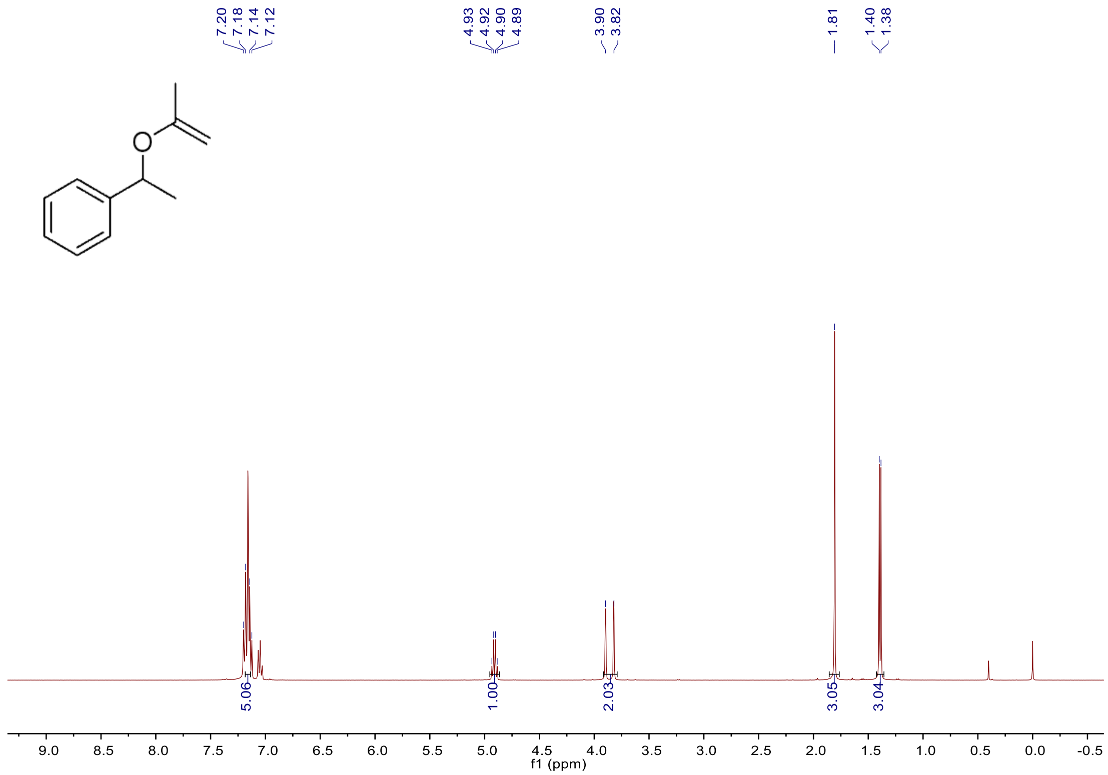

Supplementary Figure 125. ^{1}H NMR spectrum of compound **3am** (400 MHz, C_6D_6)

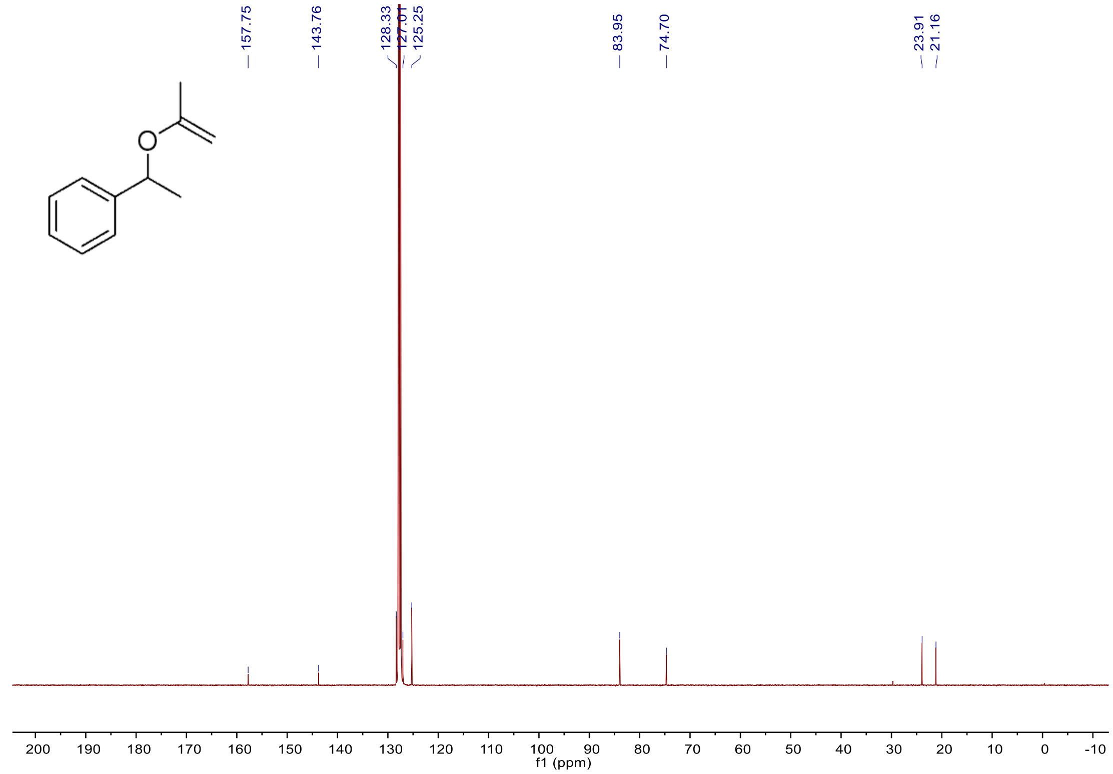

Supplementary Figure 126. ^{13}C NMR spectrum of compound **3am** (100 MHz, C_6D_6)

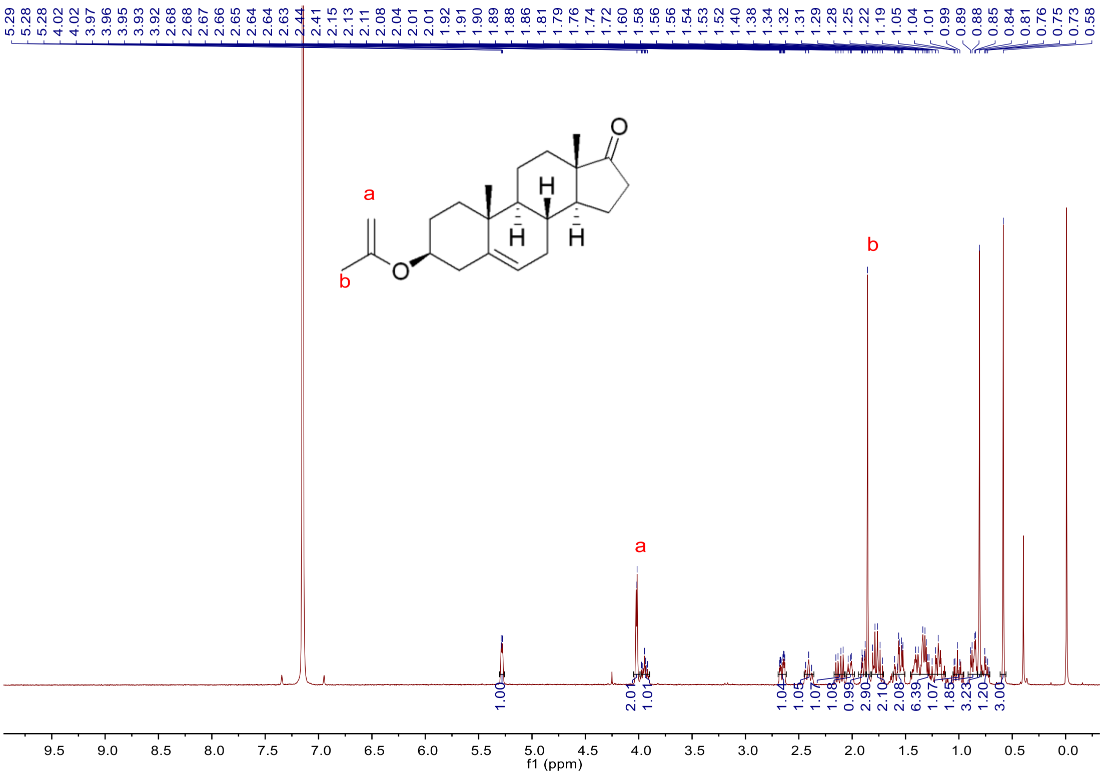

Supplementary Figure 127. ^{1}H NMR spectrum of compound **3an** (400 MHz, C_6D_6)

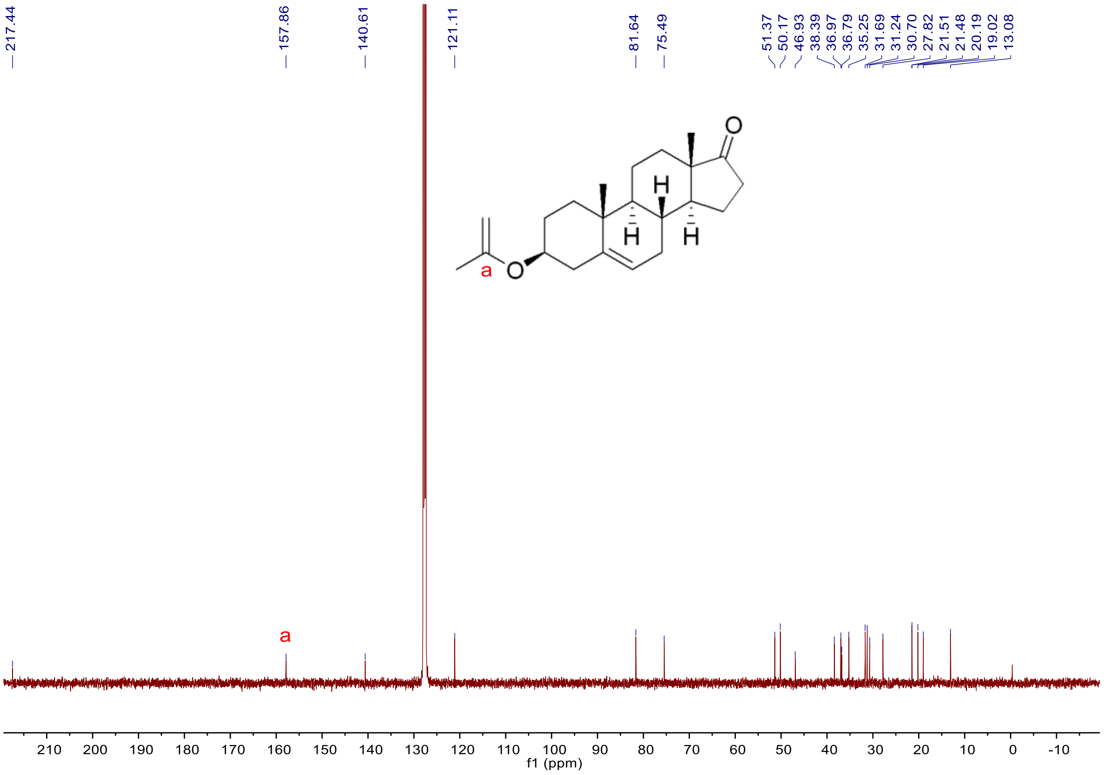

Supplementary Figure 128. ^{13}C NMR spectrum of compound **3an** (100 MHz, C_6D_6)

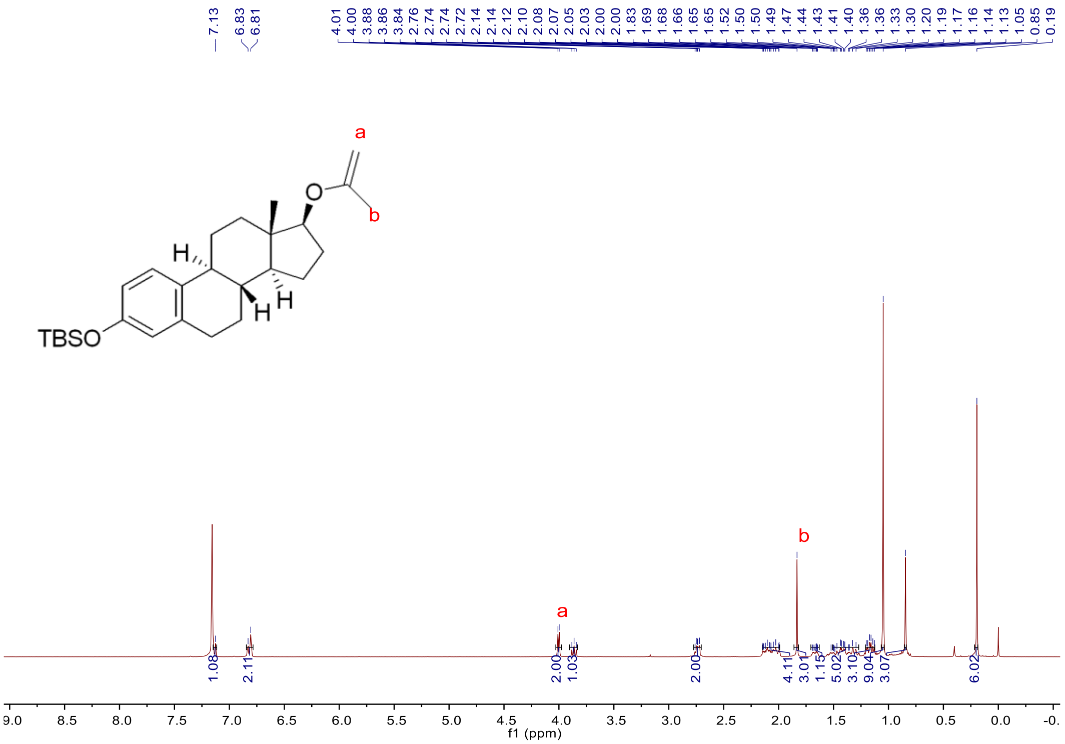

Supplementary Figure 129. ^{1}H NMR spectrum of compound **EST-3-TBS-17-IPPE 3ao** (400 MHz, C_6D_6)

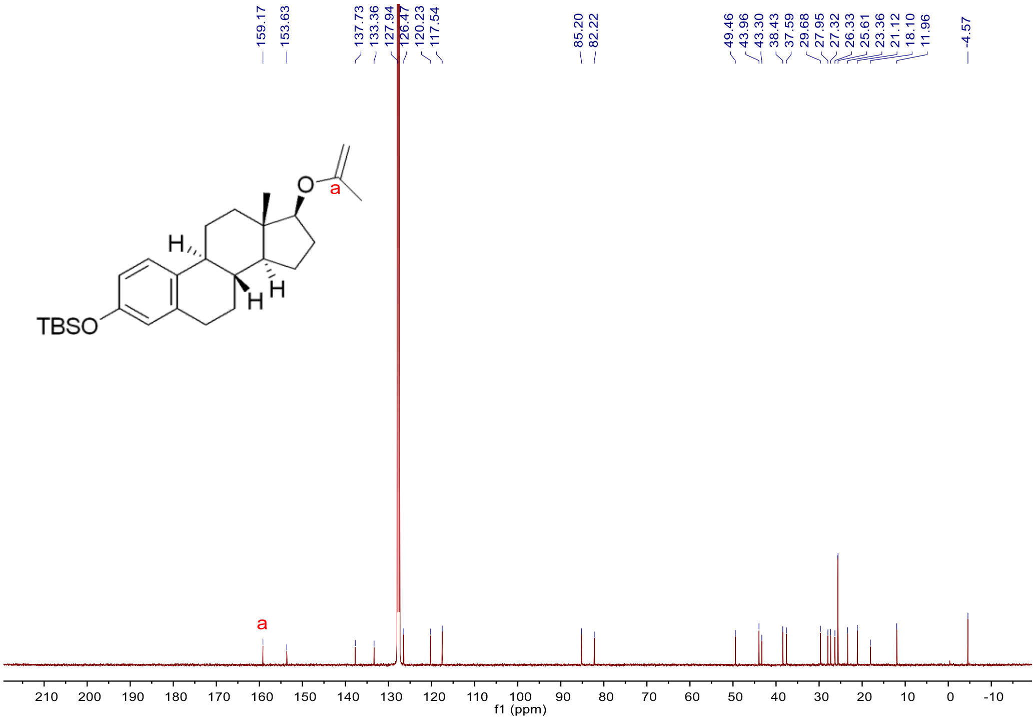

Supplementary Figure 130. ^{13}C NMR spectrum of compound **EST-3-TBS-17-IPPE 3ao** (100 MHz, C_6D_6)

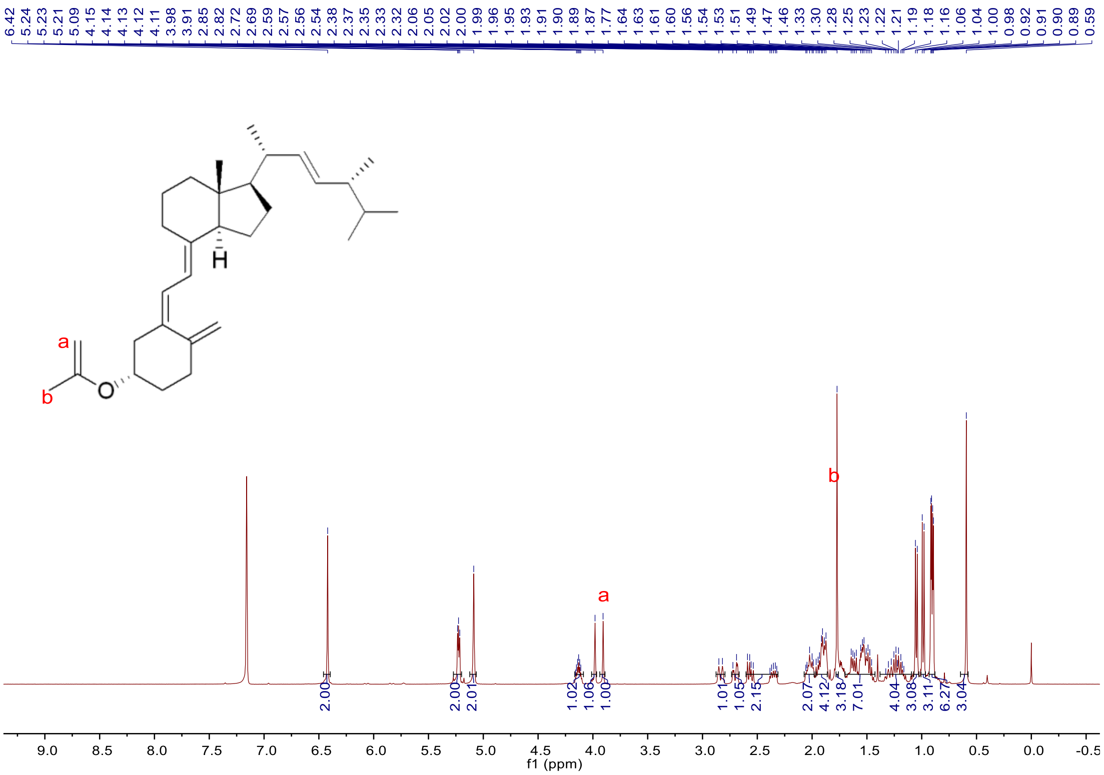

Supplementary Figure 131. ^{1}H NMR spectrum of compound **3ap** (400 MHz, C_6D_6)

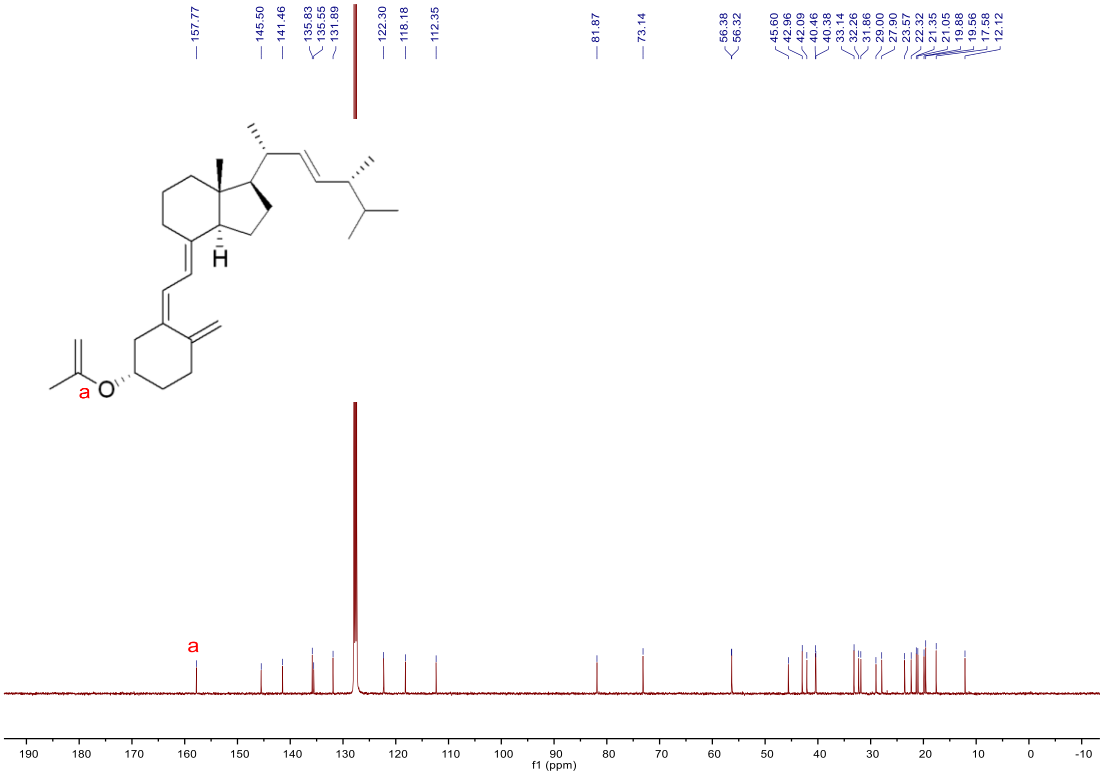

Supplementary Figure 132. ^{13}C NMR spectrum of compound **3ap** (100 MHz, C_6D_6)

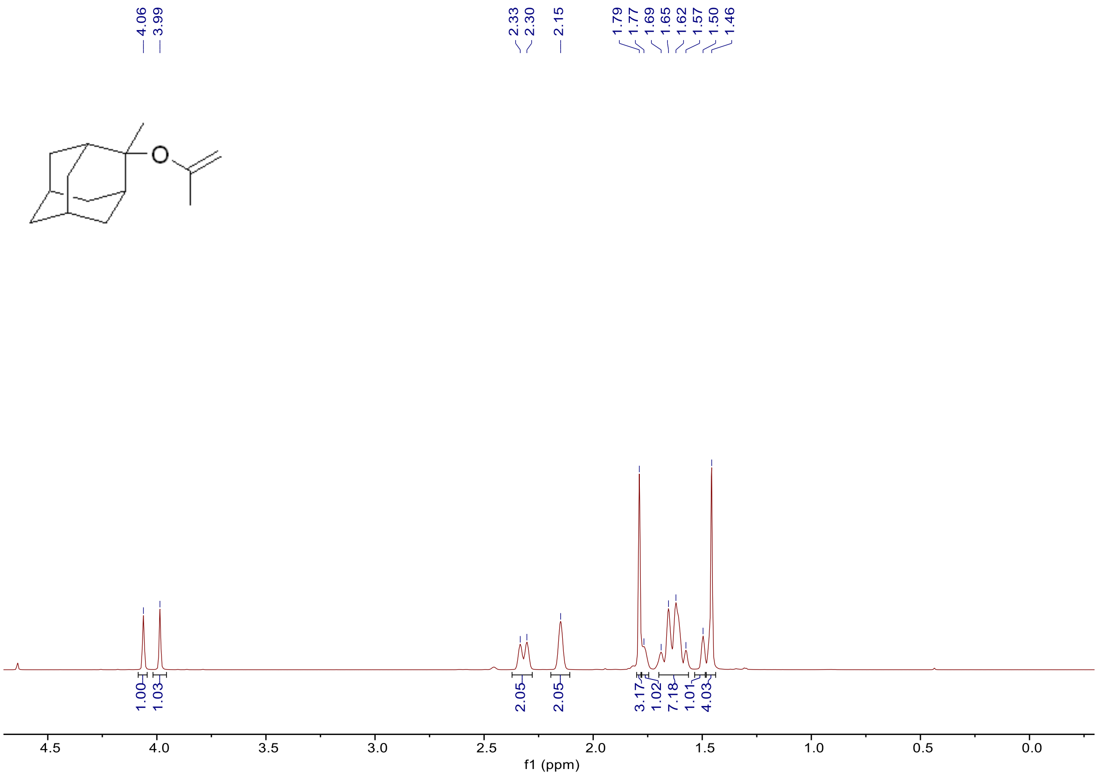

Supplementary Figure 133. ^{1}H NMR spectrum of compound **3aq** (400 MHz, C_6D_6)

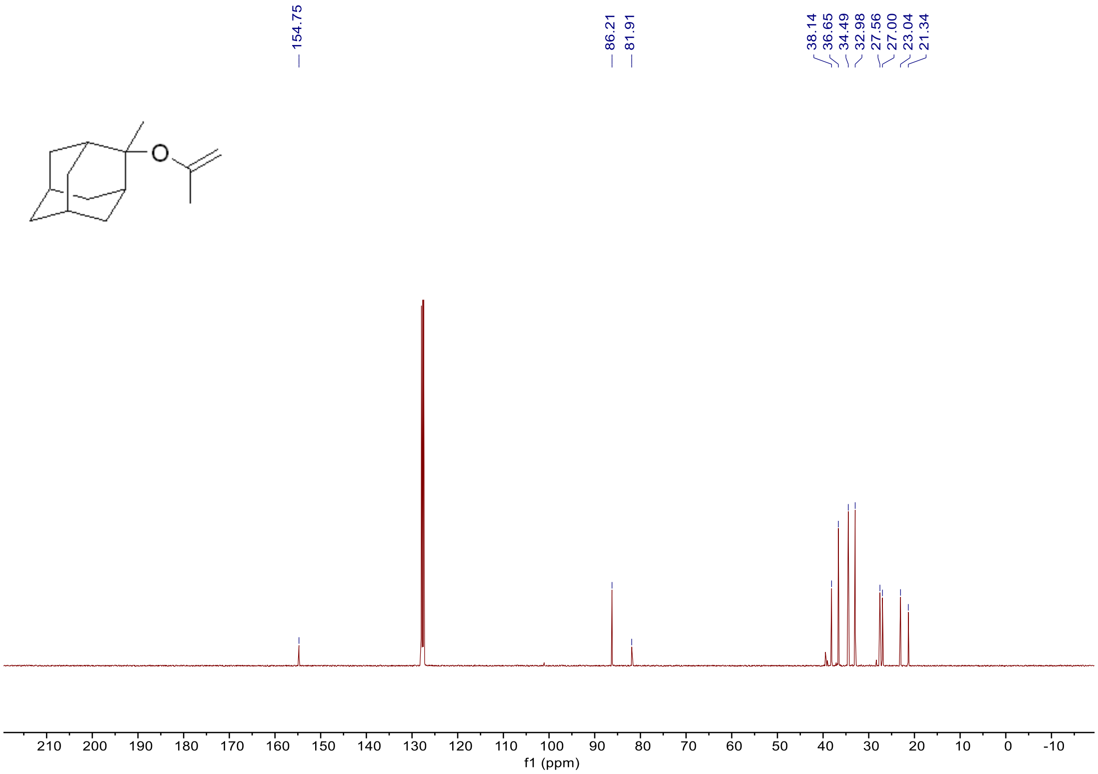

Supplementary Figure 134. ^{13}C NMR spectrum of compound **3aq** (100 MHz, C_6D_6)

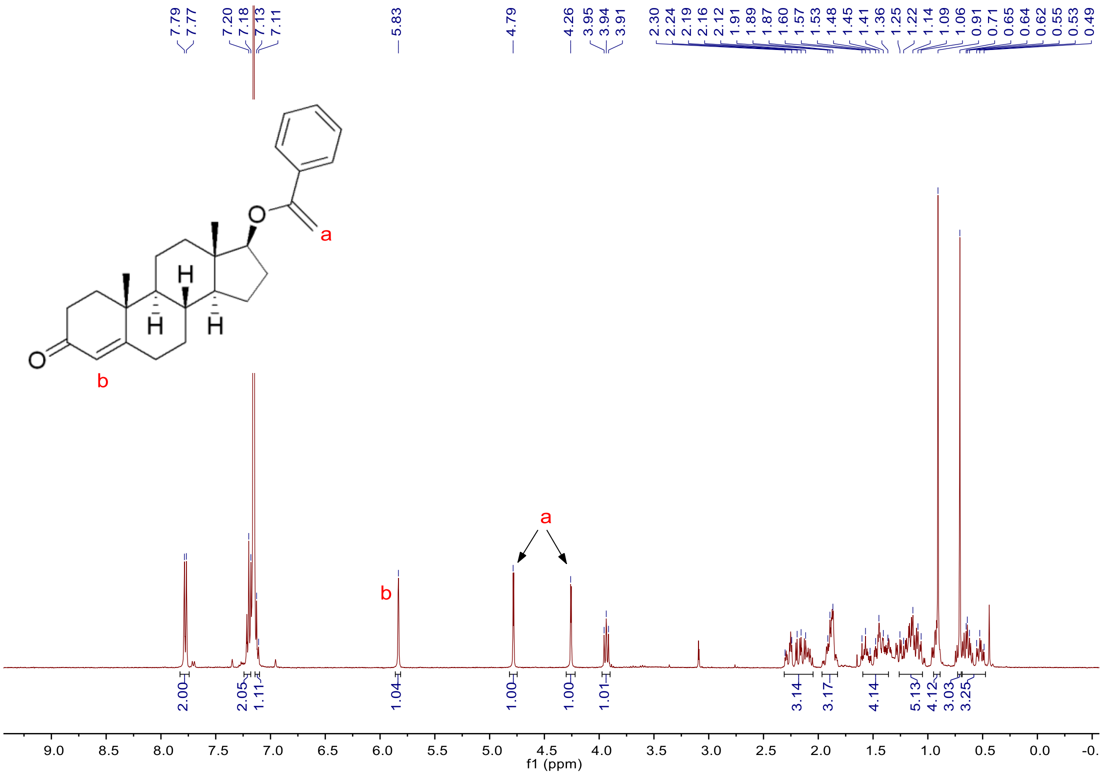

Supplementary Figure 135. ^{1}H NMR spectrum of compound **5a** (400 MHz, C_6D_6)

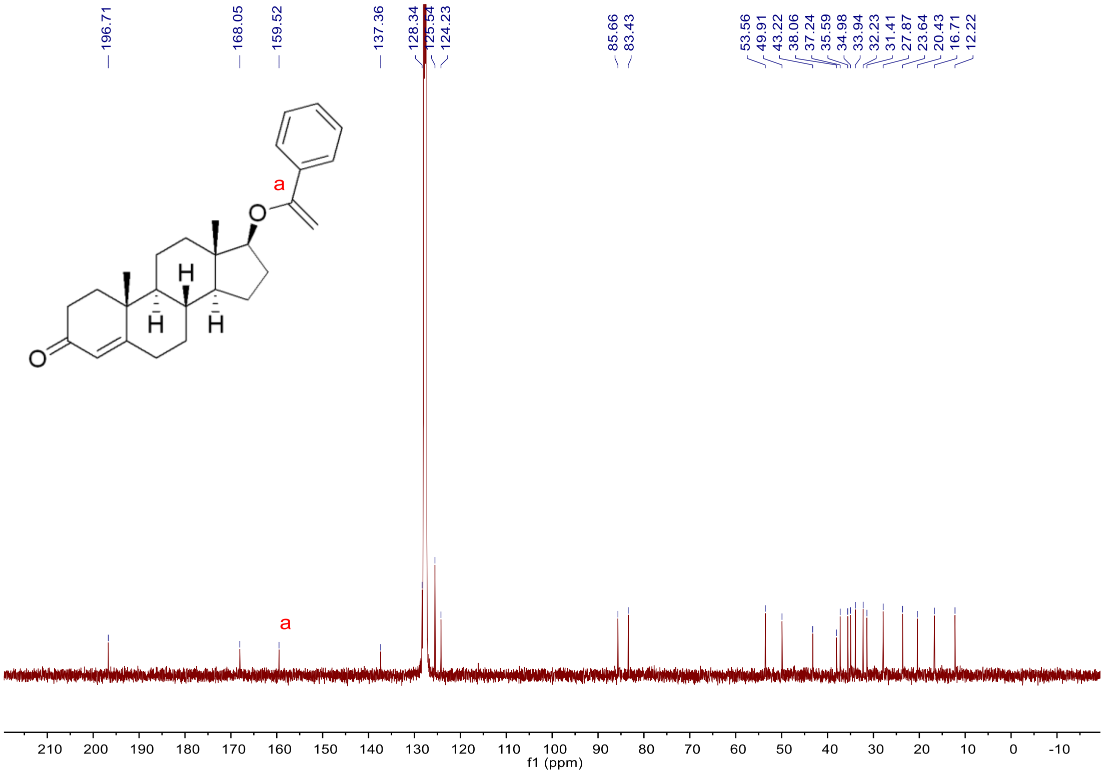

Supplementary Figure 136. ^{13}C NMR spectrum of compound **5a** (100 MHz, C_6D_6)

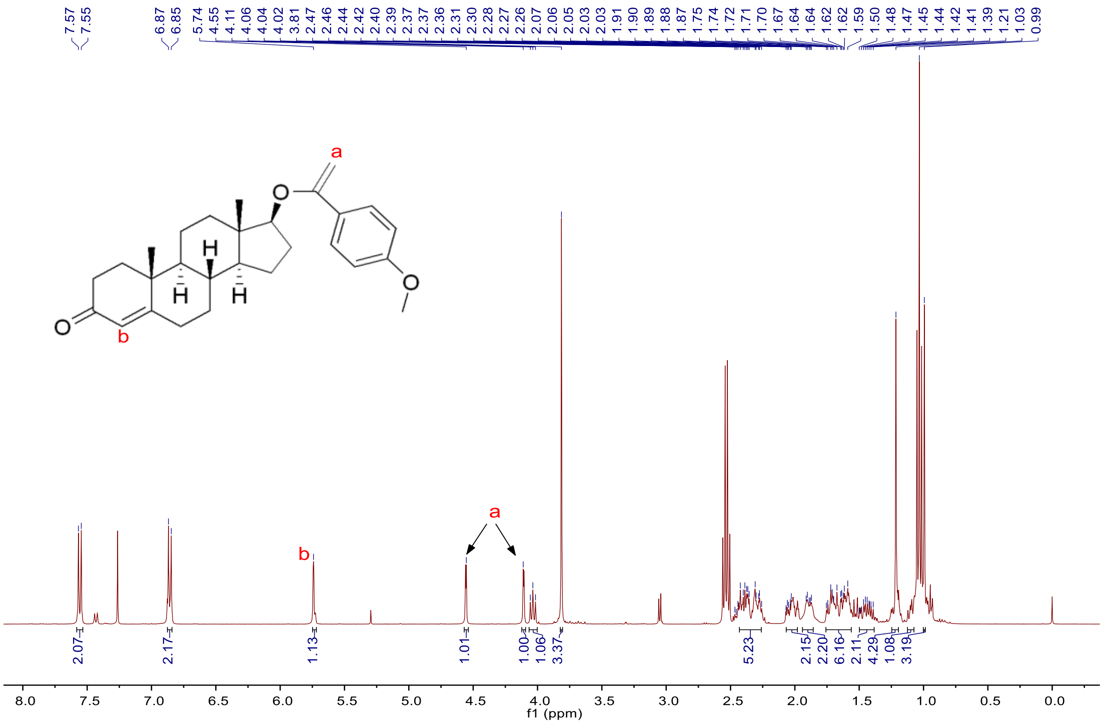

Supplementary Figure 137. ^{1}H NMR spectrum of compound **5b** (400 MHz, $CDCl_3$)

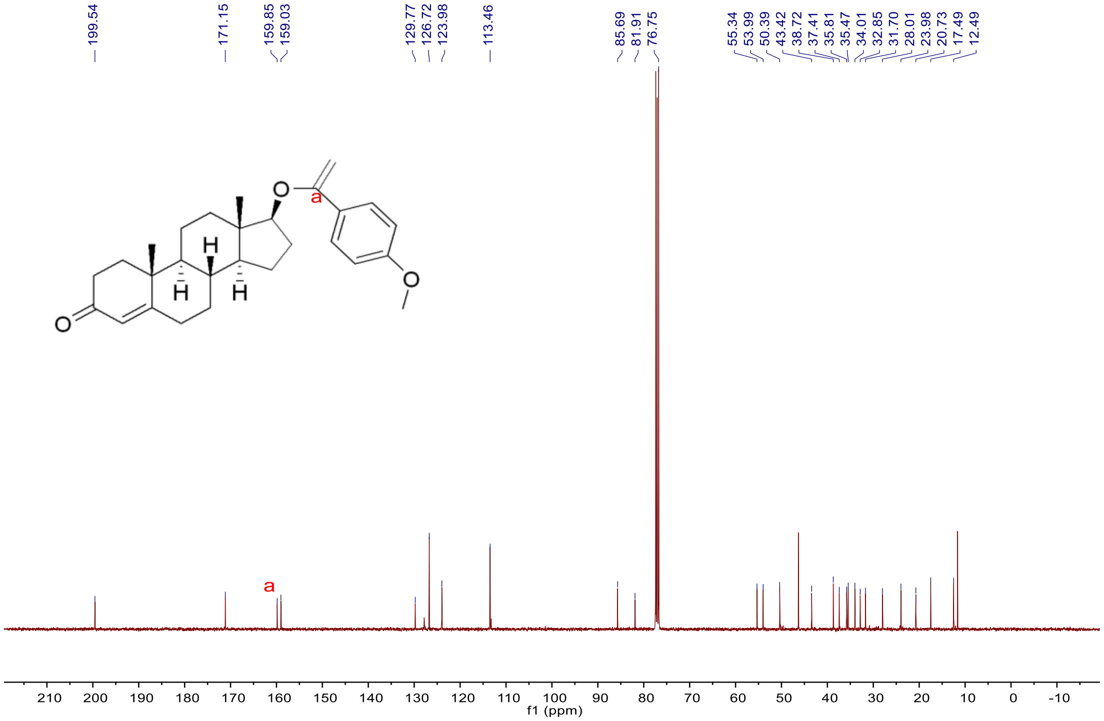

Supplementary Figure 138. ^{13}C NMR spectrum of compound **5b** (100 MHz, $CDCl_3$)

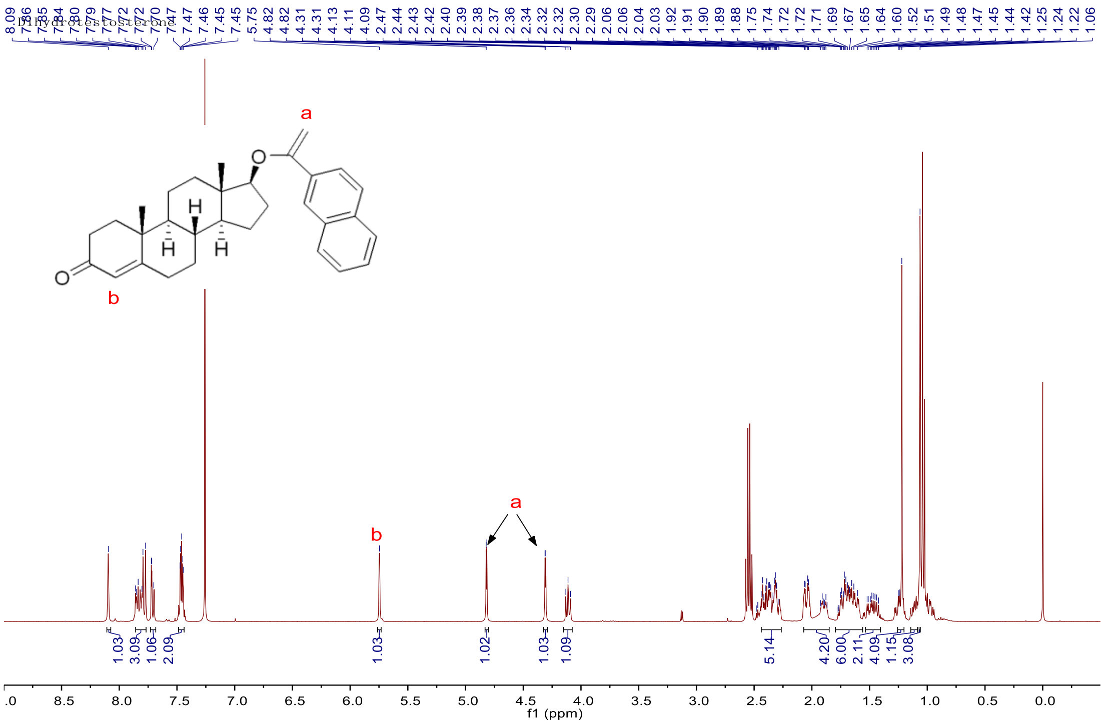

Supplementary Figure 139. ^{1}H NMR spectrum of compound **5c** (400 MHz, $CDCl_3$)

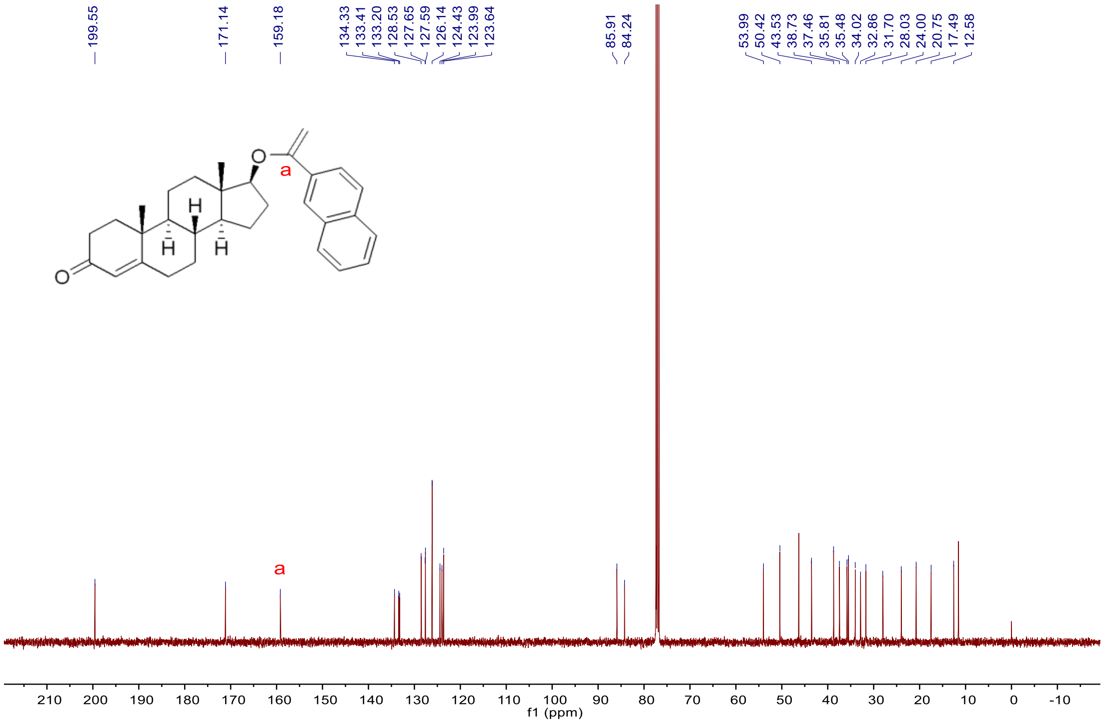

Supplementary Figure 140. ^{13}C NMR spectrum of compound **5c** (100 MHz, $CDCl_3$)

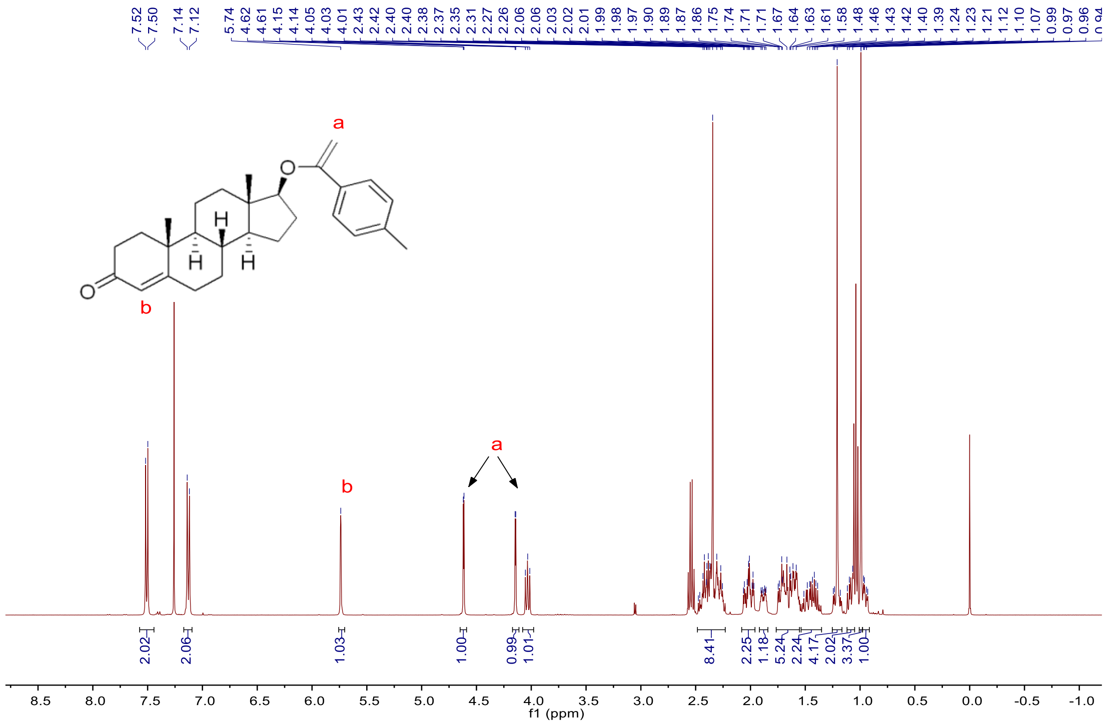

Supplementary Figure 141. ^{1}H NMR spectrum of compound **5d** (400 MHz, $CDCl_3$)

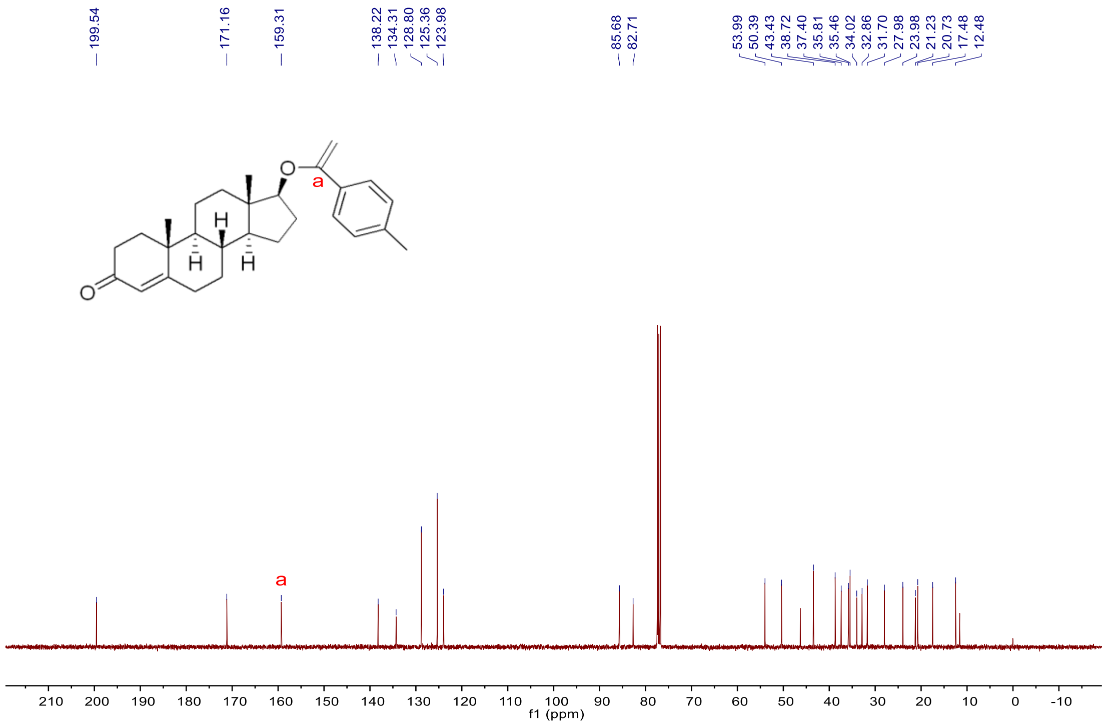

Supplementary Figure 142. ^{13}C NMR spectrum of compound **5d** (100 MHz, $CDCl_3$)

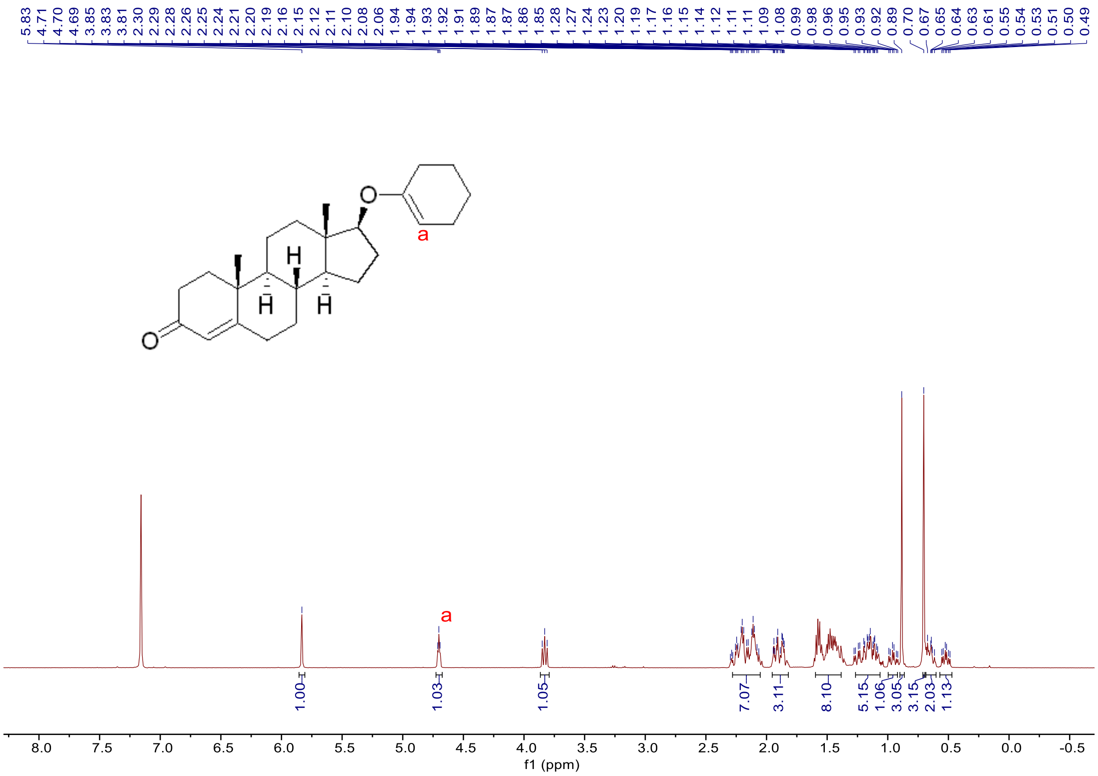

Supplementary Figure 143. ^{1}H NMR spectrum of compound **5e** (400 MHz, C_6D_6)

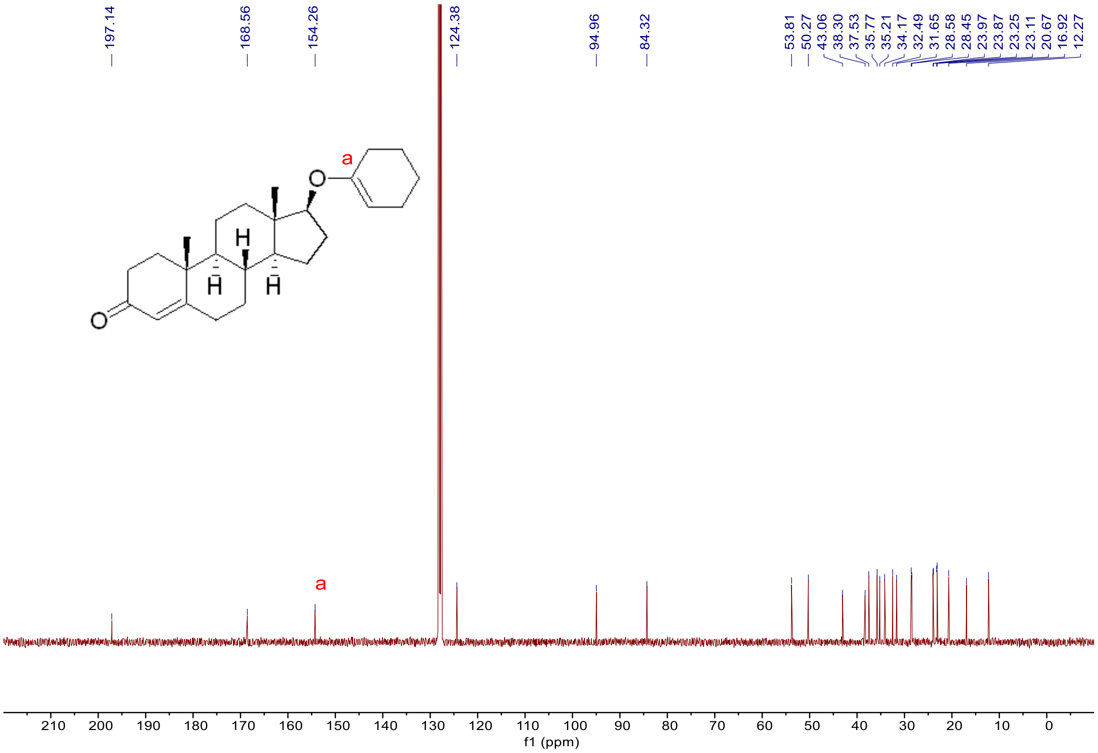

Supplementary Figure 144. ^{13}C NMR spectrum of compound **5e** (100 MHz, C_6D_6)

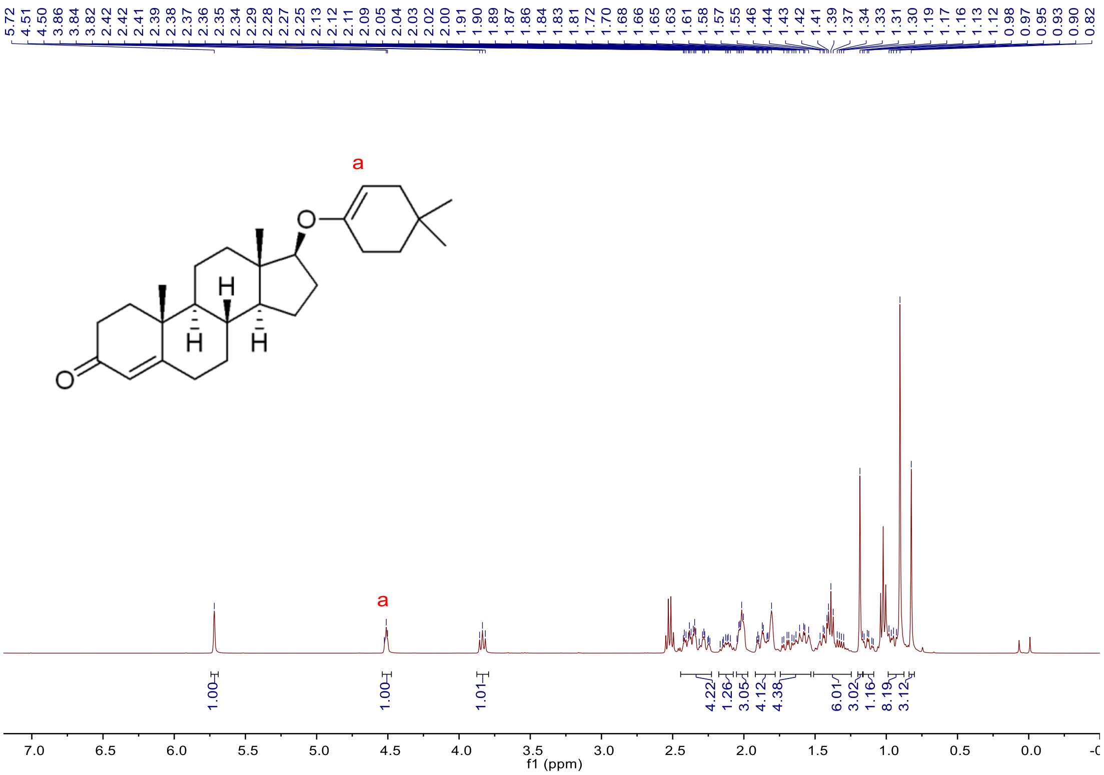

Supplementary Figure 145. ^{1}H NMR spectrum of compound **5f** (400 MHz, $CDCl_3$)

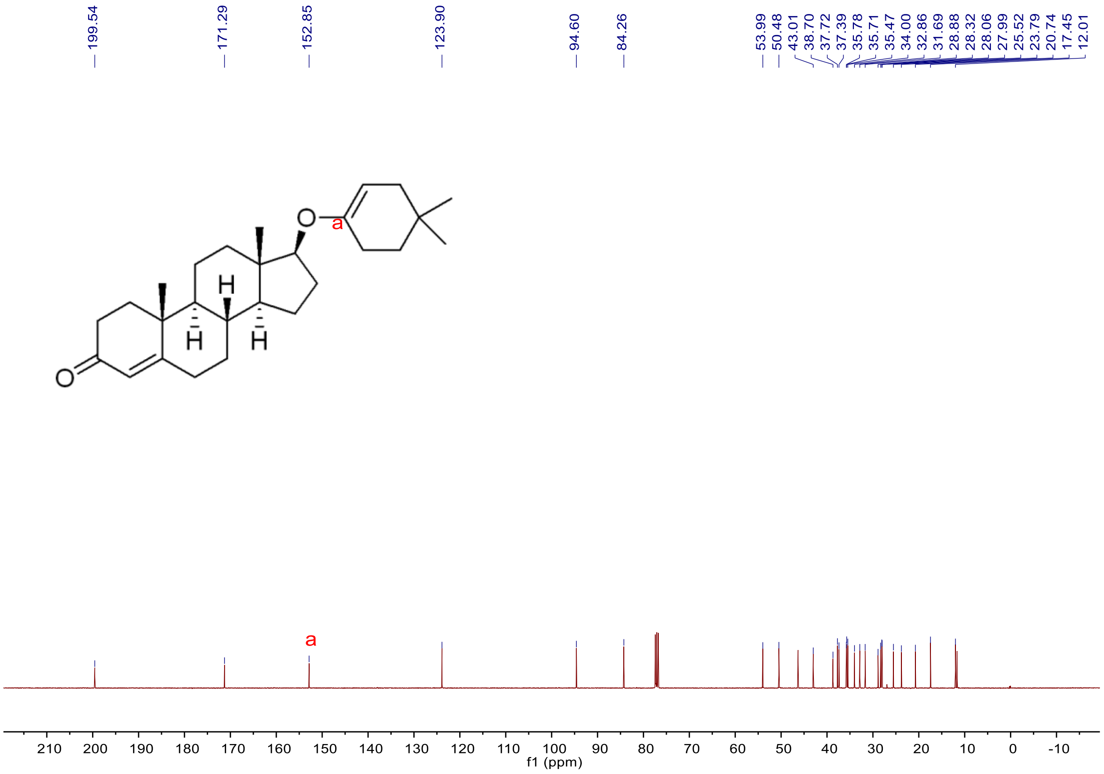

Supplementary Figure 146. ^{13}C NMR spectrum of compound **5f** (100 MHz, $CDCl_3$)

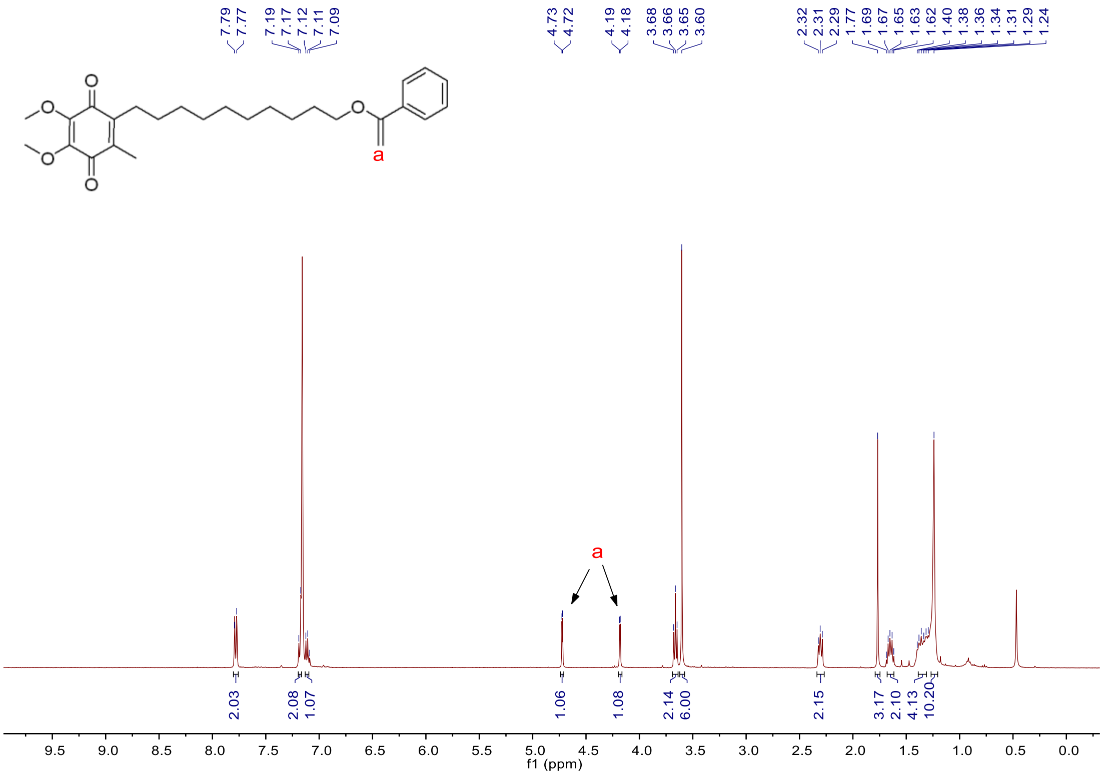

Supplementary Figure 147. ^{1}H NMR spectrum of compound **5g** (400 MHz, C_6D_6)

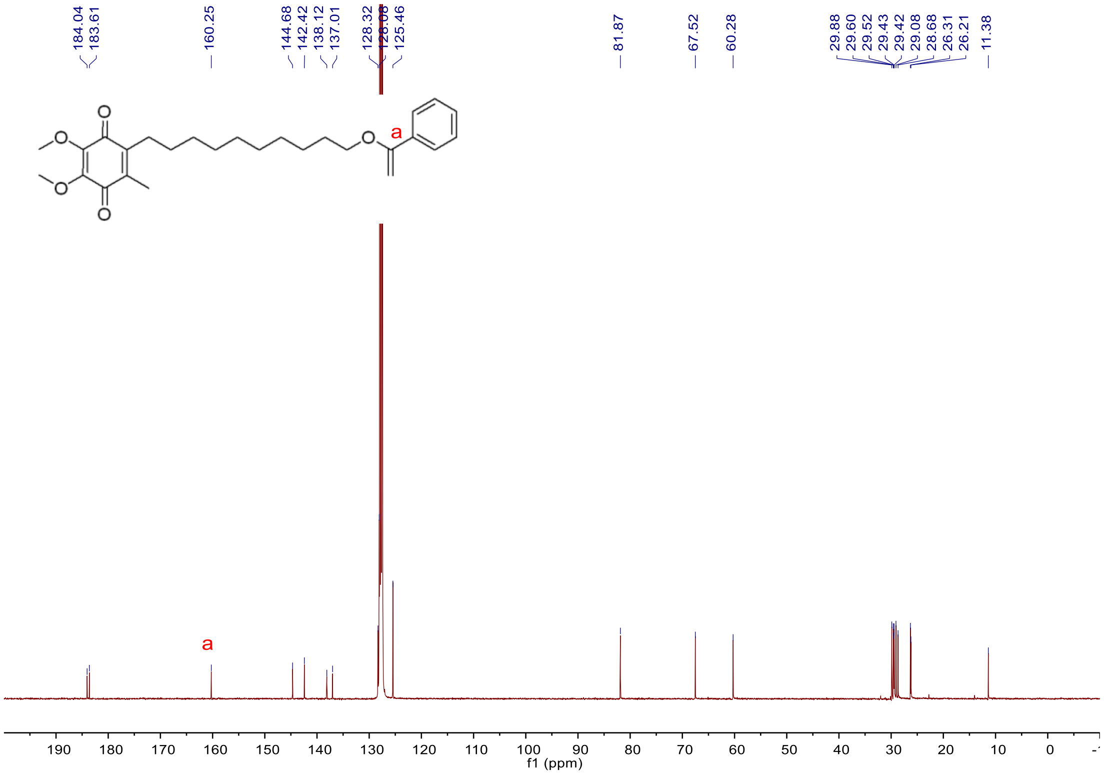

Supplementary Figure 148. ^{13}C NMR spectrum of compound **5g** (100 MHz, C_6D_6)

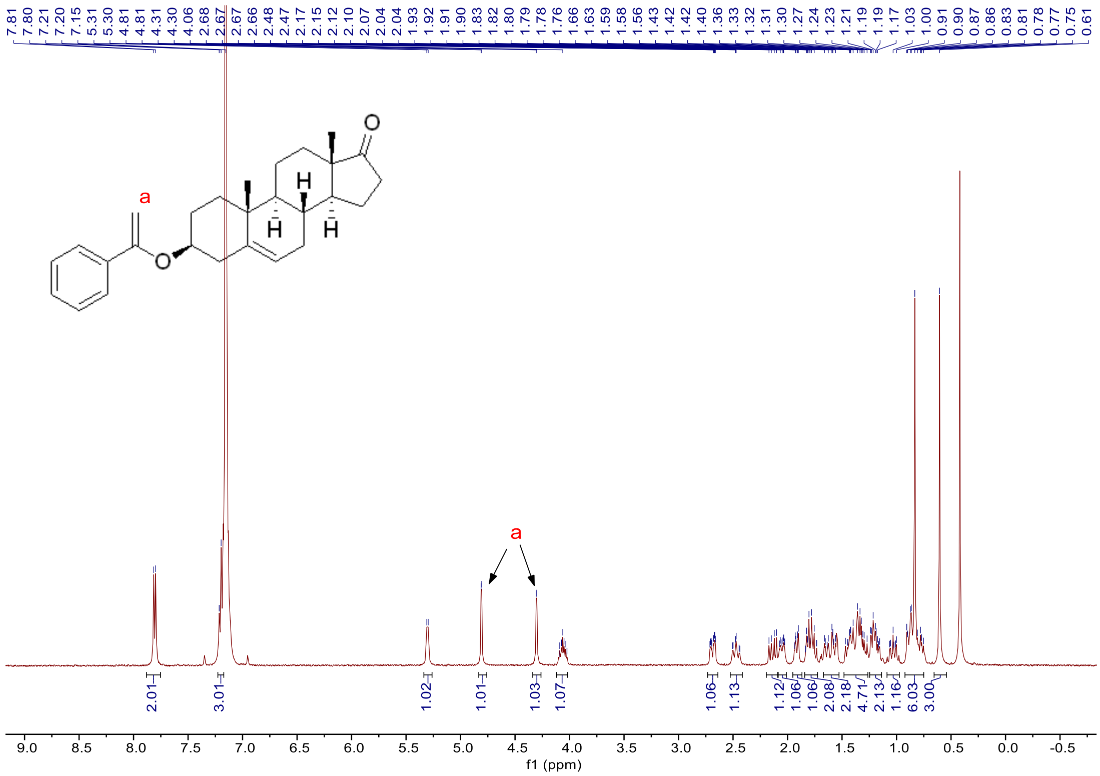

Supplementary Figure 149. ^{1}H NMR spectrum of compound **5h** (400 MHz, C_6D_6)

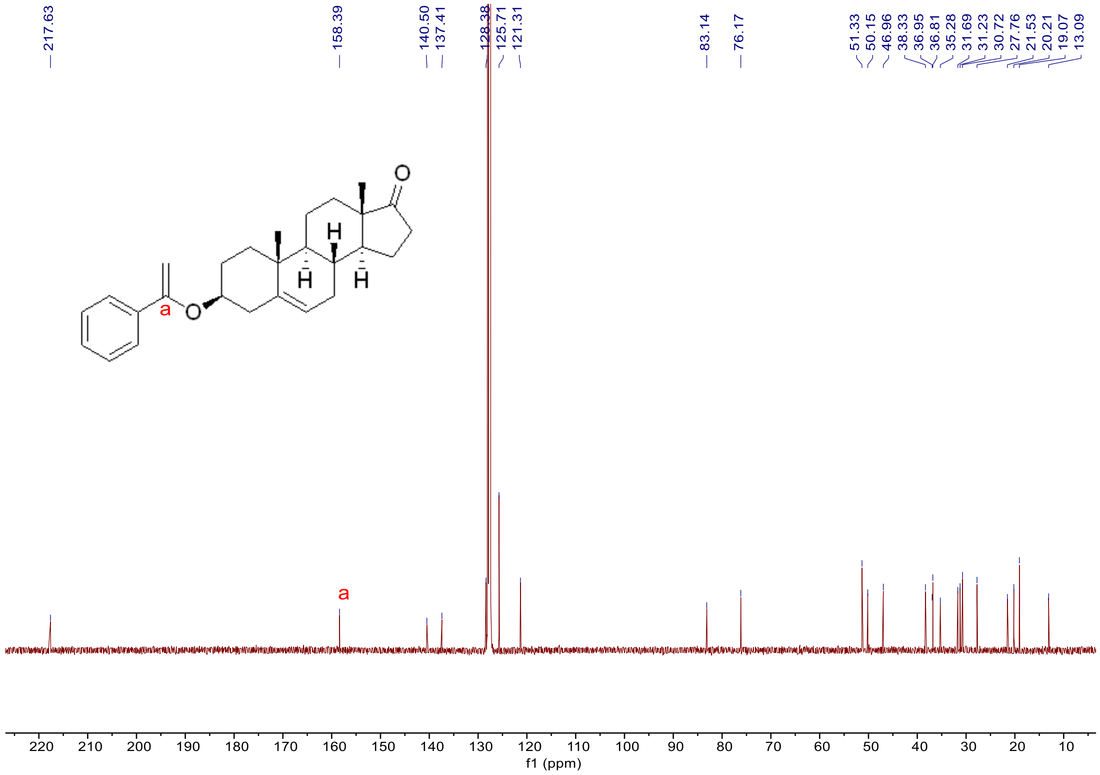

Supplementary Figure 150. ^{13}C NMR spectrum of compound **5h** (100 MHz, C_6D_6)

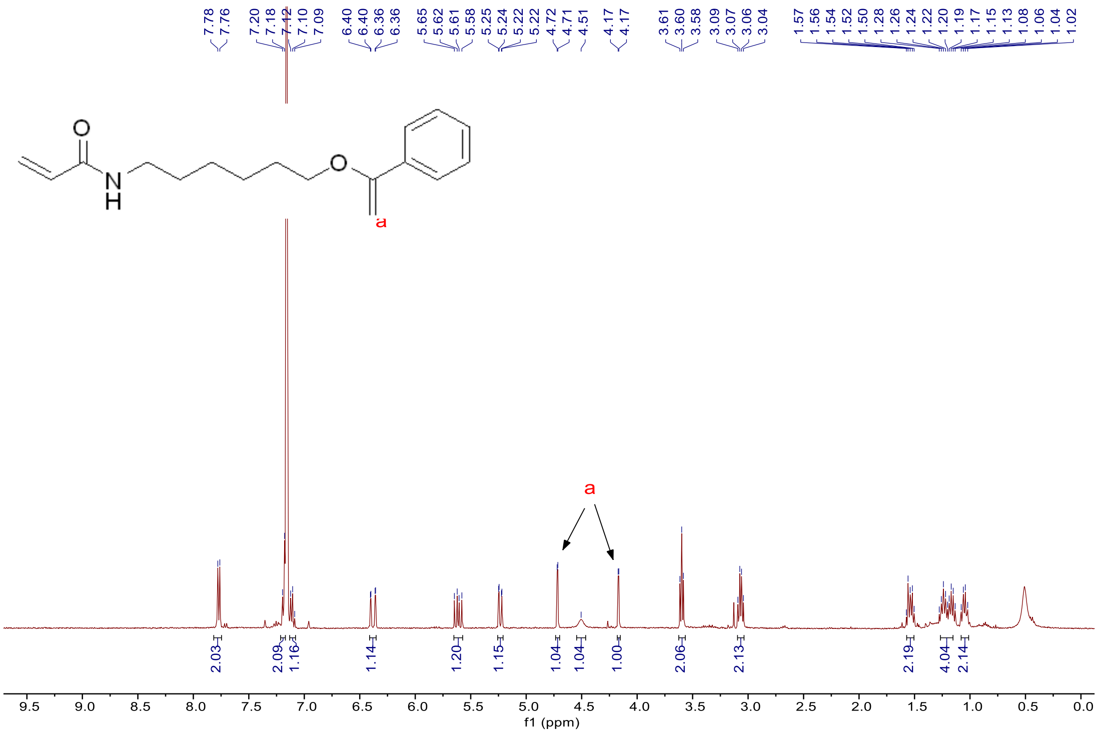

Supplementary Figure 151. ^{1}H NMR spectrum of compound **5i** (400 MHz, C_6D_6)

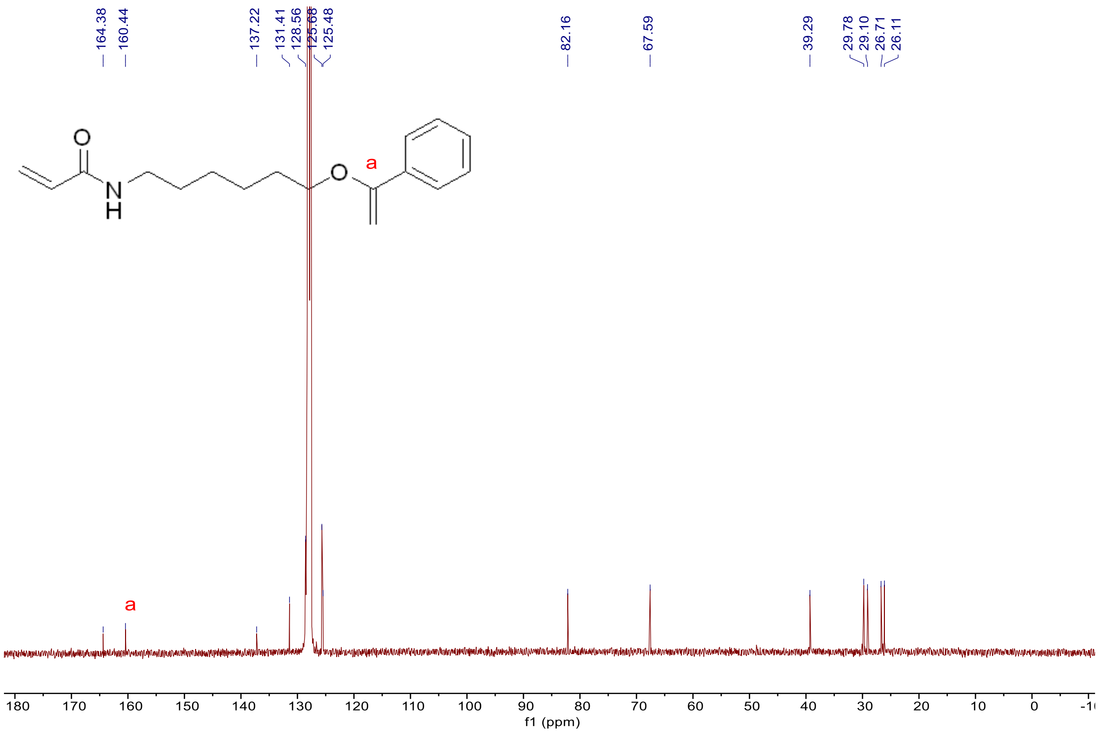

Supplementary Figure 152. ^{13}C NMR spectrum of compound **5i** (100 MHz, C_6D_6)

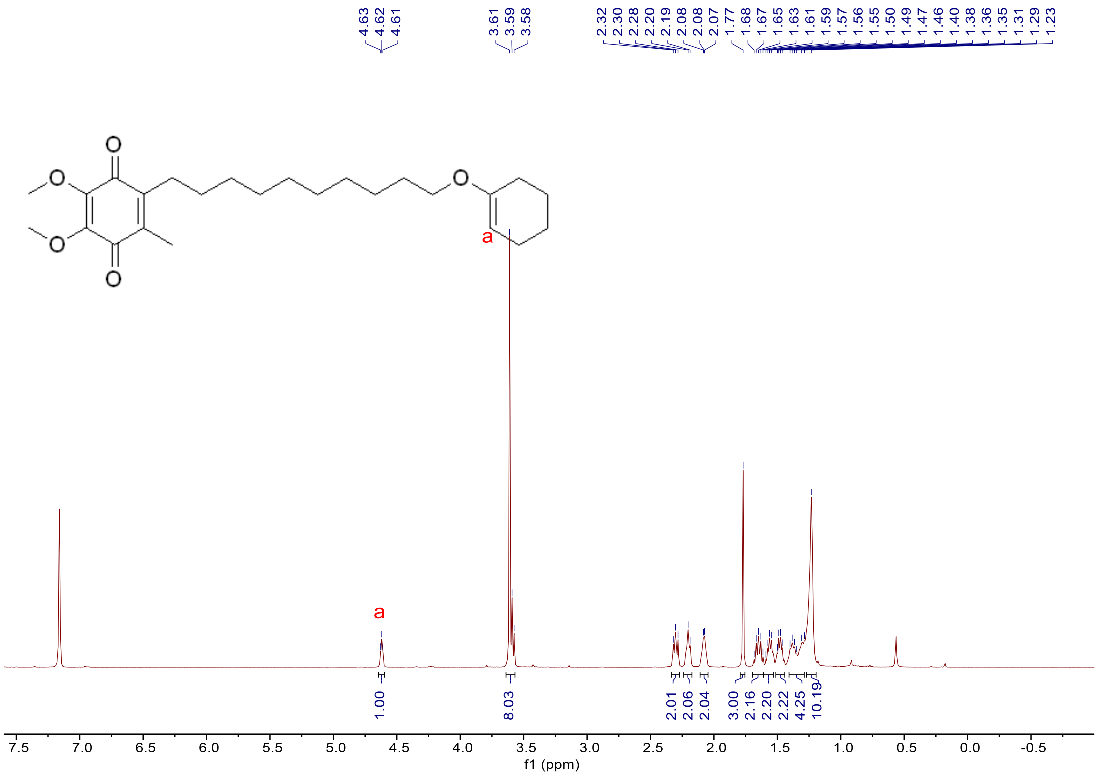

Supplementary Figure 153. ^{1}H NMR spectrum of compound **5j** (400 MHz, C_6D_6)

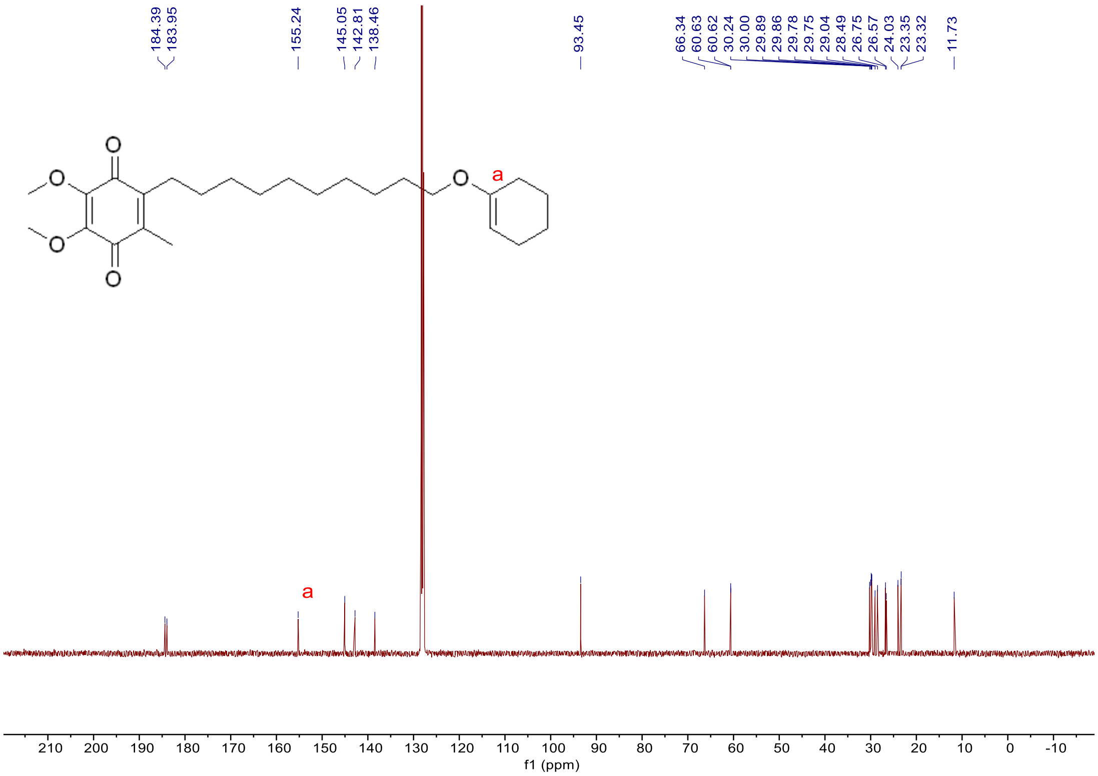

Supplementary Figure 154. ^{13}C NMR spectrum of compound **5j** (100 MHz, C_6D_6)

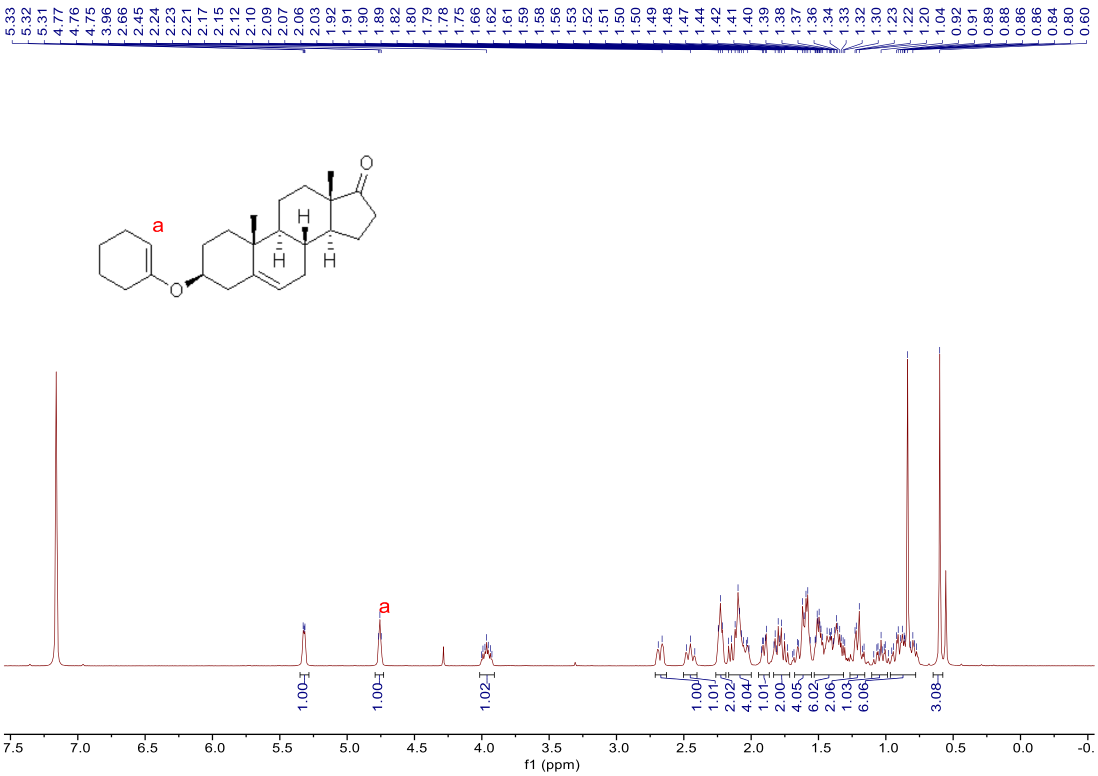

Supplementary Figure 155. ^{1}H NMR spectrum of compound **5k** (400 MHz, C_6D_6)

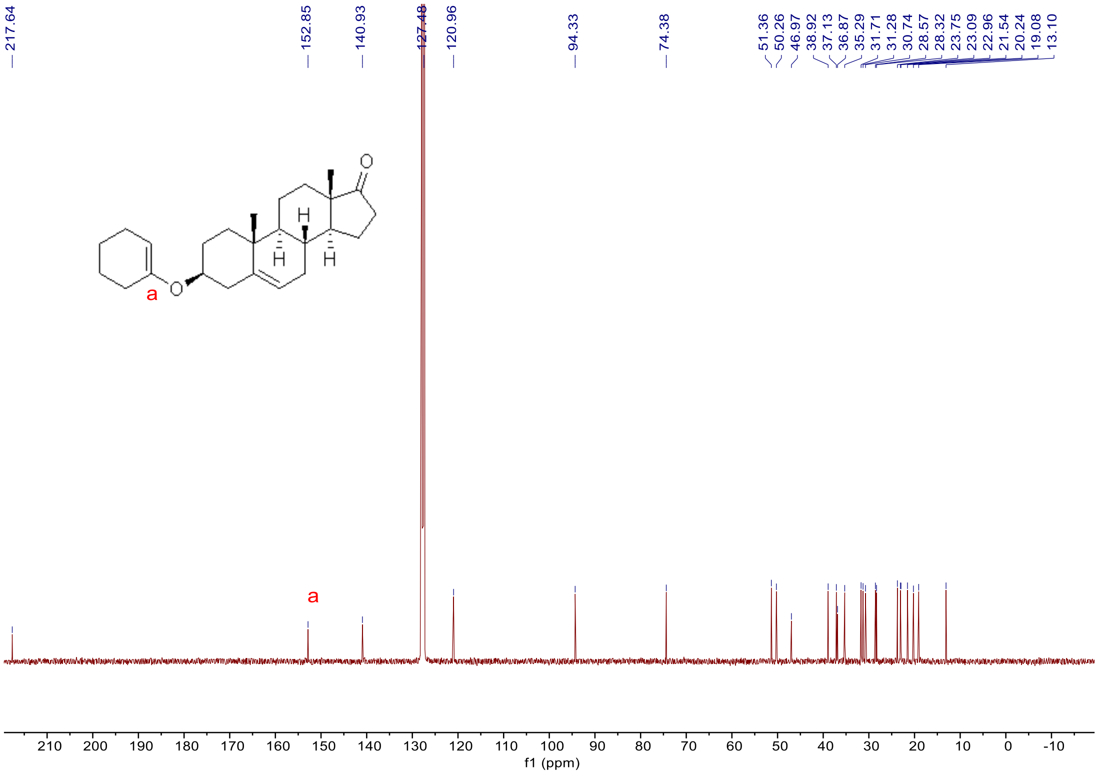

Supplementary Figure 156. ^{13}C NMR spectrum of compound **5k** (100 MHz, C_6D_6)

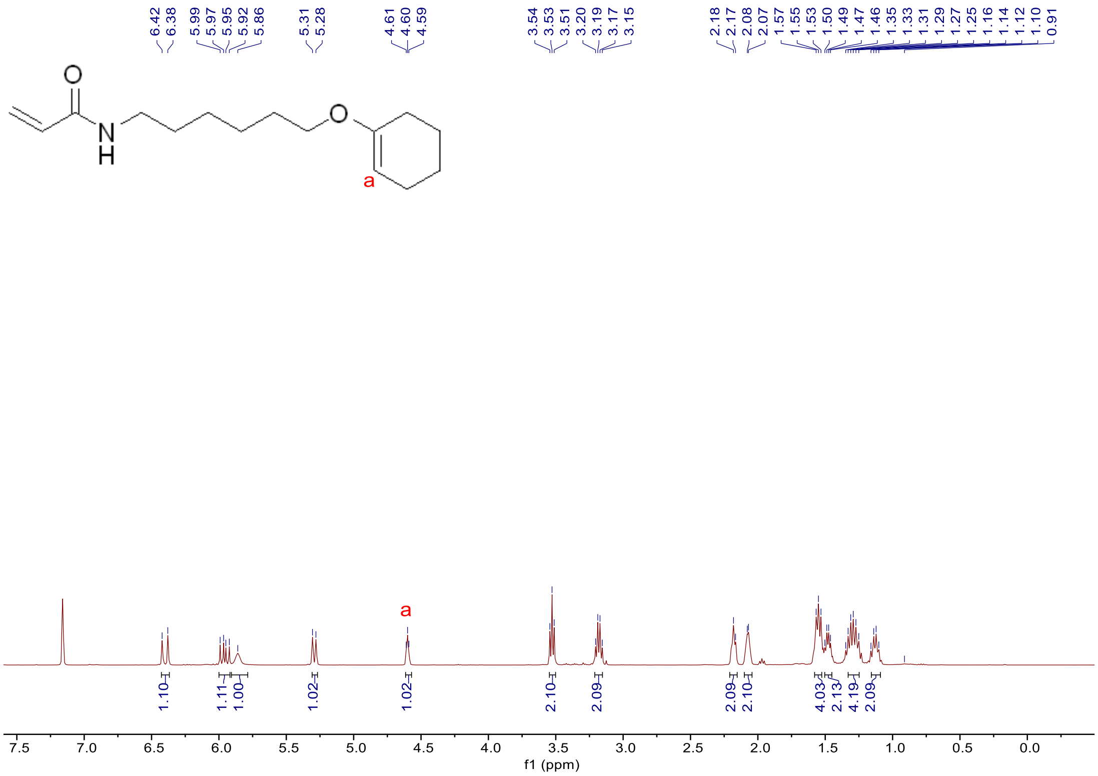

Supplementary Figure 157. ^{1}H NMR spectrum of compound **5l** (400 MHz, C_6D_6)

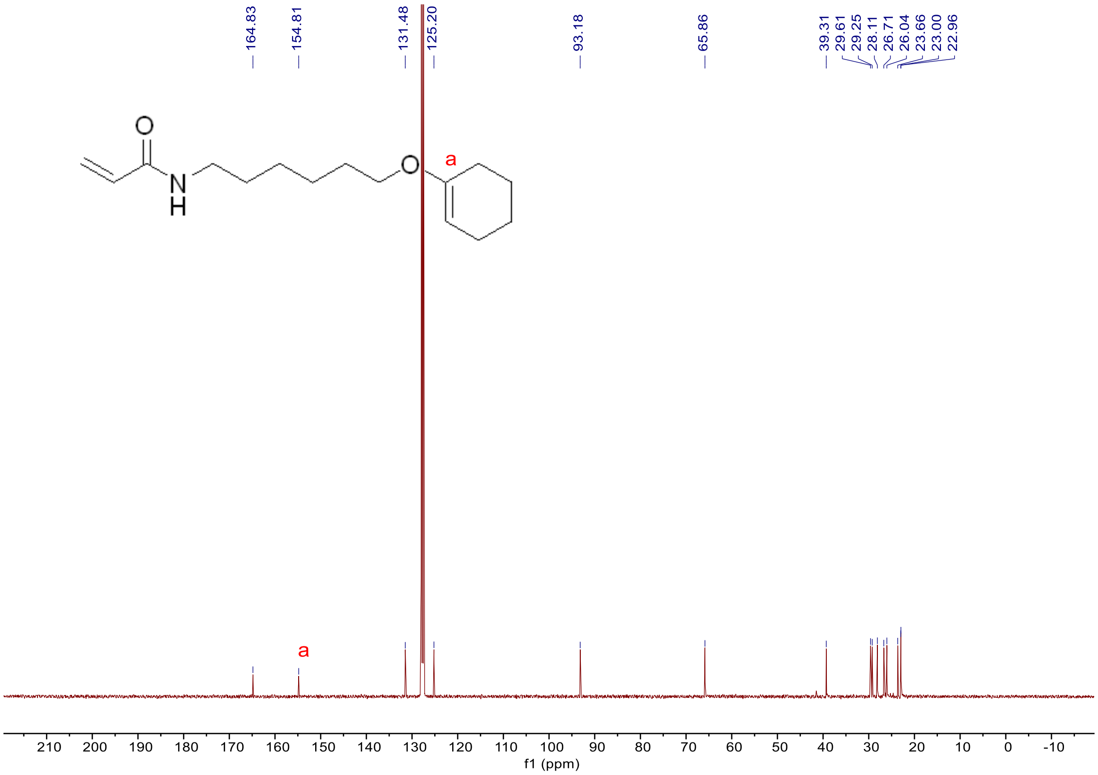

Supplementary Figure 158. ^{13}C NMR spectrum of compound **5l** (100 MHz, C_6D_6)

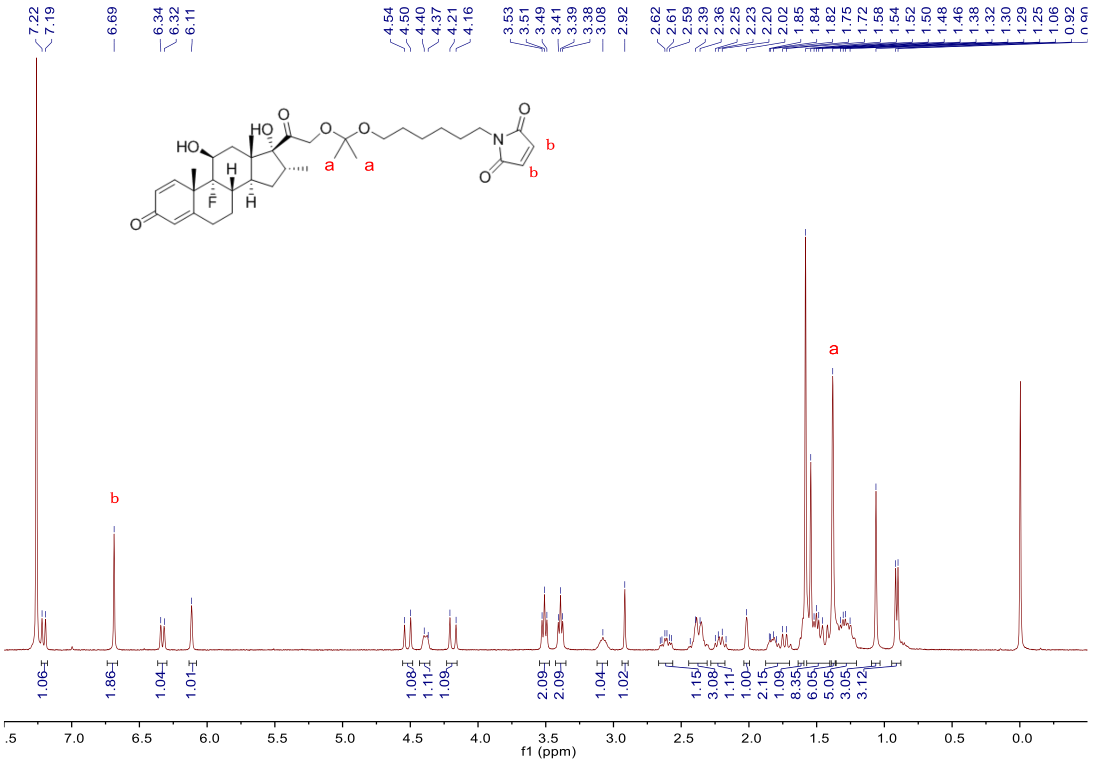

Supplementary Figure 159. ^{1}H NMR spectrum of compound **MAL-K-DEX** (400 MHz, $CDCl_3$)

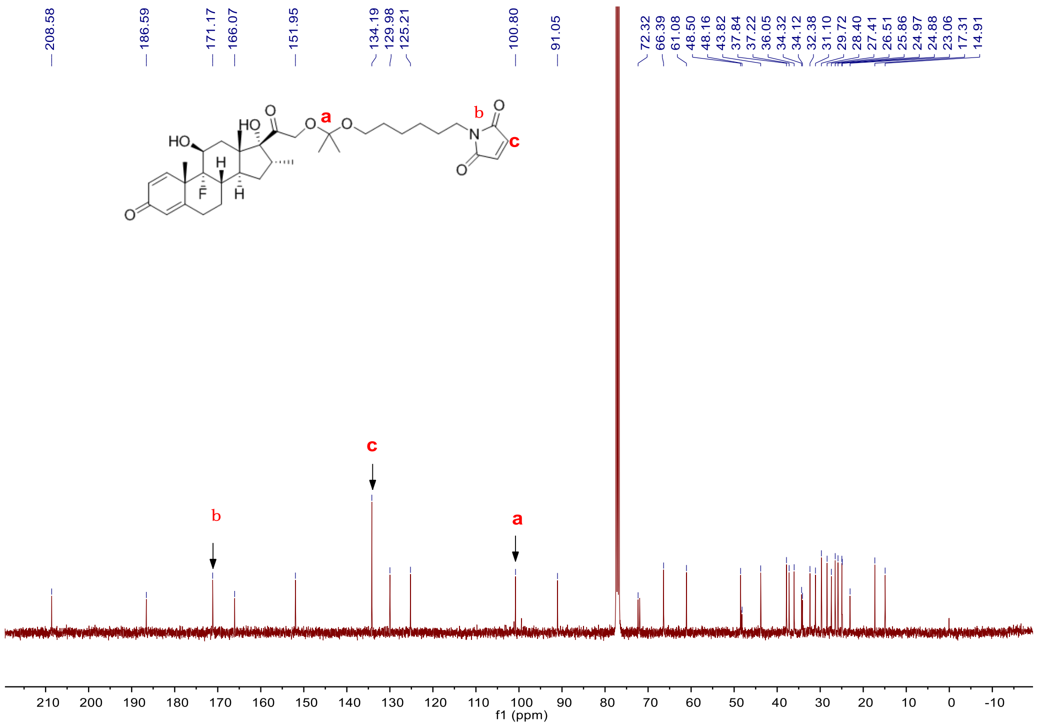

Supplementary Figure 160. ^{13}C NMR spectrum of compound **MAL-K-DEX** (100 MHz, $CDCl_3$)

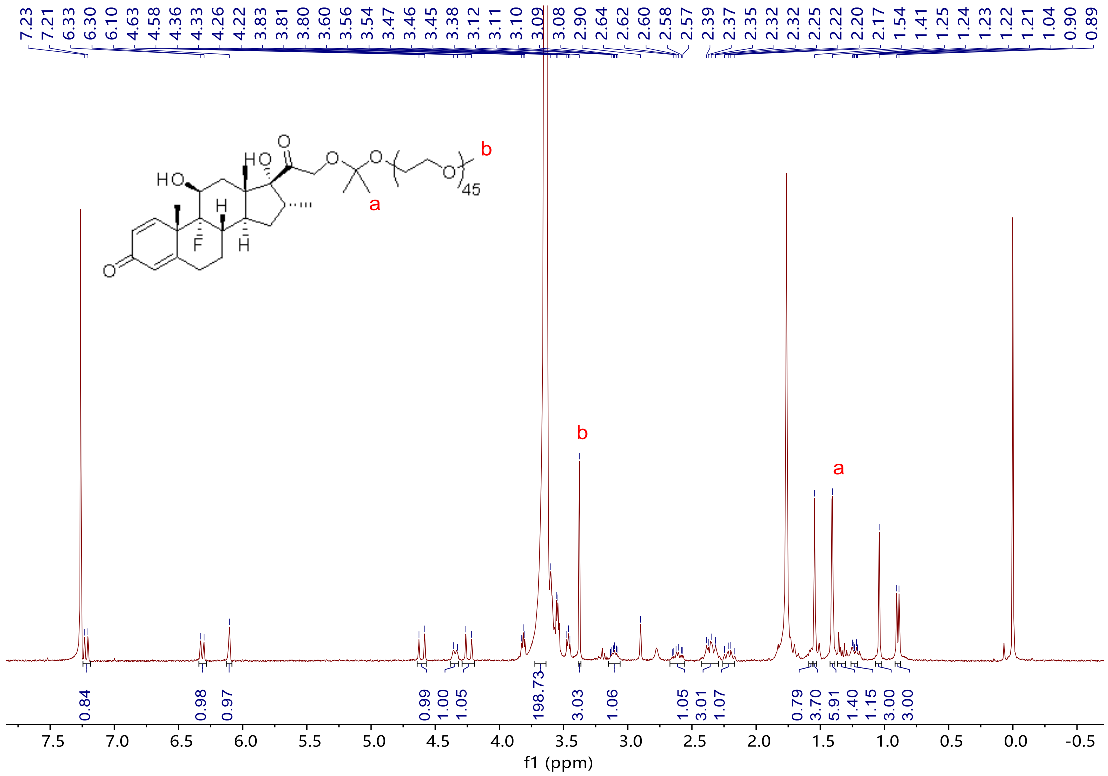

Supplementary Figure 161. ^{1}H NMR spectrum of compound **PEG-K-DEX** (400 MHz, $CDCl_3$)

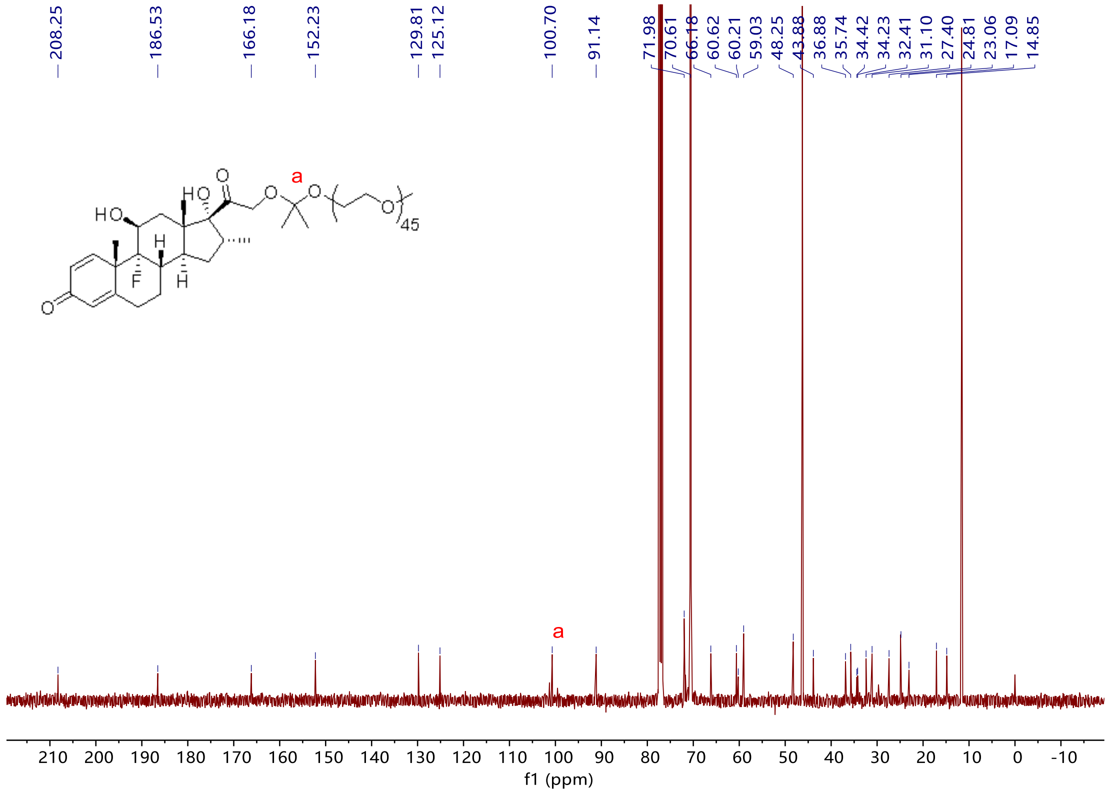

Supplementary Figure 162. ^{13}C NMR spectrum of compound **PEG-K-DEX** (100 MHz, $CDCl_3$)

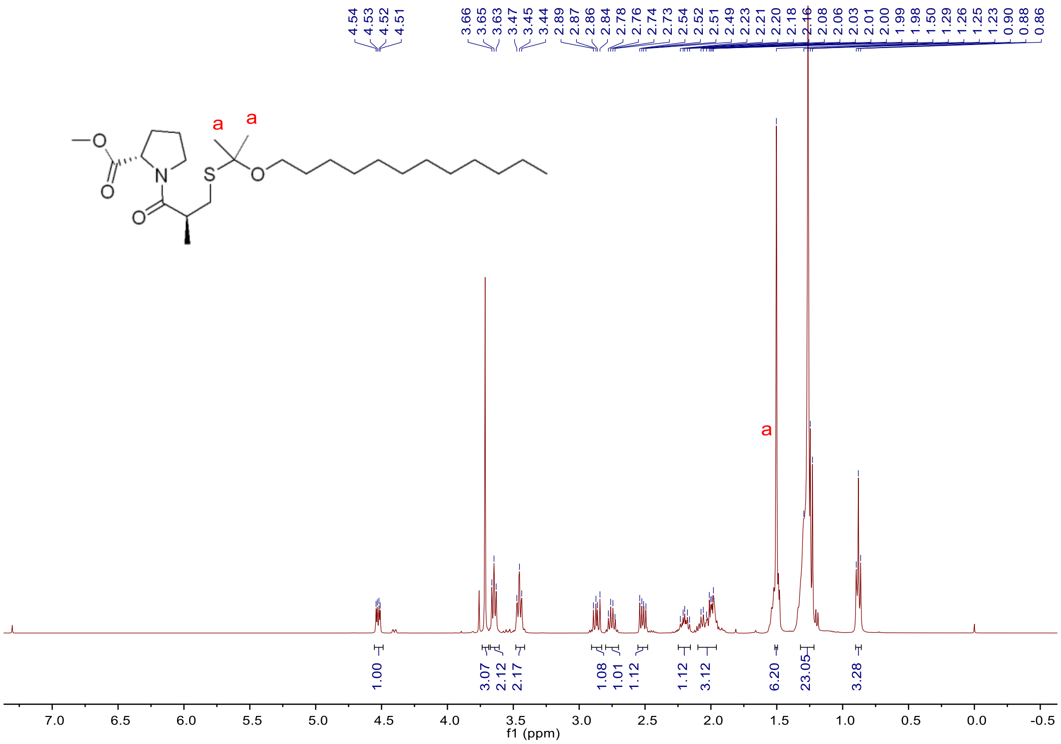

Supplementary Figure 163. ^{1}H NMR spectrum of compound **LA-K-CAPME** (400 MHz, $CDCl_3$)

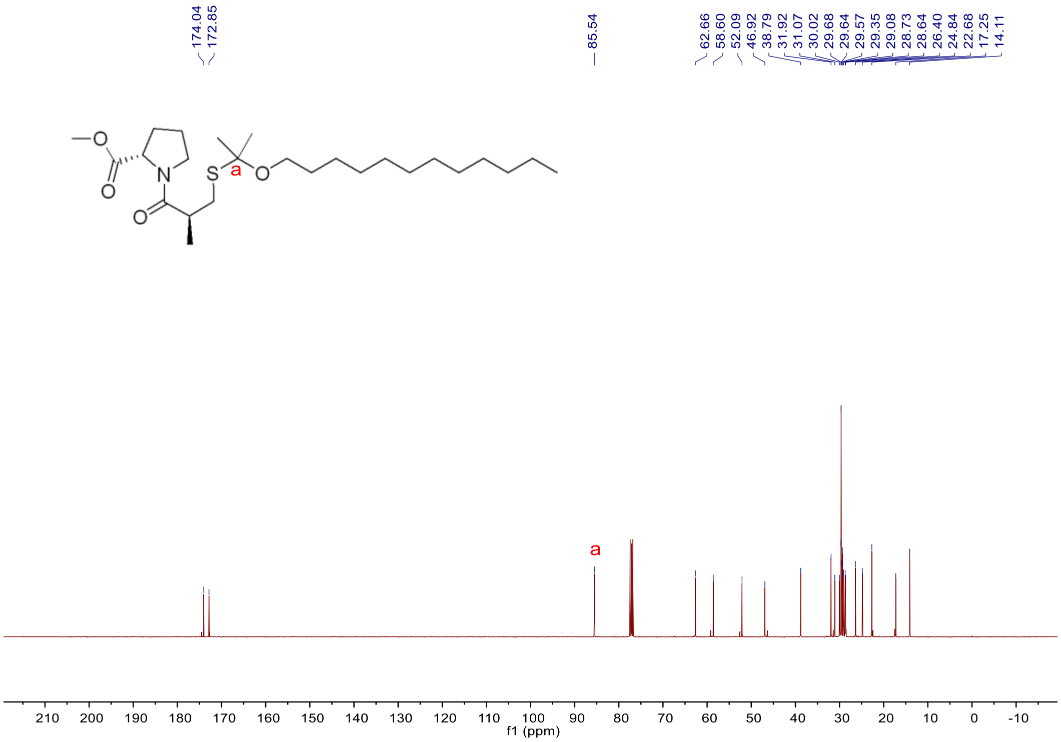

Supplementary Figure 164. ^{13}C NMR spectrum of compound **LA-K-CAPME** (100 MHz, $CDCl_3$)

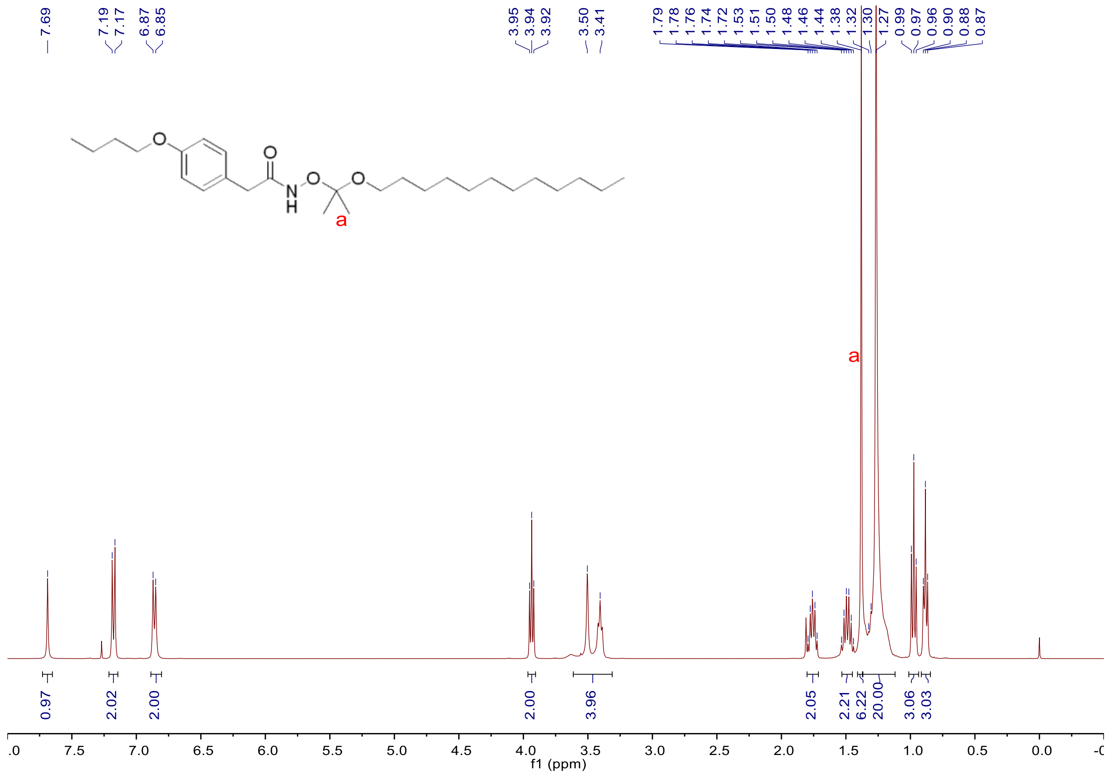

Supplementary Figure 165. ^{1}H NMR spectrum of compound **LA-K-BUF** (400 MHz, $CDCl_3$)

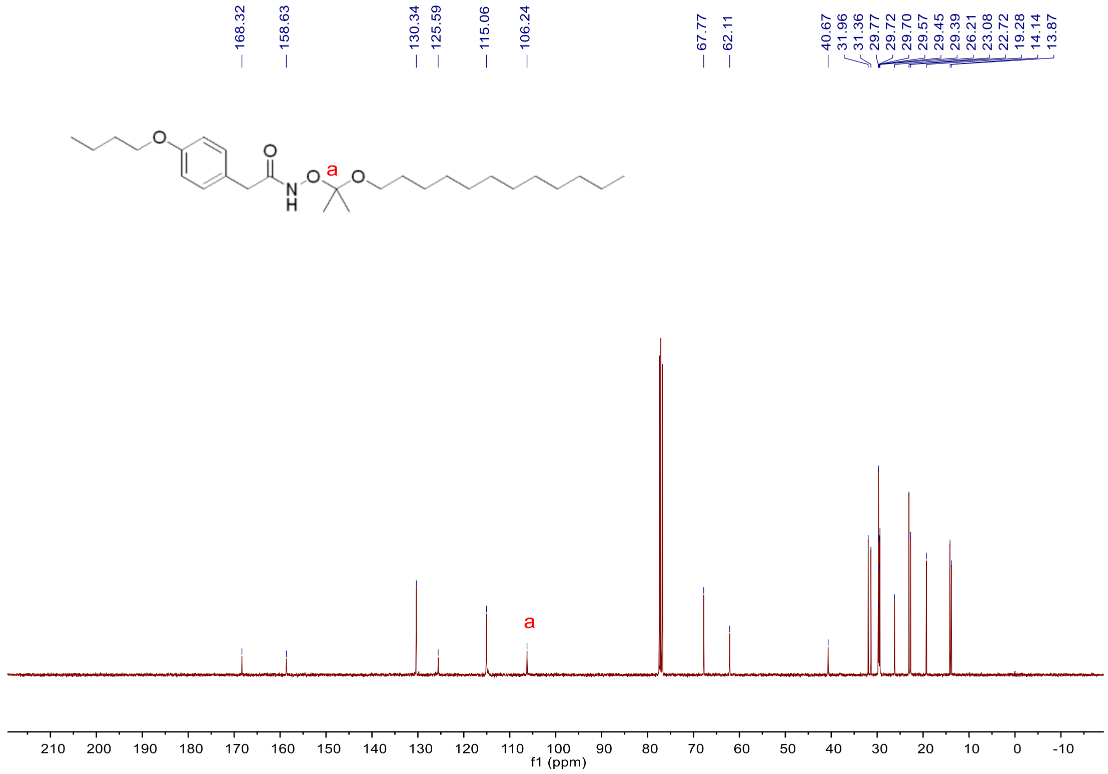

Supplementary Figure 166. ^{13}C NMR spectrum of compound **LA-K-BUF** (100 MHz, $CDCl_3$)

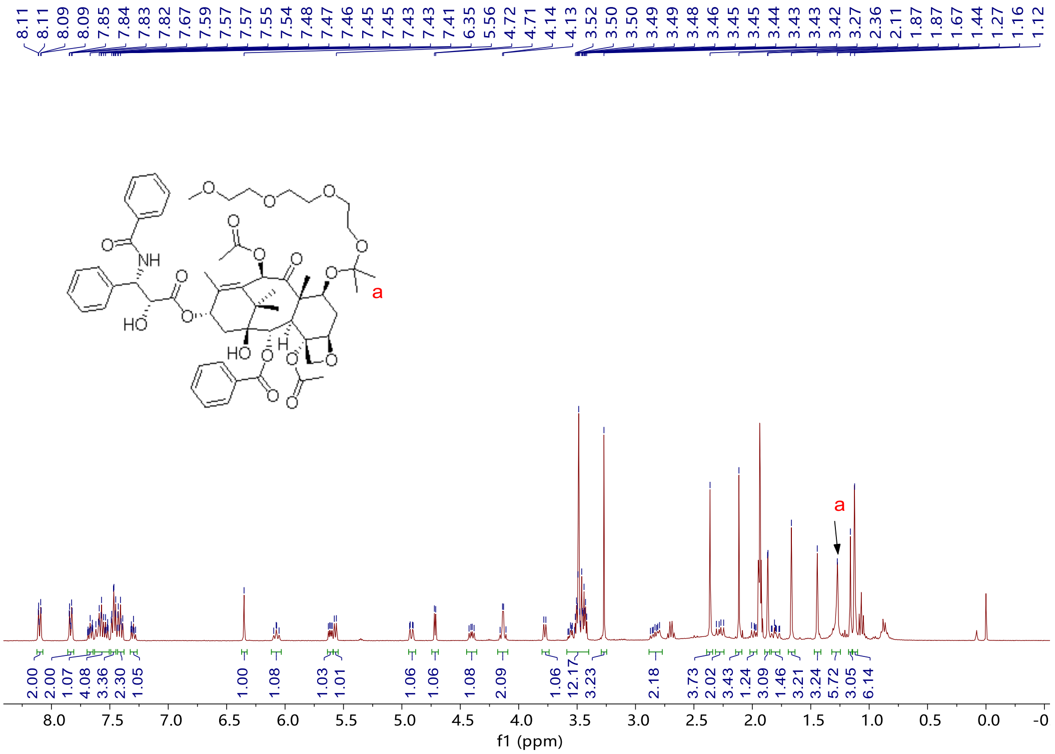

Supplementary Figure 167. ^{1}H NMR spectrum of compound **PTX-7-K-EG3** (400 MHz, CD_3CN)

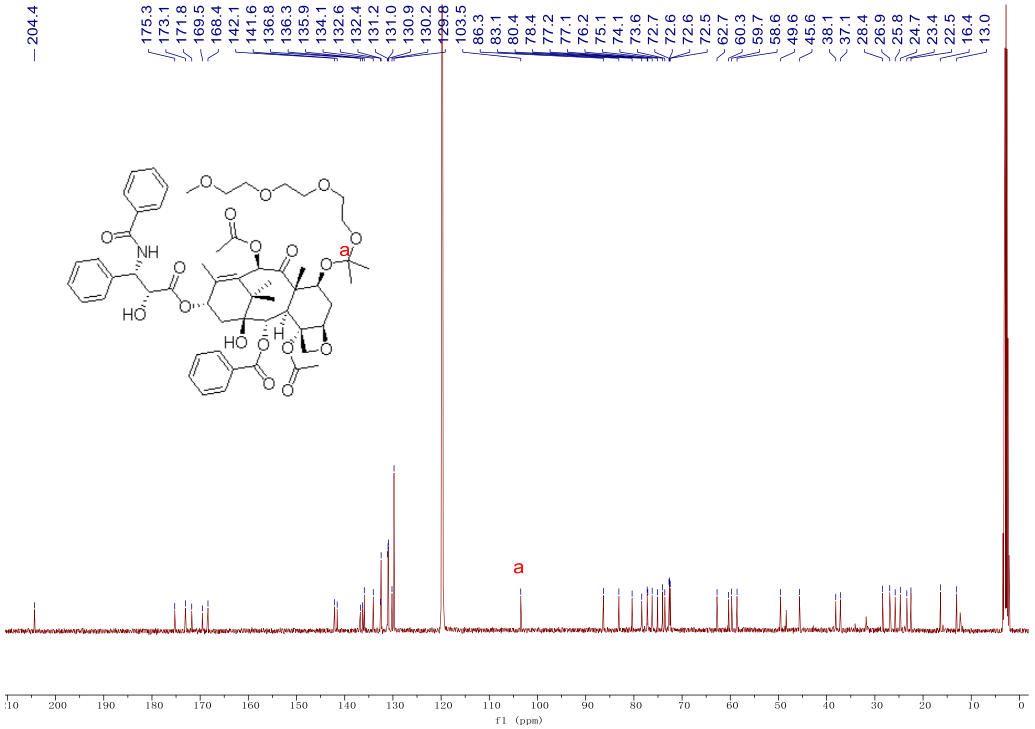

Supplementary Figure 168. ^{13}C NMR spectrum of compound **PTX-7-K-EG3** (100 MHz, CD_3CN)

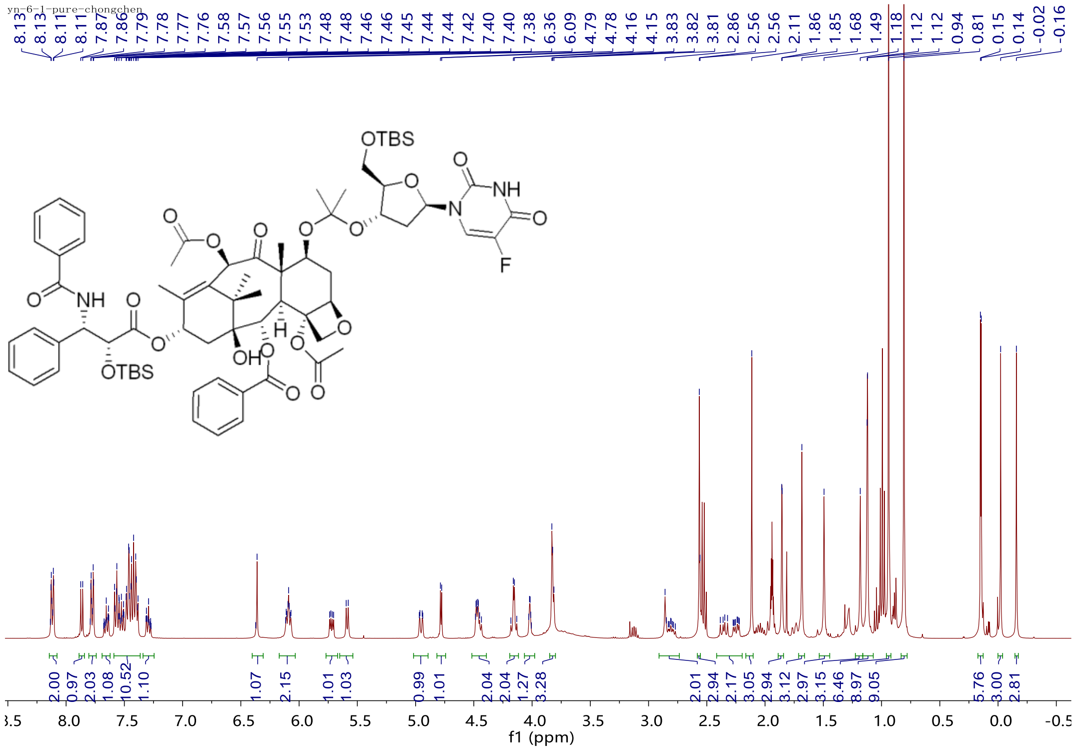

Supplementary Figure 169. ^{1}H NMR spectrum of compound **PTX-2′-TBS-7-K-(FUDR-5′-TBS)** (400 MHz, CD_3CN)

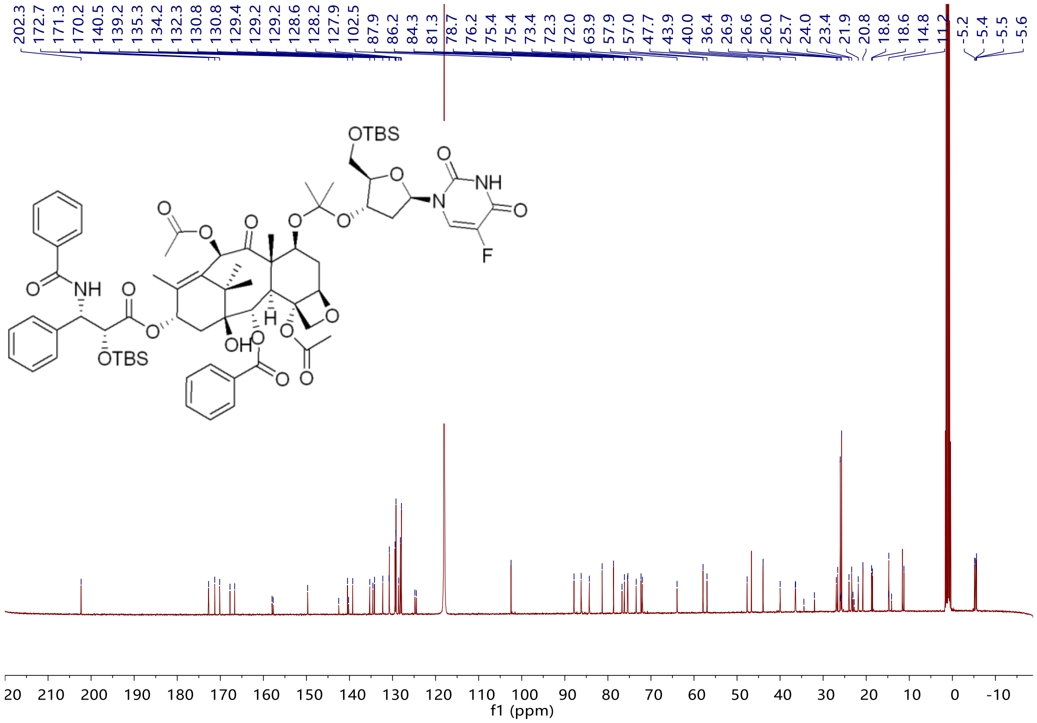

Supplementary Figure 170. ^{13}C NMR spectrum of compound **PTX-2′-TBS-7-K-(FUDR-5′-TBS)** (100 MHz, CD_3CN)

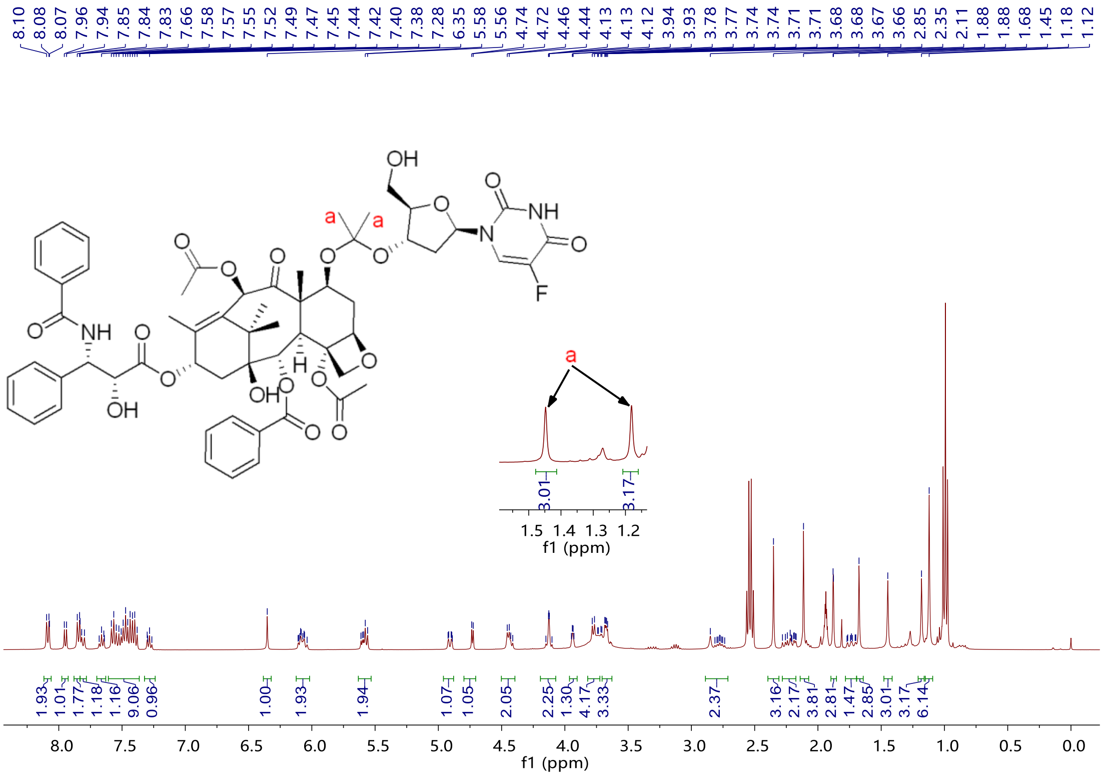

Supplementary Figure 171. ^{1}H NMR spectrum of compound **PK3F** (400 MHz, CD_3CN)

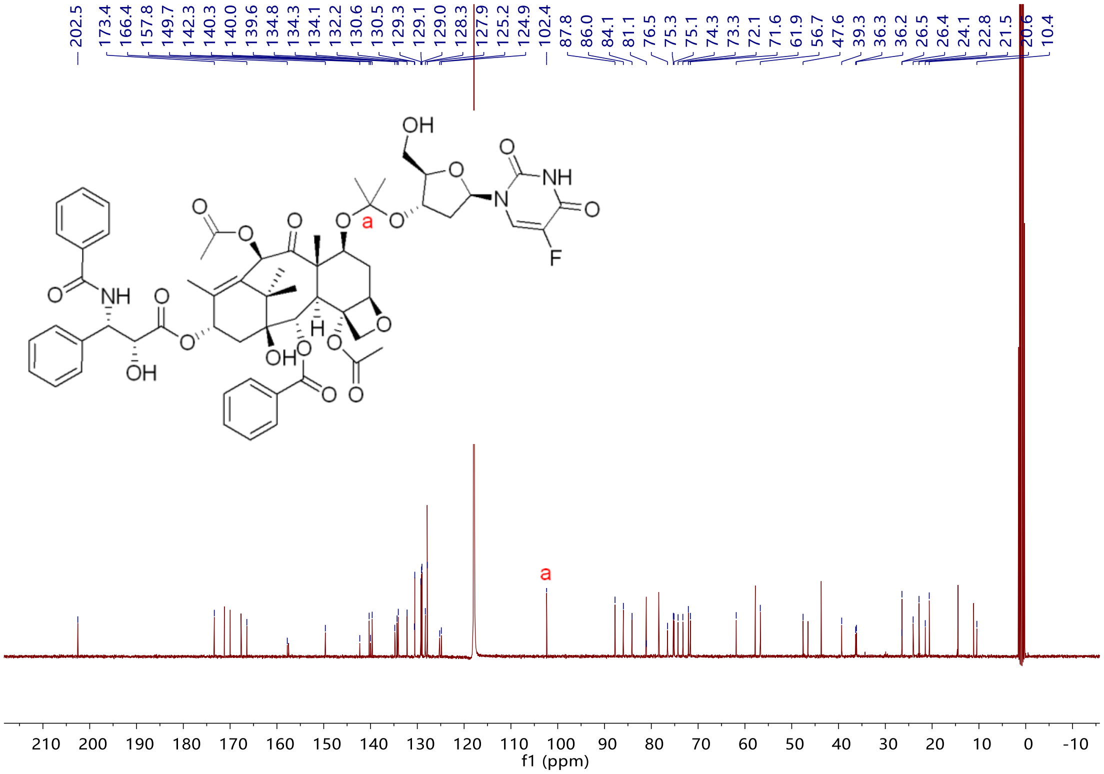

Supplementary Figure 172. ^{13}C NMR spectrum of compound **PK3F** (100 MHz, CD_3CN)

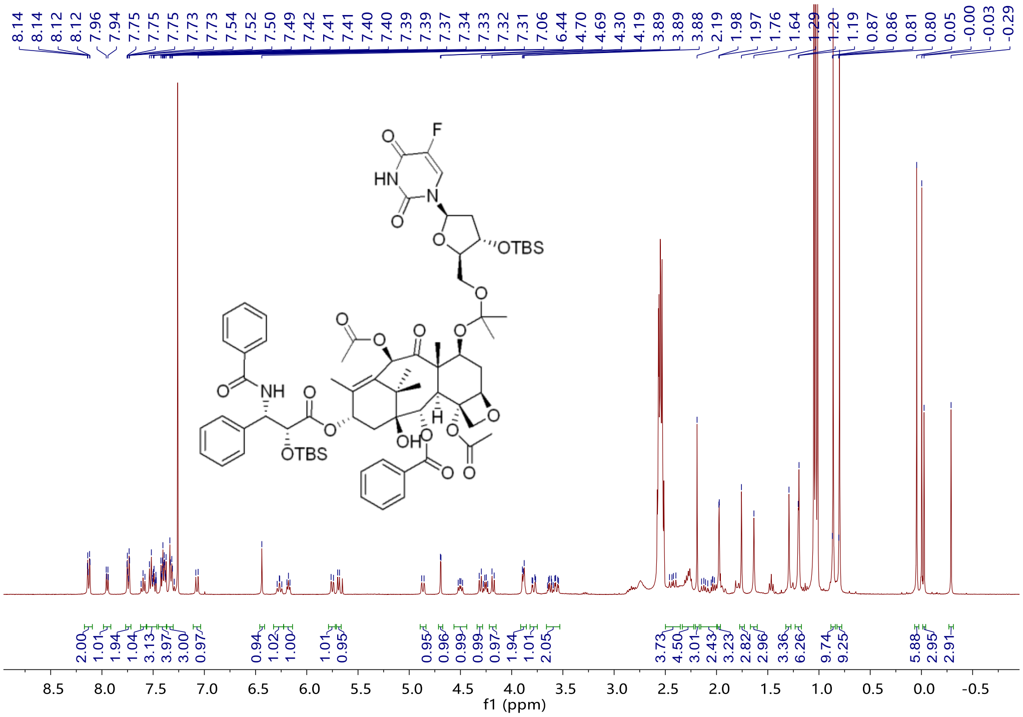

Supplementary Figure 173. ^{1}H NMR spectrum of compound **PTX-2′-TBS-7-K-(FUDR-3′-TBS)** (400 MHz, $CDCl_3$)

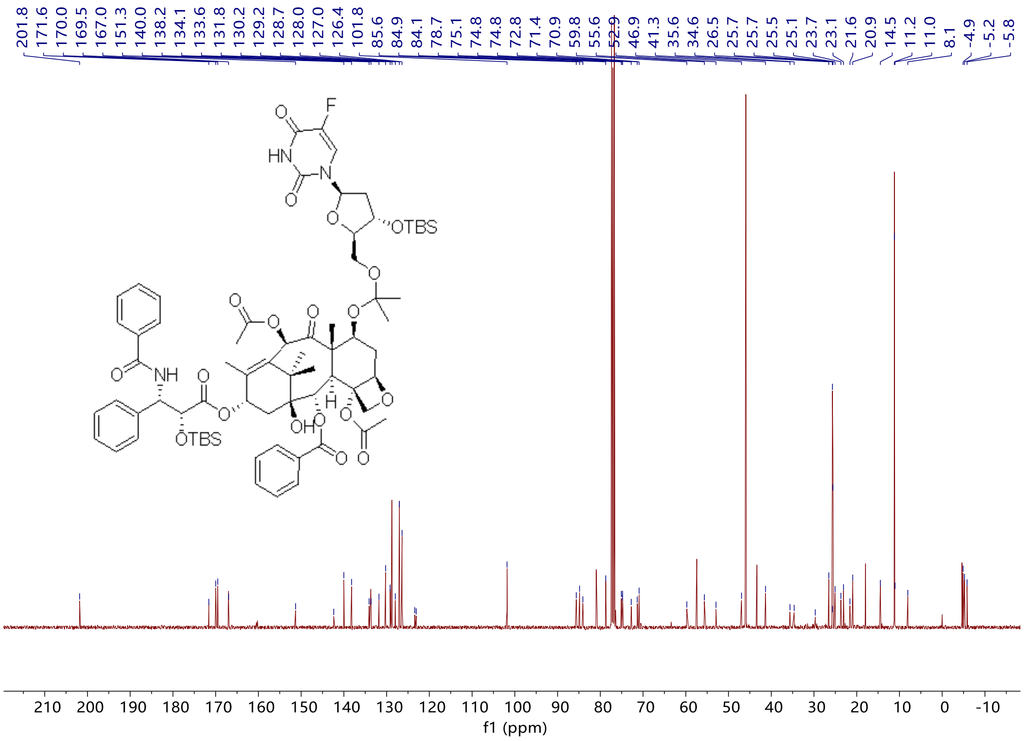

Supplementary Figure 174. ^{13}C NMR spectrum of compound **PTX-2′-TBS-7-K-(FUDR-3′-TBS)** (100 MHz, $CDCl_3$)

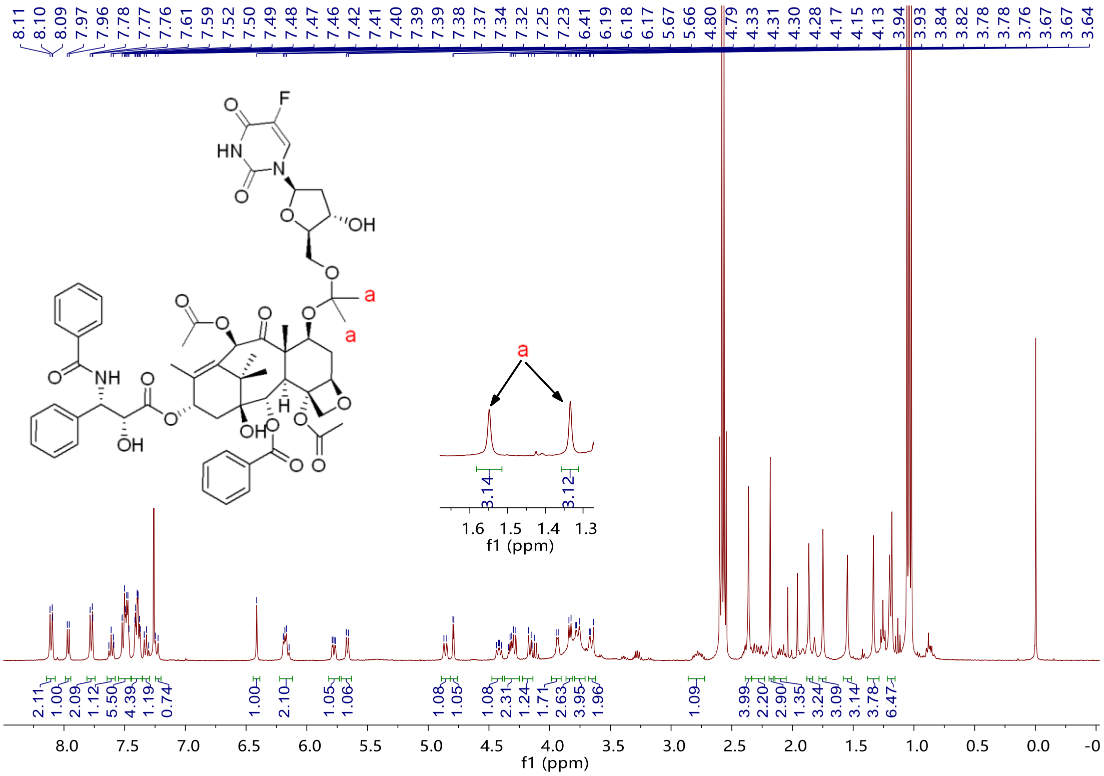

Supplementary Figure 175. ^{1}H NMR spectrum of compound **PK5F** (400 MHz, $CDCl_3$)

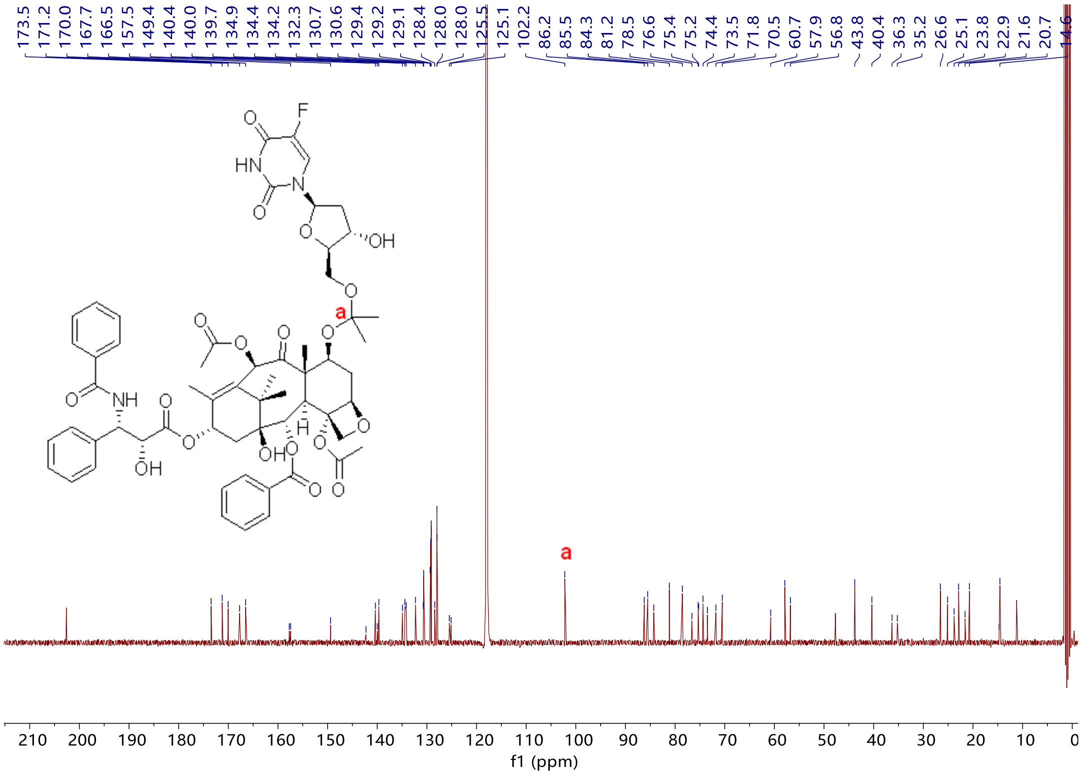

Supplementary Figure 176. ^{13}C NMR spectrum of compound **PK5F** (100 MHz, CD_3CN)

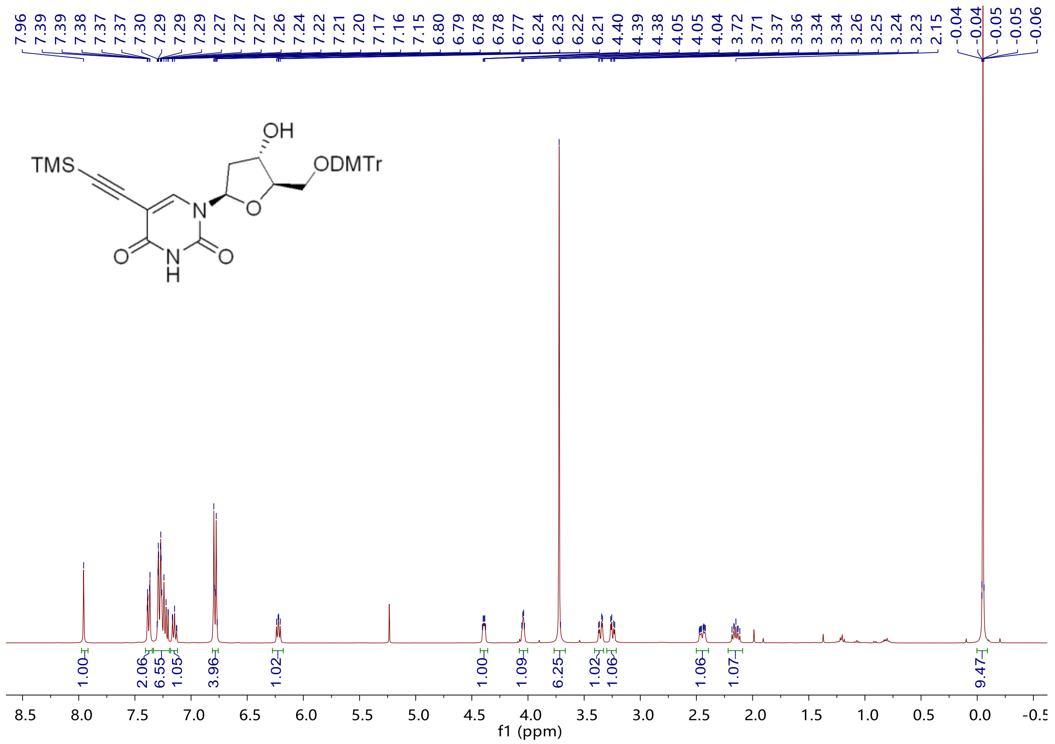

Supplementary Figure 177. ^{1}H NMR spectrum of compound **EdU-TMS-5′-DMTr** (400 MHz, $CDCl_3$)

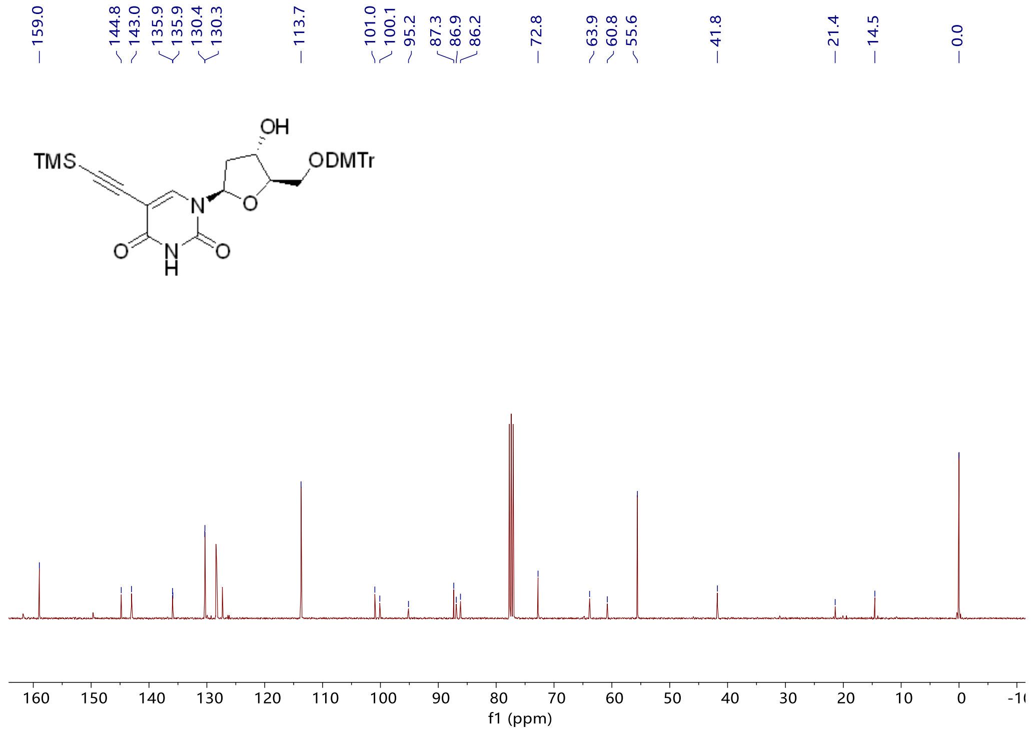

Supplementary Figure 178. ^{13}C NMR spectrum of compound **EdU-TMS-5′-DMTr** (100 MHz, $CDCl_3$)

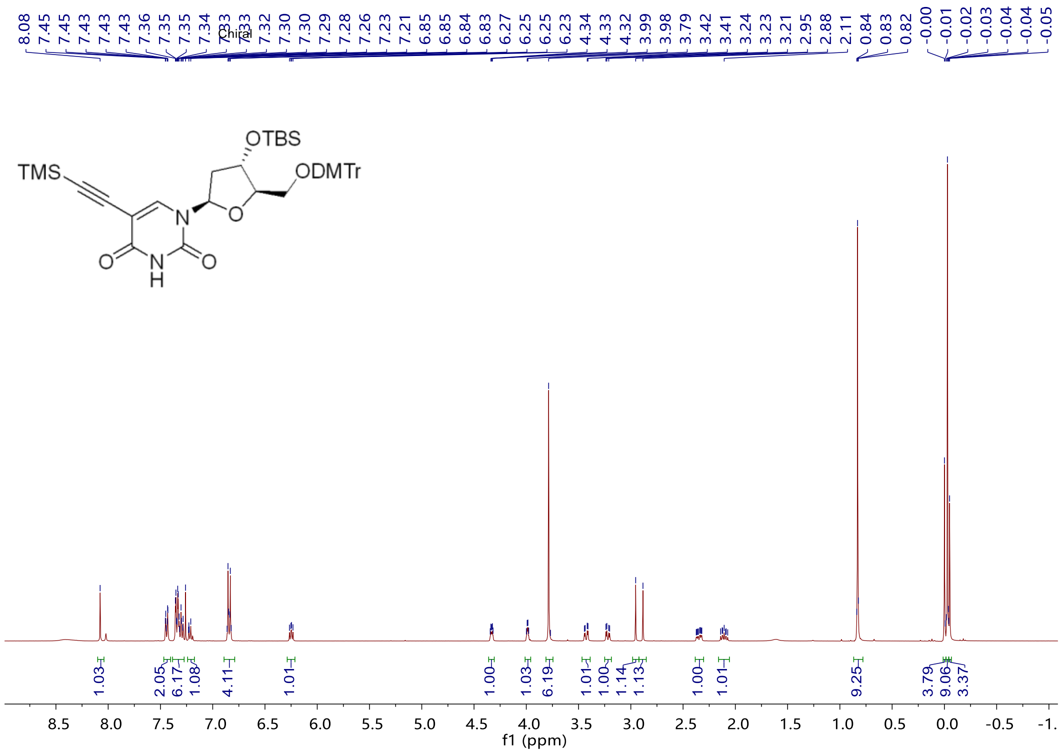

Supplementary Figure 179. ^{1}H NMR spectrum of compound **EdU-TMS-5′-DMTr-3′-TBS** (400 MHz, $CDCl_3$)

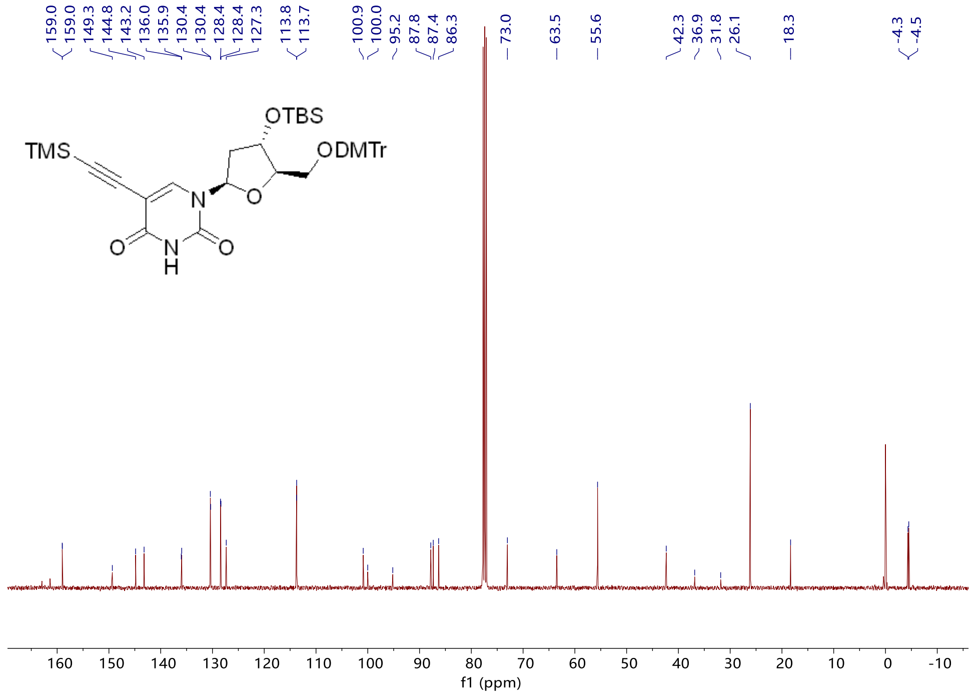

Supplementary Figure 180. ^{13}C NMR spectrum of compound **EdU-TMS-5′-DMTr-3′-TBS** (100 MHz, $CDCl_3$)

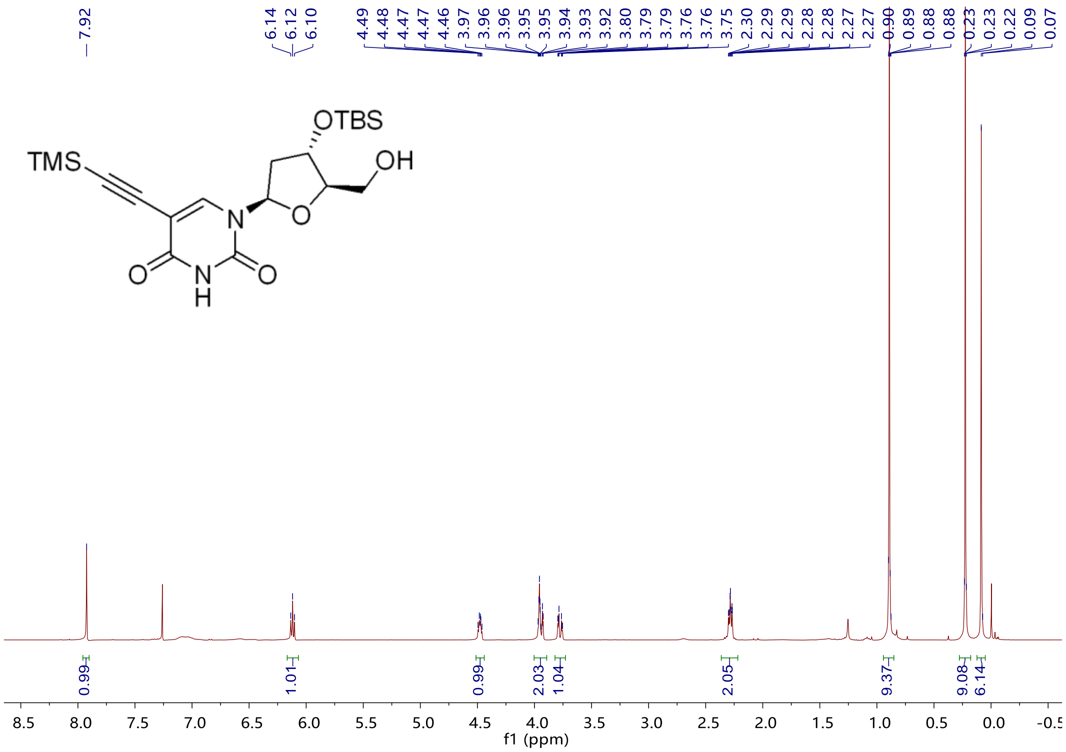

Supplementary Figure 181. ^{1}H NMR spectrum of compound **EdU-TMS-3′-TBS** (400 MHz, $CDCl_3$)

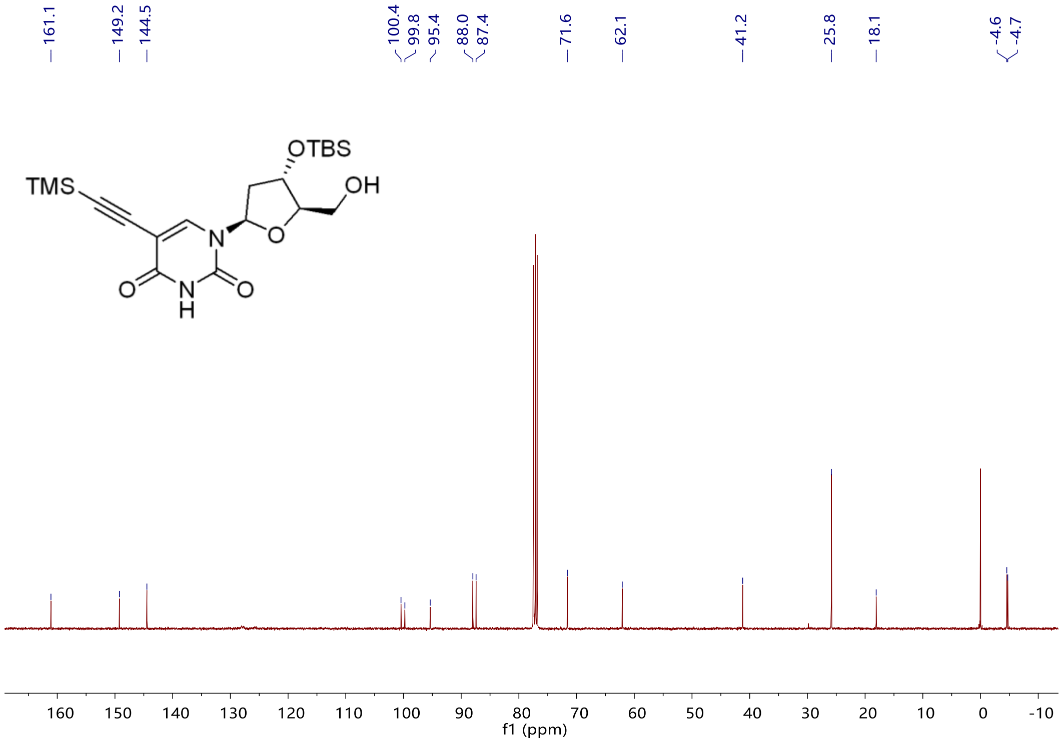

Supplementary Figure 182. ^{13}C NMR spectrum of compound **EdU-TMS-3′-TBS** (100 MHz, $CDCl_3$)

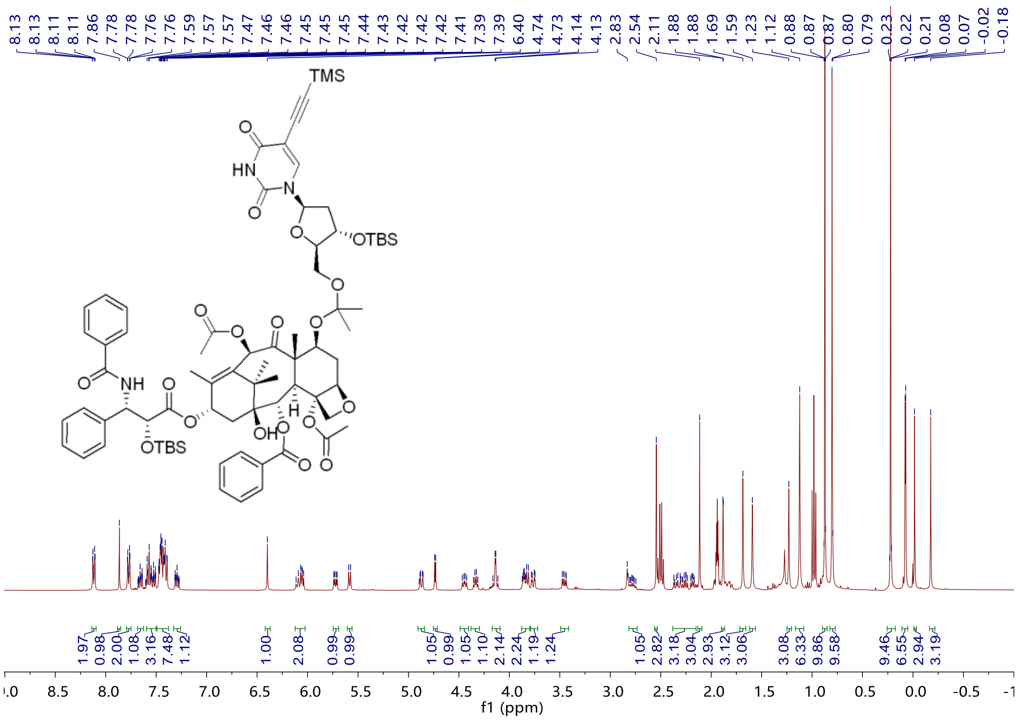

Supplementary Figure 183. ^{1}H NMR spectrum of compound **PTX-2′-TBS-7-K-(EdU-TMS-3′-TBS)** (400 MHz, CD_3CN)

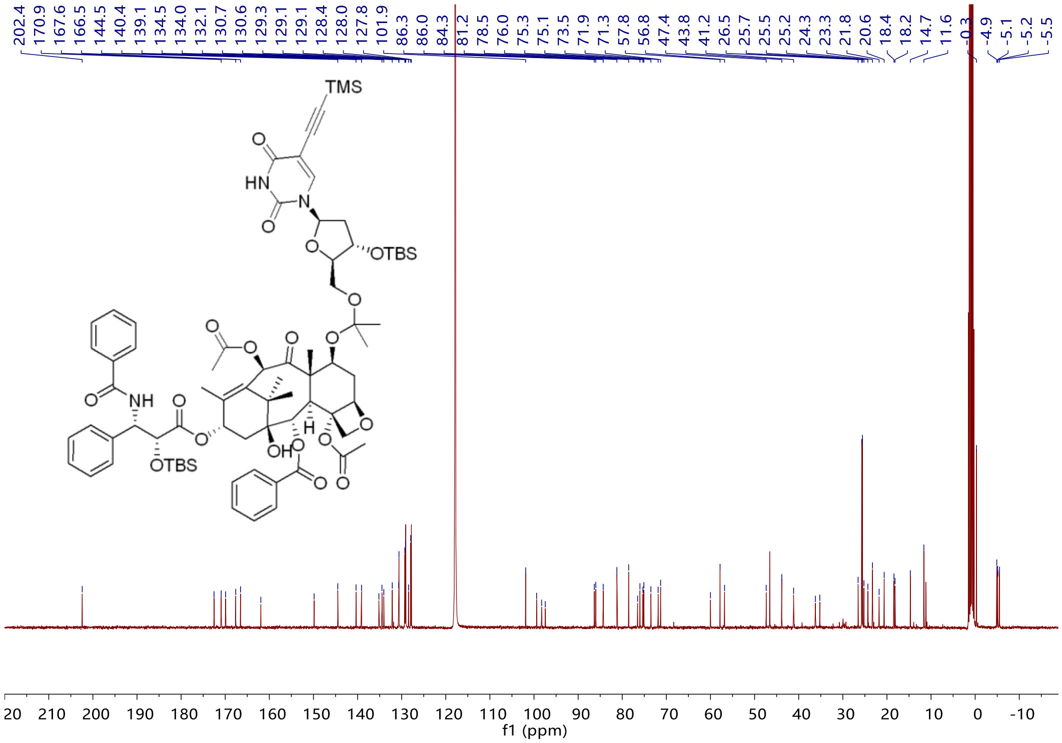

Supplementary Figure 184. ^{13}C NMR spectrum of compound **PTX-2′-TBS-7-K-(EdU-TMS-3′-TBS)** (100 MHz, CD_3CN)

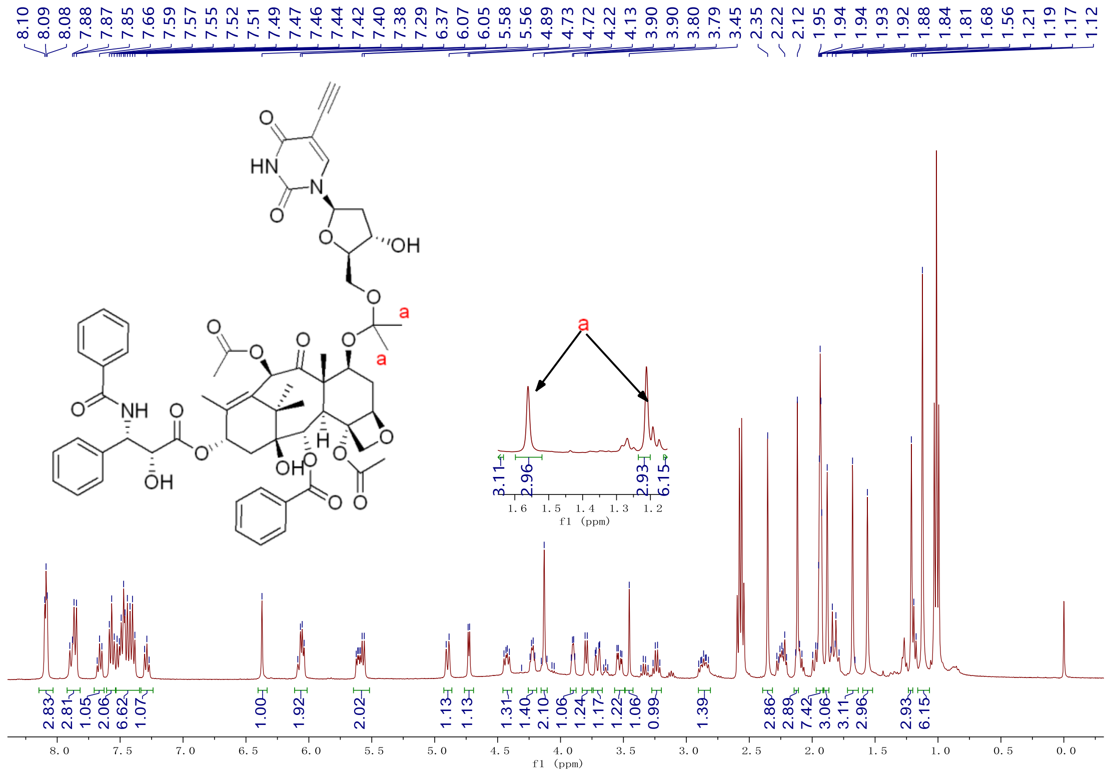

Supplementary Figure 185. ^{1}H NMR spectrum of compound **PK5E** (400 MHz, CD_3CN)

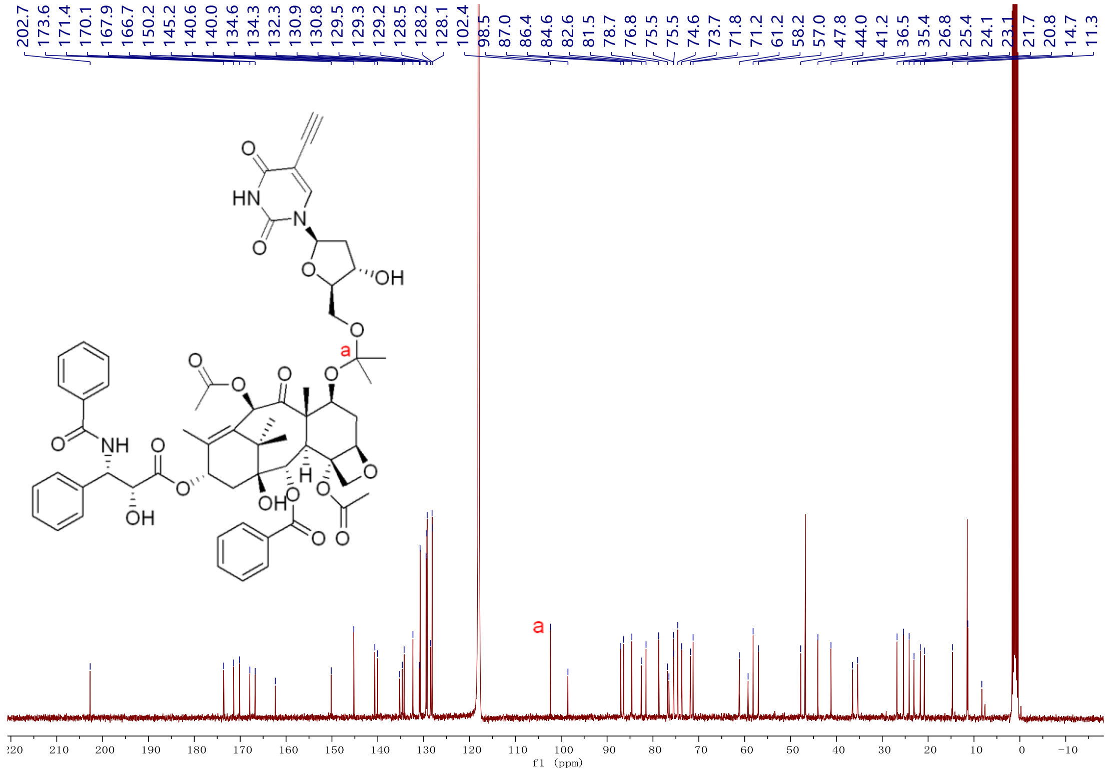

Supplementary Figure 186. ^{13}C NMR spectrum of compound **PK5E** (100 MHz, CD_3CN)

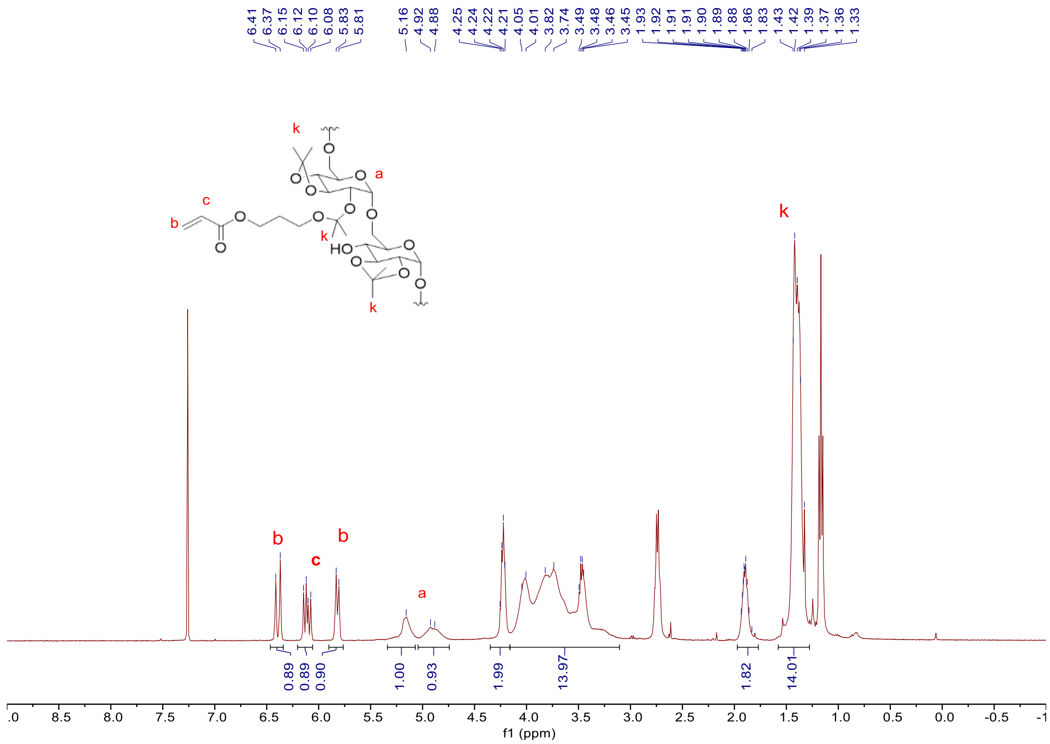

Supplementary Figure 187. ^{1}H NMR spectrum of acrylate modified dextran with **3t** (400 MHz, $CDCl_3$)

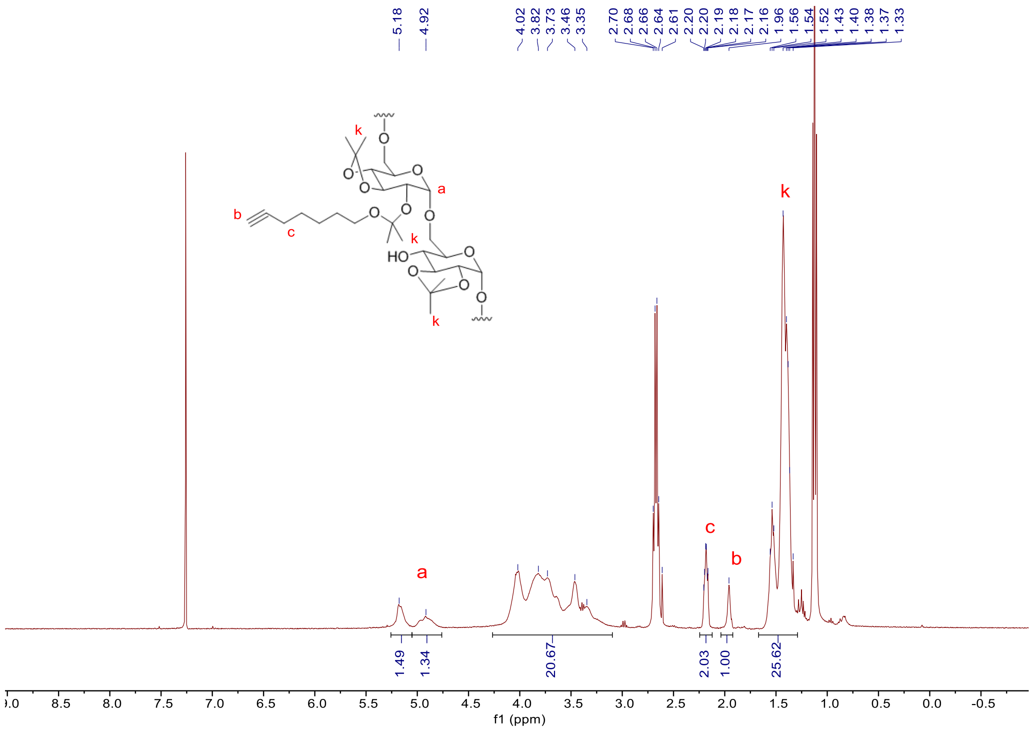

Supplementary Figure 188. ^{1}H NMR spectrum of alkyne modified dextran with **3r** (400 MHz, $CDCl_3$)

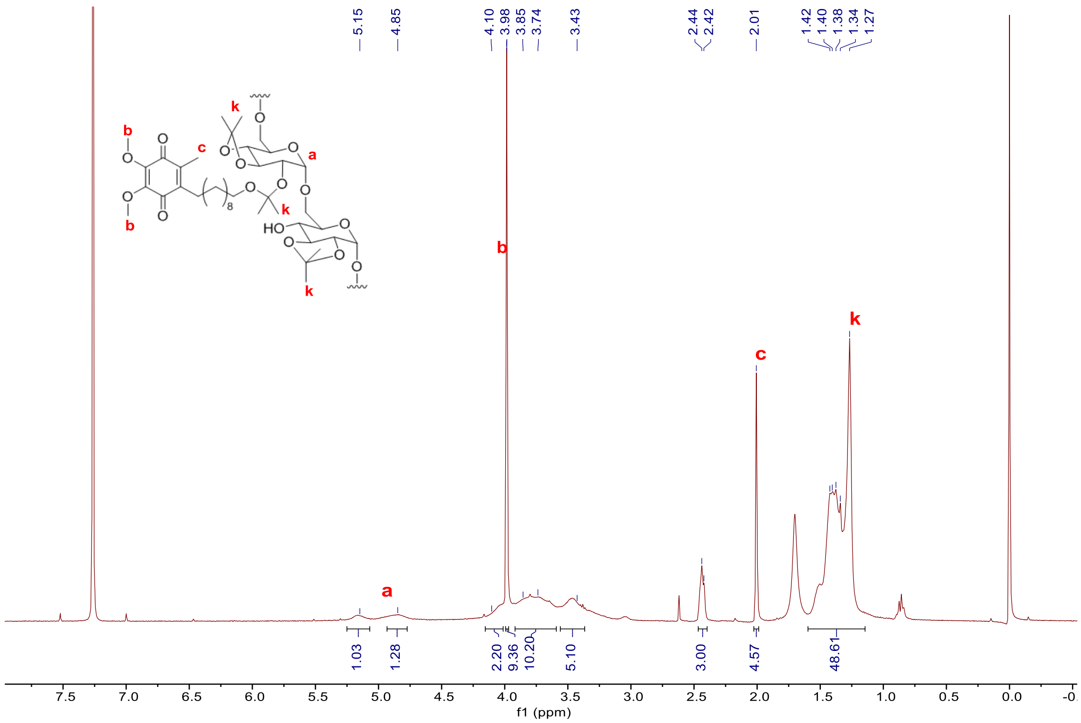

Supplementary Figure 189. ^{1}H NMR spectrum of Idebenone modified dextran with **3k** (400 MHz, $CDCl_3$)

13) Supplementary References

1. Frauchiger, S. & Baiker, A. Saucy–marbet ketonization in a continuous fixed-bed catalytic reactor. *Appl. Catal. A Gen.* **253**, 33-48 (2003).
2. Killian, D. B., Hennion, G. F. & Nieuwland, J. A. The preparation of some α-unsaturated ethers from 2,2-dimethoxyalkanes. *J. Am. Chem. Soc.* **57**, 544-545 (1935).
3. Baum, A. A. & Hennion, G. F. Some reactions of ketals. *J. Am. Chem. Soc.* **60**, 568-571 (1938).
4. Newman, M. S. & Vander Zwan, M. C. Improved synthesis of 2-methoxypropene. *J. Org. Chem.* **38**, 2910-2910 (1973).
5. Wang, Y., Li, H., Wang, C. & Jiang, H. Ionic liquids as catalytic green solvents for cracking reactions. *Chem. Commun.* 1938-1939 (2004).
6. Willmore, N. D., Hoic, D. A. & Katz, T. J. Diels-alder reactions of .Alpha.-substituted styrenes with p-benzoquinone. *J. Org. Chem.* **59**, 1889-1891 (1994).
7. Dujardin, G., Rossignol, S. & Brown, E. Efficient mercury-free preparation of vinyl and isopropenyl ethers of chiral secondary alcohols and α-hydroxyesters. *Tetrahedron Lett.* **36**, 1653-1656 (1995).
8. Hughes, K. D., Nguyen, T.-L. N., Dyckman, D., Dulay, D., Boyko, W. J. & Giuliano, R. M. Synthesis of vinyl glycosides and carbohydrate vinyl ethers from mixed acetals: A hetero-diels–alder approach to deoxygenated disaccharides. *Tetrahedron Asymmetry* **16**, 273-282

(2005).
9. Gassman, P. G., Burns, S. J. & Pfister, K. B. Synthesis of cyclic and acyclic enol ethers (vinyl ethers). *J. Org. Chem.* **58**, 1449-1457 (1993).
10. Miller, R. D. & McKean, D. R. A facile preparation of methyl enol ethers from acetals and ketals using trimethylsilyl iodide. *Tetrahedron Lett.* **23**, 323-326 (1982).
11. Cabrera, G., Fiaschi, R. & Napolitano, E. Triisobutylaluminun (tiba) as a reagent to convert 2,2-dimethoxyalkanes to 2-methoxy-1-alkenes. *Tetrahedron Lett.* **42**, 5867-5869 (2001).
12. Noble, W. J. l. & Crean, P. J. The thermal decarboxylation of β-alkoxycrotonic acids. A new synthesis of isopropenyl ethers. *J. Org. Chem.* **27**, 3875-3878 (1962).
13. Philippo, C. M. G., Vo Nha, H. & Paquette, L. A. Two-carbon intercalation. 4-cyclooctenones by tandem application of double-tebbe and claisen reactions. *J. Am. Chem. Soc.* **113**, 2762-2764 (1991).
14. Adriaenssens, L. V. & Hartley, R. C. Beta-amino acids to piperidinones by petasis methylenation and acid-induced cyclization. *J. Org. Chem.* **72**, 10287-10290 (2007).
15. Tebbe, F. N., Parshall, G. W. & Reddy, G. S. Olefin homologation with titanium methylene compounds. *J. Am. Chem. Soc.* **100**, 3611-3613 (1978).
16. Gillies, E. R., Goodwin, A. P. & Fréchet, J. M. J. Acetals as ph-sensitive linkages for drug delivery. *Bioconjug. Chem.* **15**, 1254-1263 (2004).
17. Teles, J. H., Brode, S. & Chabanas, M. Cationic gold (i) complexes: Highly efficient catalysts for the addition of alcohols to alkynes. *Angew. Chem. Int. Ed.* **37**, 1415-1418 (1998).
18. Veenboer, R. M. P., Dupuy, S. & Nolan, S. P. Stereoselective gold(i)-catalyzed intermolecular hydroalkoxlation of alkynes. *ACS Catal.* **5**, 1330-1334 (2015).
19. Corma, A., Ruiz, V. R., Leyva-Pérez, A. & Sabater, M. J. Regio- and stereoselective intermolecular hydroalkoxylation of alkynes catalysed by cationic gold(i) complexes. *Adv. Synth. Catal.* **352**, 1701-1710 (2010).
20. Iridium-catalyzed synthesis of vinyl ethers from alcohols and vinyl acetate. *Org. Synth.* **82**, (2005).
21. Guo, S. *et al.* Extended release of native drug conjugated in polyketal microparticles. *J. Am. Chem. Soc.* **138**, 6127-6130 (2016).
22. Okimoto, Y., Sakaguchi, S. & Ishii, Y. Development of a highly efficient catalytic method for synthesis of vinyl ethers. *J. Am. Chem. Soc.* **124**, 1590-1591 (2002).
23. Gibson, J. D., Khanal, B. P. & Zubarev, E. R. Paclitaxel-functionalized gold nanoparticles. *J. Am. Chem. Soc.* **129**, 11653-11661 (2007).
24. Baraniak, D., Baranowski, D., Ruszkowski, P. & Boryski, J. 3′-o- and 5′-o-propargyl derivatives of 5-fluoro-2′-deoxyuridine: Synthesis, cytotoxic evaluation and conformational analysis. *Nucleosides Nucleotides Nucleic Acids* **35**, 178-194 (2016).
25. Wappes, E. A., Vanitcha, A. & Nagib, D. A. B c–h di-halogenation via iterative hydrogen atom transfer. *Chem. Sci.* **9**, 4500-4504 (2018).
26. Xu, Y. *et al.* Modular acid-activatable acetone-based ketal-linked nanomedicine by dexamethasone prodrugs for enhanced anti-rheumatoid arthritis with low side effects. *Nano*

Lett. **20**, 2558-2568 (2020).
27. Santos, C. R. *et al.* Structure–activity relationships for dipeptide prodrugs of acyclovir: Implications for prodrug design. *Eur. J. Med. Chem.* **44**, 2339-2346 (2009).
28. Peng, Y. *et al.* Discovery of an orally active and liver-targeted prodrug of 5-fluoro-2′-deoxyuridine for the treatment of hepatocellular carcinoma. *J. Med. Chem.* **59**, 3661-3670 (2016).
29. MacPherson, I. S. *et al.* Multivalent glycocluster design through directed evolution. *Angew. Chem. Int. Ed.* **50**, 11238-11242 (2011).
30. Broaders, K. E., Cohen, J. A., Beaudette, T. T., Bachelder, E. M. & Fréchet, J. M. J. Acetalated dextran is a chemically and biologically tunable material for particulate immunotherapy. *Proc. Natl. Acad. Sci. U. S. A.* **106**, 5497-5502 (2009).
