# Supplementary material for: Modular ketal-linked prodrugs and biomaterials enabled by organocatalytic transisopropenylation of alcohols
Source: Nat Commun. 2021 Sep 20;12:5532. doi: 10.1038/s41467-021-25856-1 (PMC8452702; doi:10.1038/s41467-021-25856-1)
Supplement: Supplementary file 3 — Reporting Summary [file 41467_2021_25856_MOESM3_ESM.pdf]

nature research

Corresponding author(s): Daniel Kohane

Last updated by author(s): Feb 20, 2021

Reporting Summary

Nature Research wishes to improve the reproducibility of the work that we publish. This form provides structure for consistency and transparency in reporting. For further information on Nature Research policies, see our Editorial Policies and the Editorial Policy Checklist.

Statistics

For all statistical analyses, confirm that the following items are present in the figure legend, table legend, main text, or Methods section.

n/a	Confirmed	
☐	☒	The exact sample size (*n*) for each experimental group/condition, given as a discrete number and unit of measurement
☐	☒	A statement on whether measurements were taken from distinct samples or whether the same sample was measured repeatedly
☐	☒	The statistical test(s) used AND whether they are one- or two-sided<br>*Only common tests should be described solely by name; describe more complex techniques in the Methods section.*
☐	☒	A description of all covariates tested
☐	☒	A description of any assumptions or corrections, such as tests of normality and adjustment for multiple comparisons
☐	☒	A full description of the statistical parameters including central tendency (e.g. means) or other basic estimates (e.g. regression coefficient) AND variation (e.g. standard deviation) or associated estimates of uncertainty (e.g. confidence intervals)
☐	☒	For null hypothesis testing, the test statistic (e.g. *F*, *t*, *r*) with confidence intervals, effect sizes, degrees of freedom and *P* value noted<br>*Give P values as exact values whenever suitable.*
☒	☐	For Bayesian analysis, information on the choice of priors and Markov chain Monte Carlo settings
☒	☐	For hierarchical and complex designs, identification of the appropriate level for tests and full reporting of outcomes
☒	☐	Estimates of effect sizes (e.g. Cohen's *d*, Pearson's *r*), indicating how they were calculated

Our web collection on statistics for biologists contains articles on many of the points above.

Software and code

Policy information about availability of computer code

Data collection	No software was used.
Data analysis	GraphPad Prism 7 was used for data analysis and plotting.

For manuscripts utilizing custom algorithms or software that are central to the research but not yet described in published literature, software must be made available to editors and reviewers. We strongly encourage code deposition in a community repository (e.g. GitHub). See the Nature Research guidelines for submitting code & software for further information.

Data

Policy information about availability of data

All manuscripts must include a data availability statement. This statement should provide the following information, where applicable:

- Accession codes, unique identifiers, or web links for publicly available datasets
- A list of figures that have associated raw data
- A description of any restrictions on data availability

The source data underlying Figs. 5a–b, 5e–f, 5h–i, Supplementary Figs. 22, 24–26 and 29–30 are provided as a Source Data file. All data supporting the findings of this study are available within the article and the supplementary information, and are also available from the corresponding author upon reasonable request. Source data are provided with this paper.

Field-specific reporting

Please select the one below that is the best fit for your research. If you are not sure, read the appropriate sections before making your selection.

- [x] Life sciences
- [ ] Behavioural & social sciences
- [ ] Ecological, evolutionary & environmental sciences

For a reference copy of the document with all sections, see nature.com/documents/nr-reporting-summary-flat.pdf

Life sciences study design

All studies must disclose on these points even when the disclosure is negative.

Sample size	Sample sizes are described in each experiment/figure. No statistical methods were used to pre-determine sample sizes but our sample sizes are similar to those reported in previous publications in this field.
Data exclusions	No data were excluded.
Replication	At least 3 independent replicates were taken to successfully verify the reproducibility of the experimental findings.
Randomization	Animals were randomly assigned to one of the experimental groups. The goal of the randomization is to ensure no differences in average and standard deviation. For experiments other than those involving animals, data collection and analysis were carried out on randomly selected samples.
Blinding	Synthesis and material characterization were not blinded, as the results were obtained from the instruments. Animal studies were blinded to one of the two investigators performing the experiments together, and the results were confirmed by the non-blinded investigator. Histological assessments were performed by blinded observer.

Reporting for specific materials, systems and methods

We require information from authors about some types of materials, experimental systems and methods used in many studies. Here, indicate whether each material, system or method listed is relevant to your study. If you are not sure if a list item applies to your research, read the appropriate section before selecting a response.

Materials & experimental systems

n/a	Involved in the study	
☐	☒	Antibodies
☐	☒	Eukaryotic cell lines
☒	☐	Palaeontology and archaeology
☐	☒	Animals and other organisms
☒	☐	Human research participants
☒	☐	Clinical data
☒	☐	Dual use research of concern

Methods

n/a	Involved in the study	
☒	☐	ChIP-seq
☒	☐	Flow cytometry
☒	☐	MRI-based neuroimaging

Antibodies

Antibodies used	α-tubulin (11H10) Rabbit mab (Alexa Fluor® 488 Conjugate) (Cell Signaling Technology, USA) was used.
Validation	Antibody was validated by the manufacturer, purchased from the company and used directly.

Eukaryotic cell lines

Policy information about cell lines

Cell line source(s)	HCT116 human colon cancer cell line: ATCC; 3T3 murine fibroblast cell line; ATCC.
Authentication	Cell lines weren't authenticated by the investigators in this paper. They were purchased from the company.
Mycoplasma contamination	Mycoplasma contamination was tested, and none contamination was found.
Commonly misidentified lines (See ICLAC register)	No commonly misidentified cell lines were used.

Animals and other organisms

Policy information about studies involving animals; ARRIVE guidelines recommended for reporting animal research

Laboratory animals	Female BALB/c nude mice (18-20 g) aged 6 weeks were used.
Wild animals	The study did not involve wild animals.
Field-collected samples	The study did not involve samples collected from the field.
Ethics oversight	All experiments were carried out following the guidelines of the Beijing Laboratory Animal Center, and approved by the Ethical Commission at Nankai University.

Note that full information on the approval of the study protocol must also be provided in the manuscript.
